# Cobalt-catalyzed branched selective hydroallylation of terminal alkynes

**DOI:** 10.1038/s41467-022-32291-3

**Published:** 2022-08-03

**Authors:** Jieping Chen, Jiale Ying, Zhan Lu

**Affiliations:** 1 Center of chemistry for Frontier Technologies, Department of Chemistry, Zhejiang University, Hangzhou, 310058 China; 2 College of Chemistry, Zhengzhou University, Zhengzhou, 450001 China

**Keywords:** Synthetic chemistry methodology, Homogeneous catalysis

## Abstract

Here, we reported a cobalt-hydride-catalyzed Markovnikov-type hydroallylation of terminal alkynes with allylic electrophile to access valuable and branched skipped dienes (1,4-dienes) with good regioselectivity. This operationally simple protocol exhibits excellent functional group tolerance and exceptional substrate scope. The reactions could be carried out in gram-scale with TON (turn over number) up to 1160, and the products could be easily derivatized. The preliminary mechanism of electrophilic allylation of *α*-selective cobalt alkenyl intermediate was proposed based on deuterium labeling experiment and kinetic studies.

## Abstract (web summary)

Selectively generating “skipped” dienes, where two carbon–carbon double bonds are separated by a saturated carbon center, is an interesting problem in organic chemistry, with few reliable, catalytic methods currently available. Here, the authors report branched selective hydroallylation of terminal alkynes with allylic bromides to form skipped dienes, via cobalt catalysis.

## Introduction

**Fig. 1:**
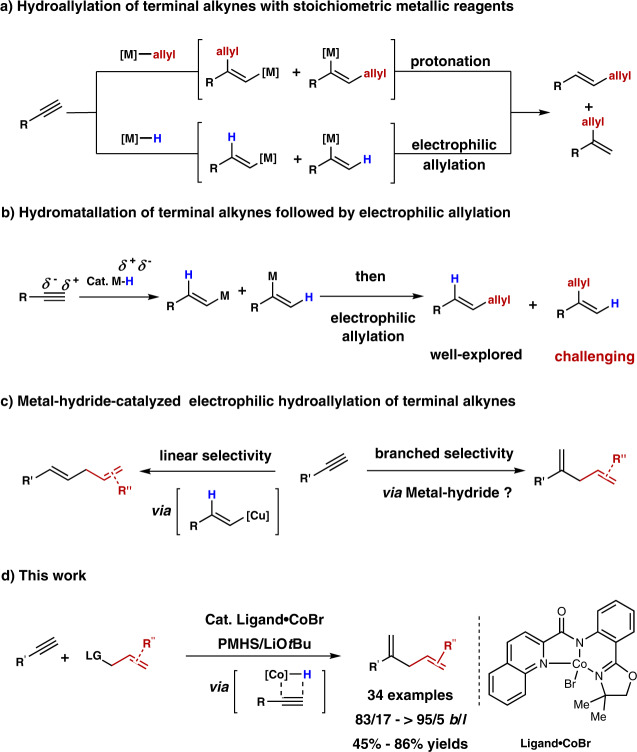
Metal-promoted and metal-hydride-catalyzed regioselective hydroallylation of terminal alkynes. **a** Hydroallylation of terminal alkynes with stoichiometric metallic reagents. **b** Hydromatallation of terminal alkynes followed by electrophilic allylation. **c** Metal-hydride-catalyzed electrophilic hydroallylation of terminal alkynes. **d** Cobalt-catalyzed branched selective hydroallylation of terminal alkynes.

Skipped dienes derivatives are important motifs in biologically active natural products and medicines^1–3^. Traditionally, there are several methods for the preparation of these useful compounds, such as allylation of alkenyl metallic reagents^4–7^, allyl metalation of alkynes^8–12^, and Alder ene reaction of alkynes^13,14^. However, these methods are mainly restricted to using stoichiometric amounts of metallic reagents or limited substrate scope. Compared to currently available methodologies for non-catalytic regioselective allylation of alkynes (Fig. 1a)^8–12,15,16^, metal-catalyzed hydroallylation of alkynes provides another step-economical approach to access skipped dienes. In 1998, Trost and co-workers reported ruthenium-catalyzed addition of alkenes with terminal alkynes to access skipped dienes with excellent functional group tolerance under mild conditions via ruthenacyclopentene intermediate^17,18^. This methodology was developed to assemble complex building blocks rapidly from simple alkenes and alkynes^17–21^. However, the aromatic terminal alkynes were not explored. In 2007, Hilt and co-workers reported cobalt-catalyzed addition of alkenes with internal alkynes to deliver 1,4-dienes with high chemo- and regio-selectivity^22^. However, the terminal alkynes were not suitable due to the preference to form polymerization products^23,24^.

Metal-hydride catalyzed selective electrophilic hydroallylation of terminal alkyne could be considered as an alternative and step-economic method for the synthesis of skipped dienes (Fig. 1b)^25–31^. However, selective electrophilic hydroallylation of terminal alkyne with allylic electrophile via metal hydride strategy is still challenging: (1) Due to the higher reactivity and instability of in-situ generated alkenyl-metal intermediate, its compatibility with other reagents, such as ligand exchange with metal hydride, carbometallation of alkynes, would affect the chemoselectivity of the reaction. (2) Compared to the direct coupling of unactivated alkyl electrophiles with metal-alkenyl intermediate^32–34^, activated allylic electrophiles were prone to process substitution reaction with other nucleophiles, such as in-situ generated metal-hydride intermediate. (3) Due to the weak electronic and steric effects of terminal alkynes, the metal hydride species would like to undergo the *anti*-Markovnikov type insertion rather than Markovnikov type insertion^25–31,35–38^. In 2017, Lalic and co-workers reported an elegant copper-hydride-catalyzed *anti*-Markovnikov type hydroallylation of terminal alkynes for the synthesis of skipped dienes with moderate to excellent regioselectivity (Fig. 1c)^35^. Subsequently, Xiong and Zhang reported one example of *anti*-Markovnikov hydroallylation of terminal alkynes^36^. Recently, Lu and Y. Fu developed a cobalt catalyzed regio- and enantioselective hydroalkylation of fluoroalkenes^37^. To the best of our knowledge, highly branched selective electrophilic hydroallylation of terminal alkyne with allylic electrophile via metal hydride strategy has not been reported. With our continuous interests on base-metal-catalyzed selective hydrofunctionalization reactions (hydrogenation, hydroboration, and hydrosilylation) of unsaturated bond^39–46^, we set out to explore base-metal-catalyzed selective hydroallylation of terminal alkyne.

In this work, we report cobalt-hydride-catalyzed branched selective hydroallylation of terminal alkynes with allylic bromides as electrophiles to access branched terminal skipped dienes with good regioselectivity and excellent functional group tolerance (Fig. 1d).

## Results

### Reaction optimization

We performed the study by using 4-ethynylanisole **1a** as a model substrate, 2-methylallylbromide **2a** as an allylic electrophile, and PMHS as a hydride source (Table 1). The Co(OAc)_2_ was used as a catalyst, *N*-(2-(4,5-dihydrooxazol-2-yl)phenyl)quinoline-2-carboxamide (**L1**) and LiO*t*Bu were used as the ligand and base, respectively. The reaction was performed in a solution of tetrahydrofuran (THF) at 50 °C for 24 h to afford electrophilic hydroallylation product in 42% yield with 83/17 *b*/*l* (**entry 1**). A significant increase in the regioselectivity was observed when a larger *gem*-dimethyl group was used on the oxazoline moiety, which gave rise to skipped diene in 70% yield with >95/5 *rr* (ratio of regioselectivity; **entries 1-3**). However, when the size of the substituent was further increased, the regioselectivity slightly decreased (**entries 4-6**) which implied that the steric hindrance of the substituents on the oxazoline moiety might affect the yield and regioselectivity. Changing the steric effect on pyridine moiety, the selectivity of the reaction decreased slightly (**entries 7-8**). Using various hydrosilane, such as PhSiH_3_, (EtO)_3_SiH, and Ph_2_MeSiH led to poor yield and selectivity (**entries 9-11**). Using Ni(OAc)_2_ instead of Co(OAc)_2_, a poor yield of hydroallylation was observed (**entry 12**). Additionally, Cu(OAc)_2_ could not promote this transformation. However, the Sonogashira coupling reaction of allyl bromides with terminal alkynes could be promoted under mild conditions (**entry 13**) (We should thank one of the reviewers for the suggestion of using nickel or copper catalyst to performing the control experiments.). Using CoBr_2_ instead of Co(OAc)_2_, this transformation could process smoothly (**entry 14**). The model reaction could be completed in 20 min (**entry 15**). The (**L3**-H)•CoBr complex reported in our previous studies^46^ could also be used as an efficient catalyst (**entry 16**). The standard conditions were identified as 1.0 mmol of terminal alkyne, 0.50 mmol of allylic electrophile, 5 mol% of (**L3**-H)•CoBr, 0.75 mmol of LiO*t*Bu, and 0.75 mmol of PMHS in a solution of THF (1 mL) at 50 °C.

**Table 1:** Effect of reaction parameters


Entry^a^	Variations from “standard conditions”	Yield of 3/4 (%)^b^	*α*/*β*^b^
1	**L1**	42	83/17
2	**L2**	40	89/11
3	**L3**	70	>95/5
4	**L4**	55	94/6
5	**L5**	45	91/9
6	**L6**	70	85/15
7	**L7**	83	91/9
8	**L8**	53	92/8
9	**L3**; PhSiH_3_	37	81/19
10	**L3**; (EtO)_3_SiH	14	76/24
11	**L3**; Ph_2_MeSiH	25	88/12
12	**L3**; Ni(OAc)_2_	< 5	–
13	**L3**; Cu(OAc)_2_	–^c^	–
14	**L3**; CoBr_2_	66	>95/5
15	**L3**; 20 min	72	>95/5
16	(**L3**-H)•CoBr; 1 h	75 (69)^d^	>95/5

^a^The reaction wFias conducted using **1a** (1 mmol), **2a** (0.5 mmol), PMHS (0.75 mmol), LiO*t*Bu (0.75 mmol), Co(OAc)_2_ (5 mol %), and ligand (6 mol %) in a solution of THF (1 mL) at 50 °C for 24 h under N_2_; *PMP* *p*-methoxyphenyl, *PMHS* (CH_3_)_3_SiO[(CH_3_)HSiO]_*n*_Si(CH_3_)_3_, *n* = 1.55. ^b^Determined by ^1^H NMR using MeNO_2_ or mesitylene as an internal standard. ^c^41% NMR yield of by-product was observed. ^d^The isolated yield in the parentheses.

### Substrate scope

**Fig. 2:**
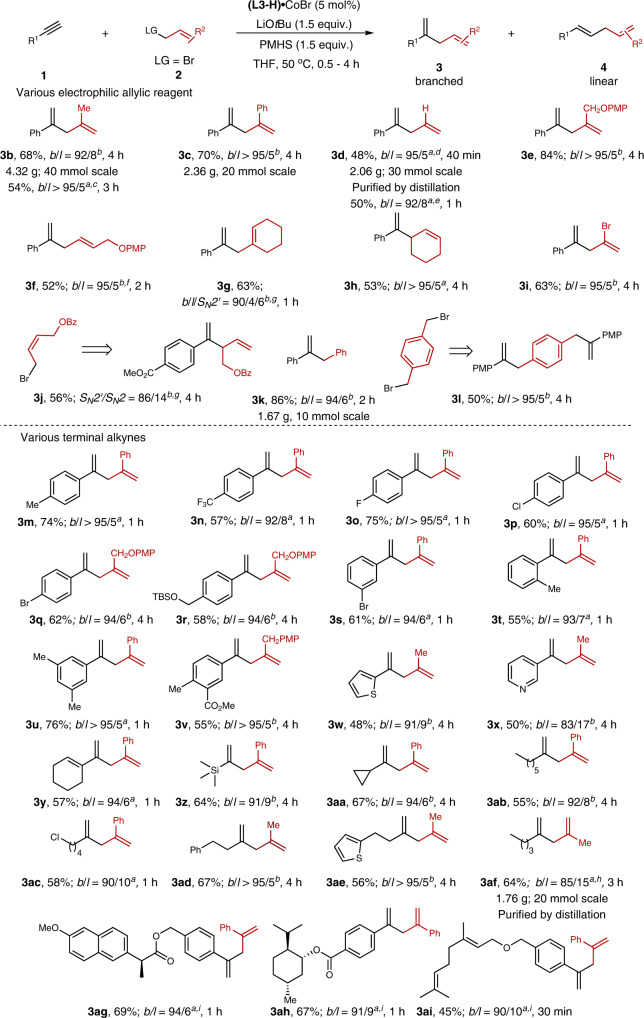
Substrate scope of hydroallylation of terminal alkynes. ^a^Condition A: **1** (2 equiv.), **2** (0.50 mmol), (**L3-**H)•CoBr (5 mol%), PMHS (1.5 equiv.), and LiOtBu (1.5 equiv.) in THF (1 mL) under N_2_ at rt, isolated yield of **3**. ^b^Condition B: **1** (2 equiv.), **2** (0.50 mmol), Co(OAc)_2_ (5 mol%), **L3** (6 mol%), PMHS (1.5 equiv.), and LiO*t*Bu (1.5 equiv.) in THF (1 mL) under N_2_ at rt, isolated yield of **3**. ^c^LG = OP(O)(OPh)_2_ instead of LG = Br; LG means leaving group. ^d^(**L3-**H)•CoBr (2 mol%) instead of (**L3**-H)•CoBr (5 mol%). ^*e*^LG = I instead of LG = Br. ^f^**L7** instead of **L3**; Si(OSiHMe_2_)_4_ instead of PMHS. ^*g*^**L7** instead of **L3**. ^*h*^(**L3-**H)•CoBr (2.5 mol%) instead of (**L3-**H)•CoBr (5 mol%). ^*i*^Si(OSiHMe_2_)_4_ (2.0 equiv.) instead of PMHS; 0.2 mmol scale.

Compared to other olefins, 2,4-disubstituted skipped dienes are difficult to obtain through classic Wittig reaction, due to the keto-enol tautomerism of 1,3-diketone. Thus, with the optimized conditions in hand, we mainly examined the substrate scope of 2,4-disubstituted skipped dienes (Fig. 2). However, due to the presence of Lewis acid metal catalysts and corresponding bases, the inevitable side reaction of terminal alkynes self-polymerization and dehalogenation of allylic bromides would reduce the yield of allylation products. Respectively, **3b** and **3c** could be obtained on gram scale. The volatile dienes (**3d**, **3af**) could also be obtained via distillation on gram-scale. Various allylic bromide (**2d**-**2i**) could be tolerated in this transformation to deliver skipped diene with moderate yield and excellent regioselectivity. *Z*−1,2-disubstituted bromide (**2j**) was also investigated. Interestingly, S_N_2′-type skipped alkene (**3j**) was obtained as a major product in this transformation. The significant regioselectivitity differences between **3** **f** and **3j** might owe to the different *E*/*Z* stereo-configurations and steric hindrance of allyl bromides between **2** **f** and **2j**. Besides allylic bromides, different allylic electrophilic reagents such as allyl iodide and allyl phosphate, could also be tolerated in this transformation with slightly decreased yield and regioselectivity (**3b**, **3d**). Benzyl electrophiles, which exhibit similar properties with allylic reagent, could also be transformed smoothly on gram-scale with excellent regioselectivity^47,48^. The 1,4-bis(bromomethyl)benzene (**2** **l**) could also react with terminal alkynes smoothly to obtained the 1,1-disubstituted alkenes with excellent regioselectivity. For broader synthetic interests, a variety of functional groups on phenyl rings were investigated. Methyl, trifluoromethyl, fluorine, bromine, chlorine, protected alcohol, and ester could be well tolerated to afford the skipped dienes (**3m**-**3v**) in moderate yields with good to excellent regioselectivity (55-76% yield, 92/8 - >95/5 *b*/*l*). The alkynes containing heterocycles, such as pyridine **1w** and thiophene **1x**, could also be tolerated to deliver branched terminal skipped dienes in 48-50% yield. Conjugated enyne (**1** **y**), silyl alkyne (**1z**), and cyclopropyl acetylene (**1aa**) could undergo this hydroallylation reaction smoothly. Simple terminal alkynes (**1ab**-**1ae**) were also amenable to this transformation to deliver the corresponding product in 55-67% yields with 91/9 to >95/5 *rr*. Additionally, terminal alkynes contained in bioactive molecules were investigated. Naproxen, menthol, and geraniol derivative (**1ag-1ai**) could be employed to deliver corresponding products in 45-69% yield.

### Catalytic efficiency and synthetic applications

**Fig. 3:**
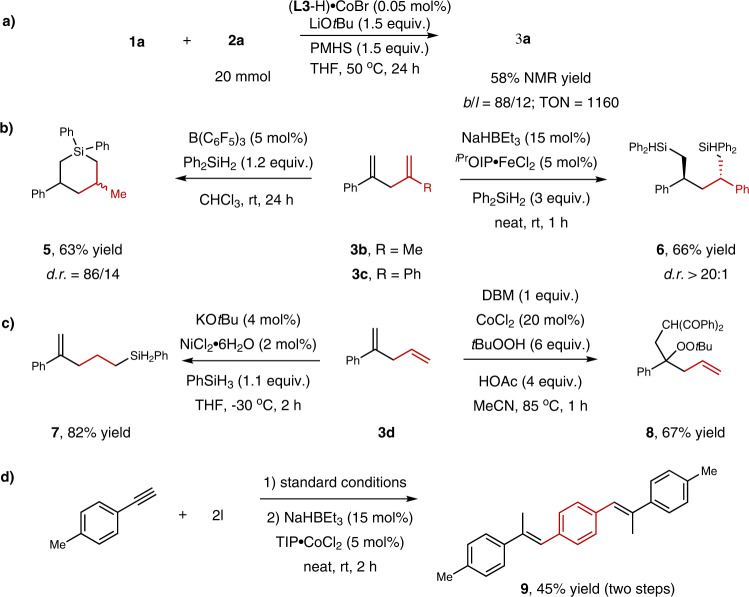
Catalytic efficiency and synthetic applications. **a** The catalytic efficiency of the catalyst. **b** Double hydrosilylation of skipped dienes. **c** Selective conversion of skipped diene’s double bonds. **d** Synthesis of conjugated alkene with AIE properties.

In order to verify the catalytic efficiency of the catalyst, the reaction using 0.05 mol% of catalyst was carried out to afford skipped dienes in 58% yield which indicated that the TON was up to 1160 (Fig. 3a). The 1,4-diene could undergo double hydrosilylation under different conditions to deliver silyl heterocycle **5**^49^ or 1,5-disilyl compound **6**^50^ in 63% and 66% yield respectively (Fig. 3b). Double bonds on skipped diene **3d** could be selectively converted via hydrosilylation reaction to deliver 4-vinyl silane compound **7**^51^. The **3d** could aslo proceed alkylation-peroxidation with 1,3-dicarbonyl compounds and *tert*-butyl hydroperoxide to deliver functionalized carbonyl compound **8** (Fig. 3c)^52^. The reaction of 1,4-bis(bromomethyl)benzene with alkyne under standard conditions could deliver a disubstituted alkene which could be further converted to conjugated trisubstituted alkene **9** via cobalt-catalyzed alkene isomerization (Fig. 3d)^53^. The conjugated alkene displayed promising aggregation-induced emission (AIE) properties^54–56^.

### Mechanistic studies

**Fig. 4:**
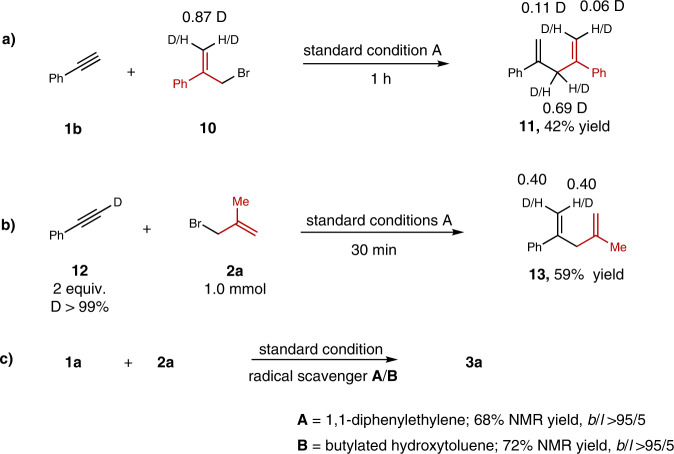
Control experiment. **a** The reaction with deuterium labeling allylic bromide. **b** The reaction with deuterium labeling terminal alkynes. **c** The hydroallylation reaction under additional radical scavenger.

To elucidate the C(*sp*^2^)-C(*sp*^3^) bond forming process in this transformation, control experiments were conducted. 3(*E*)-deuterated allylic bromide **10** was prepared to distinguish substitution at the 1- and 3-positions of the electrophile. This reaction was performed to give the deuterated skipped dienes **11** in 42% yield with 0.17 D at terminal carbon and 0.69 D at *sp*^3^ carbon (Fig. 4a). This result indicated that substitution occurred through the S_N_2′-like process accompanied with the partial S_N_2 process via attack of the postulated cobalt species at the 1,3-position of the allylic bromides. To elucidate the hydrometallation process, the hydroallylation of deuterium labeling phenylacetylene **12** was performed in 30 min to give the deuterated skipped alkene in 59% NMR yield with 0.40 D at C(*sp*^2^) 1(*E*)-position and 0.40 D at C(*sp*^2^) 1(*Z*)-position (Fig. 4b). Due to the presence of strong bases, deuterium atoms might loss during this process. This result combined with our previous mechanistic studies indicated that the *E/Z* ratio of deuterated product might owe to the relatively fast Crabtree-Ojima-type isomerization^42,46^. The reaction could undergo smoothly in the presence of 1,1-diphenylethylene or butylated hydroxytoluene (BHT), which might rule out the radical reaction pathway (Fig. 4c). The hydroallylation product (**3** **g**, **h**, **j**, **k**) showed that the configuration of the allylic bromides would affect the S_N_2 and S_N_2′ selectivity of this transformation. Additionally, the π-allyl pathway could not be exclusively ruled out.

**Fig. 5:**
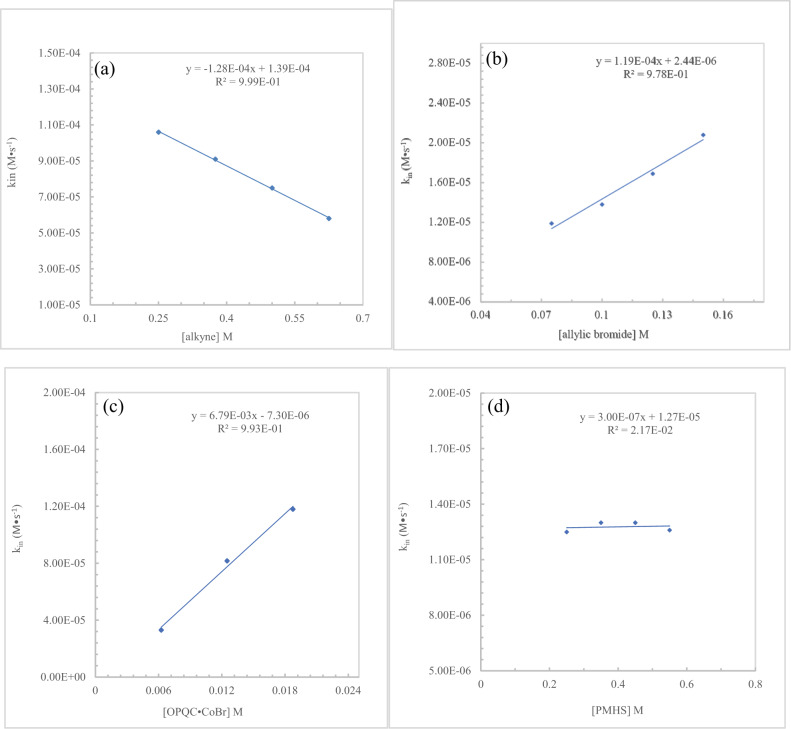
Control experiments. **a** A plot of k_in_ *vs* alkyne concentrations. **b** A plot of k_in_ *vs* allylic bromide concentrations. **c** A plot of k_in_ *vs* catalyst concentrations. **d** A plot of k_in_ *vs* PMHS concentrations.

Quantitative kinetic studies were also performed to determine the roles of alkyne, allylic bromide, hydrosilane, and (**L3**-H)•CoBr complex. Kinetic studies on alkyne showed that with a zero-order rate dependence on alkyne, however, as the concentrations of alkyne increased, the initial rates (k_in_) of the reaction decreased (Fig. 5a). This result demonstrated excessive alkyne might be a ligand to coordinate with cobalt catalyst leading to the reduction of the rate of reaction. Measurements of the initial rates (k_in_) of the reaction with different concentrations of allylic bromide and (**L3**-H)•CoBr complex showed a corresponding rise in the rates of the reactions. Plots of k_in_ versus the concentrations of allylic bromide and (**L3**-H)•CoBr complex (Fig. 5b, c) gave two linear curves (slope = 1.19 × 10^−4^ Ms^−1^; 6.79 × 10^−3^ Ms^−1^), which suggested a first-order rate dependence on allylic bromide and (**L3**-H)•CoBr complex. Similar kinetic studies on PMHS showed no change in k_in_ within a certain concentrations range (Fig. 5d), indicating a zero-order rate dependence on hydrosilane. These quantitative kinetic studies suggests that the nucleophilic substitution of cobalt(II) alkenyl intermediate with allylic bromide could be the turnover-limiting step.

**Fig. 6:**
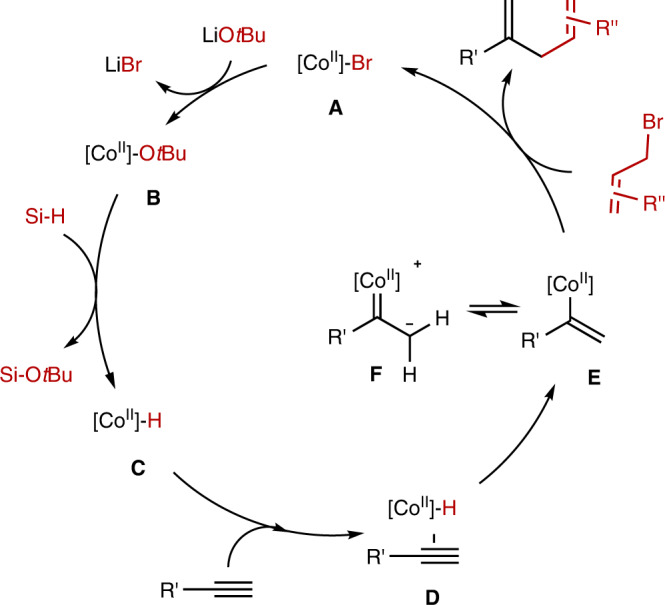
Proposed mechanism. A proposed mechanism of cobalt-hydride catalyzed electrophilic hydroallylation of terminal alkyne.

Based on the experimental studies and previously reported literatures^35,42,46,57–59^, a possible mechanism is shown in Fig. 6. The cobalt hydride species **C** was obtained from the reaction of active intermediate (**L3**-H)•CoBr with LiO*t*Bu and hydrosilane. The alkyne coordination with species **C** followed by the insertion of terminal alkyne into the cobalt hydride bond delivering majorly *α*-selective cobalt-alkenyl intermediate **E**. The quick isomerization balance between **E** and cobalt carbene zwitterion **F** led to the *E/Z* ratio variation based on the deuterium labeling experiment. The following S_N_2′ and S_N_2-like process of **E** with allylic electrophile generates species to deliver the corresponding skipped dienes. The hydrosilane and alkoxide might be likely responsible for the observed regioselectivity increase during the catalysis process.

## Discussion

In summary, we reported an efficient cobalt-hydride catalyzed branched selective electrophilic hydroallylation of terminal alkynes with allylic electrophile to access terminal skipped dienes with good regioselectivity and functional group tolerance under mild conditions. The reaction could be carried out on gram scale, and the TON was up to 1160. The primary mechanism of electrophilic allylation of *α*-selective cobalt alkenyl intermediate was proposed based on deuterium labeling experiment and kinetic studies. Various metal-hydride-catalyzed regioselective hydrofunctionalization of terminal alkynes will be further explored in our laboratory.

## Methods

### Materials

For NMR spectra of compounds in this manuscript, see Supplementary Information. For synthesis of ligands and substrates, see Supplementary Methods. For the optimization of reaction conditions, Supplementary Table 1. For isotopic labeling experiment, radical trapping experiment, and kinetic studies, see Supplementary Figs. 1–18 and Tables 2–9.

### General procedure for hydroallylation of terminal alkynes

A 25 mL Schlenk flask equipped with a magnetic stirrer and a flanging rubber plug was dried with flame under vacuum. When cooled to ambient temperature, it was vacuumed and flushed with N_2_. This degassed procedure was repeated for three times. Then (**L3**-H)•CoBr (0.025 mmol, 5 mol%), THF (1.0 mL, 0.5 M), PMHS (0.75 mmol, 1.5 equiv.), terminal alkynes (1.0 mmol, 2 equiv.), allylic bromides (0.5 mmol, 1.0 equiv.), and LiO*t*Bu (0.75 mmol, 1.5 equiv.) were added sequentially. The reaction was run at 50 °C for 30 min to 4 h. Then the resulting solution was quenched with 10 mL of PE and filtered through a pad of silica gel, washed with PE/EtOAc (5/1) (3 × 20 mL). The combined filtrate was concentrated under vacuum and the ratio of *b*/*l* was monitored by ^1^H NMR analysis. The mixture was purified by flash column chromatography to give the corresponding product.

## Supplementary Information

Supplementary Information

## Data Availability

The authors declare that the data Supplementary the findings of this study are available within the paper and its Supplementary Information file. The experimental procedures and characterization of all new compounds are provided in the Supplementary Information.
