# Supplementary material for: Cobalt-catalyzed branched selective hydroallylation of terminal alkynes
Source: Nat Commun. 2022 Aug 3;13:4518. doi: 10.1038/s41467-022-32291-3 (PMC9349270; doi:10.1038/s41467-022-32291-3)
Supplement: Supplementary file 1 — Supplementary Information [file 41467_2022_32291_MOESM1_ESM.pdf]

Supplementary Information

Cobalt-Catalyzed Branched Selective Hydroallylation of Terminal Alkynes

Jieping Chen,[1] Jiale Ying,[1] and Zhan Lu[1,2]*

[1]*Center of chemistry for Frontier Technologies, Department of Chemistry, Zhejiang University, Hangzhou 310058, China.*

[2]*College of Chemistry, Zhengzhou University, Zhengzhou 450001, China.*

Correspondence and requests for materials should be addressed to Z.L.

(email: luzhan@zju.edu.cn)

Table of contents

Supplementary Methods 3

I. General Information 3

II. Synthesis of Ligands 3

III. Synthesis of Substrates 7

IV. Cobalt-Catalyzed Branched Selective Hydroallylation of Terminal Alkynes 9

V. General Procedure for Hydroallylation of Terminal Alkynes: 9

VI. Gram Scale Reaction and synthetic applications 28

VII. Mechanistic studies and Control experiments 32

VIII. Kinetic Studies 34

IX. Unsuccessful substrates 41

X. NMR Spectra 42

Supplementary References 145

Supplementary Methods

I. General Information

THF was distilled from sodium benzophenone ketyl prior to use. $Co(OAc)_2$ (99.99%) was purchased from Aladdin and used as received. LiO*t*Bu and Poly(methylhydrosiloxane) (PMHS) were purchased from Energy-Chemical. NMR spectra were recorded on Bruker-400 instrument, Oxford-400 instrument and Agilent-600 instrument. ^{1}H NMR chemical shifts were referenced to tetramethylsilane signal (0 ppm). ^{13}C NMR chemical shifts were referenced to the solvent resonance (77.00 ppm, $CDCl_3$). The following abbreviations (or combinations) were used to explain multiplicities: s = singlet, d = doublet, t = triplet, m = multiplet, br = broad, q = quadruplet. High-resolution mass spectra (HRMS) were recorded on ESI-TOF, EI-TOF, and Waters GCT Premier (GC-TOF). IR spectra were recorded on a Perkin-Elmer Spectrum One FTIR spectrometer with diamond ATR accessory.

II. Synthesis of Ligands

L3[1] and **L8**[2] were prepared according to the previously reported procedures.

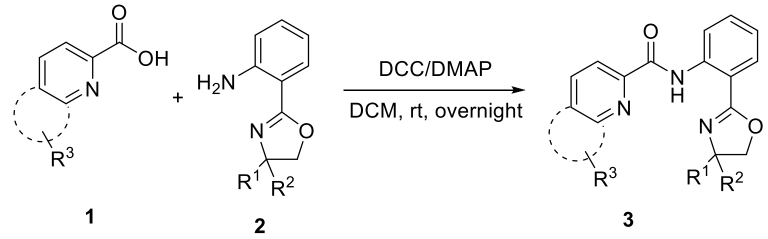

General Procedure: A 100 mL oven-dried round-bottom flask was charged with amine **2**, acid **1**, DCM, and DMAP in sequentially. Then DCC was added slowly to this reaction mixture and stirred at room temperature for overnight. The resulting solution was filtered through a pad of silica gel, concentrated in vacuo, and purified by column chromatography using PE/EA = 10/1 as the eluent to give the corresponding product.

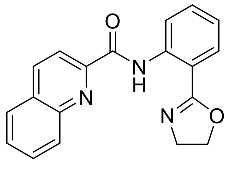

N-(2-(4,5-dihydrooxazol-2-yl)phenyl)quinoline-2-carboxamide (**L1**)

Prepared according to general procedure using quinaldic acid (0.61 g, 3.5 mmol), 2-(4,5-dihydrooxazol-2-yl)aniline (0.52 g, 3.0 mmol),

DMAP (0.0379 g, 0.3 mmol), DCC (1.23 g, 6.0 mmol), and DCM (10 mL). After overnight, the reaction mixture filtered through a pad of silica gel with DCM, concentrated in vacuo, and purified by column chromatography using PE/EA (10/1) as the eluent to afford **L1** (0.64 g, 2.0 mmol, 67% yield) as a white solid. m.p. 174 - 175 °C; IR (neat, cm^{-1}): 2931, 1678, 1644, 1582, 1503, 1449; ^{1}H NMR: (400 MHz, $CDCl_3$) δ 14.10 (brs, 1H), 9.09 (d, J = 8.4 Hz, 1H), 8.42-8.31 (m, 2H), 8.22 (d, J = 8.4 Hz, 1H), 7.97-7.89 (m, 2H), 7.84-7.78 (m, 1H), 7.68-7.62 (m, 1H), 7.58-7.52 (m, 1H), 7.18-7.12 (m, 1H), 4.53-4.38 (m, 4H); ^{13}C NMR: (100 MHz, $CDCl_3$) δ 164.3, 164.1, 150.8, 146.6, 139.5, 137.5, 132.4, 130.02, 129.98, 129.4, 129.3, 128.0, 127.7, 122.7, 120.0, 119.2, 114.6, 66.3, 55.0; HRMS (ESI) calculated for $[C_{19}H_{16}N_3O_2]^+$ (M+H$^+$) requires m/z 318.1237, found m/z 318.1238.

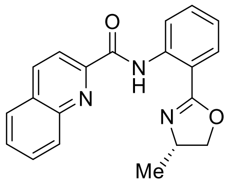

(*S*)-*N*-(2-(4-methyl-4,5-dihydrooxazol-2-yl)phenyl)quinoline-2-carboxamide (**L2**)

Prepared according to general procedure using quinaldic acid (0.54 g, 3.1 mmol), (*S*)-2-(4-methyl-4,5-dihydrooxazol-2-yl)aniline (0.61 g, 3.4 mmol), DMAP (0.0379 g, 0.3 mmol), DCC (1.50 g, 7.3 mmol), and DCM (10 mL). After overnight, the reaction mixture filtered through a pad of silica gel with DCM, concentrated in vacuo, and purified by column chromatography using PE/EA (10/1) as the eluent to afford **L2** (0.62 g, 1.89 mmol, 63% yield) as a white solid. m.p. 172 - 174 °C; IR (neat, cm^{-1}):3076, 2975, 2928, 1668, 1640, 1579, 1527; ^{1}H NMR: (400 MHz, $CDCl_3$) δ 14.00 (brs, 1H), 9.08 (d, J = 8.8 Hz, 1H), 8.42-8.33 (m, 2H), 8.24 (d, J = 8.8 Hz, 1H), 7.96-7.89 (m, 2H), 7.84-7.78 (m, 1H), 7.68-7.62 (m, 1H), 7.58-7.52 (m, 1H), 7.19-7.13 (m, 1H), 4.78-4.67 (m, 1H), 4.56 (t, J = 8.0 Hz, 1H), 4.00 (t, J = 8.0 Hz, 1H), 1.57 (d, J = 6.8 Hz, 3H); ^{13}C NMR: (100 MHz, $CDCl_3$) δ 164.3, 162.9, 151.0, 146.7, 139.5, 137.4, 132.3, 130.0, 129.8, 129.3, 129.2, 127.9, 127.8, 122.7, 120.1, 119.3, 114.7, 72.7, 62.4, 21.8; HRMS (ESI) calculated for $[C_{20}H_{18}N_3O_2]^+$ (M+H$^+$) requires m/z 332.1394, found m/z 332.1395.

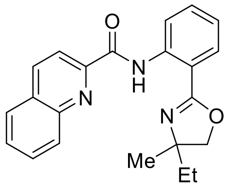

N-(2-(4-ethyl-4-methyl-4,5-dihydrooxazol-2-yl)phenyl)quinoline-2-carboxamide (**L4**)

Prepared according to general procedure using quinaldic acid

(0.2692 g, 1.6 mmol), 2-(4-ethyl-4-methyl-4,5-dihydrooxazol-2-yl)aniline (0.2873 g, 1.4 mmol), DMAP (0.0172 g, 0.15 mmol), DCC (0.60 g, 2.9 mmol), and DCM (5 mL). After overnight, the reaction mixture filtered through a pad of silica gel with DCM, concentrated in vacuo, and purified by column chromatography using PE/EA (15/1) as the eluent to afford **L4** (0.25 g, 0.66 mmol, 47% yield) as a white solid. m.p. 86 - 87 °C; IR (neat, cm^{-1}): 3064, 2968, 2928, 1678, 1642, 1602, 1504; ^{1}H NMR: (400 MHz, $CDCl_3$) δ 13.81 (brs, 1H), 9.05 (d, J = 8.4 Hz, 1H), 8.40 (d, J = 8.4 Hz, 1H), 8.35 (d, J = 8.4 Hz, 1H), 8.24 (d, J = 8.8 Hz, 1H), 7.95-7.89 (m, 2H), 7.84-7.78 (m, 1H), 7.68-7.62 (m, 1H), 7.58-7.50 (m, 1H), 7.16 (dd, J = 8.4, 8.4 Hz, 1H), 4.22 (d, J = 8.4 Hz, 1H), 4.04 (d, J = 8.4 Hz, 1H), 1.95-1.75 (m, 2H), 1.56 (s, 3H), 0.93 (t, J = 7.6 Hz, 3H); ^{13}C NMR: (100 MHz, $CDCl_3$) δ 164.4, 161.3, 151.1, 146.8, 139.5, 137.3, 132.1, 129.9, 129.8, 129.2, 127.9, 127.8, 122.7, 120.5, 119.4, 115.1, 75.4, 71.7, 33.9, 27.1, 8.5; HRMS (ESI) calculated for $[C_{22}H_{22}N_3O_2]^+$ ($M+H^+$) requires m/z 360.1707, found m/z 360.1708.

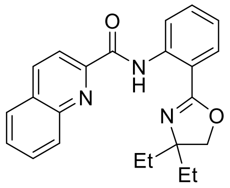

N-(2-(4,4-diethyl-4,5-dihydrooxazol-2-yl)phenyl)quinoline-2-carboxamide (**L5**)

Prepared according to general procedure with modification using quinaldic acid (0.0893 g, 0.5 mmol), 2-(4,4-diethyl-4,5-dihydrooxazol-2-yl)aniline (0.1092 g, 0.5 mmol), DMAP (0.0090 g, 0.06 mmol), DCC (0.1715 g, 0.85 mmol), and DCM (5 mL). After overnight, the reaction mixture filtered through a pad of silica gel with DCM, concentrated in vacuo, and purified by column chromatography using PE/EA (10/1) as the eluent to afford **L5** (0.13 g, 0.38 mmol, 77% yield) as a white solid. m.p. 87 - 88 °C; IR (neat, cm^{-1}): 3067, 2967, 1678, 1643, 1601, 1505; ^{1}H NMR: (400 MHz, $CDCl_3$) δ 13.77 (brs, 1H), 9.06-9.00 (m, 1H), 8.40 (d, J = 8.4 Hz, 1H), 8.34 (d, J = 8.4 Hz, 1H), 8.25 (d, J = 8.4 Hz, 1H), 7.95-7.89 (m, 2H), 7.84-7.77 (m, 1H), 7.68-7.62 (m, 1H), 7.58-7.51 (m, 1H), 7.19-7.12 (m, 1H), 4.13 (s, 2H), 1.96-1.74 (m, 4H), 0.93 (t, J = 7.2 Hz, 6H); ^{13}C NMR: (100 MHz, $CDCl_3$) δ 164.5, 161.3, 151.2, 146.8, 139.5, 137.2, 132.0, 129.9, 129.27, 129.25, 127.9, 127.8, 122.7, 120.6, 119.5, 115.2, 75.0, 72.9, 32.1, 8.3; HRMS (ESI) calculated for $[C_{23}H_{23}N_3NaO_2]^+$ ($M+Na^+$) requires m/z 396.1682, found m/z 396.1680.

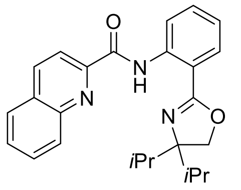

N-(2-(4,4-diisopropyl-4,5-dihydrooxazol-2-yl)phenyl)quinoline-2-carboxamide (**L6**)

Prepared according to gerenal procedure with modification using quinaldic acid (0.26 g, 1.5 mmol), 2-(4,4-diisopropyl-4,5-dihydrooxazol-2-yl)aniline (0.37 g, 1.5 mmol), DMAP (0.0100 g, 0.15 mmol), DCC (0.51 g, 2.5 mmol) and DCM (10 mL). After overnight, the reaction mixture filtered through a pad of silica gel with DCM, concentrated in vacuo, and purified by column chromatography using PE/EA (10/1) as the eluent to afford **L6** (0.30 g, 0.75 mmol, 50% yield) as a white solid. m.p. 88 - 89 °C; IR (neat, cm^{-1}): 3065, 2967, 2877, 1679, 1645, 1583, 1504; ^{1}H NMR: (400 MHz, $CDCl_3$) δ 13.63 (brs, 1H), 8.98 (d, *J* = 8.8 Hz, 1H), 8.40 (d, *J* = 8.8 Hz, 1H), 8.34 (d, *J* = 8.8 Hz, 1H), 8.24 (d, *J* = 8.8 Hz, 1H), 7.95-7.87 (m, 2H), 7.82-7.76 (m, 1H), 7.64 (dd, *J* = 7.6, 7.6 Hz, 1H), 7.58-7.51 (m, 1H), 7.16 (dd, *J* = 7.6, 7.6 Hz, 1H), 4.11 (s, 2H), 2.35-2.25 (m, 2H), 0.97 (d, *J* = 6.8 Hz, 6H), 0.86 (d, *J* = 6.8 Hz, 6H); ^{13}C NMR: (100 MHz, $CDCl_3$) δ 164.6, 160.8, 151.3, 146.8, 139.5, 137.3, 131.9, 130.0, 129.8, 129.3, 129.2, 127.84, 127.78, 122.8, 120.9, 119.6, 115.1, 80.6, 69.1, 32.6, 17.4, 17.0; HRMS (ESI) calculated for $[C_{25}H_{28}N_3O_2]^+$ ($M+H^+$) requires m/z 402.2176, found m/z 402.2178.

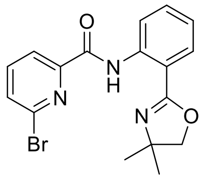

6-Bromo-*N*-(2-(4,4-dimethyl-4,5-dihydrooxazol-2-yl)phenyl)picolinamide (**L7**)

Prepared according to general procedure with modification using 6-bromopicolinic acid (1.12 g, 5.0 mmol), 2-(4,4-dimethyl-4,5-dihydrooxazol-2-yl)aniline (0.83 g, 5.0 mmol), DMAP (0.0682 g, 0.55 mmol), DCC (1.60 g, 7.5 mmol), and DCM (20 mL). After overnight, the reaction mixture filtered through a pad of silica gel with DCM, concentrated in vacuo, and purified by column chromatography using PE/EA (15/1) as the eluent to afford **L7** (1.77 g, 4.50 mmol, 90% yield) as a white solid. m.p. 124 - 125 °C; IR (neat, cm^{-1}): 3085, 2967, 1680, 1643, 1581, 1448; ^{1}H NMR: (400 MHz, $CDCl_3$) δ 13.75 (brs, 1H), 8.99 (d, *J* = 8.4 Hz, 1H), 8.27 (d, *J* = 7.6 Hz, 1H), 7.91 (dd, *J* = 1.2, 8.0 Hz, 1H), 7.75 (dd, *J* = 8.0, 8.0 Hz, 1H), 7.65 (d, *J* = 8.0 Hz, 1H), 7.55-7.48 (m, 1H), 7.18-7.12 (m, 1H), 4.10 (s, 2H), 1.57 (s, 6H); ^{13}C NMR: (100 MHz, $CDCl_3$) δ 162.5, 161.1, 152.2, 140.7, 139.5, 139.2, 132.1, 130.7, 129.2, 123.0, 122.0, 120.3, 115.0, 78.0, 68.5, 28.5; HRMS (ESI) calculated for

$[C_{17}H_{17}BrN_3O_2]^+$ ($M+H^+$) requires m/z 374.0499, found m/z 374.0501.

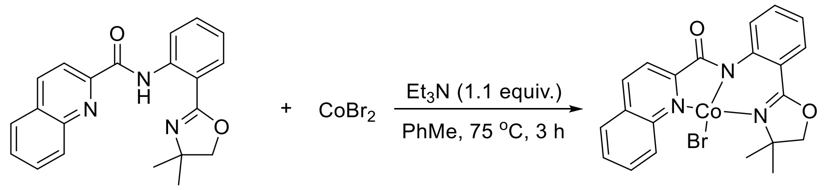

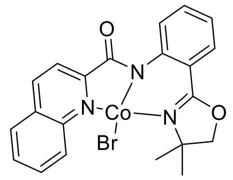

(**L3**-H)•CoBr.

To a 25 mL flame-dried Schlenk flask cooled under nitrogen, $CoBr_2$ (0.6562 g, 3.0 mmol), *N*-(2-(4,4-dimethyl-4,5-dihydrooxazol-2-yl)phenyl)quinoline-2-carboxamide (1.0880 g, 3.15 mmol), Et_3N (459 μL, 3.3 mmol), and dry toluene (10 mL) were added. The mixture was stirred at 75 °C for 3 h. The resulting solid was filtered and the cake was transferred to round-bottomed flask. Then the cake was washed with H_2O under stirring, and the residual solid was filtered. Repeat the above experimental operation 3 times. The obtained solid was dried in vacuo using toluene as azeotropic solvent to remove the residual water. The complex was obtained with 80% yield (1.16 g, 2.4 mmol) as a brown powder. Anal. Calcd for $C_{21}H_{18}BrCoN_3O_2$ + 0.5 H_2O: C, 51.24; H, 3.89; N, 8.54; Found: C, 51.57; H, 3.78; N, 8.63.

III. Synthesis of Substrates

1a, **1b**, **1n**, **1o**, **1u**, **1w**, **1x**, **1y**, **1z**, **1aa**, **1ab**, **1ac**, and **1af** were used as received. **1m**[3], **1p**[4], **1q**[3], **1r**[5], **1s**[3], **1t**[4], **1u**[6], **1v**[7], **1ad**[8], **1ae**[8], and **1ah**[9] were synthesized via procedure and the spectroscopic data were agreement with the reported ones. **2a**, **2b**, **2i**, **2k,** and **2l** were purchased from Energy-Chemical. **2c**[10], **2e**[11], **2f**[12], **2g**[13], **2h**[14], and **2j**[15] were synthesized via procedure and the spectroscopic data were agreement with the reported ones.

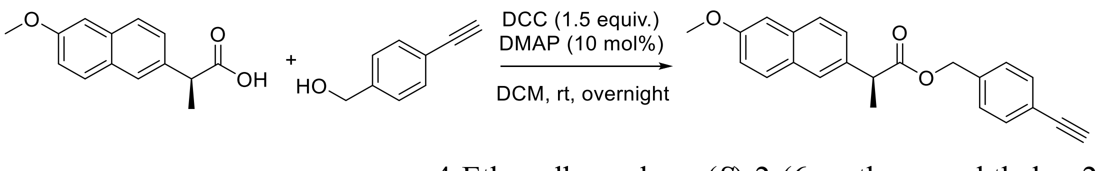

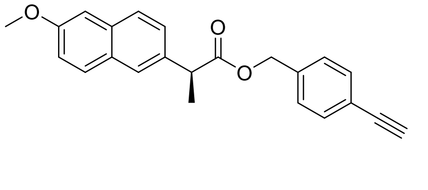

4-Ethynylbenzyl (*S*)-2-(6-methoxynaphthalen-2-yl)propanoate (**1ag**)

Prepared according to the previously reported

procedures[16] using (*S*)-2-(6-methoxynaphthalen-2-yl)propanoic acid and (4-ethynylphenyl)methanol as starting materials. IR (neat, cm^{-1}): 3284, 2977, 2938, 2839, 1732, 1634, 1507; ^{1}H NMR: (400 MHz, $CDCl_3$) δ 7.72-7.62 (m, 3H), 7.42-7.35 (m, 3H), 7.19-7.10 (m, 4H), 5.10 (d, J = 2.8 Hz, 2H), 3.95-3.87 (m, 4H), 3.06 (s, 1H), 1.58 (d, J = 7.2 Hz, 3H); ^{13}C NMR: (100 MHz, $CDCl_3$) δ 174.3, 157.6, 136.7, 135.3, 133.7, 132.2, 129.2, 128.9, 127.7, 127.2, 126.2, 126.0, 121.8, 119.0, 105.5, 83.2, 77.5, 65.8, 55.3, 45.4, 18.4; HRMS (ESI) calculated for $[C_{23}H_{20}NaO_3]^+$ ($M+Na^+$) requires m/z 367.1305, found m/z 367.1304.

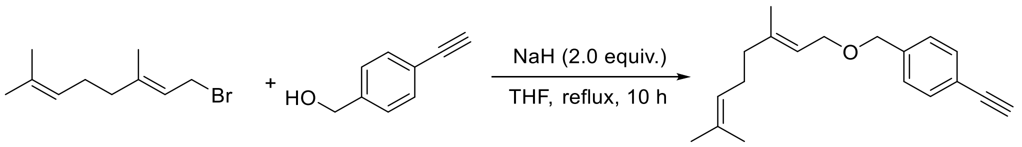

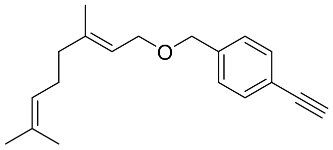

(*E*)-1-(((3,7-dimethylocta-2,6-dien-1-yl)oxy)methyl)-4-ethynylbenzene (**1ai**)

Prepared according to the previously reported procedures[16] using geranyl bromide and (4-ethynylphenyl)methanol as starting materials. IR (neat, cm^{-1}): 3290, 2968, 2915, 2854, 1914, 1735, 1608; ^{1}H NMR: (400 MHz, $CDCl_3$) δ 7.47 (d, J = 8.4 Hz, 2H), 7.30 (d, J = 8.4 Hz, 2H), 5.45-5.35 (m, 1H), 5.15-5.05 (m, 1H), 4.49 (s, 2H), 4.03 (d, J = 6.8 Hz, 2H), 3.06 (s, 1H), 2.15-2.00 (m, 4H), 1.68 (s, 3H), 1.64 (s, 3H), 1.60 (s, 3H); ^{13}C NMR: (100 MHz, $CDCl_3$) δ 140.6, 139.5, 132.1, 131.6, 127.5, 123.9, 121.1, 120.5, 83.5, 71.3, 66.6, 39.5, 26.3, 25.7, 17.6, 16.4; HRMS (ESI) calculated for $[C_{19}H_{24}NaO]^+$ ($M+Na^+$) requires m/z 291.1719, found m/z 291.1721.

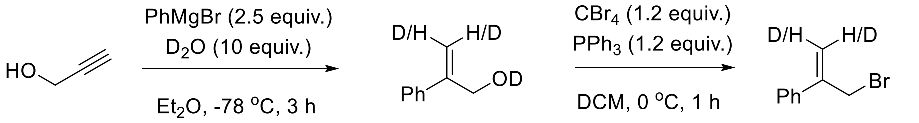

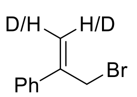

(*Z*)-(3-bromoprop-1-en-2-yl-1-d)benzene (**10**)

Prepared according to the previously reported procedures[17,18] using Phenylmagnesium bromide, Deuterium oxide, carbon tetrabromide, and triphenylphosphine as starting materials. ^{1}H NMR: (400 MHz, $CDCl_3$) δ 7.52-7.46 (m, 2H), 7.41-7.30 (m, 3H), 5.57-5.53 (m, 0.90H), 5.51-5.48 (m, 0.23H), 4.41-4.36 (m, 2H).

IV. Cobalt-Catalyzed Branched Selective Hydroallylation of Terminal Alkynes

Supplementary Table 1. Effect of Reaction Parameters.[a]

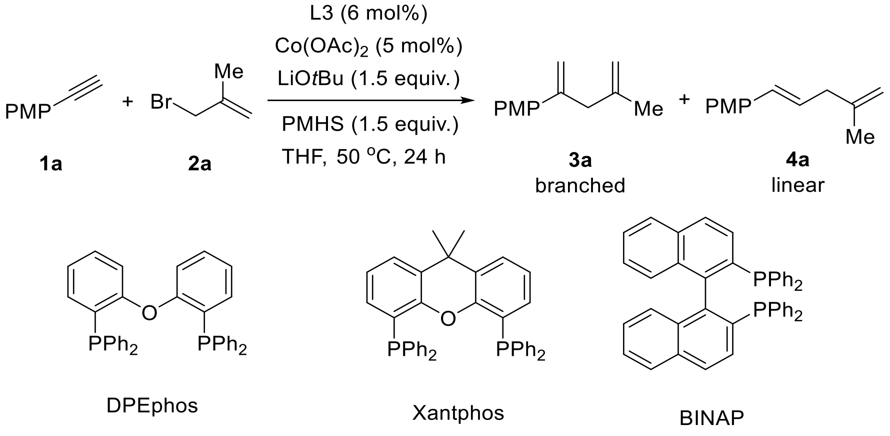

entry	variation from "standard conditions"	yield of **3**/**4** (%)[b]	*b*/*l*[b]
1	Li_2CO_3 instead of LiO*t*Bu	-	-
2	LiOH instead of LiO*t*Bu	57	94/6
3	LiOMe instead of LiO*t*Bu	31	93/7
4	TDMS instead of PMHS	70	95/5
5	Et_3SiH instead of PMHS	-	-
6	dioxane instead of THF	55	93/7
7	PhMe instead of THF	25	90/10
8	30 °C instead of 50 °C	61	>95/5
9	$CoCl_2$ instead of $Co(OAc)_2$	68	>95/5
10	CoI_2 instead of $Co(OAc)_2$	65	>95/5
11	DPEPhos instead of **L3**	7	-
12	XantPhos instead of **L3**	6	-
13	BINAP instead of **L3**	6	-

[a]The reaction was conducted using **1a** (1 mmol), **2a** (0.5 mmol), PMHS (0.75 mmol), LiO*t*Bu (0.75 mmol), $Co(OAc)_2$ (5 mol %), and ligand (6 mol %) in a solution of THF (1 mL) at 50 °C for 24 h under N_2; PMP = *p*-methoxyphenyl; PMHS = $(CH_3)_3SiO[(CH_3)HSiO]_nSi(CH_3)_3$, n =1.55. [b]Determined by ^1H NMR using $MeNO_2$ or mesitylene as an internal standard.

V. General Procedure for Hydroallylation of Terminal Alkynes:

General Procedure A: A 25 mL Schlenk flask equipped with a magnetic stirrer and a

flanging rubber plug was dried with flame under vacuum. When cooled to ambient temperature, it was vacuumed and flushed with N_2. This degassed procedure was repeated for three times. Then (**L3**-H)•CoBr (0.025 mmol, 5 mol %), THF (1.0 mL, 0.5 M), PMHS (0.75 mmol, 1.5 equiv.), terminal alkynes (1.0 mmol, 2 equiv.), allylic bromides (0.5 mmol, 1.0 equiv.), and LiO*t*Bu (0.75 mmol, 1.5 equiv.) were added sequentially. The reaction was run at 50 °C for 30 min to 4 h. Then the resulting solution was quenched with 10 mL of PE and filtered through a pad of silica gel, washed with PE/EtOAc (5/1) (3 x 20 mL). The combined filtrate was concentrated under vacuum and the ratio of *b*/*l* was monitored by ^{1}H NMR analysis. The mixture was purified by flash column chromatography to give the corresponding product.

General Procedure B: A 25 mL Schlenk flask equipped with a magnetic stirrer and a flanging rubber plug was dried with flame under vacuum. When cooled to ambient temperature, it was vacuumed and flushed with N_2. This degassed procedure was repeated for three times. Then ligand (0.030 mmol, 6 mol %), $Co(OAc)_2$ (0.025 mmol, 5 mol%), and THF (1 mL, 0.5 M) were added sequentially. The mixture was stirred for 30 min. Then PMHS (0.75 mmol, 1.5 equiv.), terminal alkynes (1.0 mmol, 2 equiv.), allylic bromides (0.5 mmol, 1.0 equiv.), and LiO*t*Bu (0.75 mmol) were added sequentially. The reaction was run at 50 °C for 30 min to 4 h. The resulting solution was quenched with 10 mL of PE and filtered through a pad of silica gel, washed with PE/EtOAc (5/1) (3 x 20 mL). The combined filtrate was concentrated under vacuum and the ratio of *b*/*l* was monitored by ^{1}H NMR analysis. The mixture was purified by flash column chromatography to give the corresponding product.

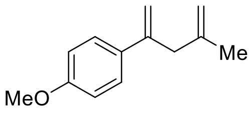

1-Methoxy-4-(4-methylpenta-1,4-dien-2-yl)benzene (**3a**)

Prepared according to the general procedure A using 0.0047 g (0.025 mmol) of (**L3**-H)•CoBr, 180 μL (0.75 mmol) of PMHS, 130 μL (1.019 g/mL, 1 mmol) of 1-ethynyl-4-methoxybenzene, 53 μL (1.339 g/mL, 0.5 mmol) of 3-bromo-2-methylpropene, 0.0602 g (0.75 mmol) of LiO*t*Bu, and 1.0 mL (0.5 M) of THF. After 1 h, the reaction was worked up. The combined filtrate was

concentrated and the regioselectivity (*b*/*l* > 95/5) was monitored by ^{1}H NMR analysis. The mixture was purified by flash column chromatography using PE to PE/EA (200/1) as the eluent to give 0.0650 g (0.35 mmol, 69% yield) of the title compound as a colorless oil. IR (neat, cm^{-1}): 3074, 2963, 2840, 1670, 1604, 1510, 1453; ^{1}H NMR: (400 MHz, $CDCl_3$) δ 7.40-7.36 (m, 2H), 6.87-6.82 (m, 2H), 5.36 (d, J = 1.2 Hz, 1H), 5.04-5.00 (m, 1H), 4.80 (s, 1H), 4.76 (s, 1H), 3.80 (s, 3H), 3.19 (s, 2H), 1.71 (s, 3H); ^{13}C NMR: (100 MHz, $CDCl_3$) δ 158.9, 144.8, 143.6, 133.3, 127.1, 113.4, 112.7, 112.4, 55.1, 44.0, 22.2; HRMS (EI) calculated for $[C_{13}H_{16}O]^+$ (M^+) requires m/z 188.1201, found m/z 188.1199.

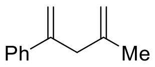

(4-Methylpenta-1,4-dien-2-yl)benzene (**3b**)

(a) Prepared according to the general procedure B using 0.3243 g (2.0 1mmol) of $Co(OAc)_2$, 0.8126 g (2.4 mmol) of **L3**, 14.4 mL (60.0 mmol) of PMHS, 8.8 mL (0.930 g/mL, 80.0 mmol) of phenylacetylene, 4.1 mL (1.339 g/mL, 40.0 mmol) of 3-bromo-2-methylpropene, 4.76 g (60.0 mmol) of LiO*t*Bu, and 80.0 mL (0.5 M) of THF. After 4 h, the reaction was worked up. The combined filtrate was concentrated and the regioselectivity (*b*/*l* = 92/8) was monitored by ^{1}H NMR analysis. The crude mixture was purified by flash column chromatography using hexane as the eluent to give 4.32 g (27.2 mmol, 68% yield) of the title compound as a colorless oil.

(b) Prepared according to the general procedure A using 0.0125 g (0.025 mmol) of (**L3**-H)•CoBr, 180 μL (0.75 mmol) of PMHS, 110 μL (0.930 g/mL, 1.0 mmol) of phenylacetylene, 130 μL (1.171 g/mL, 0.5 mmol) of 2-methylallyl diphenyl phosphate, 0.0603 g (0.75 mmol) of LiO*t*Bu, and 1.0 mL (0.5 M) of THF. After 3 h, the reaction was worked up. The combined filtrate was concentrated and the regioselectivity (*b*/*l* > 95/5) was monitored by ^{1}H NMR analysis. The crude mixture was purified by flash column chromatography using hexane as the eluent to give 0.0425 g (0.27 mmol, 54% yield) of the title compound as a colorless oil.

^{1}H NMR: (400 MHz, $CDCl_3$) δ 7.45-7.43 (m, 2H), 7.34-7.30 (m, 2H), 7.28-7.24 (m, 1H), 5.44 (s, 1H), 5.12 (s, 1H), 4.81 (s, 1H), 4.77 (s, 1H), 3.22 (s, 2H), 1.72 (s, 3H); ^{13}C NMR: (100 MHz, $CDCl_3$) δ 145.6, 143.5, 140.9, 128.1, 127.3, 126.1, 114.3, 112.5, 44.0, 22.3; The NMR spectra were consistent with the spectra reported in the literature.[19]

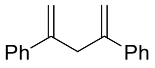

Penta-1,4-diene-2,4-diyldibenzene (**3c**)

Prepared according to the general procedure B using 0.1328 g (0.75 mmol)

of Co(OAc)$_2$, 0.3117 g (0.90 mmol) of **L3**, 5.4 mL (22.5 mmol) of PMHS, 3.3 mL (0.930 g/mL, 30.0 mmol) of phenylacetylene, 2.2 mL (1.370 g/mL, 15.0 mmol) of (3-bromoprop-1-en-2-yl)benzene, 1.8022 g (22.5 mmol) of LiO*t*Bu, and 30.0 mL (0.5 M) of THF. After 4 h, the reaction was worked up. The combined filtrate was concentrated and the regioselectivity (*b*/*l* > 95/5) was monitored by ^{1}H NMR analysis. The crude mixture was purified by flash column chromatography using PE as the eluent to give 2.36 g (10.5 mmol, 70% yield) of the title compound as a colorless oil. IR (neat, cm^{-1}): 3056, 2910, 2135, 1626, 1574, 1493; ^{1}H NMR: (400 MHz, CDCl$_3$) δ 7.46-7.42 (m, 4H), 7.34-7.24 (m, 6H), 5.47 (s, 2H), 5.14-5.12 (m, 2H), 3.67 (s, 2H); ^{13}C NMR: (100 MHz, CDCl$_3$) δ 145.3, 140.9, 128.2, 127.4, 126.0, 114.8, 40.8; HRMS (EI) calculated for $[C_{17}H_{16}]^+$ (M^+) requires m/z 220.1252, found m/z 220.1251.

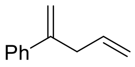

Penta-1,4-dien-2-ylbenzene (**3d**)

(a) Prepared according to the general procedure A using 0.2900 g (0.60 mmol) of (**L3**-H)•CoBr, 10.8 mL (45.0 mmol) of PMHS, 6.6 mL (0.930 g/mL, 60.0 mmol) of phenylacetylene, 2.6 mL (1.398 g/mL, 30.0 mmol) of allyl bromide, 3.67 g (45.0 mmol) of LiO*t*Bu, and 60.0 mL (0.5 M) of THF. After 45 min, the reaction was worked up. The combined filtrate was concentrated and the regioselectivity (*b*/*l* = 95/5) was monitored by ^{1}H NMR analysis. The crude mixture was distilled to give 2.06 g (14.4 mmol, 48% yield) of the title compound as a colorless oil. The boiling range of distillate was 58 ºC - 64 ºC (~2 mmHg).

(b) Prepared according to the general procedure using 0.0245 g (0.050 mmol) of (**L3**-H)•CoBr, 360 μL (1.5 mmol) of PMHS, 220 μL (0.930 g/mL, 2.0 mmol) of phenylacetylene, 92 μL (1.837 g/mL, 1.0 mmol) of 3-iodoprop-1-ene, 0.1243 g (1.5 mmol) of LiO*t*Bu, and 2.0 mL (0.5 M) of THF. After 1 h, the reaction was worked up. The combined filtrate was concentrated and the regioselectivity (*b*/*l* = 92/8) was monitored by ^{1}H NMR analysis. The crude mixture was distilled to give 0.0724 g (0.50 mmol, 50% yield) of the title compound as a colorless oil.

^{1}H NMR: (400 MHz, CDCl$_3$) δ 7.47-7.40 (m, 2H), 7.35-7.23 (m, 3H), 5.97-5.85 (m, 1H), 5.39 (s, 1H), 5.15-5.04 (m, 3H), 3.25 (d, J = 6.4 Hz, 2H); ^{13}C NMR: (100 MHz, CDCl$_3$) δ 146.3, 140.9, 136.2, 128.2, 127.4, 126.0, 116.4, 113.1, 39.5; The NMR spectra were consistent with the spectra reported in the literature.[19]

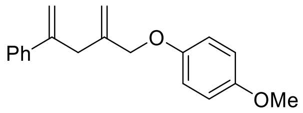

1-Methoxy-4-((2-methylene-4-phenylpent-4-en-1-yl)oxy)benzene (**3e**)

Prepared according to the general procedure B using 0.0045 g (0.025 mmol) of $Co(OAc)_2$, 0.0106 g (0.030 mmol) of **L3**, 180 μL (0.75 mmol) of PMHS, 110 μL (0.930 g/mL, 1.0 mmol) of phenylacetylene, 98 μL (1.315 g/mL, 0.50 mmol) of 1-((2-(bromomethyl)allyl)oxy)-4-methoxybenzene, 0.0597 g (0.75 mmol) of LiO*t*Bu, and 1.0 mL (0.5 M) of THF. After 4 h, the reaction was worked up. The combined filtrate was concentrated and the regioselectivity (*b*/*l* > 95/5) was monitored by ^{1}H NMR analysis. The crude mixture was purified by flash column chromatography using PE to PE/EA (50/1) as the eluent to give 0.1187 g (0.42 mmol, 84% yield) of the title compound as a colorless oil. IR (neat, cm^{-1}): 3078, 3027, 2972, 1719, 1651, 1598, 1494; ^{1}H NMR: (400 MHz, $CDCl_3$) δ 7.47-7.40 (m, 2H), 7.35-7.25 (m, 3H), 6.82-6.75 (m, 4H), 5.47 (d, J = 0.8 Hz, 1H), 5.20-5.16 (m, 2H), 5.05 (s, 1H), 4.41 (s, 2H), 3.76 (s, 3H), 3.37 (s, 2H); ^{13}C NMR: (100 MHz, $CDCl_3$) δ 153.8, 152.8, 144.8, 142.6, 140.5, 128.2, 127.5, 126.1, 115.7, 114.9, 114.6, 114.5, 71.0, 55.7, 39.3; HRMS (EI) calculated for $[C_{19}H_{20}O_2]^+$ (M^+) requires m/z 280.1463, found m/z 280.1461.

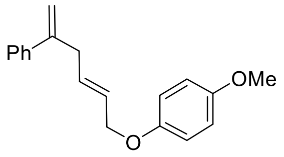

(*E*)-1-methoxy-4-((5-phenylhexa-2,5-dien-1-yl)oxy)benzene (**3f**)

Prepared according to the general procedure B using 0.0045 g (0.025 mmol) of $Co(OAc)_2$, 0.0113 g (0.030 mmol) of **L7**, 186 μL (0.75 mmol) of $Si(OSiHMe_2)_4$, 165 μL (0.930 g/mL, 1.5 mmol) of phenylacetylene, 0.1287 g (0.50 mmol) of (*E*)-1-(4-bromobut-2-en-1-yl)-4-methoxybenzene, 0.0607 g (0.75 mmol) of LiO*t*Bu, and 1.0 mL (0.5 M) of THF. After 2 h, the reaction was worked up. The combined filtrate was concentrated and the regioselectivity (*b*/*l* = 95/5) was monitored by ^{1}H NMR analysis. The crude mixture was purified by flash column chromatography using PE to PE/EA (50/1) as the eluent to give 0.0723 g (0.26 mmol, 52% yield) of the title compound as a colorless oil. IR (neat, cm^{-1}): 3027, 2962, 2907, 2134, 1595, 1507, 1461; ^{1}H NMR: (400 MHz, $CDCl_3$) δ 7.45-7.40 (m, 2H), 7.35-7.26 (m, 3H), 6.86-6.78 (m, 4H), 5.95-5.86 (m, 1H), 5.83-5.74 (m, 1H), 5.39 (s, 1H), 5.09 (d, J = 1.6 Hz, 1H), 4.43 (d, J = 6.4 Hz, 2H), 3.76 (s, 3H), 3.29 (d, J = 6.4 Hz, 2H); ^{13}C NMR: (100 MHz, $CDCl_3$) δ 153.8, 152.7, 146.1, 140.7, 132.1, 128.3, 127.5, 127.3, 126.0, 115.8, 114.5, 113.3, 69.2, 55.7, 38.0; HRMS (EI) calculated for $[C_{19}H_{20}O_2]^+$ (M^+) requires m/z 280.1463, found m/z 280.1465.

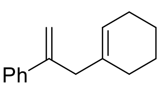

(3-(cyclohex-1-en-1-yl)prop-1-en-2-yl)benzene (**3g**)

Prepared according to the general procedure B using 0.0045 g (0.025 mmol) of $Co(OAc)_2$, 0.0106 g (0.030 mmol) of **L7**, 180 μL (0.75 mmol) of PMHS, 110 μL (0.930 g/mL, 1.0 mmol) of 1-ethynylcyclohexene, 68 μL (1.287 g/mL, 0.50 mmol) of 1-(bromomethyl)cyclohexene, 0.0605 g (0.75 mmol) of LiO*t*Bu, and 1.0 mL (0.5 M) of THF. After 1 h, the reaction was worked up. The combined filtrate was concentrated and the regioselectivity (*b*/*l*/S_N2' = 90/4/6) was monitored by ^1H NMR analysis. The crude mixture was purified by flash column chromatography using PE as the eluent to give 0.0621 g (0.32 mmol, 63% yield) of the title compound as a colorless oil. IR (neat, cm^{-1}): 3081, 3055, 2925, 2835, 1945, 1801, 1724, 1574, 1494; ^1H NMR: (400 MHz, $CDCl_3$) δ 7.44-7.40 (m, 2H), 7.33-7.27 (m, 2H), 7.25-7.20 (m, 1H), 5.51-5.45 (m, 1H), 5.40-5.38 (m, 1H), 5.08-5.05 (m, 1H), 3.11 (s, 2H), 2.02-1.88 (m, 4H), 1.63-1.55 (m, 2H), 1.53-1.47 (m, 2H); ^{13}C NMR: (100 MHz, $CDCl_3$) δ 146.0, 141.4, 135.4, 128.1, 127.2, 126.1, 123.5, 113.7, 44.0, 28.2, 25.3, 23.0, 22.4; HRMS (EI) calculated for $[C_{15}H_{18}]^+$ (M^+) requires m/z 198.1409, found m/z 198.1408.

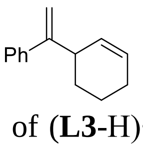

(1-(cyclohex-2-en-1-yl)vinyl)benzene (**3h**)

Prepared according to the general procedure A using 0.0125 g (0.025 mmol) of (**L3**-H)•CoBr, 180 μL (0.75 mmol) of PMHS, 110 μL (0.930 g/mL, 1.0 mmol) of phenylacetylene, 58 μL (1.400 g/mL, 0.50 mmol) of 1-bromo-2-cyclohexene, 0.0597 g (0.75 mmol) of LiO*t*Bu, and 1.0 mL (0.5 M) of THF. After 4 h, the reaction was worked up. The combined filtrate was concentrated and the regioselectivity (*b*/*l* > 95/5) was monitored by ^1H NMR analysis. The crude mixture was purified by flash column chromatography using PE as the eluent to give 0.0489 g (0.27 mmol, 53% yield) of the title compound as a colorless oil. ^1H NMR: (400 MHz, $CDCl_3$) δ 7.42-7.37 (m, 2H), 7.35-7.30 (m, 2H), 7.29-7.26 (m, 1H), 5.87-5.81 (m, 1H), 5.74-5.68 (m, 1H), 5.29 (d, J = 1.6 Hz, 1H), 5.07-5.04 (m, 1H), 3.41-3.33 (m, 1H), 2.06-1.99 (m, 2H), 1.85-1.76 (m, 1H), 1.72-1.63 (m, 1H), 1.55-1.40 (m, 2H); ^{13}C NMR: (100 MHz, $CDCl_3$) δ 152.4, 142.0, 129.7, 128.3, 128.2, 127.2, 126.6, 113.1, 39.8, 28.4, 25.2, 20.3. The NMR spectra were consistent with the spectra reported in the literature.[20]

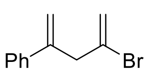

(4-Bromopenta-1,4-dien-2-yl)benzene (**3i**)

Prepared according to the general procedure B using 0.0043 g (0.025

mmol) of $Co(OAc)_2$, 0.0105 g (0.030 mmol) of **L3**, 180 μL (0.75 mmol) of PMHS, 110 μL (0.930 g/mL, 1.0 mmol) of phenylacetylene, 52 μL (1.934 g/mL, 0.50 mmol) of 2,3-dibromoprop-1-ene, 0.0605 g (0.75 mmol) of LiO*t*Bu, and 1.0 mL (0.5 M) of THF. After 4 h, the reaction was worked up. The combined filtrate was concentrated and the regioselectivity (*b*/*l* = 95/5) was monitored by ^{1}H NMR analysis. The crude mixture was purified by flash column chromatography using PE as the eluent to give 0.0702 g (0.32 mmol, 63% yield) of the title compound as a colorless oil. IR (neat, cm^{-1}): 3062, 2956, 1689, 1595, 1565, 1473; ^{1}H NMR: (400 MHz, $CDCl_3$) δ 7.45-7.40 (m, 2H), 7.36-7.25 (m, 3H), 5.63-5.60 (m, 1H), 5.56-5.54 (m, 1H), 5.50-5.47 (m, 1H), 5.25-5.22 (m, 1H), 3.63 (s, 2H); ^{13}C NMR: (100 MHz, $CDCl_3$) δ 143.6, 139.7, 131.2, 128.3, 127.7, 126.0, 118.7, 116.1, 47.3; HRMS (EI) calculated for $[C_{11}H_{11}Br]^+$ (M^+) requires m/z 222.0044, found m/z 222.0043.

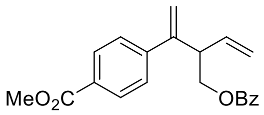

Methyl 4-(3-((benzoyloxy)methyl)penta-1,4-dien-2-yl)benzoate (**3j**)

Prepared according to the general procedure **B** using 0.0044 g (0.025 mmol) of $Co(OAc)_2$, 0.0107 g (0.030 mmol) of **L7**, 180 μL (0.75 mmol) of PMHS, 130 μL (0.916 g/mL, 1.0 mmol) of 4-(Methoxycarbonyl)phenylacetylene, 100 μL (1.278 g/mL, 0.50 mmol) of (*Z*)-4-bromobut-2-en-1-yl benzoate, 0.0607 g (0.75 mmol) of LiO*t*Bu, and 1.0 mL (0.5 M) of THF. After 1 h, the reaction was worked up. The combined filtrate was concentrated and the regioselectivity (S_N2'/S_N2 = 86/14; *b*/*l* > 95/5) was monitored by ^{1}H NMR analysis. The crude mixture was purified by flash column chromatography using PE/EA (5/1) as the eluent to give 0.0948 g (0.28 mmol, 56% yield) of the title compound as a colorless oil. IR (neat, cm^{-1}): 3077, 2953, 2903, 2168, 1718, 1607; ^{1}H NMR: (400 MHz, $CDCl_3$) δ 8.02-7.93 (m, 4H), 7.55-7.38 (m, 5H), 6.01-5.90 (m, 1H), 5.51 (s, 1H), 5.32 (s, 1H), 5.28-5.20 (m, 2H), 4.50-4.40 (m, 2H), 3.92 (s, 3H), 3.85-3.77 (m, 1H); ^{13}C NMR: (100 MHz, $CDCl_3$) δ 166.8, 166.3, 147.2, 146.1, 136.8, 132.9, 130.0, 129.7, 129.5, 129.2, 128.3, 126.6, 117.5, 116.2, 66.2, 52.0, 47.1; HRMS (ESI) calculated for $[C_{21}H_{20}NaO_2]^+$ (M+Na^+) requires m/z 359.1254, found m/z 359.1255.

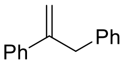

Prop-2-ene-1,2-diyldibenzene (**3k**)

Prepared according to the general procedure using 0.0876 g (0.025 mmol)

of $Co(OAc)_2$, 0.2011 g (0.030 mmol) of **L3**, 3.6 mL (15.0 mmol) of PMHS, 2.2 mL (0.930 g/mL, 20.0 mmol) of ethynylbenzene, 1.2 mL (10.0 mmol) of (bromomethyl)benzene, 1.25 g (15.0 mmol) of LiO*t*Bu, and 20.0 mL (0.5 M) of THF. After 4 h, the reaction was worked up. The combined filtrate was concentrated and the regioselectivity (*b*/*l* = 94/6) was monitored by ^1H NMR analysis. The crude mixture was purified by flash column chromatography using PE as the eluent to give 1.67 g (8.6 mmol, 86% yield) of the title compound as a colorless oil. ^1H NMR: (400 MHz, $CDCl_3$) δ 7.46-7.41 (m, 2H), 7.32-7.22 (m, 7H), 7.20-7.15 (m, 1 H), 5.49 (s, 1H), 5.04-5.00 (m, 1H), 3.84 (s, 2H); ^{13}C NMR: (100 MHz, $CDCl_3$) δ 146.9, 140.8, 139.5, 128.9, 128.3, 128.2, 127.4, 126.09, 126.06, 114.6, 41.6; The NMR spectra were consistent with the spectra reported in the literature.[21]

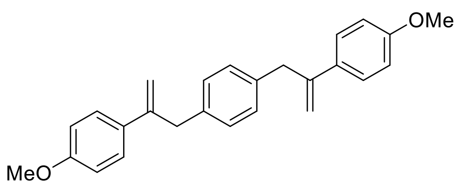

1,4-Bis(2-(4-methoxyphenyl)allyl)benzene (**3l**)
Prepared according to the general procedure B using 0.0044 g (0.025 mmol) of $Co(OAc)_2$, 0.0105 g (0.030 mmol) of **L3**, 360 μL (1.5 mmol) of PMHS, 260 μL (0.926 g/mL, 1.0 mmol) of 1-ethynyl-4-methoxybenzene, 0.1327 g (0.50 mmol) of 1,4-bis(bromomethyl)benzene, 0.1204 g (1.50 mmol) of LiO*t*Bu, and 1.0 mL (0.5 M) of THF. After 4 h, the reaction was worked up. The combined filtrate was concentrated and the regioselectivity (*b*/*l* > 95/5) was monitored by ^1H NMR analysis. The crude mixture was purified by flash column chromatography using PE to PE/EA (100/1) as the eluent to give 0.0926 g (0.25 mmol, 50% yield) of the title compound as a white solid. IR (neat, cm^{-1}): 3014, 2924, 2846, 1607, 1512, 1443; ^1H NMR: (400 MHz, $CDCl_3$) δ 7.39-7.33 (m, 4H), 7.13-7.10 (m, 4H), 6.83-6.78 (m, 4H), 5.40 (s, 2H), 4.91 (s, 2H), 3.78 (s, 6H), 3.75 (s, 4H); ^{13}C NMR: (100 MHz, $CDCl_3$) δ 159.0, 146.1, 137.3, 133.2, 128.8, 127.4, 127.2, 114.1, 113.5, 112.8, 55.2, 41.2; HRMS (EI) calculated for $[C_{26}H_{26}O]^+$ (M^+) requires m/z 370.1933, found m/z 370.1934.

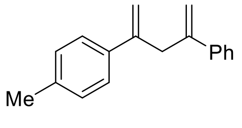

1-Methyl-4-(4-phenylpenta-1,4-dien-2-yl)benzene (**3m**)
Prepared according to the general procedure A using 0.0122 g (0.025 mmol) of (**L3**-H)•CoBr, 180 μL (0.75 mmol) of PMHS, 127 μL (0.916 g/mL, 1.0 mmol) of 4-ethynyltoluene, 72 μL (1.370 g/mL, 0.50 mmol) of (3-bromoprop-1-en-2-yl)benzene, 0.0610 g (0.75 mmol) of LiO*t*Bu, and 1.0 mL (0.5 M) of THF. After 1 h, the reaction was worked up. The combined filtrate was concentrated

and the regioselectivity (*b*/*l* > 95/5) was monitored by ^{1}H NMR analysis. The crude mixture was purified by flash column chromatography using PE as the eluent to give 0.0867 g (0.37 mmol, 74% yield) of the title compound as a colorless oil. IR (neat, cm^{-1}): 3084, 3054, 3026, 1901, 1800, 1728, 1686, 1572; ^{1}H NMR: (400 MHz, $CDCl_3$) δ 7.46-7.42 (m, 2H), 7.36-7.25 (m, 5H), 7.14-7.10 (m, 2H), 5.48-5.44 (m, 2H), 5.14-5.07 (m, 2H), 3.65 (s, 2H), 2.34 (s, 3H); ^{13}C NMR: (100 MHz, $CDCl_3$) δ 145.4, 145.1, 141.0, 138.0, 137.1, 128.9, 128.2, 127.4, 125.9, 125.8, 114.7, 114.0, 40.8, 21.1; HRMS (EI) calculated for $[C_{18}H_{18}]^+$ (M^+) requires m/z 234.1409, found m/z 234.1407.

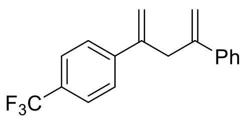

1-(4-Phenylpenta-1,4-dien-2-yl)-4-(trifluoromethyl)benzene (**3n**)
Prepared according to the general procedure A using 0.0121 g (0.025 mmol) of (**L3**-H)•CoBr, 180 μL (0.75 mmol) of PMHS, 163 μL (1.043 g/mL, 1.0 mmol) of 4-ethynyl-*α*,*α*,*α*-trifluorotoluene, 72 μL (1.370 g/mL, 0.50 mmol) of (3-bromoprop-1-en-2-yl)benzene, 0.0602 g (0.75 mmol) of LiO*t*Bu, and 1.0 mL (0.5 M) of THF. After 1 h, the reaction was worked up. The combined filtrate was concentrated and the regioselectivity (*b*/*l* = 92/8) was monitored by ^{1}H NMR analysis. The crude mixture was purified by flash column chromatography using PE as the eluent to give 0.0822 g (0.29 mmol, 57% yield) of the title compound as a colorless oil. IR (neat, cm^{-1}): 3056, 2957, 2899, 1877, 1730, 1690, 1623, 1494; ^{1}H NMR: (400 MHz, $CDCl_3$) δ 7.58-7.49 (m, 4H), 7.43-7.39 (m, 2H), 7.35-7.26 (m, 3H), 5.53 (s, 1H), 5.47 (s, 1H), 5.24 (s, 1H), 5.11 (d, J = 0.8 Hz, 1H), 3.68 (s, 2H); ^{13}C NMR: (100 MHz, $CDCl_3$) δ 145.0, 144.4, 144.3, 140.7, 128.3, 127.6, 126.3, 126.0, 125.2 (q, J =3.6 Hz), 116.9, 115.0, 40.8; ^{19}F NMR: (376 MHz, $CDCl_3$) δ -62.44; HRMS (EI) calculated for $[C_{18}H_{15}F]^+$ (M^+) requires m/z 288.1126, found m/z 288.1128.

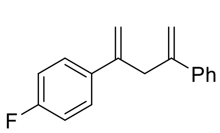

1-Fluoro-4-(4-phenylpenta-1,4-dien-2-yl)benzene (**3o**)
Prepared according to the general procedure A using 0.0123 g (0.025 mmol) of (**L3**-H)•CoBr, 180 μL (0.75 mmol) of PMHS, 115 μL (1.048 g/mL, 1.0 mmol) of 4-fluorophenylacetylene, 72 μL (1.370 g/mL, 0.50 mmol) of (3-bromoprop-1-en-2-yl)benzene, 0.0600 g (0.75 mmol) of LiO*t*Bu, and 1.0 mL (0.5 M) of THF. After 1 h, the reaction was worked up. The combined filtrate was concentrated and the regioselectivity (*b*/*l* > 95/5) was monitored by ^{1}H NMR analysis. The crude mixture was purified by flash column chromatography using PE as the eluent to give 0.0894 g (0.38 mmol, 75% yield) of the title compound as a colorless oil. IR

(neat, cm^{-1}): 3083, 3054, 2956, 1728, 1687, 1603, 1530, 1494; ^{1}H NMR: (400 MHz, $CDCl_3$) δ 7.44-7.35 (m, 4H), 7.34-7.25 (m, 3H), 7.02-6.95 (m, 2H), 5.46 (s, 1H), 5.40 (s, 1H), 5.13-5.10 (m, 2H), 3.64 (s, 2H); ^{13}C NMR: (100 MHz, $CDCl_3$) δ 161.2 (J = 245.0 Hz), 144.8 (J = 86.8 Hz), 140.9, 136.9 (J = 3.0 Hz), 128.3, 127.6 (J = 7.3 Hz), 127.5, 126.0, 115.1, 114.9, 114.84, 114.77, 41.1; ^{19}F NMR: (376 MHz, $CDCl_3$) δ -115.24; HRMS (EI) calculated for $[C_{17}H_{15}F]^+$ (M^+) requires m/z 238.1158, found m/z 238.1157.

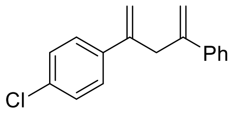

1-Chloro-4-(4-phenylpenta-1,4-dien-2-yl)benzene (**3p**)

Prepared according to the general procedure A using 0.0124 g (0.025 mmol) of (**L3**-H)•CoBr, 180 μL (0.75 mmol) of PMHS, 110 μL (1.240 g/mL, 1.0 mmol) of 4-chlorophenylacetylen, 72 μL (1.370 g/mL, 0.5 mmol) of (3-bromoprop-1-en-2-yl)benzene, 0.0670 g (0.75 mmol) of LiO*t*Bu, and 1.0 mL (0.5 M) of THF. After 1 h, the reaction was worked up. The combined filtrate was concentrated and the regioselectivity (*b*/*l* = 95/5) was monitored by ^{1}H NMR analysis. The crude mixture was purified by flash column chromatography using PE as the eluent to give 0.0867 g (0.30 mmol, 60% yield) of the title compound as a colorless oil. IR (neat, cm^{-1}): 3058, 2963, 1684, 1594, 1491, 1402; ^{1}H NMR: (400 MHz, $CDCl_3$) δ 7.43-7.39 (m, 2H), 7.37-7.31 (m, 3H), 7.31-7.25 (m, 4H), 5.47-5.44 (m, 2H), 5.17-5.14 (m, 1H), 5.12-5.08 (m, 1H), 3.64 (s, 2H); ^{13}C NMR: (100 MHz, $CDCl_3$) δ 145.1, 144.2, 140.8, 139.2, 133.2, 128.35, 128.27, 127.5, 127.3, 125.9, 115.4, 114.9, 40.8; HRMS (EI) calculated for $[C_{17}H_{15}Cl]^+$ (M^+) requires m/z 254.0862, found m/z 254.0861.

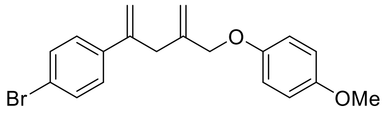

1-Bromo-4-(4-((4-methoxyphenoxy)methyl)penta-1,4-dien-2-yl)benzene (**3q**)

Prepared according to the general procedure B using 0.0044 g (0.025 mmol) of $Co(OAc)_2$, 0.0105 g (0.030 mmol) of **L3**, 180 μL (0.75 mmol) of PMHS, 0.1819 g (1.0 mmol) of 1-bromo-4-ethynylbenzene, 98 μL (1.315 g/mL, 0.50 mmol) of 1-((2-(bromomethyl)allyl)oxy)-4-methoxybenzene, 0.0605 g (0.75 mmol) of LiO*t*Bu, and 1.0 mL (0.5 M) of THF. After 4 h, the reaction was worked up. The combined filtrate was concentrated and the regioselectivity (*b*/*l* = 94/6) was monitored by ^{1}H NMR analysis. The crude mixture was purified by flash column chromatography using PE to PE/EA (80/1) as the eluent to give 0.1108 g (0.31 mmol, 62% yield) of the title compound as a colorless oil. IR (neat, cm^{-1}): 3084, 2952, 2857, 1509, 1464, 1376; ^{1}H NMR: (400 MHz,

CDCl3) δ 7.41 (d, J = 8.8 Hz, 2H), 7.28 (d, J = 8.8 Hz, 2H), 6.82-6.77 (m, 4H), 5.46 (s, 1H), 5.22-5.17 (m, 2H), 5.01 (s, 1H), 4.38 (s, 2H), 3.75 (s, 3H), 3.33 (s, 2H); ^{13}C NMR: (100 MHz, CDCl3) δ 153.8, 152.7, 143.8, 142.3, 139.3, 131.3, 127.8, 121.4, 115.6, 115.5, 114.9, 114.5, 70.9, 55.6, 39.1; HRMS (EI) calculated for $[C_{19}H_{19}O_2Br]^+$ (M^+) requires m/z 358.0568, found m/z 358.0569.

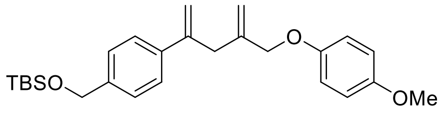

Tert-butyl((4-(4-((4-methoxyphenoxy)methyl)penta-1,4-dien-2-yl)benzyl)oxy)dimethylsilane (**3r**)

Prepared according to the general procedure B using 0.0045 g (0.025 mmol) of $Co(OAc)_2$, 0.0107 g (0.030 mmol) of **L3**, 180 μL (0.75 mmol) of PMHS, 280 μL (0.869 g/mL, 1.0 mmol) of *tert*-butyl((4-ethynylbenzyl)oxy)dimethylsilane, 98 μL (1.315 g/mL, 0.50 mmol) of 1-((2-(bromomethyl)allyl)oxy)-4-methoxybenzene, 0.0607 g (0.75 mmol) of LiO*t*Bu, and 1.0 mL (0.5 M) of THF. After 4 h, the reaction was worked up. The combined filtrate was concentrated and the regioselectivity (*b*/*l* = 94/6) was monitored by ^{1}H NMR analysis. The crude mixture was purified by flash column chromatography using PE to PE/EA (100/1) as the eluent to give 0.1127 g (0.29 mmol, 58% yield) of the title compound as a colorless oil. IR (neat, cm^{-1}): 2958, 2923, 2860, 2299, 1813, 1697, 1608, 1531; ^{1}H NMR: (400 MHz, CDCl3) δ 7.42-7.38 (m, 2H), 7.28-7.24 (m, 2H), 6.84-6.78 (m, 4H), 5.46 (d, J = 1.2 Hz, 1H), 5.19 (s, 1H), 5.14 (d, J = 1.2 Hz, 1H), 5.04 (s, 1H), 4.73 (s, 2H), 4.40 (s, 2H), 3.76 (s, 3H), 3.36 (s, 2H), 0.94 (s, 9H), 0.10 (s, 6H); ^{13}C NMR: (100 MHz, CDCl3) δ 153.8, 152.8, 144.6, 142.6, 140.7, 139.1, 126.0, 125.9, 115.7, 114.6, 114.51, 114.49, 71.0, 64.7, 55.7, 39.3, 25.9, 18.4, -5.3; HRMS (ESI) calculated for $[C_{26}H_{36}NaO_3Si]^+$ ($M+Na^+$) requires m/z 447.2326, found m/z 447.2329.

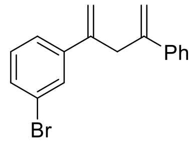

1-Bromo-3-(4-phenylpenta-1,4-dien-2-yl)benzene (**3s**)

Prepared according to the general procedure A using 0.0121 g (0.025 mmol) of (**L3**-H)•CoBr, 180 μL (0.75 mmol) of PMHS, 0.1251 g (1.0 mmol) of 3-bromophenylacetylene, 72 μL (1.370 g/mL, 0.50 mmol) of (3-bromoprop-1-en-2-yl)benzene, 0.0605 g (0.75 mmol) of LiO*t*Bu, and 1.0 mL (0.5 M) of THF. After 1 h, the reaction was worked up. The combined filtrate was concentrated and the regioselectivity (*b*/*l* = 94/6) was monitored by ^{1}H NMR analysis. The crude mixture was purified by flash column chromatography using PE as the eluent to give 0.0916 g

(0.32 mmol, 61% yield) of the title compound as a colorless oil. IR (neat, cm^{-1}): 3062, 3024, 2921, 1956, 1688, 1596; ^{1}H NMR: (400 MHz, $CDCl_3$) δ 7.58-7.55 (m, 1H), 7.44-7.36 (m, 3H), 7.35-7.26 (m, 4H), 7.20-7.14 (m, 1H), 5.48-5.45 (m, 2H), 5.18-5.15 (m, 1H), 5.13-5.10 (m, 1H), 3.63 (s, 2H); ^{13}C NMR: (100 MHz, $CDCl_3$) δ 144.9, 144.1, 143.1, 140.7, 130.3, 129.7, 129.1, 128.3, 127.5, 125.9, 124.6, 122.5, 116.0, 115.0, 40.7; HRMS (EI) calculated for $[C_{17}H_{15}Br]^+$ (M^+) requires m/z 298.0357, found m/z 298.0357.

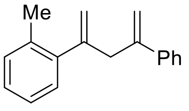

1-Methyl-2-(4-phenylpenta-1,4-dien-2-yl)benzene (**3t**)

Prepared according to the general procedure A using 0.0121 g of (**L3**-H)•CoBr, 180 μL (0.75 mmol) of PMHS, 126 μL (0.922 g/mL, 1.0 mmol) of 1-ethynyl-2-methylbenzene, 72 μL (1.370 g/mL, 0.50 mmol) of (3-bromoprop-1-en-2-yl)benzene, 0.0599 g (0.75 mmol) of LiO*t*Bu, and 1.0 mL (0.5 M) of THF. After 1 h, the reaction was worked up. The combined filtrate was concentrated and the regioselectivity (*b*/*l* = 93/7) was monitored by ^{1}H NMR analysis. The crude mixture was purified by flash column chromatography using PE as the eluent to give 0.0647 g (0.24 mmol, 55% yield) of the title compound as a colorless oil. IR (neat, cm^{-1}): 3061, 3023, 2955, 2918, 1731, 1628, 1491, 1448; ^{1}H NMR: (400 MHz, $CDCl_3$) δ 7.44-7.40 (m, 2H), 7.34-7.26 (m, 3H), 7.16-7.08 (m, 3H), 7.05-7.00 (m, 1H), 5.43-5.40 (m, 1H), 5.23-5.20 (m, 1H), 5.10-5.06 (m, 1H), 4.95-4.92 (m, 1H), 3.53 (s, 2H), 2.24 (s, 3H); ^{13}C NMR: (100 MHz, $CDCl_3$) δ 147.2, 145.1, 142.7, 140.8, 134.8, 130.1, 128.3, 128.2, 127.3, 126.8, 126.2, 125.3, 116.1, 115.3, 43.5, 19.8; HRMS (EI) calculated for $[C_{18}H_{18}]^+$ (M^+) requires m/z 234.1409, found m/z 234.1408.

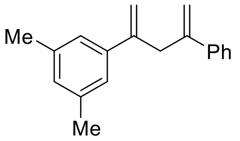

1,3-Dimethyl-5-(4-methylpenta-1,4-dien-2-yl)benzene (**3u**)

Prepared according to the general procedure A using 0.0123 g of (**L3**-H)•CoBr, 180 μL (0.75 mmol) of PMHS, 140 μL (0.930 g/mL, 1.0 mmol) of 1-ethynyl-3,5-dimethylbenzene, 72 μL (1.370 g/mL, 0.50 mmol) of (3-bromoprop-1-en-2-yl)benzene, 0.0601 g (0.75 mmol) of LiO*t*Bu, and 1.0 mL (0.5 M) of THF. After 1 h, the reaction was worked up. The combined filtrate was concentrated and the regioselectivity (*b*/*l* > 95/5) was monitored by ^{1}H NMR analysis. The crude mixture was purified by flash column chromatography using PE as the eluent to give 0.0944 g (0.38 mmol, 76% yield) of the title compound as a colorless oil. IR (neat, cm^{-1}): 3083, 3029, 2916, 1728, 1625, 1599; ^{1}H NMR: (400 MHz, $CDCl_3$) δ 7.45 (d, J = 7.6

Hz, 2H), 7.35-7.23 (m, 3H), 7.06 (s, 2H), 6.91 (s, 1H), 5.49 (s, 1H), 5.43 (s, 1H), 5.14 (s, 1H), 5.08 (s, 1H), 3.64 (s, 2H), 2.30 (s, 6H); ^{13}C NMR: (100 MHz, $CDCl_3$) δ 145.6, 145.4, 141.1, 141.0, 137.6, 129.1, 128.2, 127.4, 126.0, 123.9, 114.7, 114.4, 40.9, 21.4; HRMS (EI) calculated for $[C_{19}H_{20}]^+$ (M^+) requires m/z 248.1565, found m/z 248.1563.

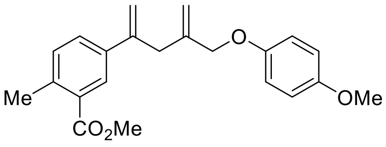

1,3-Dimethyl-5-(4-methylpenta-1,4-dien-2-yl)benzene (**3v**)

Prepared according to the general procedure B using 0.0045 g (0.025 mmol) of $Co(OAc)_2$, 0.0106 g (0.030 mmol) of **L3**, 180 μL (0.75 mmol) of PMHS, 0.1760 g (1.0 mmol) of methyl 5-ethynyl-2-methylbenzoate, 98 μL (1.315 g/mL, 0.50 mmol) of 1-((2-(bromomethyl)allyl)oxy)-4-methoxybenzene, 0.0598 g (0.75 mmol) of LiO*t*Bu, and 1.0 mL (0.5 M) of THF. After 4 h, the reaction was worked up. The combined filtrate was concentrated and the regioselectivity (*b*/*l* > 95/5) was monitored by ^{1}H NMR analysis. The crude mixture was purified by flash column chromatography using PE to PE/EA (20/1) as the eluent to give 0.0961 g (0.28 mmol, 55% yield) of the title compound as a colorless oil. IR (neat, cm^{-1}): 2996, 2928, 1724, 1508, 1440; ^{1}H NMR: (400 MHz, $CDCl_3$) δ 8.00 (d, J = 2.0 Hz, 1H), 7.45 (dd, J = 8.0, 2.0 Hz, 1H), 7.18 (d, J = 8.0 Hz, 1H), 6.84-6.76 (m, 4H), 5.49 (d, J = 1.2 Hz, 1H), 5.21-5.17 (m, 2H), 5.04 (d, J = 0.8 Hz, 1H), 4.40 (s, 2H), 3.89 (s, 3H), 3.76 (s, 3H), 3.37 (s, 2H), 2.57 (s, 3H); ^{13}C NMR: (100 MHz, $CDCl_3$) δ 167.9, 153.8, 152.7, 143.7, 142.3, 139.3, 137.9, 131.6, 129.5, 129.3, 128.2, 115.6, 115.0, 114.8, 114.4, 70.9, 55.6, 51.8, 39.0, 21.4; HRMS (EI) calculated for $[C_{22}H_{24}O_4]^+$ (M^+) requires m/z 352.1675, found m/z 352.1677.

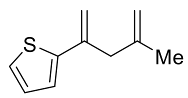

2-(4-Methylpenta-1,4-dien-2-yl)thiophene (**3w**)

Prepared according to the general procedure B using 0.0044 g (0.025 mmol) of $Co(OAc)_2$, 0.0104 g (0.030 mmol) of **L3**, 180 μL (0.75 mmol) of PMHS, 100 μL (1.080 g/mL, 1.0 mmol) of 2-ethynylthiophene, 52 μL (1.339 g/mL, 0.50 mmol) of 3-bromo-2-methylpropene, 0.0607 g (0.75 mmol) of LiO*t*Bu, and 1.0 mL (0.5 M) of THF. After 4 h, the reaction was worked up. The combined filtrate was concentrated and the regioselectivity (*b*/*l* = 91/9) was monitored by ^{1}H NMR analysis. The crude mixture was purified by flash column chromatography using pentane as the eluent to give 0.0393 g (0.24 mmol, 48% yield) of the title compound as a colorless oil. IR (neat, cm^{-1}): 3102, 2957, 2922, 1764, 1716, 1655, 1413; ^{1}H NMR: (400 MHz, $CDCl_3$) δ 7.14

(d, J = 5.2 Hz, 1H), 7.06 (d, J = 3.2 Hz, 1H), 6.95 (dd, J = 4.4, 4.4 Hz, 1H), 5.50 (s, 1H), 4.99 (s, 1H), 4.88-4.83 (m, 2H), 3.19 (s, 2H), 1.75 (s, 3H); ^{13}C NMR: (100 MHz, $CDCl_3$) δ 145.0, 143.1, 139.1, 127.3, 124.1, 123.8, 112.8, 44.2, 22.1; HRMS (EI) calculated for $[C_{10}H_{12}S]^+$ (M^+) requires m/z 164.0660, found m/z 164.0661.

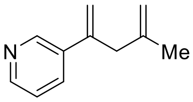

3-(4-Methylpenta-1,4-dien-2-yl)pyridine (**3x**)

Prepared according to the general procedure B using 0.0044 g (0.025 mmol) of $Co(OAc)_2$, 0.0102 g (0.030 mmol) of **L3**, 180 μL (0.75 mmol) of PMHS, 100 μL (1.020 g/mL, 1.0 mmol) of 3-ethynylpyridine, 52 μL (1.339 g/mL, 0.50 mmol) of 3-bromo-2-methylpropene, 0.0600 g (0.75 mmol) of LiO*t*Bu, and 1.0 mL (0.5 M) of THF. After 4 h, the reaction was worked up. The combined filtrate was concentrated and the regioselectivity (*b*/*l* = 83/17) was monitored by ^{1}H NMR analysis. The crude mixture was purified by flash column chromatography using PE to PE/EA (5/1) as the eluent to give 0.0393 g (0.25 mmol, 50% yield) of the title compound as a colorless oil. IR (neat, cm^{-1}): 3080, 2963, 1689, 1587, 1416; ^{1}H NMR: (400 MHz, $CDCl_3$) δ 8.70 (s, 1H), 8.50 (d, 4.4 Hz, 1H), 7.70 (d, J = 8.0 Hz, 1H), 7.26-7.20 (m, 1H), 5.48 (s, 1H), 5.22 (s, 1H), 4.82 (s, 1H), 4.76 (s, 1H), 3.21 (s, 2H), 1.71 (s, 3H); ^{13}C NMR: (100 MHz, $CDCl_3$) δ 148.5, 147.6, 142.7, 142.6, 136.3, 133.3, 123.1, 116.0, 113.1, 43.8, 22.2; HRMS (EI) calculated for $[C_{11}H_{13}N]^+$ (M^+) requires m/z 159.1048, found m/z 159.1050.

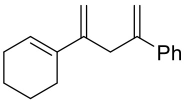

(4-(Cyclohex-1-en-1-yl)penta-1,4-dien-2-yl)benzene (**3y**)

Prepared according to the general procedure A using 0.0122 g (0.025 mmol) of (**L3**-H)•CoBr, 180 μL (0.75 mmol) of PMHS, 178 μL (0.903 g/mL, 1.0 mmol) of 1-ethynylcyclohexene, 72 μL (1.370 g/mL, 0.50 mmol) of (3-bromoprop-1-en-2-yl)benzene, 0.0602 g (0.75 mmol) of LiO*t*Bu, and 1.0 mL (0.5 M) of THF. After 1 h, the reaction was worked up. The combined filtrate was concentrated and the regioselectivity (*b*/*l* = 94/6) was monitored by ^{1}H NMR analysis. The crude mixture was purified by flash column chromatography using PE as the eluent to give 0.0636 g (0.29 mmol, 57% yield) of the title compound as a colorless oil. IR (neat, cm^{-1}): 3061, 2926, 2125, 1722, 1602, 1493; ^{1}H NMR: (400 MHz, $CDCl_3$) δ 7.46-7.40 (m, 2H), 7.34-7.28 (m, 2H), 7.26-7.21 (m, 1H), 5.91 (t, J = 4.0 Hz, 1H), 5.47 (s, 1H), 5.12 (s, 1H), 5.08 (d, J = 0.8 Hz, 1H), 4.87 (s, 1H), 3.42 (s, 2H), 2.24-2.17 (m, 2H), 2.16-2.10 (m, 2H), 1.71-1.64 (m, 2H), 1.61-1.55 (m, 2H); ^{13}C NMR: (100 MHz, $CDCl_3$) δ 146.0, 145.7, 141.3, 135.6, 128.2, 127.3, 125.9, 124.8, 114.1, 111.3, 39.2, 26.0, 25.9, 22.9,

22.2; HRMS (EI) calculated for $[C_{17}H_{20}]^+$ (M^+) requires m/z 224.1565, found m/z 224.1565.

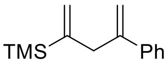

Trimethyl(4-phenylpenta-1,4-dien-2-yl)silane (**3z**)

Prepared according to the general procedure B using 0.0045 g (0.025 mmol) of $Co(OAc)_2$, 0.0106 g (0.030 mmol) of **L3**, 180 μL (0.75 mmol) of PMHS, 212 μL (0.695 g/mL, 1.5 mmol) of ethynyltrimethylsilane, 72 μL (1.370 g/mL, 0.50 mmol) of (3-bromoprop-1-en-2-yl)benzene, 0.0613 g (0.75 mmol) of LiO*t*Bu, and 1.0 mL (0.5 M) of THF. After 4 h, the reaction was worked up. The combined filtrate was concentrated and the regioselectivity (*b*/*l* = 91/9) was monitored by ^{1}H NMR analysis. The crude mixture was purified by flash column chromatography using PE as the eluent to give 0.0692 g (0.32 mmol, 64% yield) of the title compound as a colorless oil. IR (neat, cm^{-1}): 3082, 3055, 2956, 1690, 1624, 1575, 1444, 1249; ^{1}H NMR: (400 MHz, $CDCl_3$) δ 7.38-7.34 (m, 2H), 7.29-7.24 (m, 2H), 7.23-7.19 (m, 1H), 5.56-5.53 (m, 1H), 5.44 (d, J = 1.6 Hz, 1H), 5.39-5.37 (m, 1H), 5.03 (d, J = 1.2 Hz, 1H), 3.28 (d, J = 1.2 Hz, 2H), 0.06 (s, 9H); ^{13}C NMR: (100 MHz, $CDCl_3$) δ 149.3, 145.9, 141.1, 128.1, 127.2, 126.2, 126.1, 114.6, 41.4, -1.5; HRMS (EI) calculated for $[C_{14}H_{20}Si]^+$ (M^+) requires m/z 216.1334, found m/z 216.1332.

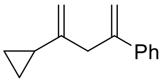

(4-Cyclopropylpenta-1,4-dien-2-yl)benzene (**3aa**)

Prepared according to the general procedure B using 0.0045 g (0.025 mmol) of $Co(OAc)_2$, 0.0105 g (0.030 mmol) of **L3**, 180 μL (0.75 mmol) of PMHS, 127 μL (0.780 g/mL, 1.5 mmol) of ethynylcyclopropane, 72 μL (1.370 g/mL, 0.50 mmol) of (3-bromoprop-1-en-2-yl)benzene, 0.0610 g (0.75 mmol) of LiO*t*Bu, and 1.0 mL (0.5 M) of THF. After 4 h, the reaction was worked up. The combined filtrate was concentrated and the regioselectivity (*b*/*l* = 94/6) was monitored by ^{1}H NMR analysis. The crude mixture was purified by flash column chromatography using pentane as the eluent to give 0.0617 g (0.34 mmol, 67% yield) of the title compound as a colorless oil. IR (neat, cm^{-1}): 3082, 2928, 2870, 1759, 1685, 1600, 1449, 1383; ^{1}H NMR: (400 MHz, $CDCl_3$) δ 7.45-7.41 (m, 2H), 7.33-7.28 (m, 2H), 7.27-7.22 (m, 1H), 5.46 (d, J = 1.2 Hz, 1H), 5.16 (d, J = 0.8 Hz, 1H), 4.72-4.68 (m, 2H), 3.23 (s, 2H), 1.40-1.31 (m, 1H), 0.64-0.59 (m, 2H), 0.47-0.42 (m, 2H); ^{13}C NMR: (100 MHz, $CDCl_3$) δ 148.5, 145.7, 141.1, 128.1, 127.3, 126.0, 114.5, 108.7, 42.0, 16.1, 6.2; HRMS (EI) calculated for $[C_{14}H_{16}]^+$ (M^+) requires m/z 184.1252, found m/z 182.1254.

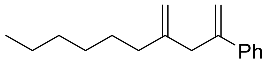

(4-Methylenedec-1-en-2-yl)benzene (**3ab**)

Prepared according to the general procedure B using 0.0044 g (0.025 mmol) of $Co(OAc)_2$, 0.0108 g (0.030 mmol) of **L3**, 180 μL (0.75 mmol) of PMHS, 221 μL (0.747 g/mL, 1.50 mmol) of oct-1-yne, 72 μL (1.370 g/mL, 0.50 mmol) of (3-bromoprop-1-en-2-yl)benzene, 0.0611 g (0.75 mmol) of LiO*t*Bu, and 1.0 mL (0.5 M) of THF. After 4 h, the reaction was worked up. The combined filtrate was concentrated and the regioselectivity (*b*/*l* = 92/8) was monitored by ^{1}H NMR analysis. The crude mixture was purified by flash column chromatography using PE as the eluent to give 0.0633 g (0.22 mmol, 55% yield) of the title compound as a colorless oil. IR (neat, cm^{-1}): 2954, 1686, 1602, 1451, 1377; ^{1}H NMR: (400 MHz, $CDCl_3$) δ 7.44-7.40 (m, 2H), 7.33-7.28 (m, 2H), 7.27-7.24 (m, 1H), 5.45-5.42 (m, 1H), 5.12-5.09 (m, 1H), 4.80 (s, 1H), 4.78 (s, 1H), 3.21 (s, 2H), 2.01 (t, J = 8.0 Hz, 2H), 1.48-1.38 (m, 2H), 1.32-1.22 (m, 6H), 0.91-0.84 (m, 3H); ^{13}C NMR: (100 MHz, $CDCl_3$) δ 147.5, 145.7, 141.0, 128.1, 127.3, 126.1, 126.0, 114.4, 111.4, 42.3, 35.8, 31.8, 29.0, 27.6, 22.6, 14.1; HRMS (EI) calculated for $[C_{17}H_{24}]^+$ (M^+) requires m/z 228.1878, found m/z 228.1879.

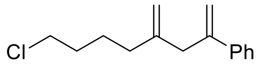

(8-Chloro-4-methyleneoct-1-en-2-yl)benzene (**3ac**)

Prepared according to the general procedure A using 0.0121 g (0.025 mmol) of (**L3**-H)•CoBr, 180 μL (0.75 mmol) of PMHS, 121 μL (0.962 g/mL, 1.0 mmol) of 6-chlorohex-1-yne, 72 μL (1.370 g/mL, 0.50 mmol) of (3-bromoprop-1-en-2-yl)benzene, 0.0589 g (0.75 mmol) of LiO*t*Bu, and 1.0 mL (0.5 M) of THF. After 1 h, the reaction was worked up. The combined filtrate was concentrated and the regioselectivity (*b*/*l* = 90/10) was monitored by ^{1}H NMR analysis. The crude mixture was purified by flash column chromatography using PE to PE/EA (150/1) as the eluent to give 0.0681 g (0.29 mmol, 58% yield) of the title compound as a colorless oil. IR (neat, cm^{-1}): 3061, 2954, 2930, 1720, 1684, 1599, 1449; ^{1}H NMR: (400 MHz, $CDCl_3$) δ 7.44-7.40 (m, 2H), 7.34-7.28 (m, 2H), 7.27-7.24 (m, 1H), 5.44 (d, J = 1.2 Hz, 1H), 5.11 (d, J = 1.2 Hz, 1H), 4.83-4.80 (m, 2H), 3.53 (t, J = 6.4 Hz, 2H), 3.23 (s, 2H), 2.04 (t, J = 7.8 Hz, 2H), 1.78-1.72 (m, 2H), 1.62-1.56 (m, 2H); ^{13}C NMR: (100 MHz, $CDCl_3$) δ 146.4, 145.5, 140.8, 128.2, 127.4, 126.1, 114.6, 112.1, 45.0, 42.2, 34.8, 32.1, 24.7; HRMS (EI) calculated for $[C_{15}H_{19}Cl]^+$ (M^+) requires m/z 234.1175, found m/z 234.1173.

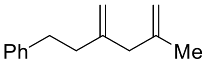 (5-Methyl-3-methylenehex-5-en-1-yl)benzene (**3ad**)

Prepared according to the general procedure B using 0.0044 g (0.025 mmol) of $Co(OAc)_2$, 0.0105 g (0.030 mmol) of **L3**, 180 μL (1.50 mmol) of PMHS, 141 μL (0.926 g/mL, 1.0 mmol) of but-3-yn-1-ylbenzene, 52 μL (1.339 g/mL, 0.50 mmol) of 3-bromo-2-methylpropene, 0.0607 g (0.75 mmol) of LiO*t*Bu, and 1.0 mL (0.5 M) of THF. After 4 h, the reaction was worked up. The combined filtrate was concentrated and the regioselectivity (*b*/*l* > 95/5) was monitored by ^{1}H NMR analysis. The crude mixture was purified by flash column chromatography using PE as the eluent to give 0.0622 g (0.34 mmol, 67% yield) of the title compound as a colorless oil. IR (neat, cm^{-1}): 3075, 2977, 2859, 1643, 1604, 1496, 1449; ^{1}H NMR: (400 MHz, $CDCl_3$) δ 7.30-7.24 (m, 2H), 7.21-7.14 (m, 3H), 4.85 (s, 1H), 4.83-4.80 (m, 2H), 4.76 (s, 1H), 2.79-2.72 (m, 4H), 2.28 (t, J = 8.0 Hz, 2H), 1.67 (s, 3H); ^{13}C NMR: (100 MHz, $CDCl_3$) δ 146.7, 143.5, 142.2, 128.32, 128.26, 125.7, 112.3, 111.4, 45.7, 36.9, 34.2, 21.8; HRMS (EI) calculated for $[C_{14}H_{18}]^+$ (M^+) requires m/z 186.1409, found m/z 186.1411.

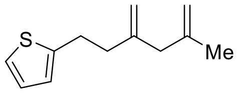 2-(5-Methyl-3-methylenehex-5-en-1-yl)thiophene (**3ae**)

Prepared according to the general procedure B using 0.0044 g (0.025 mmol) of $Co(OAc)_2$, 0.0105 g (0.030 mmol) of **L3**, 180 μL (0.75 mmol) of PMHS, 130 μL (1.057 g/mL, 1.0 mmol) of 2-(but-3-yn-1-yl)thiophene, 52 μL (1.339 g/mL, 0.50 mmol) of 3-bromo-2-methylpropene, 0.0605 g (0.75 mmol) of LiO*t*Bu, and 1.0 mL (0.5 M) of THF. After 4 h, the reaction was worked up. The combined filtrate was concentrated and the regioselectivity (*b*/*l* > 95/5) was monitored by ^{1}H NMR analysis. The crude mixture was purified by flash column chromatography using PE as the eluent to give 0.0535 g (0.28 mmol, 56% yield) of the title compound as a colorless oil. IR (neat, cm^{-1}): 3074, 2974, 2914, 1642, 1534, 1440; ^{1}H NMR: (400 MHz, $CDCl_3$) δ 7.10 (d, J = 5.2 Hz, 1H), 6.93-6.89 (m, 1H), 6.81-6.77 (m, 1H), 4.87 (s, 1H), 4.84 (s, 1H), 4.82 (s, 1H), 4.76 (s, 1H), 2.97 (t, J = 8.0 Hz, 2H), 2.77 (s, 2H), 2.36 (t, J = 8.0 Hz, 2H), 1.68 (s, 3H); ^{13}C NMR: (100 MHz, $CDCl_3$) δ 146.0, 145.0, 143.3, 126.6, 124.0, 122.9, 112.4, 111.9, 45.6, 37.0, 28.2, 21.8; HRMS (EI) calculated for $[C_{12}H_{16}S]^+$ (M^+) requires m/z 192.0973, found m/z 192.0971.

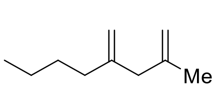 2-Methyl-4-methyleneoct-1-ene (**3af**)

Prepared according to the general procedure using using 0.2483 g (0.50 mmol) of (**L3**-

H)•CoBr, 7.5 mL (40.0 mmol) of $Si(OSiHMe_2)_4$, 6.9 mL (1.339 g/mL, 60.0 mmol) of 1-hexyne, 2.1 mL (1.339 g/mL, 20.0 mmol) of 3-bromo-2-methylpropene, 2.41 g (30.0 mmol) of LiO*t*Bu, and 40.0 mL (0.5 M) of THF. After 3 h, the reaction was worked up. The combined filtrate was concentrated and the regioselectivity (*b*/*l* = 85/15) was monitored by ^{1}H NMR analysis. The crude mixture was purified by distillation to give 4.4236 g (12.8 mmol, 64% yield, 40 ω%) of the title compound as a colorless oil. The boiling range of distillate was 68 °C - 71 °C (~2 mmHg). ^{1}H NMR: (400 MHz, $CDCl_3$) δ 4.81-4.77 (m, 2H), 4.76 (s, 1H), 4.74-4.72 (m, 1H), 2.73 (s, 2H), 2.00-1.94 (m, 2H), 1.67 (s, 3H), 1.45-1.31 (m, 4H), 0.90 (t, *J* = 7.2 Hz, 3H). HRMS (EI) calculated for $[C_{10}H_{18}]^+$ (M^+) requires m/z 138.1409, found m/z 138.1405.

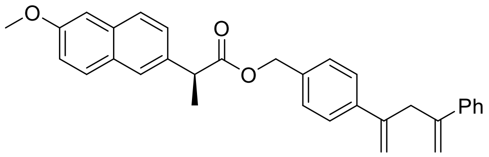

4-(4-Methylpenta-1,4-dien-2-yl)benzyl (*S*)-2-(6-methoxynaphthalen-2-yl)propanoate (**3ag**)

Prepared according to the general procedure A using 0.0055 g (0.010 mmol) of (**L3**-H)•CoBr, 149 μL (0.4 mmol) of $Si(OSiHMe_2)_4$, 0.1376 g (0.4 mmol) of 4-ethynylbenzyl (*S*)-2-(6-methoxynaphthalen-2-yl)propanoate, 29 μL (1.370 g/mL, 0.20 mmol) of (3-bromoprop-1-en-2-yl)benzene, 0.0320 g (0.40 mmol) of LiO*t*Bu, and 0.4 mL (0.5 M) of THF. After 1 h, the reaction was worked up. The combined filtrate was concentrated and the regioselectivity (*b*/*l* = 94/6) was monitored by ^{1}H NMR analysis. The crude mixture was purified by flash column chromatography using PE/EA (20/1) as the eluent to give 0.0637 g (0.14 mmol, 69% yield) of the title compound as a colorless oil. IR (neat, cm^{-1}): 3054, 2974, 2939, 1732, 1606, 1452; ^{1}H NMR: (400 MHz, $CDCl_3$) δ 7.71-7.61 (m, 4H), 7.44-7.30 (m, 7H), 7.20-7.16 (m, 2H), 7.14-7.09 (m, 2H), 5.47-5.44 (m, 2H), 5.14-5.07 (m, 4H), 3.92-3.89 (m, 4H), 3.63 (s, 2H), 1.59 (d, J = 7.2 Hz, 3H); ^{13}C NMR: (100 MHz, $CDCl_3$) δ 174.4, 157.6, 145.2, 144.8, 140.9, 140.7, 135.5, 135.1, 133.7, 132.2, 129.3, 128.9, 128.2, 127.9, 127.4, 127.1, 126.3, 126.0, 125.9, 118.9, 115.1, 114.8, 105.6, 66.2, 55.3, 45.4, 40.8, 18.5; HRMS (ESI) calculated for $[C_{32}H_{30}NaO_3]^+$ $(M+Na)^+$ requires m/z 485.2087, found m/z 485.2090.

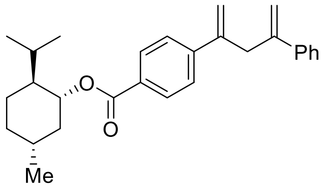

(*1R,2S,5R*)-2-isopropyl-5-methylcyclohexyl 4-(4-phenylpenta-1,4-dien-2-yl)benzoate (**3ag**)

Prepared according to the general procedure A using 0.0055 g (0.010 mmol) of (**L3**-H)•CoBr, 149 μL (0.4 mmol) of $Si(OSiHMe_2)_4$, 0.1141 g (0.4 mmol) of (*1R,2S,5R*)-2-isopropyl-5-methylcyclohexyl 4-ethynylbenzoate, 29 μL (1.370 g/mL, 0.20 mmol) of (3-bromoprop-1-en-2-yl)benzene, 0.0323 g (0.40 mmol) of LiO*t*Bu, and 0.4 mL (0.5 M) of THF. After 1 h, the reaction was worked up. The combined filtrate was concentrated and the regioselectivity (*b*/*l* = 91/9) was monitored by ^{1}H NMR analysis. The crude mixture was purified by flash column chromatography using PE as the eluent to give 0.0545 g (0.13 mmol, 67% yield) of the title compound as a colorless oil. IR (neat, cm^{-1}): 3055, 2954, 2868, 1711, 1608, 1454; ^{1}H NMR: (400 MHz, $CDCl_3$) δ 8.02-7.96 (m, 2H), 7.51-7.46 (m, 2H), 7.44-7.39 (m, 2H), 7.34-7.26 (m, 3H), 5.55 (s, 1H), 5.46 (s, 1H), 5.24 (s, 1H), 5.11 (s, 1H), 3.69 (s, 2H), 2.16-2.08 (m, 1H), 2.00-1.90 (m, 1H), 1.99-1.92 (m, 1H), 1.76-1.66 (m, 2H), 1.58-1.52 (m, 2H), 0.95-0.89 (m, 8H), 0.79 (d, J = 6.8 Hz, 2H); ^{13}C NMR: (100 MHz, $CDCl_3$) δ 165.9, 145.2, 145.1, 144.7, 140.8, 132.0, 129.6, 128.3, 127.5, 126.0, 125.9, 116.7, 115.0, 74.7, 47.3, 41.0, 40.8, 34.3, 31.4, 26.5, 23.6, 22.0, 20.7, 16.5;HRMS (ESI) calculated for $[C_{28}H_{34}NaO_2]^+$ $(M+Na)^+$ requires m/z 425.2451, found m/z 425.2448.

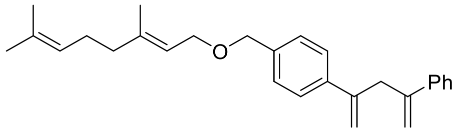

(*E*)-1-(((3,7-dimethylocta-2,6-dien-1-yl)oxy)methyl)-4-(4-phenylpenta-1,4-dien-2-yl)benzene (**3ai**)

Prepared according to the general procedure A using 0.0130 g (0.025 mmol) of (**L3**-H)•CoBr, 180 μL (0.75 mmol) of PMHS, 0.2671 g (1.0 mmol) of (*E*)-1-(((3,7-dimethylocta-2,6-dien-1-yl)oxy)methyl)-4-ethynylbenzene, 72 μL (1.370 g/mL, 0.50 mmol) of (3-bromoprop-1-en-2-yl)benzene, 0.0600 g (0.75 mmol) of LiO*t*Bu, and 1.0 mL (0.5 M) of THF. After 1 h, the reaction was worked up. The combined filtrate was concentrated and the regioselectivity (*b*/*l* = 90/10) was monitored by ^{1}H NMR analysis. The crude mixture was purified by flash column chromatography using PE as the eluent to give 0.0875 g (0.23 mmol, 45% yield) of the title compound as a colorless oil. IR

(neat, cm^{-1}): 3083, 3055, 2967, 2858, 1806, 1719, 1673, 1446; ^{1}H NMR: (400 MHz, $CDCl_3$) δ 7.46-7.39 (m, 4H), 7.35-7.27 (m, 5H), 5.47 (d, J = 5.2 Hz, 2H), 5.40 (t, J = 6.4 Hz, 1H), 5.16-5.07 (m, 3H), 4.49 (s, 2H), 4.03 (d, J = 6.8 Hz, 2H), 3.66 (s, 2H), 2.15-2.01 (m, 4H), 1.68 (s, 3H), 1.65 (s, 3H), 1.60 (s, 3H); ^{13}C NMR: (100 MHz, $CDCl_3$) δ 145.3, 145.1, 140.9, 140.4, 140.2, 137.8, 131.7, 128.2, 127.8, 127.4, 126.0, 124.0, 120.8, 114.8, 114.7, 71.6, 66.6, 40.9, 39.6, 26.3, 25.7, 17.7, 16.5; HRMS (ESI) calculated for $[C_{28}H_{34}NaO]^+$ $(M+Na)^+$ requires m/z 409.2502, found m/z 409.2500.

VI. Gram Scale Reaction and synthetic applications

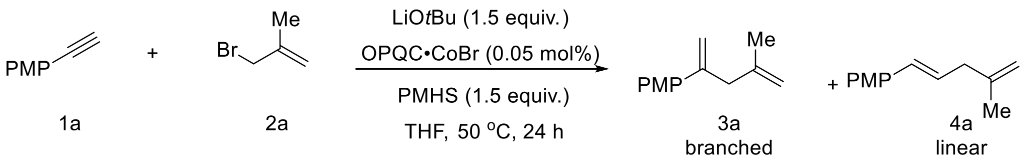

Supplementary Figure 1. Gram-Scale Reaction.

100 mL Schlenk flask equipped with a magnetic stirrer and a flanging rubber plug was dried with flame under vacuum. When cooled to ambient temperature, it was vacuumed and flushed with N_2. This degassed procedure was repeated for three times. Then 0.0050 g (0.00025 mmol, 0.05 mol %) of (**L3**-H)•CoBr, 40.0 mL(0.5 M) of THF, 7.2 mL (30.0 mmol, 1.5 equiv.) of PMHS, 4.4 mL (40.0 mmol, 2 equiv.) of terminal alkynes, 2.1 mL (20.0 mmol, 1.0 equiv.) of allylic bromides, and 2.40 g (30.0 mmol, 1.5 equiv.) of LiO*t*Bu were added sequentially. The reaction was run at 50 °C for 24 h. Then the resulting solution was quenched with 50mL of PE and filtered through a pad of silica gel, washed with PE/EtOAc (5/1) (3 x 20 mL). The combined filtrate was concentrated under vacuum. 58% NMR yield with 88/12 *b*/*l* of the skipped diene was obtained by ^{1}H NMR analysis.

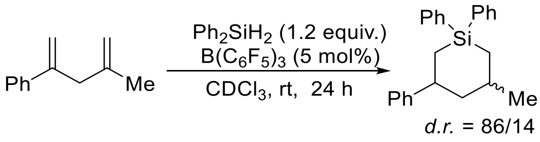

Supplementary Figure 2. The synthesis of silyl heterocycle.

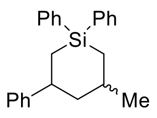

3-Methyl-1,1,5-triphenylsilinane **(5)**

Prepared according to the previously reported procedures.[19] A 25 mL

Schlenk flask equipped with a magnetic stirrer and a flanging rubber plug was dried with flame under vacuum. When cooled to ambient temperature, it was vacuumed and flushed with N_2. This degassed procedure was repeated for three times. Then 0.0285 g (0.05 mmol, 0.05 equiv.) of tris(pentafluorophenyl)borane, 1.0 mL of $CDCl_3$, 223 μL of (1.2 mmol, 2.0 equiv.) Ph_2SiH_2, and 176 μL of (1.0 mmol, 1.0 equiv.) (4-methylpenta-1,4-dien-2-yl)benzene were added sequentially. The mixture was stirred for 24 h. The combined filtrates were concentrated and purified by flash column chromatography using PE as the eluent to give 0.2090 g (0.61 mmol, 61% yield, *d.r.* = 86/14) of the title compound as a white solid. ^1H NMR: (400 MHz, $CDCl_3$) δ 7.71-7.65 (m, 2H), 7.45-7.39 (m, 5H), 7.34-7.25 (m, 7H), 7.21-7.15 (m, 1H), 3.14-3.04 (m, 1H), 2.55-2.45 (m, 1H), 1.82 (ddd, J = 25.2, 13.6, 4.0 Hz, 1H), 1.76-1.64 (m, 2H), 1.32-1.25 (m, 3H), 0.96 (d, J = 7.2 Hz, 3H); ^{13}C NMR: (100 MHz, $CDCl_3$) δ 150.2, 137.9, 136.8, 134.5, 134.3, 129.2, 129.1, 128.4, 128.0, 127.8, 126.5, 125.7, 43.7, 35.6, 28.6, 22.5, 18.7, 16.8; The NMR spectra were consistent with the spectra reported in the literature.[19]

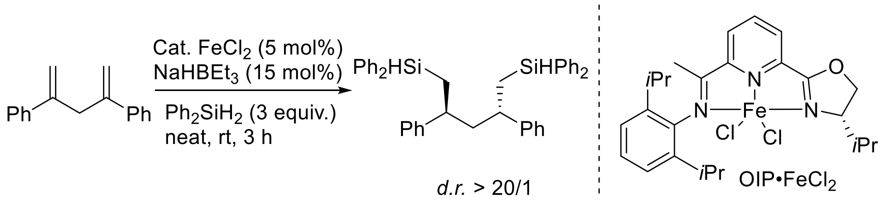

Supplementary Figure 3. The synthesis of 1,5-disilyl compound.

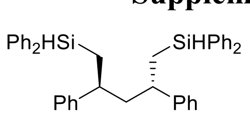

(2,4-Diphenylpentane-1,5-diyl)bis(diphenylsilane) (**6**)

Prepared according to the previously reported procedures.[22] In a nitrogen-filled glovebox, an oven-dried 25 mL vial that contained a magnetic stir bar was charged with 0.0132 g (0.025 mmol, 5 mol %) of OIP•$FeCl_2$, 75 μL of $NaBHEt_3$ (0.075 mmol,15 mol %, 1.0 M/L in THF), 0.1105 g (0.50 mmol, 1.0 equiv.) of alkene, and 278 μL (1.50 mmol, 3.0 equiv.) of Ph_2SiH_2 sequentially. The mixture was stirred for 3 h. Then the resulting solution was transferred into room and quenched with 10 mL of PE. The reaction mixture was filtered through a short pad of silica gel, with PE/EA (5/1) (3 x 20 mL) as the eluent. The combined filtrate was concentrated under vacuum

and the NMR yield and regioselectivity were monitored by crude ^{1}H NMR analysis. Then, the residue was purified by chromatography using PE as the eluent to give 0.1937 g (0.33mmol, 66% yield, *d.r.* > 20/1) of the corresponding product. IR (neat, cm^{-1}): 3063, 3023, 2914, 2124, 1956, 1682, 1597, 1491, 1428; ^{1}H NMR: (400 MHz, $CDCl_3$) δ 7.41-7.37 (m, 4H), 7.36-7.31 (m, 3H), 7.30-7.25 (m, 9H), 7.23-7.20 (m, 4H), 7.15-7.10 (m, 6H), 6.85-6.80 (m, 4H), 4.61 (t, J = 4.0 Hz, 2H), 2.49-2.39 (m, 2H), 2.10 (t, J = 7.2 Hz, 2H), 1.45-1.30 (m, 4H); ^{13}C NMR: (100 MHz, $CDCl_3$) δ 146.4, 135.1, 134.9, 134.4, 134.4, 129.4, 129.2, 128.2, 127.9, 127.8, 127.5, 125.9, 47.8, 39.3, 22.2.

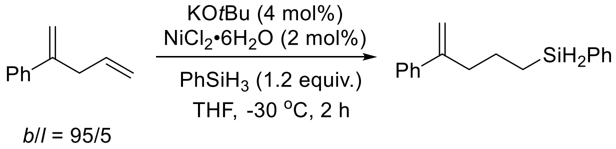

Supplementary Figure 4. Selective hydrosilylation of skipped diene.

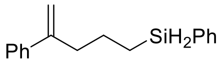

Phenyl(4-phenylpent-4-en-1-yl)silane (**7**)

Prepared according to the previously reported procedures[23] with modification. A dried Schlenk flask containing a magnetic stir bar was charged with 0.0097 g (0.04 mmol, 2.0 mol%) of $NiCl_2 \cdot 6H_2O$, 0.0098 g (0.08 mmol, 4.0 mol%) of *t*BuOK, 0.2884 g (2.0 mmol, 1.0 equiv.) of alkene, 0.2597 g of (2.4 mmol, 1.2 equiv.) $PhSiH_3$, and 3.0 mL of THF under N_2 atmosphere. Then the reaction was stirred at -30 °C for 2 h. The yield was monitored by TLC. The reaction mixture was filtered through a short pad of silica gel, with PE/EA (5/1) (3 x 20 mL) as the eluent. The combined filtrate was concentrated under vacuum. Then, the residue was purified by chromatography using PE as the eluent to give 0.4147 g (1.64 mmol, 82% yield) of the corresponding product. IR (neat, cm^{-1}): 3022, 3909, 2127, 1597, 1491, 1404; ^{1}H NMR: (400 MHz, $CDCl_3$) δ 7.55-7.49 (m, 2H), 7.40-7.27 (m, 8H), 5.29-5.26 (m, 1H), 5.06-5.02 (m, 1H), 4.27 (t, J = 3.6 Hz, 2H), 2.57 (t, J = 7.2 Hz, 2H), 1.67-1.57 (m, 2H), 1.01-0.93 (m, 2H); ^{13}C NMR: (100 MHz, $CDCl_3$) δ 147.9, 141.1, 135.1, 132.4, 129.5, 128.2, 127.9, 127.3, 126.1, 112.6, 38.2, 23.6, 9.5; HRMS (EI) calculated for $[C_{17}H_{20}Si]^+$ (M^+) requires m/z 252.1334, found m/z 252.1333.

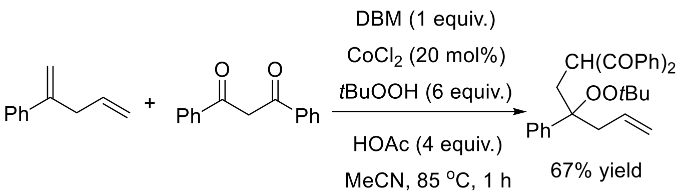

Supplementary Figure 5. Selective alkylation-peroxidation of skipped diene.

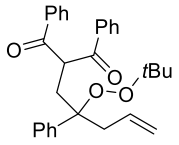

2-(2-(*Tert*-butylperoxy)-2-phenylpent-4-en-1-yl)-1,3-diphenylpropane-1,3-dione (**8**)

Prepared according to the previously reported procedures.[24] A dried Schlenk flask containing a magnetic stir bar was charged with 0.2163 g (1.5 mmol, 3.0 equiv.) of penta-1,4-dien-2-ylbenzene, 0.1153 g (0.5 mmol, 1.0 equiv.) of dibenzoylmethane, 0.0132 g (0.1 mmol, 0.2 equiv.) of cobalt dichloride, 0.27 g (3.0 mmol, 6.0 equiv.)of *tert*-butyl hydroperoxide, and 114 μL (2.0 mmol, 4 equiv.) of acetic acid under N_2 atmosphere. Then the reaction was stirred at 85 ºC for 1 h. The yield was monitored by TLC. The reaction mixture was filtered through a short pad of silica gel, with PE/EA (5/1) (3 x 20 mL) as the eluent. The combined filtrate was concentrated under vacuum. The crude mixture was purified by flash column chromatography using PE/EtOAc (20/1) as the eluent to give **8** as a colorless oil with 67% yield (0.34 mmol, 0.1524 g). ^{1}H NMR: (400 MHz, $CDCl_3$) δ 7.96-7.91 (m, 2H), 7.54-7.49 (m, 1H), 7.45-7.39 (m, 5H), 7.35-7.32 (m, 2H), 7.28-7.20 (m, 5H), 5.86-5.74 (m, 1H), 5.41 (t, J = 5.2 Hz, 1H), 5.02 (d, J = 5.2 Hz, 1H), 4.99 (s, 1H), 3.06 (dd, J = 6.0, 14.8 Hz, 1H), 2.91-2.77 (m, 2H), 2.55 (dd, J = 4.8, 14.8 Hz, 1H), 1.19 (s, 9H); ^{13}C NMR: (100 MHz, $CDCl_3$) δ 195.3, 194.6, 142.2, 136.3, 135.7, 133.8, 133.1, 132.9, 128.8, 128.6, 128.5, 128.4, 128.0, 127.0, 126.5, 117.8, 85.2, 79.6, 52.1, 42.0, 37.9, 26.6; The NMR spectra were consistent with the spectra reported in the literature.[24]

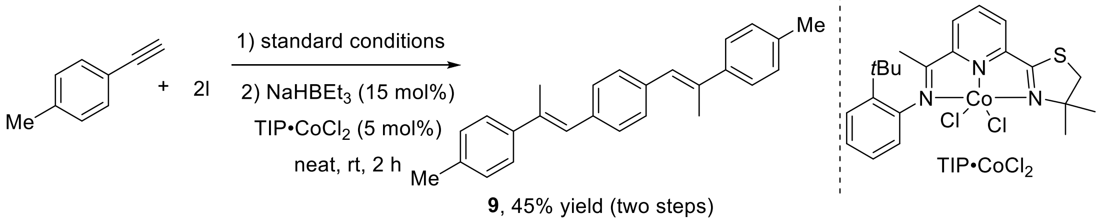

9, 45% yield (two steps)

Supplementary Figure 6. Synthesis of conjugated alkene with AIE properties.

Prepared according to the general procedure B using 0.0440 g (0.025 mmol) of $Co(OAc)_2$, 0.1040 g (0.030 mmol) of **L3**, 3.6 mL (15.0 mmol) of PMHS, 2.8 mL (0.916 g/mL, 20 mmol) of 1-ethynyl-4-methylbenzene, 1.3243 g (5.0 mmol) of 1,4-bis(bromomethyl)benzene, 1.1578 g (15 mmol) of LiO*t*Bu, and 10.0 mL (0.5 M) of THF. After 4 h, the reaction was worked up. The crude mixture was purified by flash column chromatography using PE as the eluent to give 1.03 g (3.05 mmol, 61% yield) of the alkene. Next, prepared according to the general procedure.[25] In a nitrogen-filled glovebox, an oven-dried 25 mL vial that contained a magnetic stir bar was charged with TIP•$CoCl_2$ (0.0074 g, 0.015 mmol, 5 mol %), $NaBHEt_3$ (45 μL, 0.045 mmol, 15 mol %, 1 M/L in THF), alkene (0.3 mmol, 1.0 equiv.), and THF (0.3 mL) sequentially. The mixture was stirred for 2 h. Then the resulting solution was transferred into room and quenched with 10 mL of PE. The reaction mixture was filtered through a short pad of silica gel, with DCM (50 mL) as the eluent. The combined filtrate was concentrated under vacuum and the NMR yield and regioselectivity were monitored by crude ^1H NMR analysis. Then, the residue was purified by chromatography using PE/DCM (50/1) as the eluent to give 0.0264 g (0.22 mmol, 73% yield) of corresponding product. The final product was obtained with 45% yield in two steps. ^1H NMR: (400 MHz, $CDCl_3$) δ 7.44 (d, J = 8.0 Hz, 4H), 7.39-7.36 (m, 4H), 7.18 (d, J = 8.0 Hz, 4H), 6.82 (s, 2H), 2.37 (s, 6H), 2.31 (s, 6H); ^{13}C NMR: (100 MHz, $CDCl_3$) δ 141.2, 137.2, 136.9, 136.5, 129.0, 128.9, 126.8, 125.8, 21.1, 17.6; The NMR spectra were consistent with the spectra reported in the literature.[21]

VII. Mechanistic studies and Control experiments

Isotopic labeling experiments

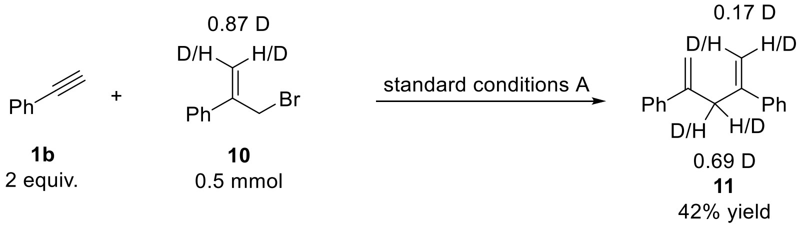

Supplementary Figure 7. The reaction with deuterium labeling allylic bromide.

Prepared according to the general procedure A using 0.0124 g (0.025 mmol) of (**L3-**H)•CoBr, 180 μL (0.75 mmol) of PMHS, 110 μL (0.930 g/mL, 1.0 mmol) of phenylacetylene, 72 μL (1.400 g/mL, 0.50 mmol) of **10**, 0.0601 g (0.75 mmol) of LiO*t*Bu, and 1.0 mL (0.5 M) of THF. After 1 h, the reaction was worked up. The crude mixture was purified by flash column chromatography using PE as the eluent to give 0.0460 g (0.21 mmol, 42% yield) of the title compound as a colorless oil. ^{1}H NMR: (400 MHz, $CDCl_3$) δ 7.45-7.42 (m, 4H), 7.34-7.27 (m, 6H), 5.50-5.46 (m, 1.94H), 5.15-5.12 (m, 1.89H), 3.69-3.65 (m, 1.31H); ^{2}H NMR: (77 MHz, $CDCl_3$) δ 5.60-5.50 (m, 0.06D), 5.28-5.15 (m, 0.11D), 3.80-3.60 (m, 0.69D).

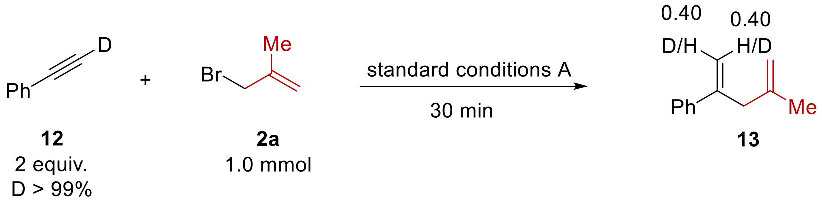

Supplementary Figure 8. The reaction with deuterium labeling terminal alkynes.

Prepared according to the general procedure A using 0.0248 g (0.050 mmol) of (**L3-**H)•CoBr, 360 μL (1.50 mmol) of PMHS, 220 μL (0.930 g/mL, 1.0 mmol) of (ethynyl-d)benzene, 106 μL (1.339 g/mL, 0.50 mmol) of **2a**, 0.1200 g (1.50 mmol) of LiO*t*Bu, and 2.0 mL (0.5 M) of THF. After 30 min, the reaction was worked up. The crude mixture was purified by flash column chromatography using PE as the eluent to give 0.0927 g (0.59 mmol, 59% yield) of the title compound as a colorless oil. ^{1}H NMR: (400 MHz, $CDCl_3$) δ 7.45-7.42 (m, 2H), 7.34-7.28 (m, 2H), 7.27-7.24 (m, 1H), 5.44-5.41 (s, 0.60H), 5.12-5.09 (s, 0.60H), 4.81 (s, 1H), 4.76 (s, 1H), 3.21 (s, 2H), 1.72 (s, 3H); ^{2}H NMR: (77 MHz, $CDCl_3$) δ 5.58-5.47 (m, 0.40D), 5.25-5.13 (m, 0.40D).

Radical-trapping experiment

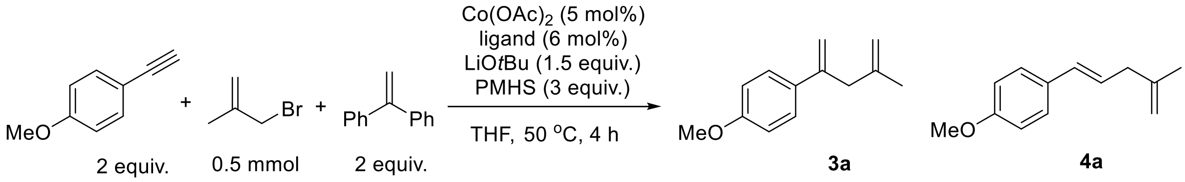

Supplementary Figure 9. The hydroallylation reaction under additional 1,1-diphenylethylene radical scavenger.

(a) Prepared according to the general procedure A using 0.0045 g (0.025 mmol) of $Co(OAc)_2$, 0.0104 g (0.030 mmol) of **L3**, 180 μL (0.75 mmol) of PMHS, 130 μL (1.019 g/mL, 1 mmol) of 1-ethynyl-4-methoxybenzene, 53 μL (1.339 g/mL, 0.5 mmol) of 3-bromo-2-methylpropene, 0.0600 g (0.75 mmol) of LiO*t*Bu, and 1.0 mL (0.5 M) of THF, with additionally 177 μL (1.0 mmol) of 1,1-diphenylethylene as a radical scavenger. After 4 h, the reaction was worked up, 68% yield of skipped diene with > 95/5 ratio of *b*/*l* was detected from ^{1}H NMR.

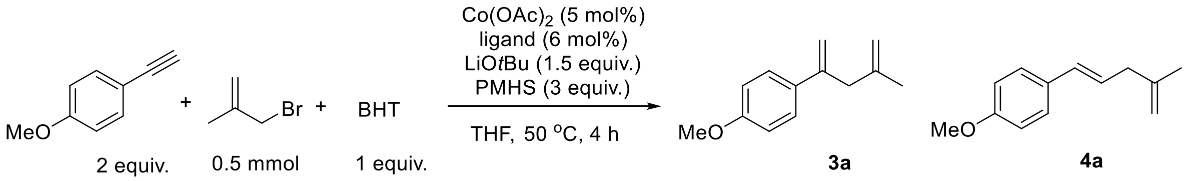

Supplementary Figure 10. The hydroallylation reaction under additional 2,6-di-*tert*-butyl-4-methylphenol radical scavenger.

(b) Prepared according to the general procedure A using 0.0044 g (0.025 mmol) of $Co(OAc)_2$, 0.0106 g (0.030 mmol) of **L3**, 180 μL (0.75 mmol) of PMHS, 130 μL (1.019 g/mL, 1 mmol) of 1-ethynyl-4-methoxybenzene, 53 μL (1.339 g/mL, 0.50 mmol) of 3-bromo-2-methylpropene,0.0600 g (0.75 mmol) of LiO*t*Bu, and 1.0 mL (0.5 M) of THF, with additionally 0.1103 g (0.5 mmol) of 2,6-di-*tert*-butyl-4-methylphenol (BHT) as a radical scavenger. After 4 h, the reaction was worked up, 72% yield of skipped diene with > 95/5 ratio of *b*/*l* was detected from ^{1}H NMR.

VIII. Kinetic Studies

General procedure to determine the dependence of reaction rate on the concentration of alkyne:

For the reaction of alkyne (0.50 M), allylic bromide (0.25 M), PMHS (0.375 M), and (**L3**-H)•CoBr (0.0125 M): A 25 mL Schlenk flask equipped with a magnetic stirrer and a flanging rubber plug was dried with flame under vacuum. When cooled to ambient temperature, it was vacuumed and flushed with N_2. This degassed procedure was repeated for three times. Then (**L3**-H)•CoBr (0.1 mmol, 5 mol %), THF (8.0 mL, 0.25 M), PMHS (3.0 mmol, 1.5 equiv.), terminal alkynes (4.0 mmol, 2 equiv.), internal

standard (mesitylene, 40 μL), allylic bromides (2 mmol, 1.0 equiv.), and LiO*t*Bu (3.0 mmol, 1.5 equiv.) were added sequentially. 100 μL of the reaction mixture was taken out at 120, 240, 360, 480, 600 s and quenched with $CDCl_3$ immediately under air. The sample was analyzed by ^{1}H NMR.

For the reaction of alkyne (0.25 M), allylic bromide (0.25 M), PMHS (0.375 M), and (**L3**-H)•CoBr (0.0125 M): The procedure for this reaction was the same as above but instead of 1 equiv. of alkyne (0.25 M).

For the reaction of alkyne (0.375 M), allylic bromide (0.25 M), PMHS (0.375 M), and (**L3**-H)•CoBr (0.0125 M): The procedure for this reaction was the same as above but instead of 1.5 equiv. of alkyne (0.375 M).

For the reaction of alkyne (0.625 M), allylic bromide (0.25 M), PMHS (0.375 M), and (**L3**-H)•CoBr (0.0125 M): The procedure for this reaction was the same as above but instead of 2.5 equiv. of alkyne (0.625 M).

The molar concentrations of the product **3a** were calculated by integrating against mesitylene as an internal standard. The molar concentration of product **3a** (only the data corresponding to the linear portion of the graph, typically < 20% yield, was used) was plotted against the reaction time and the slope of linear portion of the curve was used to determine the initial rates of this transformation. The table showing molar concentration of product **3a** in different concentration of alkyne, graph showing the rate at different concentration of alkyne, table with k_{in} value and the graph showing k_{in} versus [alkyne] are shown below:

Supplementary Table 2. The molar concentration of product **3a** in different concentration of alkyne at different time interval

Time (s)	0.250 M [alkyne]	0.375 M [alkyne]	0.500 M [alkyne]	0.625 M [alkyne]
120	0.010675	0.011475	0.010850	0.008550
240	0.023175	0.021250	0.022575	0.015075
360	0.034050	0.035725	0.032125	0.022850
480	0.047400	0.043350	0.040700	0.030600
600	0.062275	0.055150	0.046750	0.035550

Supplementary Table 3. The k_{in} value of product **3a** in different concentration of alkyne

[alkyne] M	k_{in}/Ms^{-1}
0.250 M	1.06 x 10^{-4}
0.375 M	9.13 x 10^{-5}
0.500 M	7.50 x 10^{-5}
0.625 M	5.76 x 10^{-5}

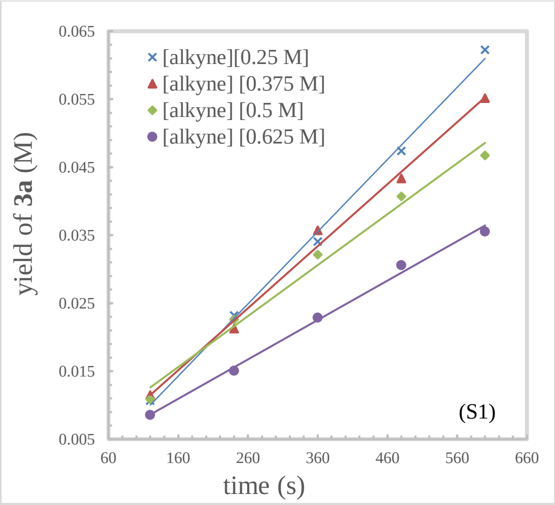

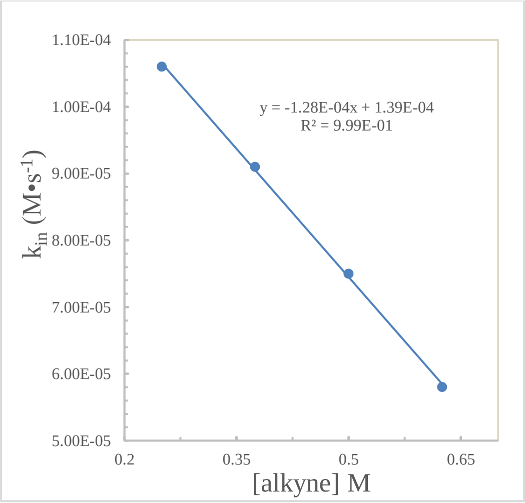

Supplementary Figure 11. Plot of the rise of product a from the reaction of allylic bromide (0.25 M), PMHS (0.375 M), (**L3**-H)•CoBr (0.0125 M) with 0.25 M, 0.375 M, 0.5 M, and 0.625 M of alkyne in different time interval at 50 °C. **Supplementary Figure 12**. Plot of k_{in} versus [alkyne] from the reaction of allylic bromide (0.25 M), PMHS (0.375 M), (**L3**-H)•CoBr (0.0125 M) with 0.25 M, 0.375 M, 0.50 M, and 0.625 M of alkyne.

General procedure to determine the dependence of reaction rate on the concentration of allylic bromide:

For the reaction of alkyne (0.20 M), allylic bromide (0.10 M), PMHS (0.15 M), and (**L3**-H)•CoBr (0.005 M): A 25 mL Schlenk flask equipped with a magnetic stirrer and a flanging rubber plug was dried with flame under vacuum. When cooled to ambient temperature, it was vacuumed and flushed with N_2. This degassed procedure was repeated for three times. Then (**L3**-H)•CoBr (0.05 mmol, 5 mol %), THF (10.0 mL, 0.25 M), PMHS (1.5 mmol, 1.5 equiv.), terminal alkynes (2.0 mmol, 2 equiv.), internal standard (mesitylene, 20 μL), allylic bromides (1 mmol, 1.0 equiv.), and LiO*t*Bu (1.5 mmol, 1.5 equiv.) were added sequentially. 100 μL of the reaction mixture was taken out at 150, 300, 450, 600, 750 s and quenched with $CDCl_3$ immediately under air. The sample was analyzed by ^{1}H NMR.

For the reaction of allylic bromide (0.075 M), alkyne (0.20 M), PMHS (0.15 M), and (**L3**-H)•CoBr (0.005 M): The procedure for this reaction was the same as above but instead of 0.75 equiv. of allylic bromide (0.075 M).

For the reaction of allylic bromide (0.125 M), alkyne (0.20 M), PMHS (0.15 M), and (**L3**-H)•CoBr (0.005 M): The procedure for this reaction was the same as above but

instead of 1.25 equiv. of allylic bromide (0.125 M).

For the reaction of allylic bromide (0.15 M), alkyne (0.20 M), PMHS (0.15 M), and (**L3**-H)•CoBr (0.005 M): The procedure for this reaction was the same as above but instead of 1.50 equiv. of allylic bromide (0.150 M).

The molar concentrations of the product **3a** were calculated by integrating against mesitylene as an internal standard. The molar concentration of product **3a** (only the data corresponding to the linear portion of the graph, typically < 20% yield, was used) was plotted against the reaction time and the slope of linear portion of the curve was used to determine the initial rates of this transformation. The table showing molar concentration of product **3a** in different concentration of allylic bromide, graph showing the rate at different concentration of allylic bromide, table with k_{in} value and the graph showing k_{in} versus [allylic bromide] are shown below:

Supplementary Table 4. The molar concentration of product **3a** in different concentration of allylic bromide at different time interval

Time (s)	0.075 M [allylic bromide]	0.100 M [allylic bromide]	0.125 M [allylic bromide]	0.150 M [allylic bromide]
150	0.001660	0.001725	0.002365	0.003420
300	0.003760	0.003675	0.004510	0.006465
450	0.005830	0.005785	0.006880	0.010095
600	0.007025	0.007680	0.009125	0.013020
750	0.008925	0.010085	0.012740	0.015710

Supplementary Table 5. The k_{in} value of product **3a** in different concentration of allylic bromide

[allylic bromide] M	k_{in}/Ms^{-1}
0.075 M	1.19 x 10^{-5}
0.100 M	1.38 x 10^{-5}
0.125 M	1.69 x 10^{-5}
0.150 M	2.08 x 10^{-5}

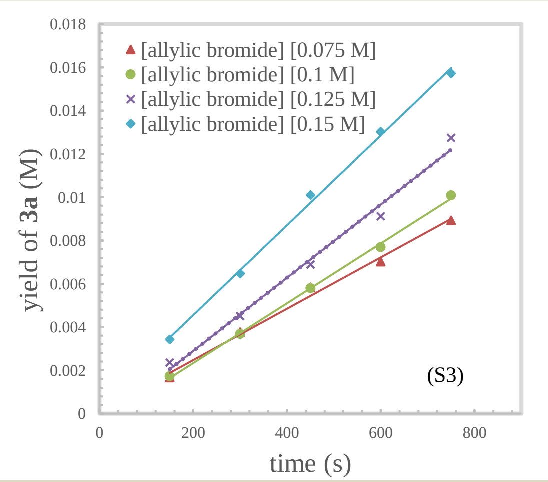

(S3)

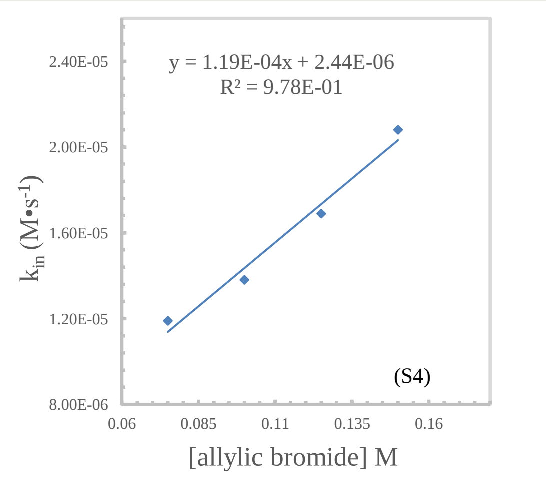

(S4)

Supplementary Figure 13. Plot of the rise of product a from the reaction of alkyne (0.20 M), PMHS (0.15 M), (**L3**-H)•CoBr (0.005 M) with 0.075 M, 0.100 M, 0.125 M, and 0.150 M of allylic bromide in different time interval at 50 ºC. **Supplementary Figure 14**. Plot of k_{in} versus [allylic bromide] from the reaction of alkyne (0.20 M), PMHS (0.15 M), (**L3**-H)•CoBr (0.005 M) with 0.075 M, 0.100 M, 0.125 M, and 0.150 M of allylic bromide.

General procedure to determine the dependence of reaction rate on the concentration of (L3-H)•CoBr:

For the reaction of alkyne (0.50 M), allylic bromide (0.25 M), PMHS (0.375 M), and (**L3**-H)•CoBr (0.0125 M): A 25 mL Schlenk flask equipped with a magnetic stirrer and a flanging rubber plug was dried with flame under vacuum. When cooled to ambient temperature, it was vacuumed and flushed with N_2. This degassed procedure was repeated for three times. Then (**L3**-H)•CoBr (0.1 mmol, 5 mol %), THF (8.0 mL, 0.25 M), PMHS (3.0 mmol, 1.5 equiv.), terminal alkynes (4.0 mmol, 2 equiv.), internal standard (mesitylene, 40 μL), allylic bromides (2 mmol, 1.0 equiv.), and LiO*t*Bu (3.0 mmol, 1.5 equiv.) were added sequentially. 100 μL of the reaction mixture was taken out at 120, 240, 360, 480, 600 s and quenched with $CDCl_3$ immediately under air. The sample was analyzed by ^{1}H NMR.

For the reaction of alkyne (0.50 M), allylic bromide (0.25 M), PMHS (0.375 M), and (**L3**-H)•CoBr (0.00625 M): The procedure for this reaction was the same as above but instead of 2.5 mol% of (**L3**-H)•CoBr (0.00625 M).

For the reaction of alkyne (0.5 M), allylic bromide (0.25 M), PMHS (0.375 M), and (**L3**-H)•CoBr (0.01875 M): The procedure for this reaction was the same as above but instead of 7.5 mol% of (**L3**-H)•CoBr (0.01875 M).

The molar concentrations of the product **3a** were calculated by integrating against mesitylene as an internal standard. The molar concentration of product **3a** (only the data corresponding to the linear portion of the graph, typically < 20% yield, was used) was plotted against the reaction time and the slope of linear portion of the curve was used to determine the initial rates of this transformation. The table showing molar concentration of product **3a** in different concentration of (**L3**-H)•CoBr, graph showing the rate at different concentration of (**L3**-H)•CoBr, table with k_{in} value and the graph showing kin versus [(**L3**-H)•CoBr] are shown below:

Supplementary Table 6. The molar concentration of product **3a** in different

concentration of (**L3**-H)•CoBr at different time interval

Time (s)	0.00625 [(**L3**-H)•CoBr]	0.01250 [(**L3**-H)•CoBr]	0.01875 [(**L3**-H)•CoBr]
120	0.005600	0.014350	0.015950
240	0.008725	0.027650	0.028425
360	0.013250	0.035050	0.045200
480	0.016925	0.040700	0.060100
600	0.021375	0.053350	0.070925

Supplementary Table 7. The k_{in} value of product **3a** in different concentration of (**L3**-H)•CoBr

[(**L3**-H)•CoBr] M	k_{in}/Ms^{-1}
0.00625 M	3.31 x 10^{-5}
0.01250 M	7.59 x 10^{-5}
0.01875 M	1.18 x 10^{-4}

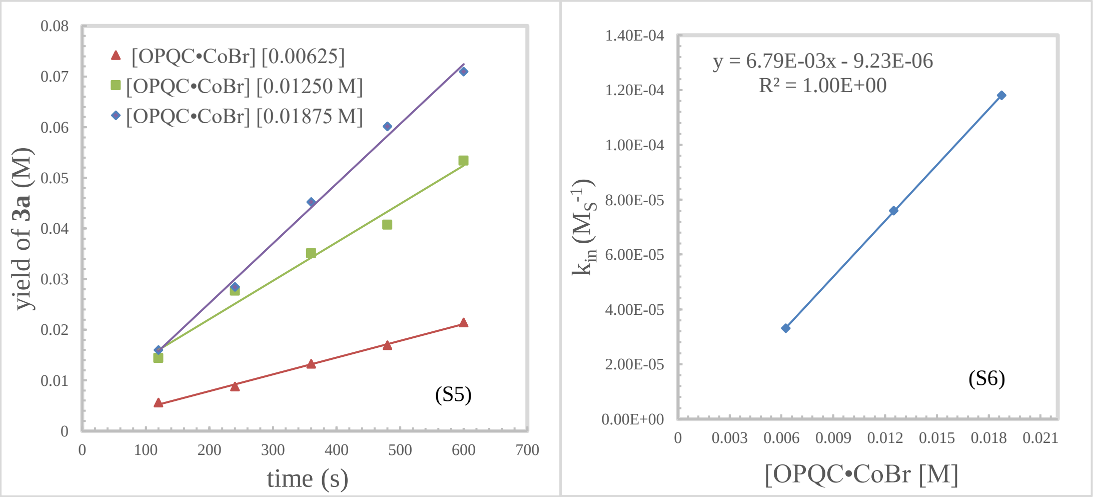

Supplementary Figure 15. Plot of the rise of product a from the reaction of alkyne (0.50 M), allylic bromide (0.25 M), and PMHS (0.375 M) with 0.00625 M, 0.01250 M, and 0.01875 M of (**L3**-H)•CoBr in different time interval at 50 °C. **Supplementary Figure 16**. Plot of k_{in} versus [(**L3**-H)•CoBr] from the reaction of alkyne (0.50 M), allylic bromide (0.25 M), PMHS (0.375 M) with 0.00625 M, 0.01250 M, and 0.01875 M of (**L3**-H)•CoBr.

General procedure to determine the dependence of reaction rate on the concentration of PMHS:

For the reaction of alkyne (0.20 M), allylic bromide (0.10 M), PMHS (0.25 M), and (**L3**-H)•CoBr (0.05 M): A 25 mL Schlenk flask equipped with a magnetic stirrer and a flanging rubber plug was dried with flame under vacuum. When cooled to ambient temperature, it was vacuumed and flushed with N_2. This degassed procedure was repeated for three times. Then (**L3**-H)•CoBr (0.05 mmol, 5 mol %), THF (10.0 mL, 0.1

M), PMHS (2.5 mmol, 2.5 equiv.), terminal alkynes (2.0 mmol, 2.0 equiv.), internal standard (mesitylene, 20 μL), allylic bromides (1 mmol, 1.0 equiv.), and LiO*t*Bu (1.5 mmol, 1.5 equiv.) were added sequentially. 100 μL of the reaction mixture was taken out at 180, 360, 540, 720, 900 s and quenched with $CDCl_3$ immediately under air. The sample was analyzed by ^1H NMR.

For the reaction of alkyne (0.20 M), allylic bromide (0.10 M), PMHS (0.35 M), and (**L3**-H)•CoBr (0.005 M): The procedure for this reaction was the same as above but instead of 3.5 equiv. of PMHS (0.35 M).

For the reaction of alkyne (0.20 M), allylic bromide (0.10 M), PMHS (0.45 M), and (**L3**-H)•CoBr (0.005 M): The procedure for this reaction was the same as above but instead of 3.5 equiv. of PMHS (0.45 M).

For the reaction of alkyne (0.20 M), allylic bromide (0.10 M), PMHS (0.55 M), and (**L3**-H)•CoBr (0.005 M): The procedure for this reaction was the same as above but instead of 5.5 equiv. of PMHS (0.55 M).

The molar concentrations of the product **3a** were calculated by integrating against mesitylene as an internal standard. The molar concentration of product **3a** (only the data corresponding to the linear portion of the graph, typically < 20% yield, was used) was plotted against the reaction time and the slope of linear portion of the curve was used to determine the initial rates of this transformation. The table showing molar concentration of product **3a** in different concentration of PMHS, graph showing the rate at different concentration of PMHS, table with k_{in} value and the graph showing k_{in} versus [PMHS] are shown below:

Supplementary Table 8. The molar concentration of product **3a** in different concentration of PMHS at different time interval

Time (s)	0.25 M [PMHS]	0.35 M [PMHS]	0.45 M [PMHS]	0.55 M [PMHS]
180	0.002510	0.002460	0.003070	0.004505
360	0.005370	0.004125	0.005675	0.007040
540	0.007680	0.007565	0.007780	0.009265
720	0.009790	0.009090	0.010050	0.011615
900	0.011525	0.011665	0.012580	0.013540

Supplementary Table 9. The k_{in} value of product **3a** in different concentration of PMHS

[PMHS] M	k_{in}/Ms^{-1}
0.25 M	1.25×10^{-5}
0.35 M	1.30×10^{-5}
0.45 M	1.30×10^{-5}
0.55 M	1.26×10^{-5}

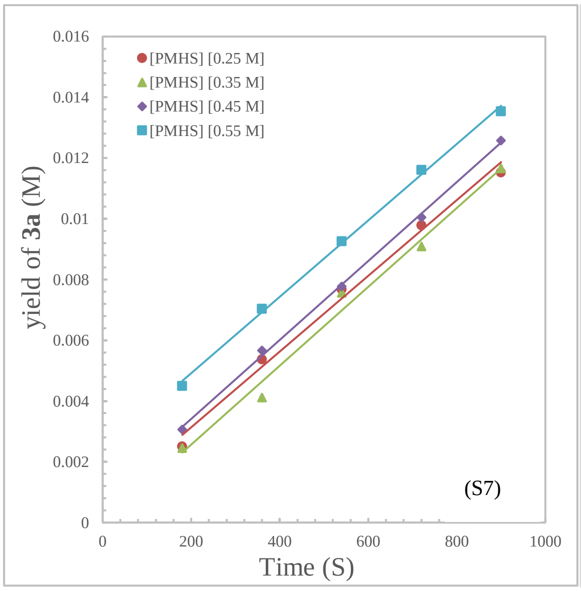

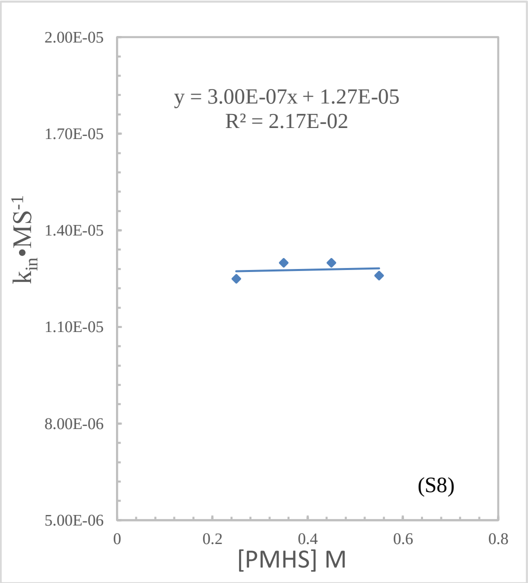

Supplementary Figure 17. Plot of the rise of product a from the reaction of alkyne (0.20 M), allylic bromide (0.1 M), and (**L3**-H)•CoBr (0.005 M) with 0.25 M, 0.35 M, 0.45 M, and 0.55 M of PMHS in different time interval at 50 ºC. **Supplementary Figure 18**. Plot of k_{in} versus [PMHS] from the reaction of alkyne (0.20 M), allylic bromide (0.10 M), (**L3**-H)•CoBr (0.005 M) with 0.25 M, 0.35 M, 0.45 M, and 0.55 M of PMHS.

IX. Unsuccessful substrates

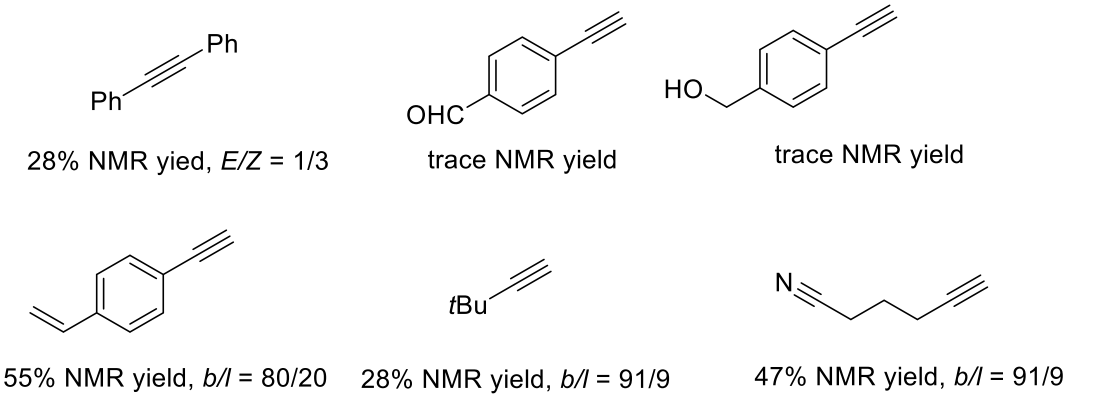

Supplementary Figure 19. Unsuccessful substrates

X. NMR Spectra

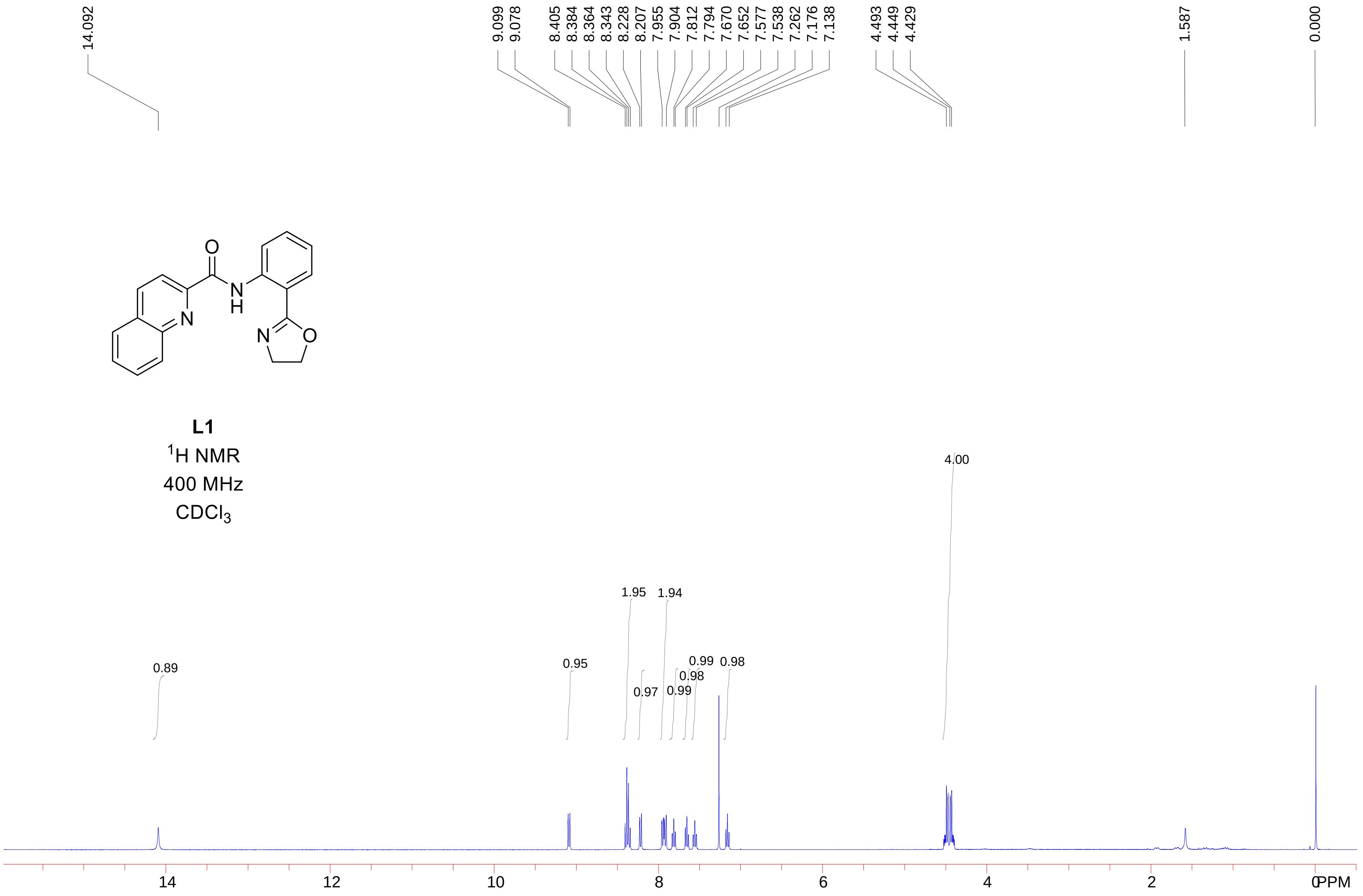

Supplementary Figure 20. ^{1}H NMR spectrum of **L1**

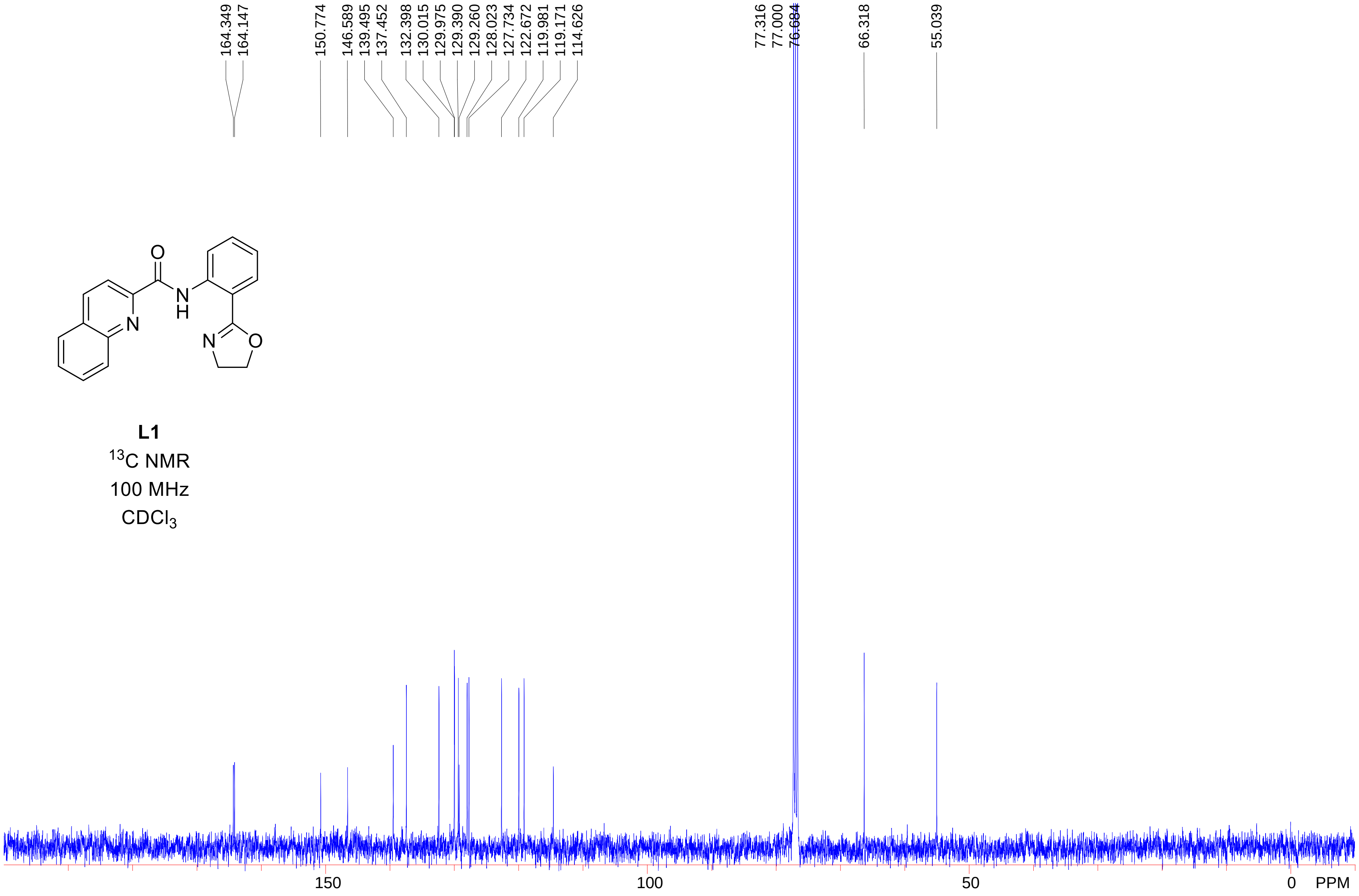

Supplementary Figure 21. ^{13}C NMR spectrum of **L1**

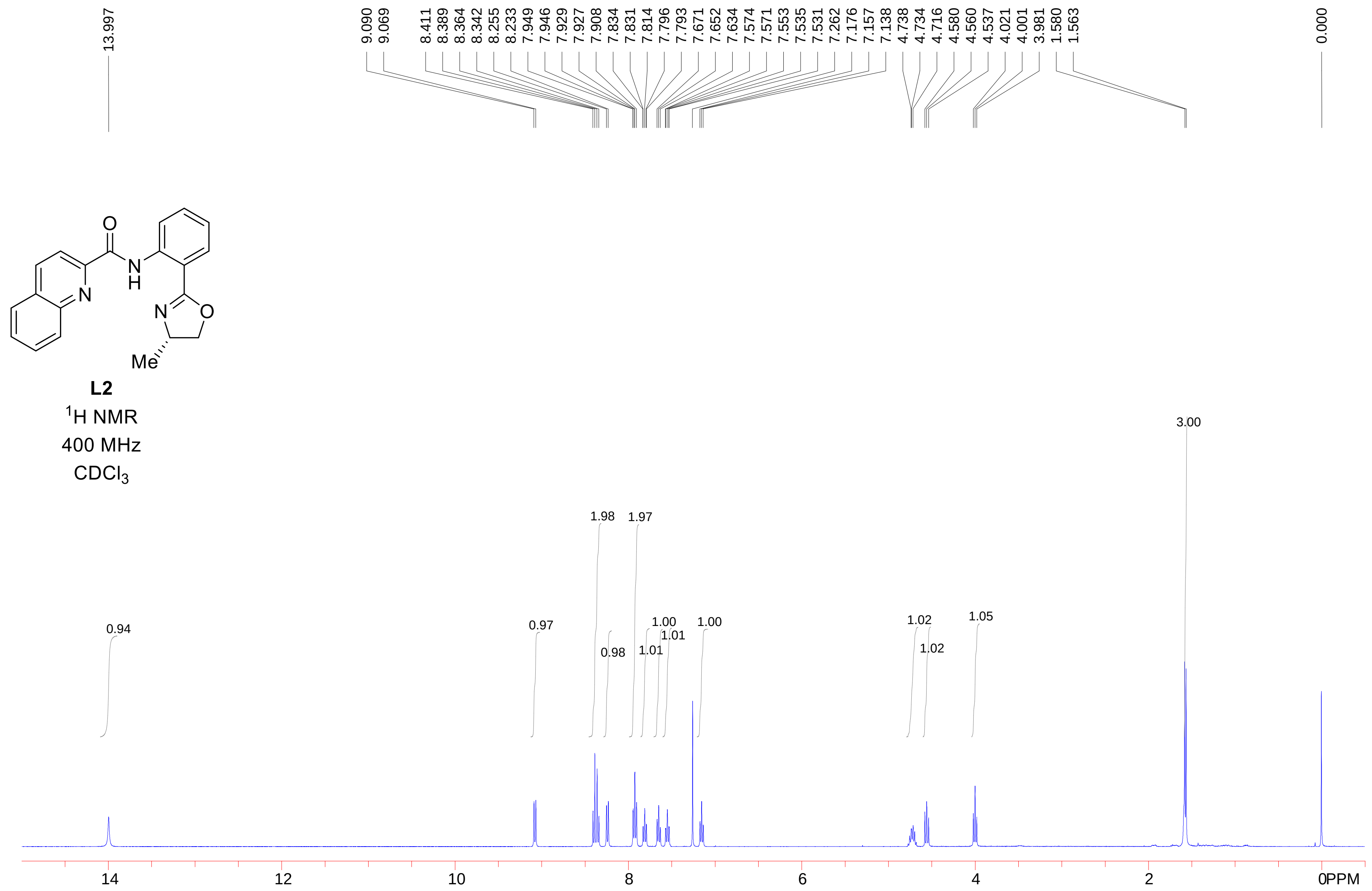

Supplementary Figure 22. ^{1}H NMR spectrum of **L2**

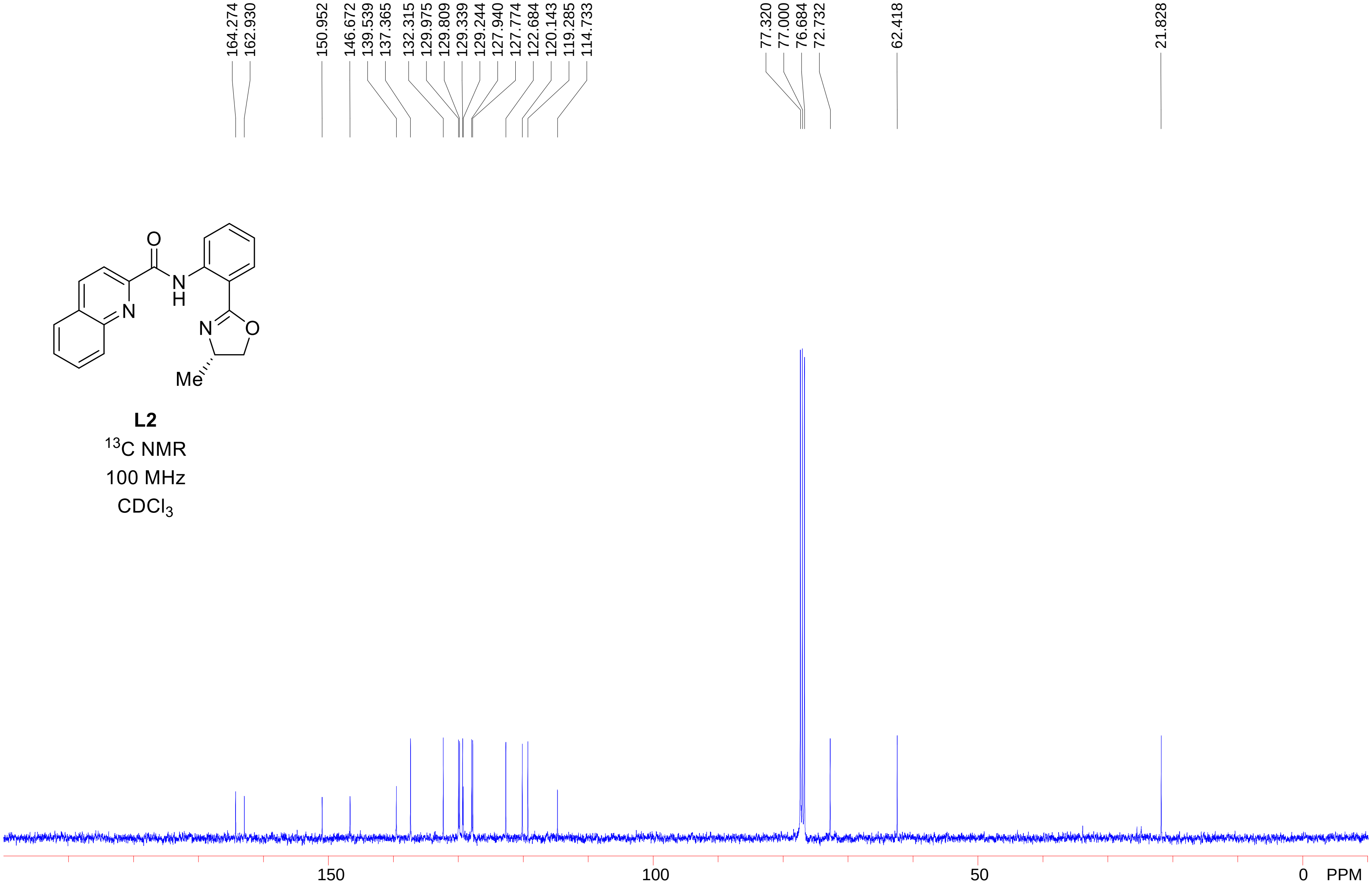

Supplementary Figure 23. ^{13}C NMR spectrum of **L2**

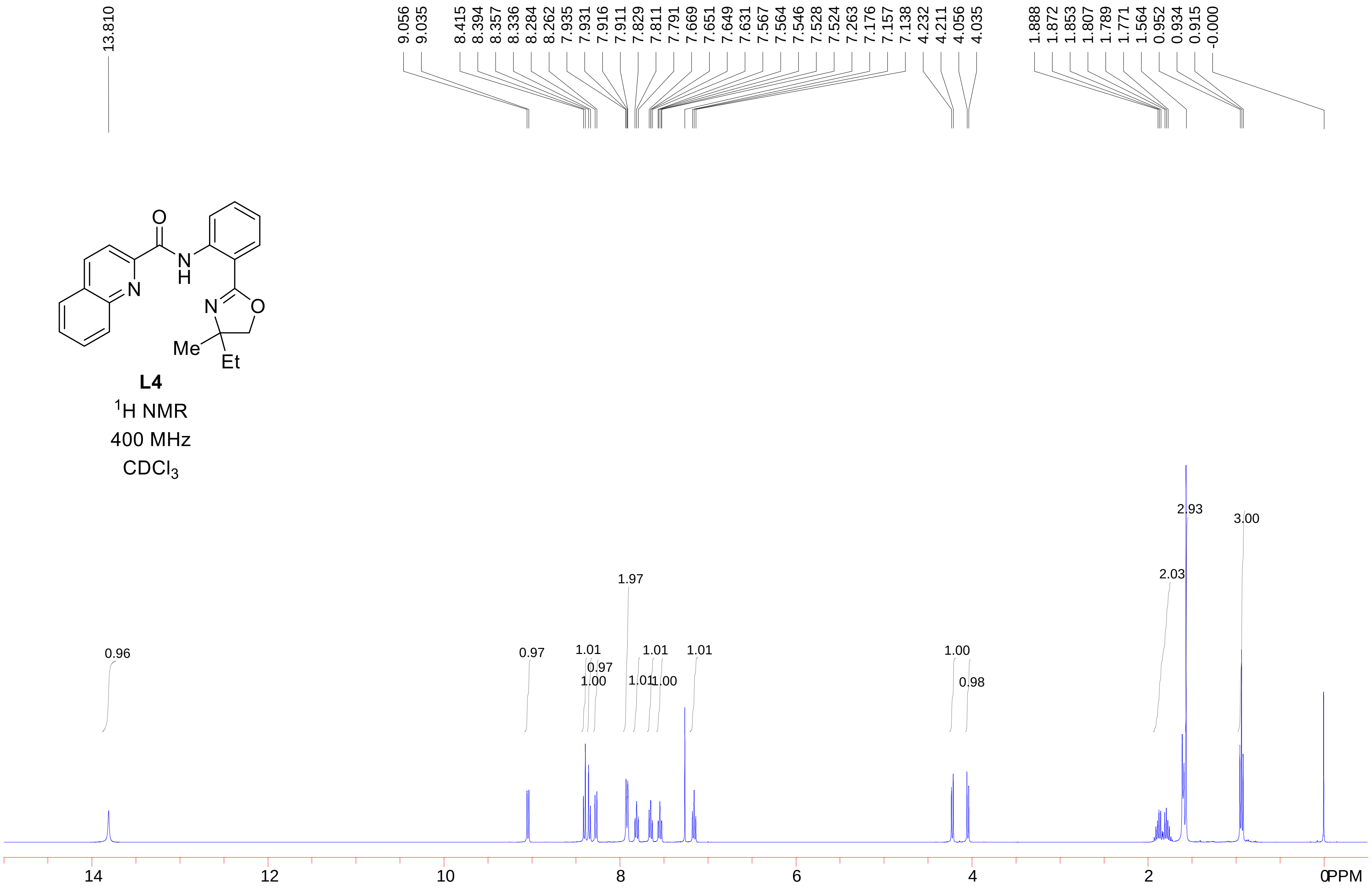

Supplementary Figure 24. ^{1}H NMR spectrum of **L4**

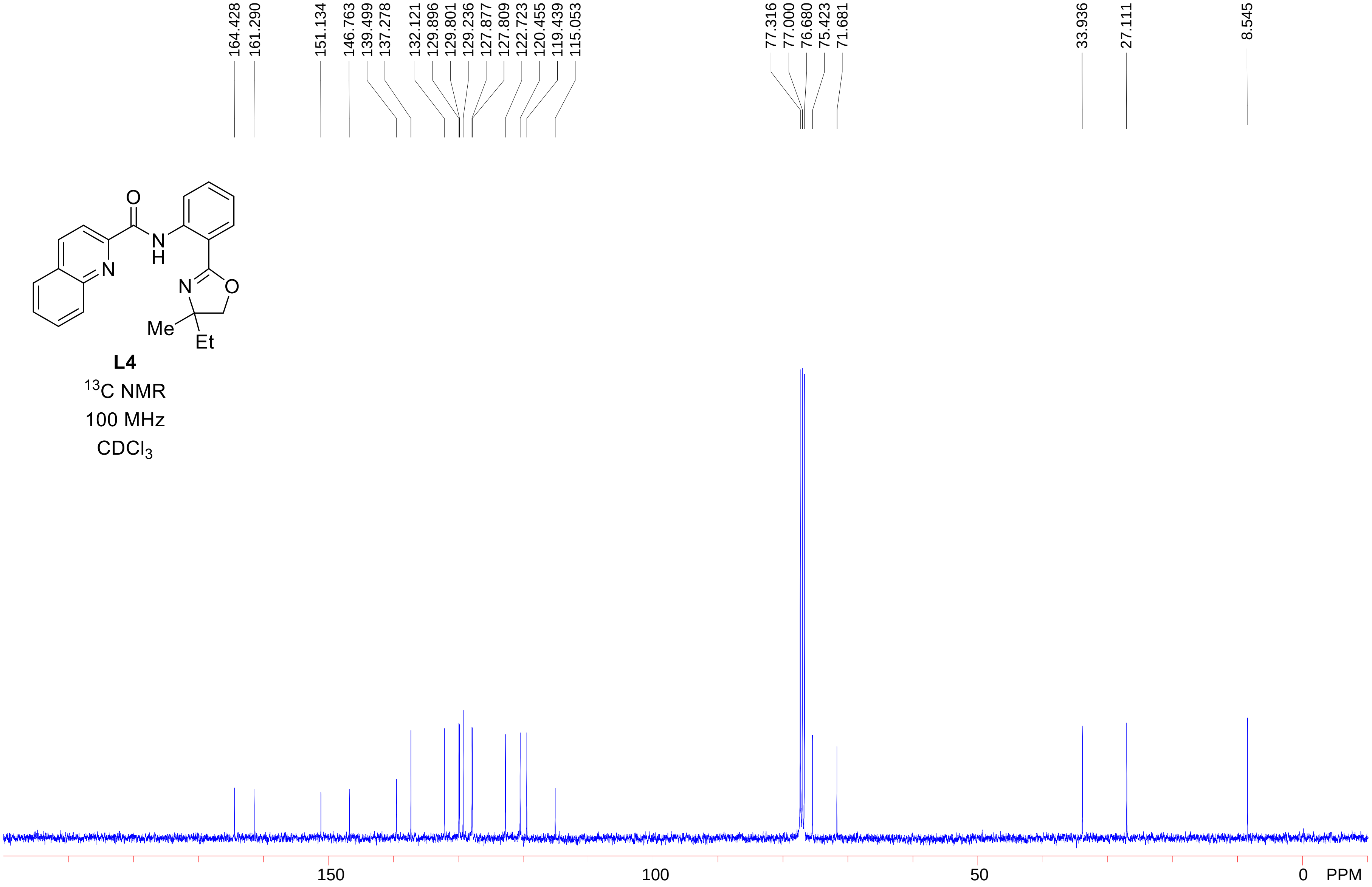

Supplementary Figure 25. ^{13}C NMR spectrum of **L4**

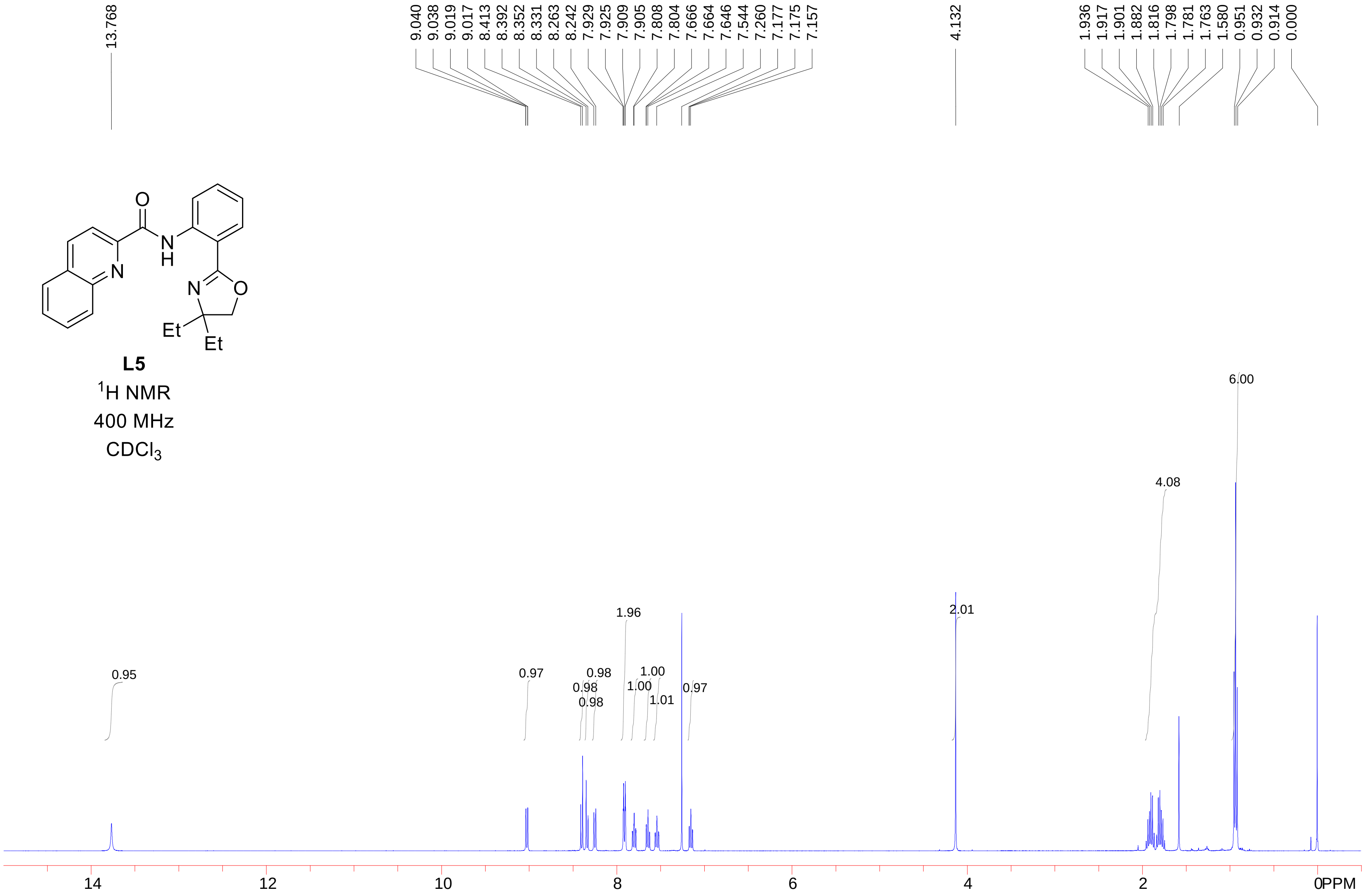

Supplementary Figure 26. ^{1}H NMR spectrum of **L5**

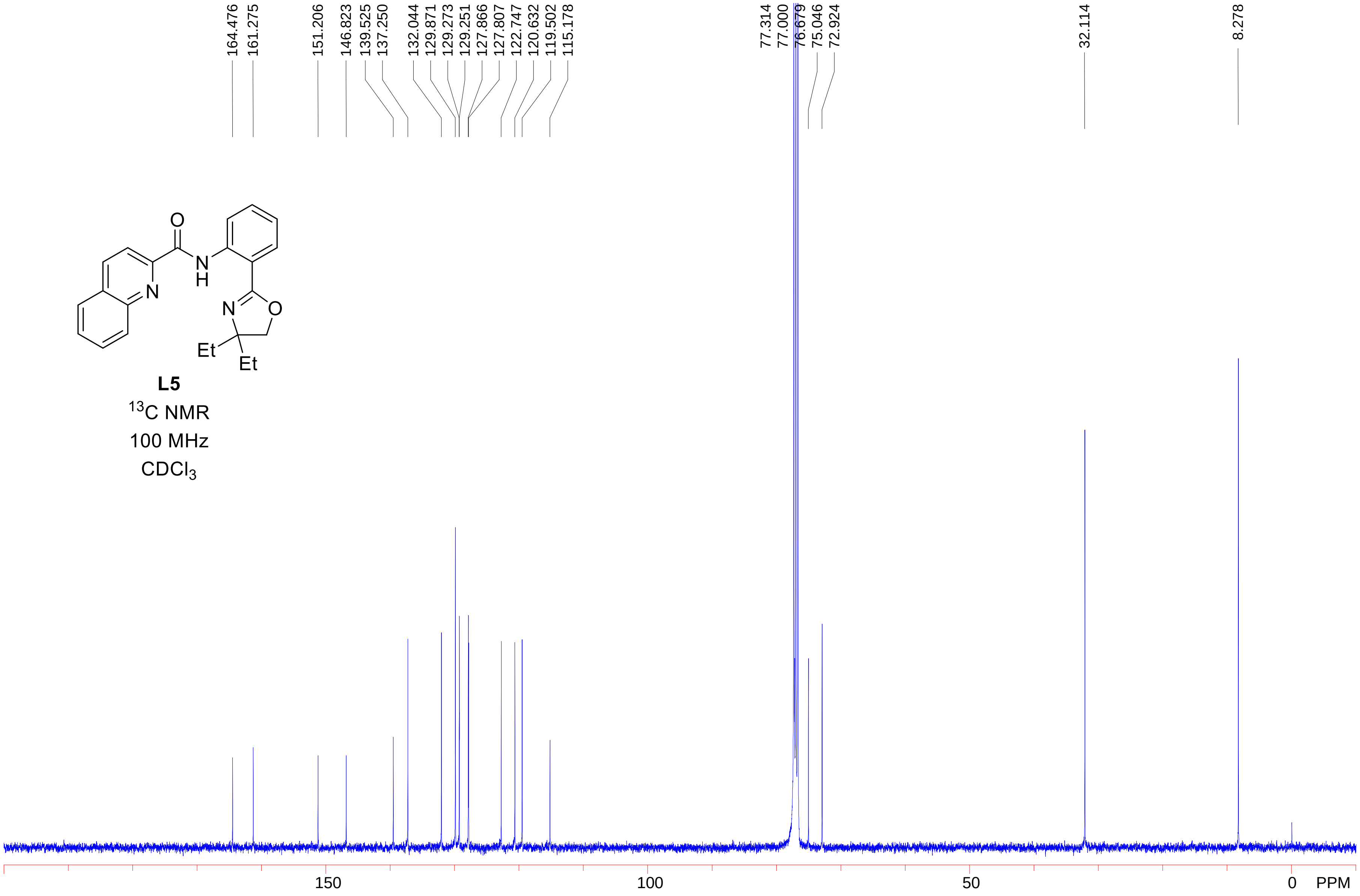

Supplementary Figure 27. ^{13}C NMR spectrum of **L5**

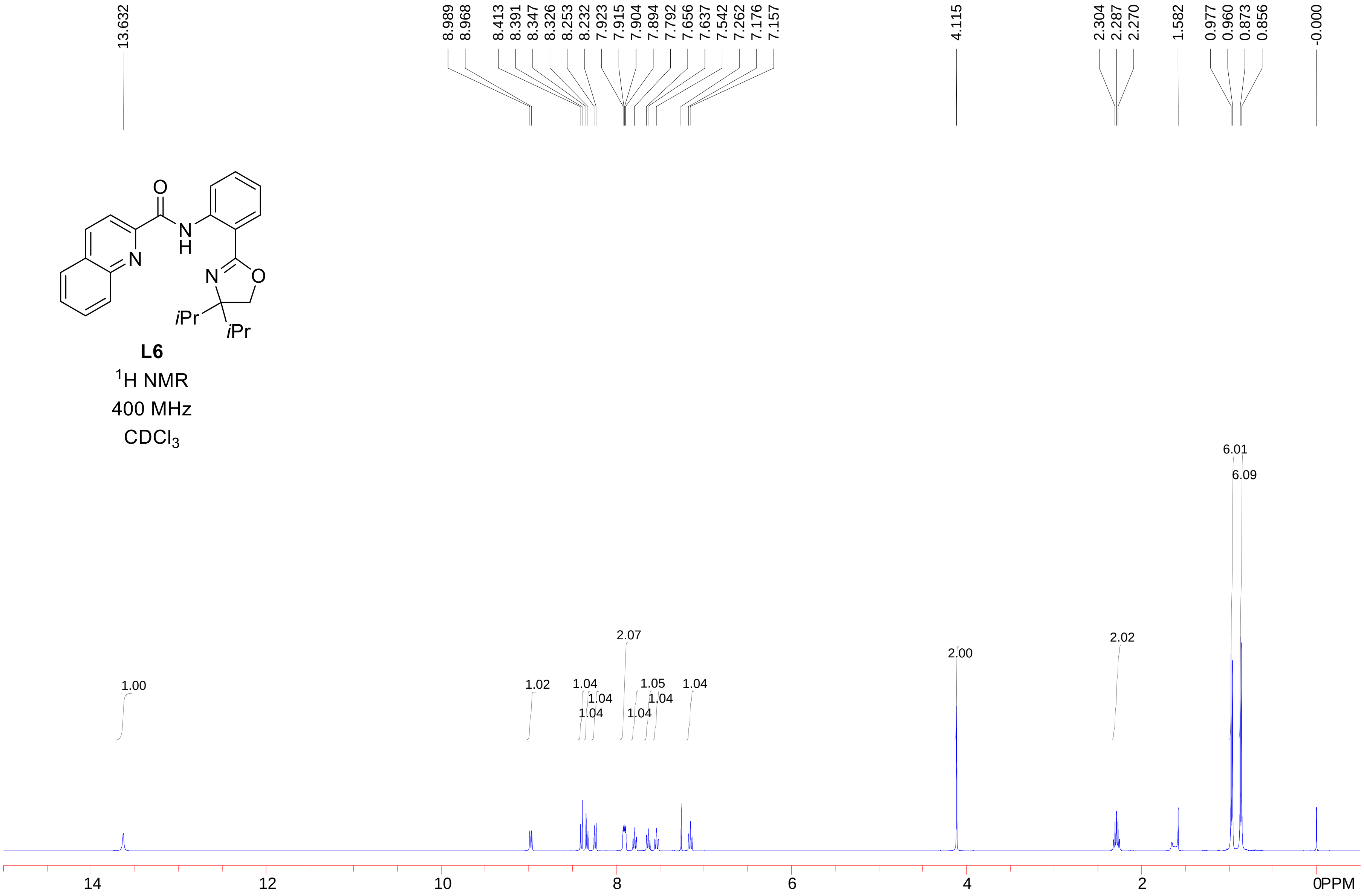

Supplementary Figure 28. ^{1}H NMR spectrum of **L6**

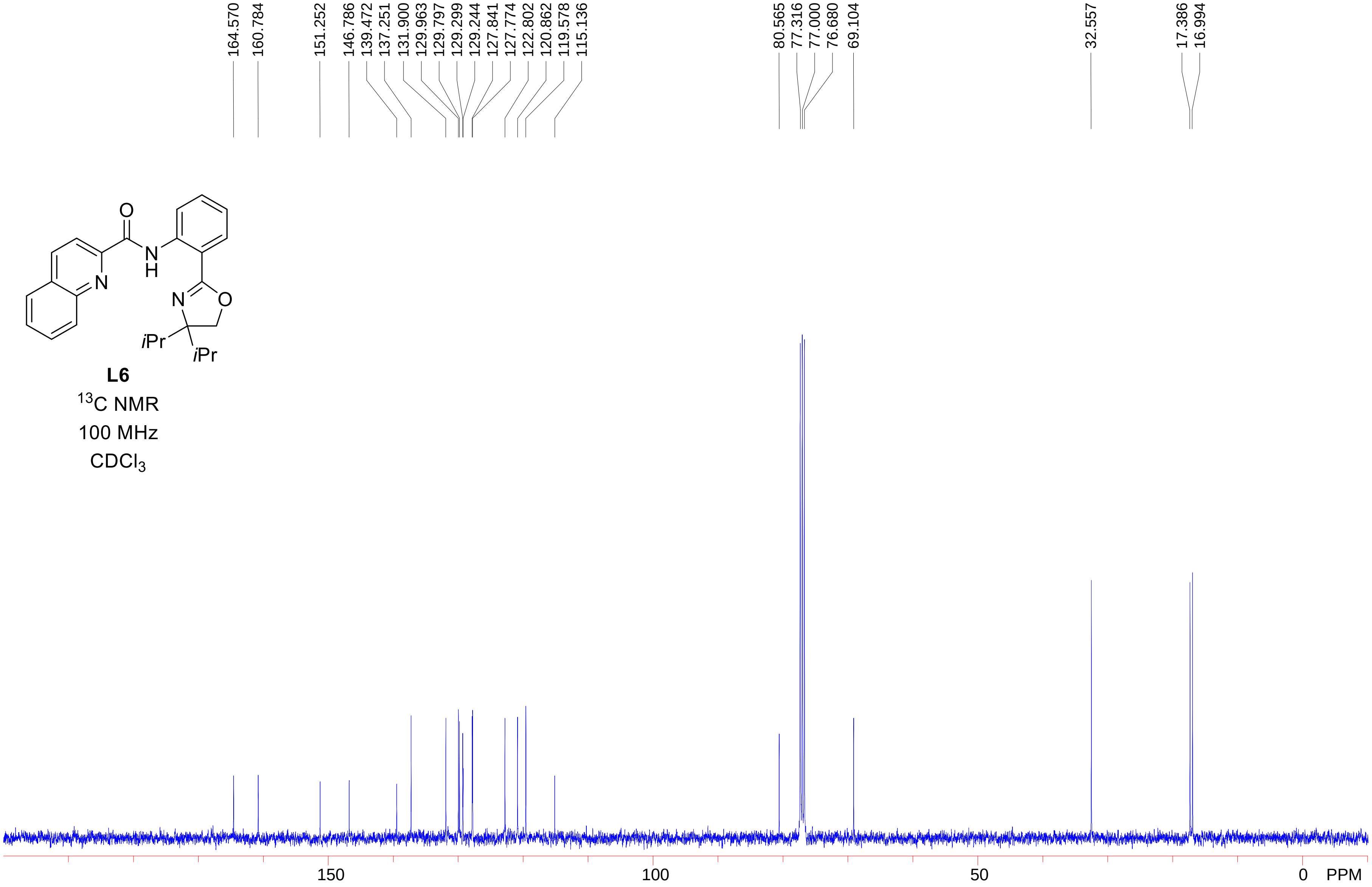

Supplementary Figure 29. ^{13}C NMR spectrum of **L6**

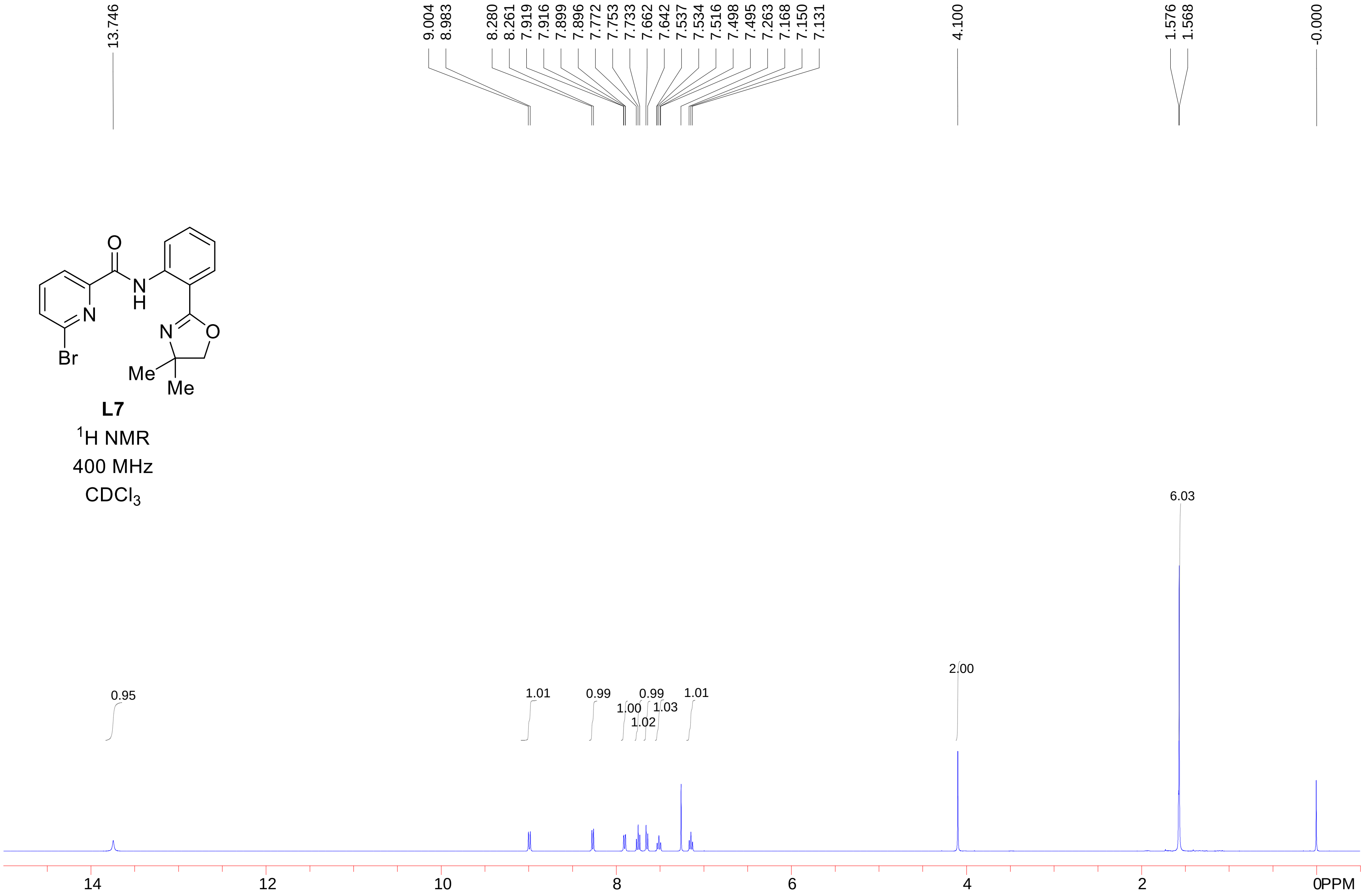

Supplementary Figure 30. ^{1}H NMR spectrum of **L7**

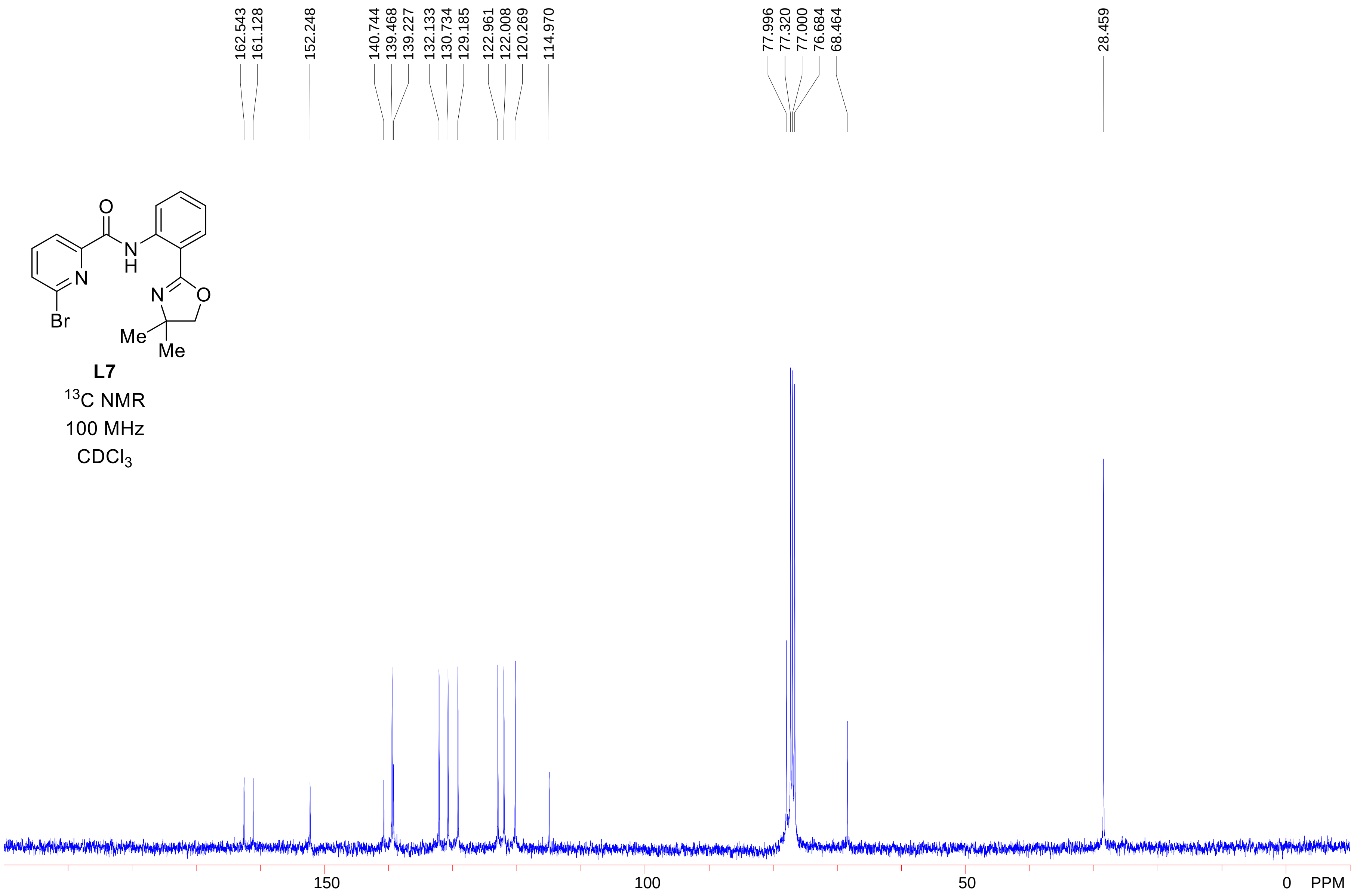

Supplementary Figure 31. ^{13}C NMR spectrum of **L7**

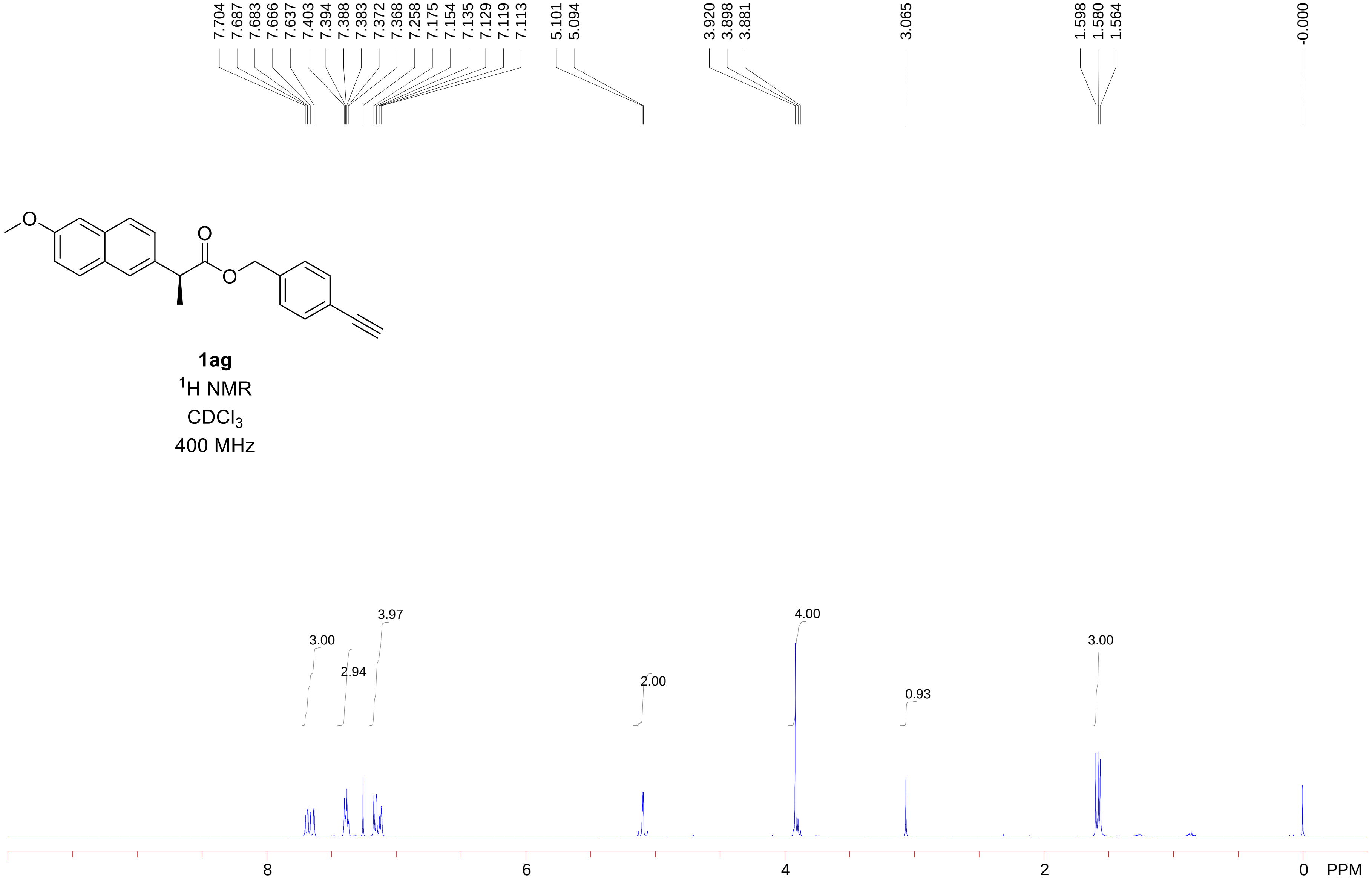

Supplementary Figure 32. ^{1}H NMR spectrum of **1ag**

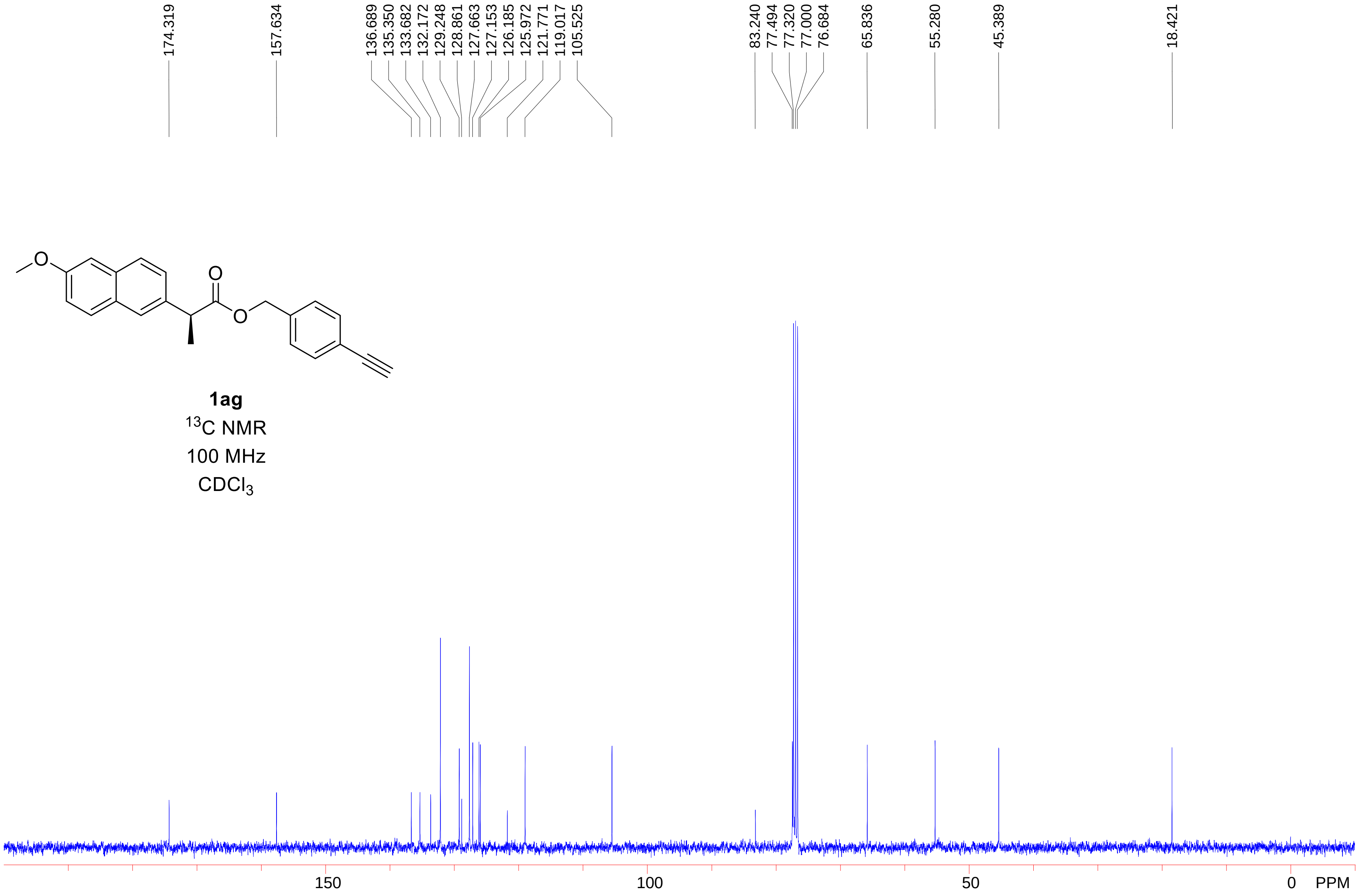

Supplementary Figure 33. ^{13}C NMR spectrum of **1ag**

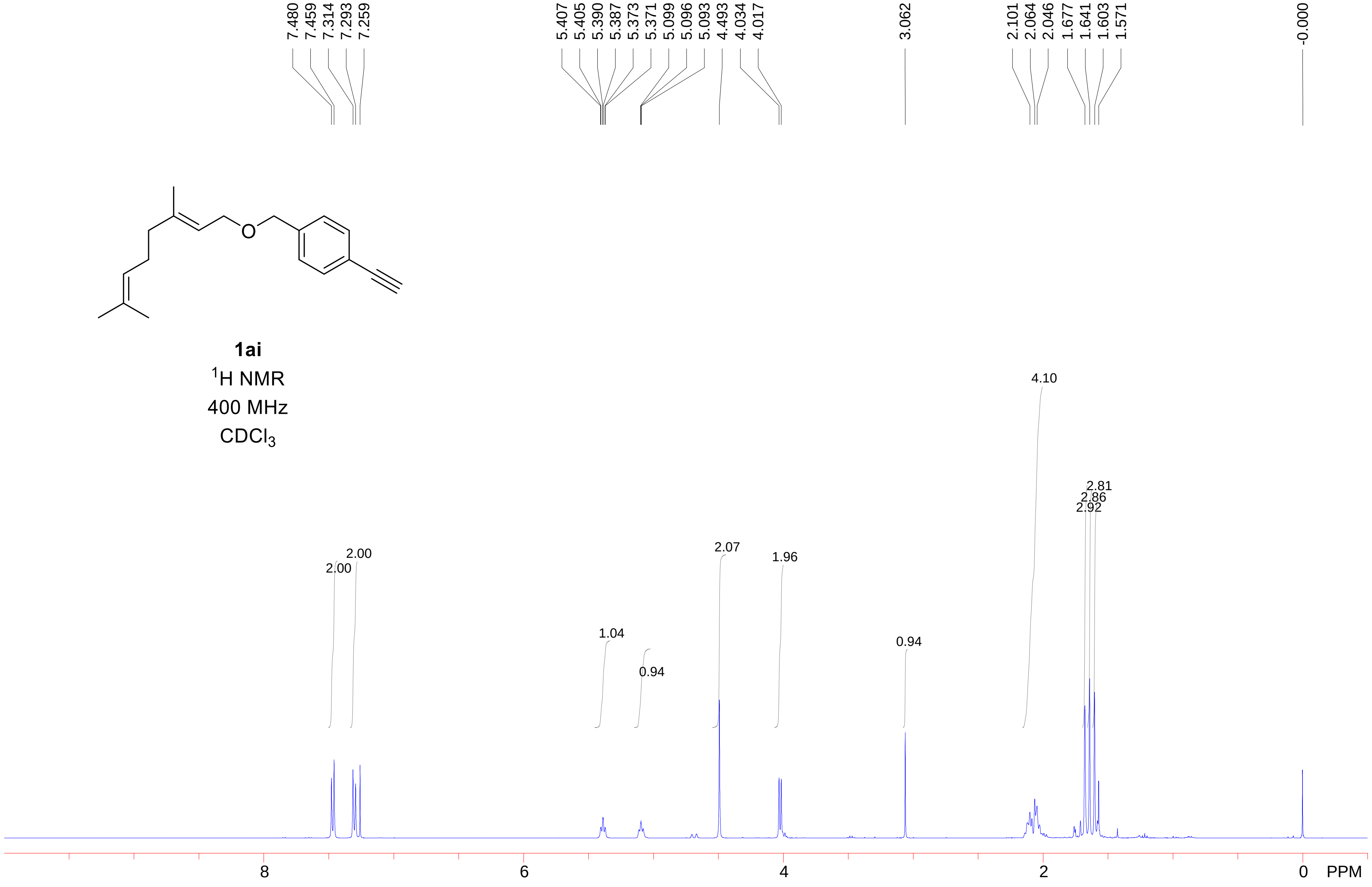

Supplementary Figure 34. ^{1}H NMR spectrum of **1ai**

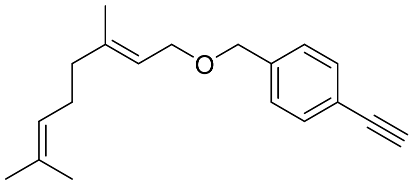

1ai

^{13}C NMR

100 MHz

$CDCl_3$

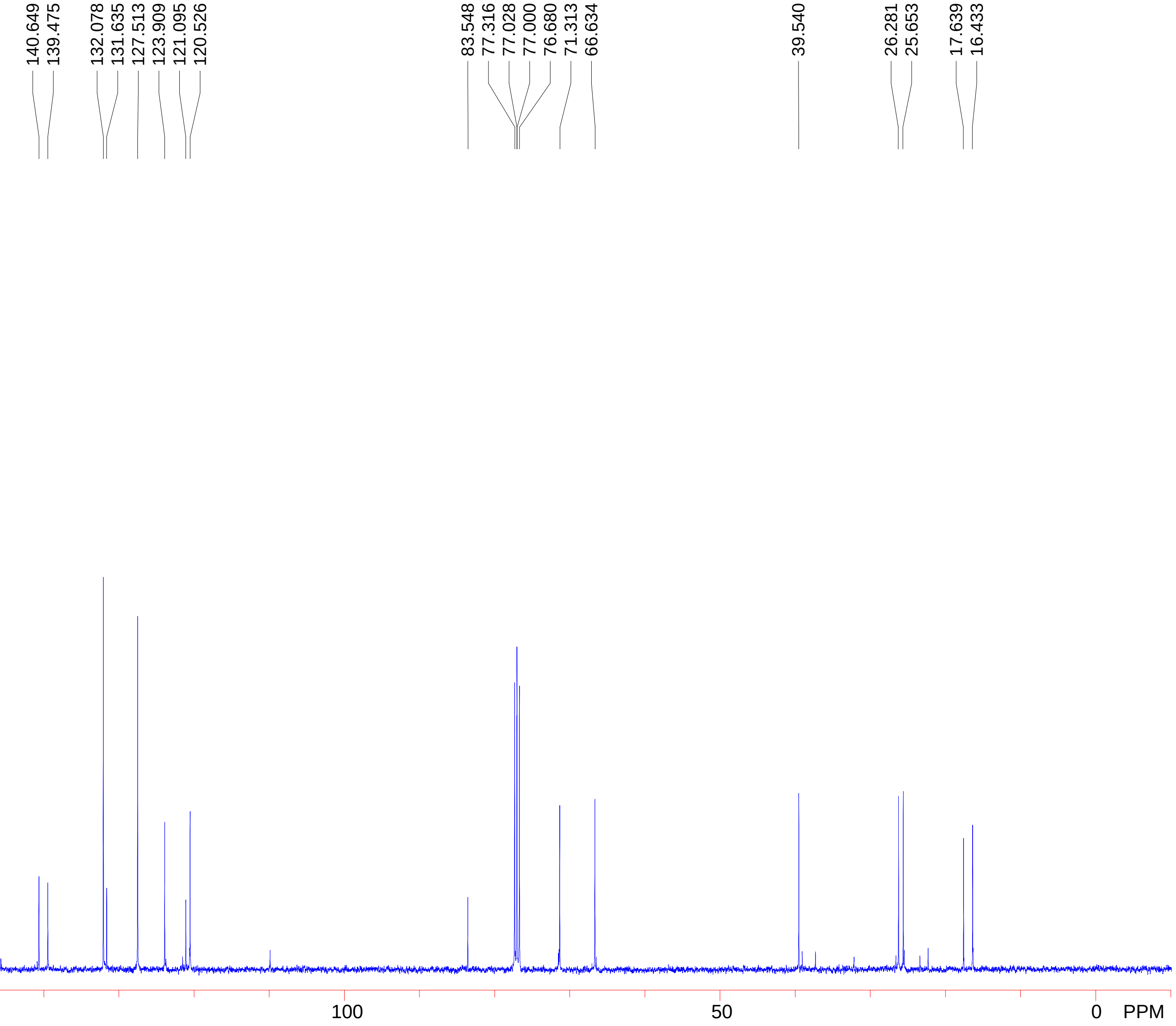

Supplementary Figure 35. ^{13}C NMR spectrum of **1ai**

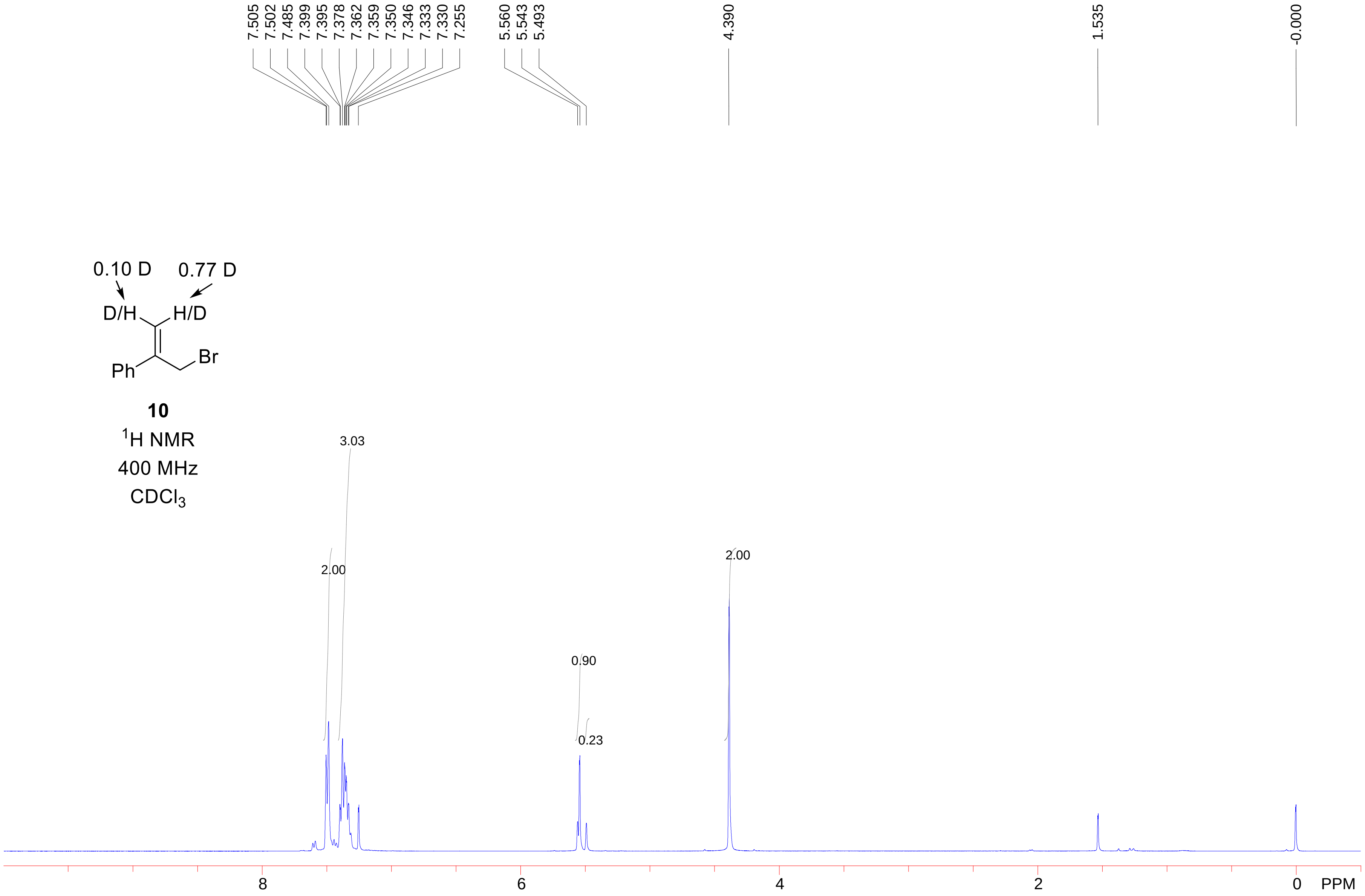

Supplementary Figure 36. ^{1}H NMR spectrum of **10**

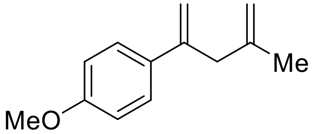

3a

^{1}H NMR

400 MHz

$CDCl_3$

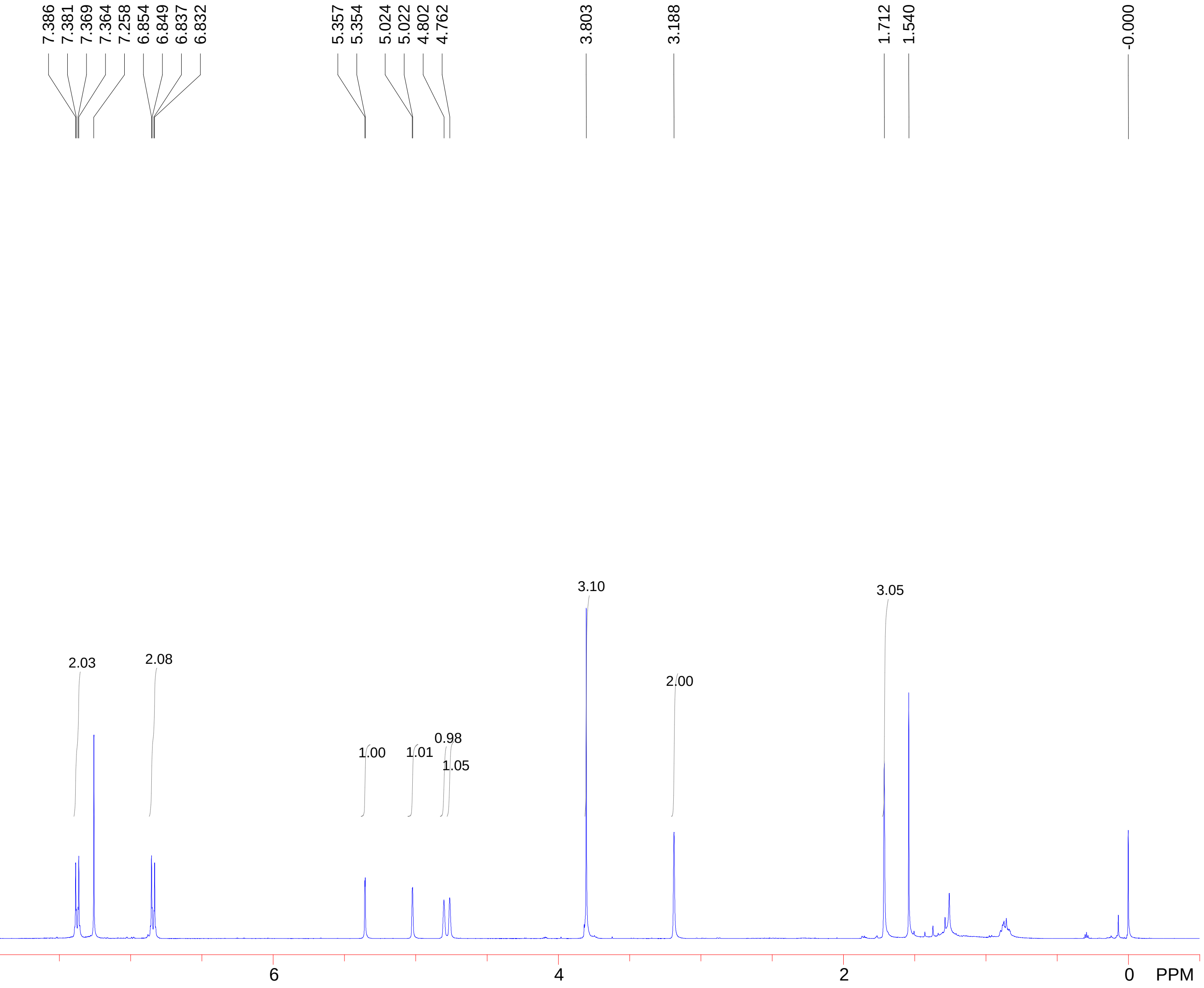

Supplementary Figure 37. ^{1}H NMR spectrum of **3a**

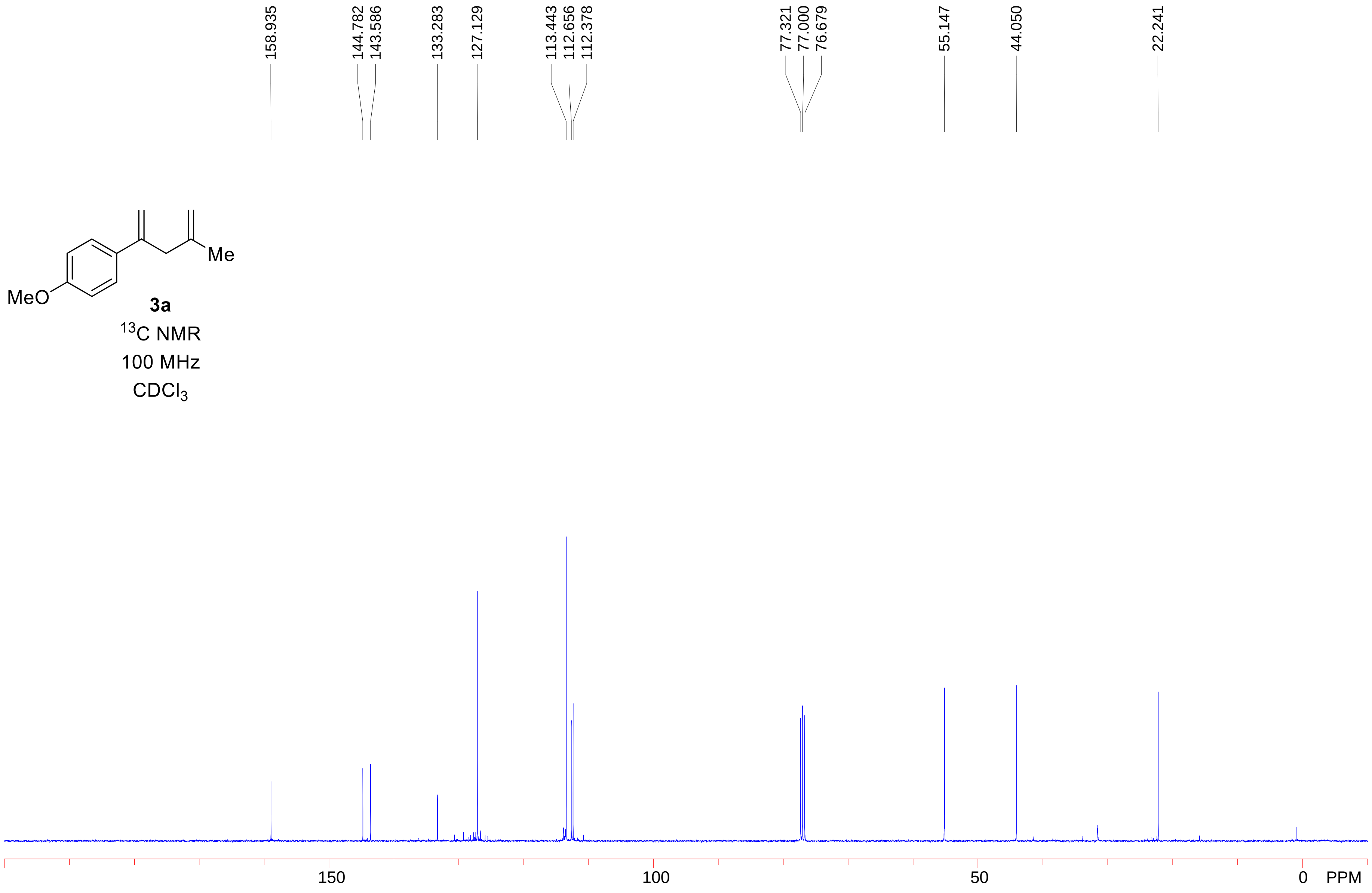

Supplementary Figure 38. ^{13}C NMR spectrum of **3a**

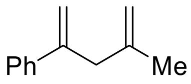

3b
^{1}H NMR
400 MHz
$CDCl_3$

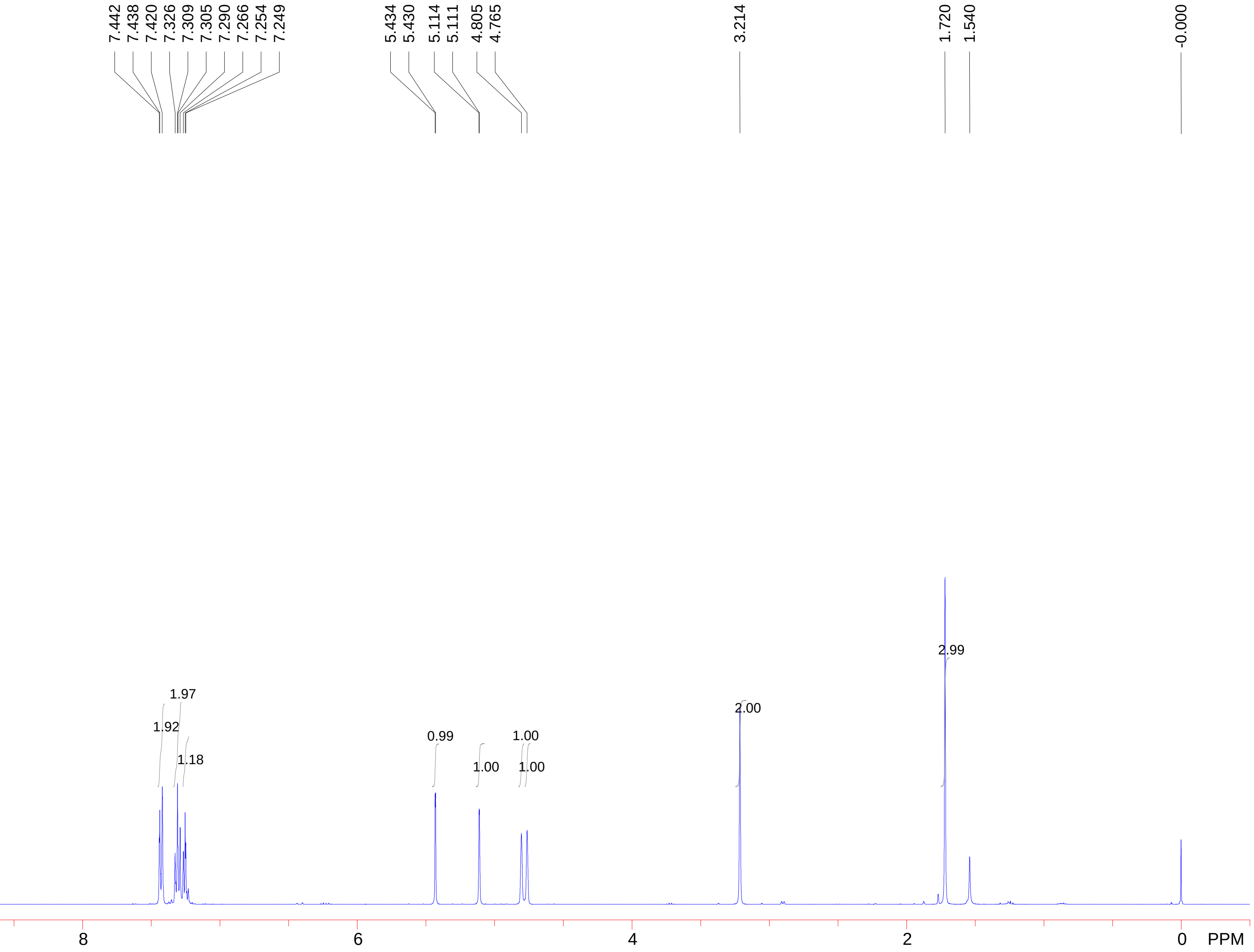

Supplementary Figure 39. ^{1}H NMR spectrum of **3b**

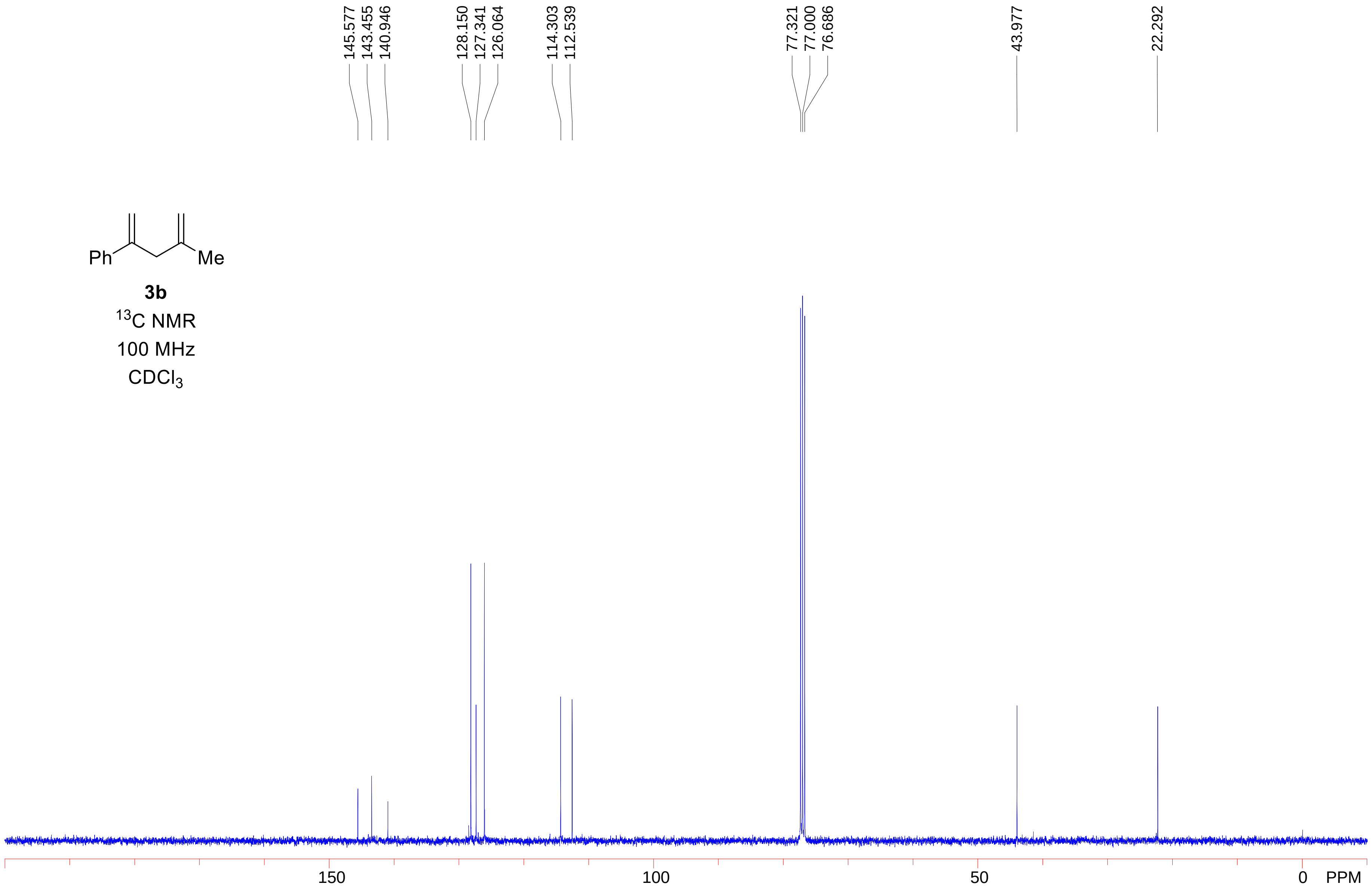

Supplementary Figure 40. ^{13}C NMR spectrum of **3b**

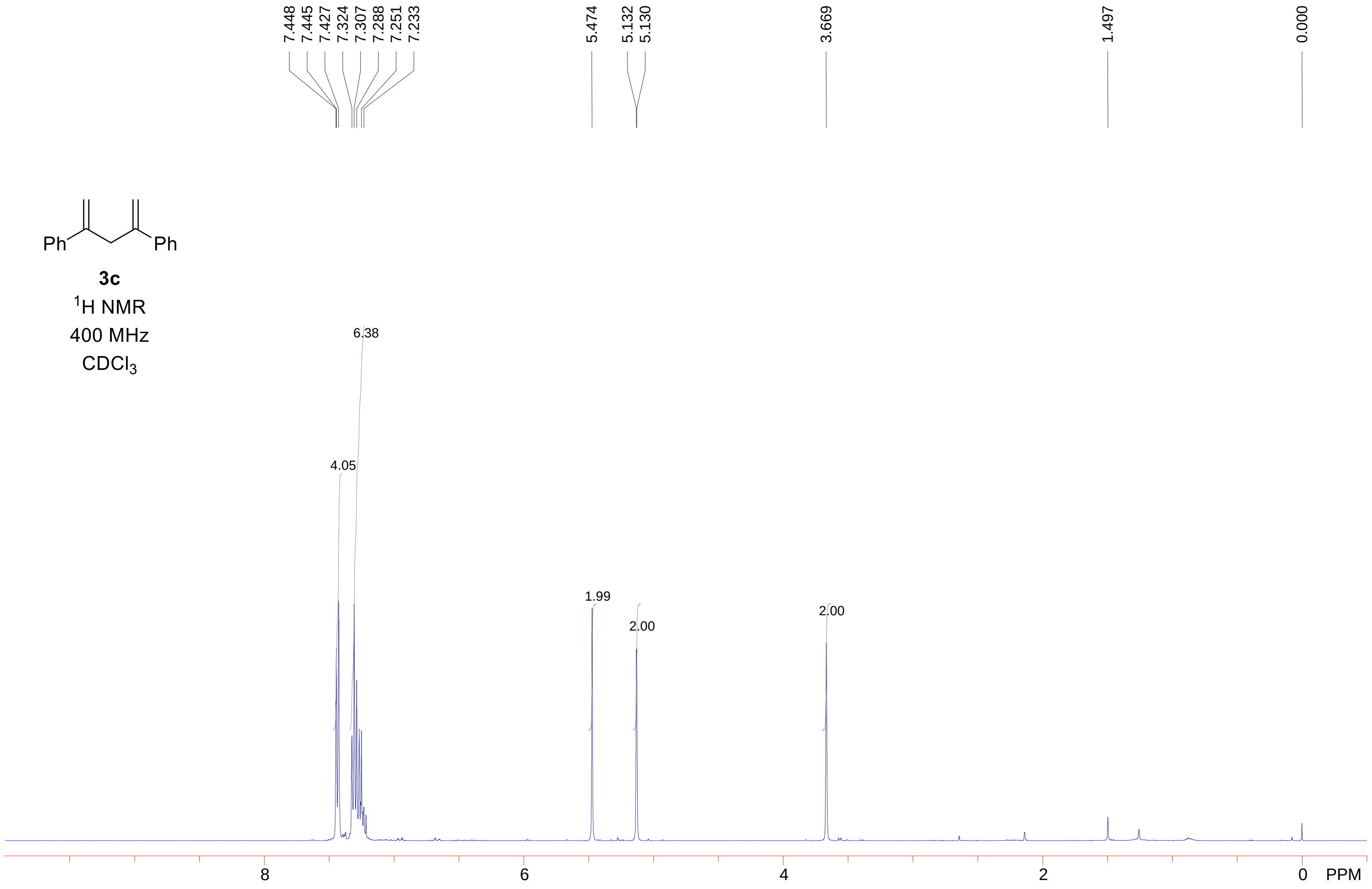

Supplementary Figure 41. ^{1}H NMR spectrum of **3c**

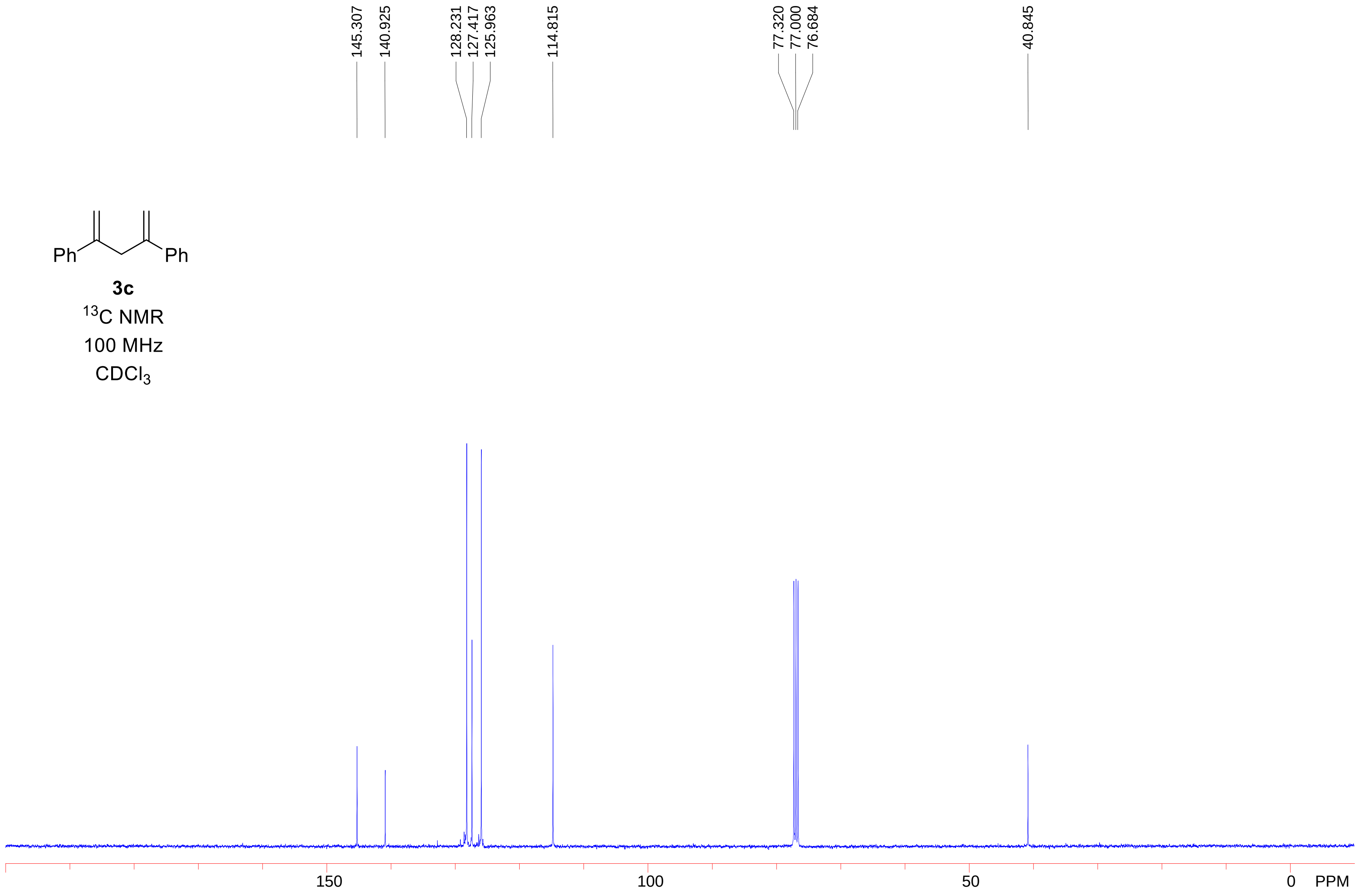

Supplementary Figure 42. ^{13}C NMR spectrum of **3c**

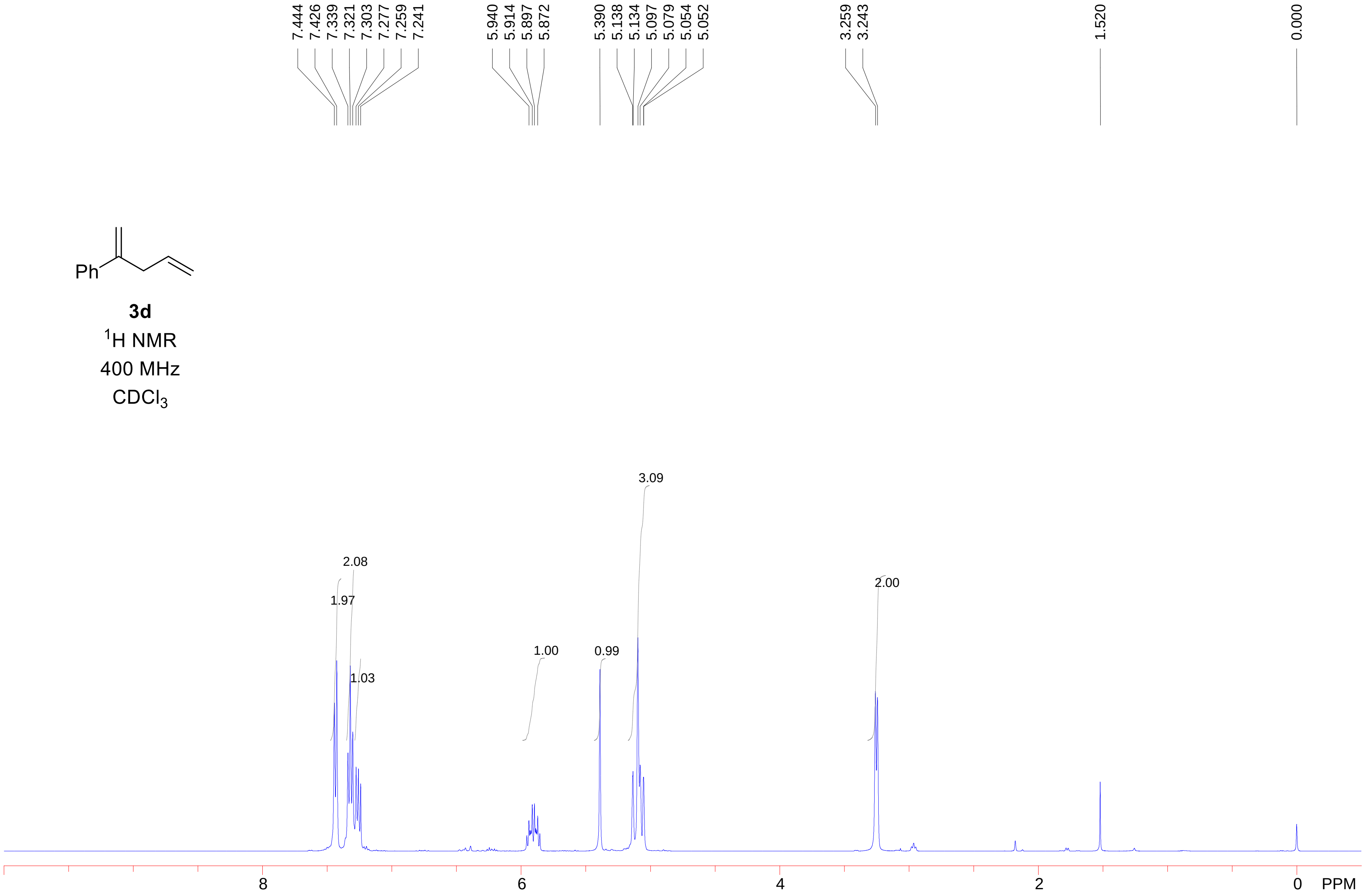

Supplementary Figure 43. ^{1}H NMR spectrum of **3d**

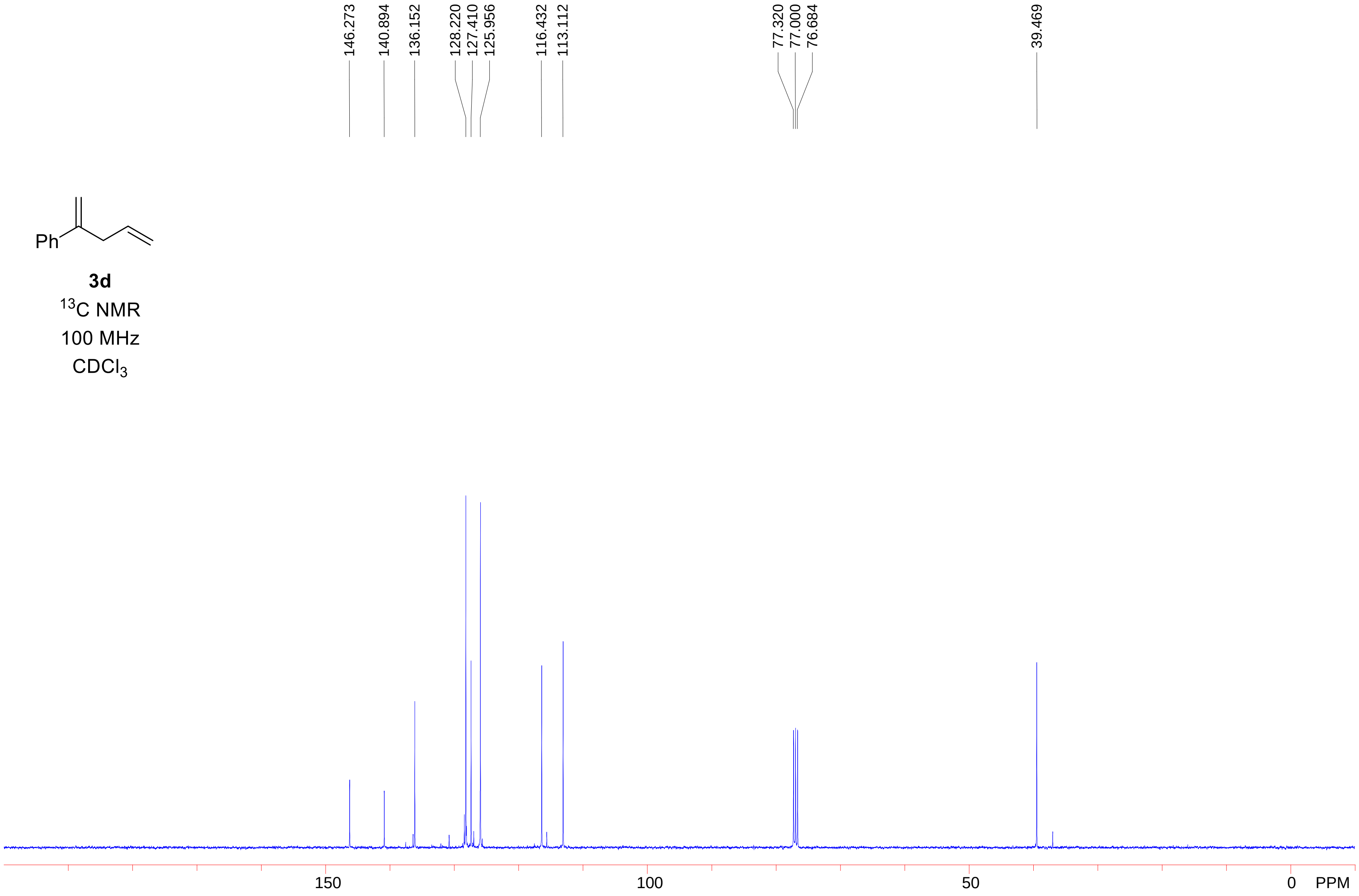

Supplementary Figure 44. ^{13}C NMR spectrum of **3d**

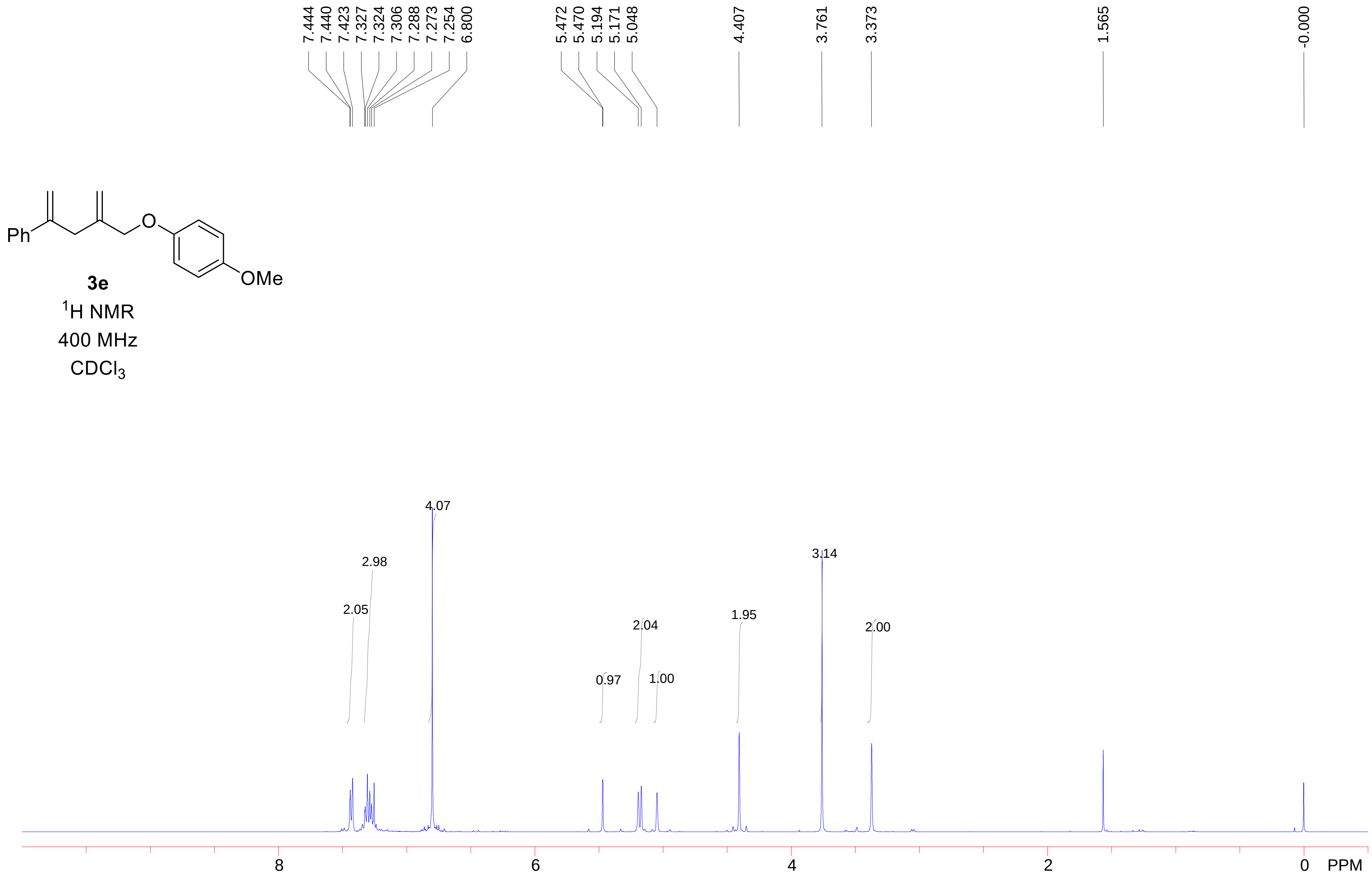

Supplementary Figure 45. ^{1}H NMR spectrum of **3e**

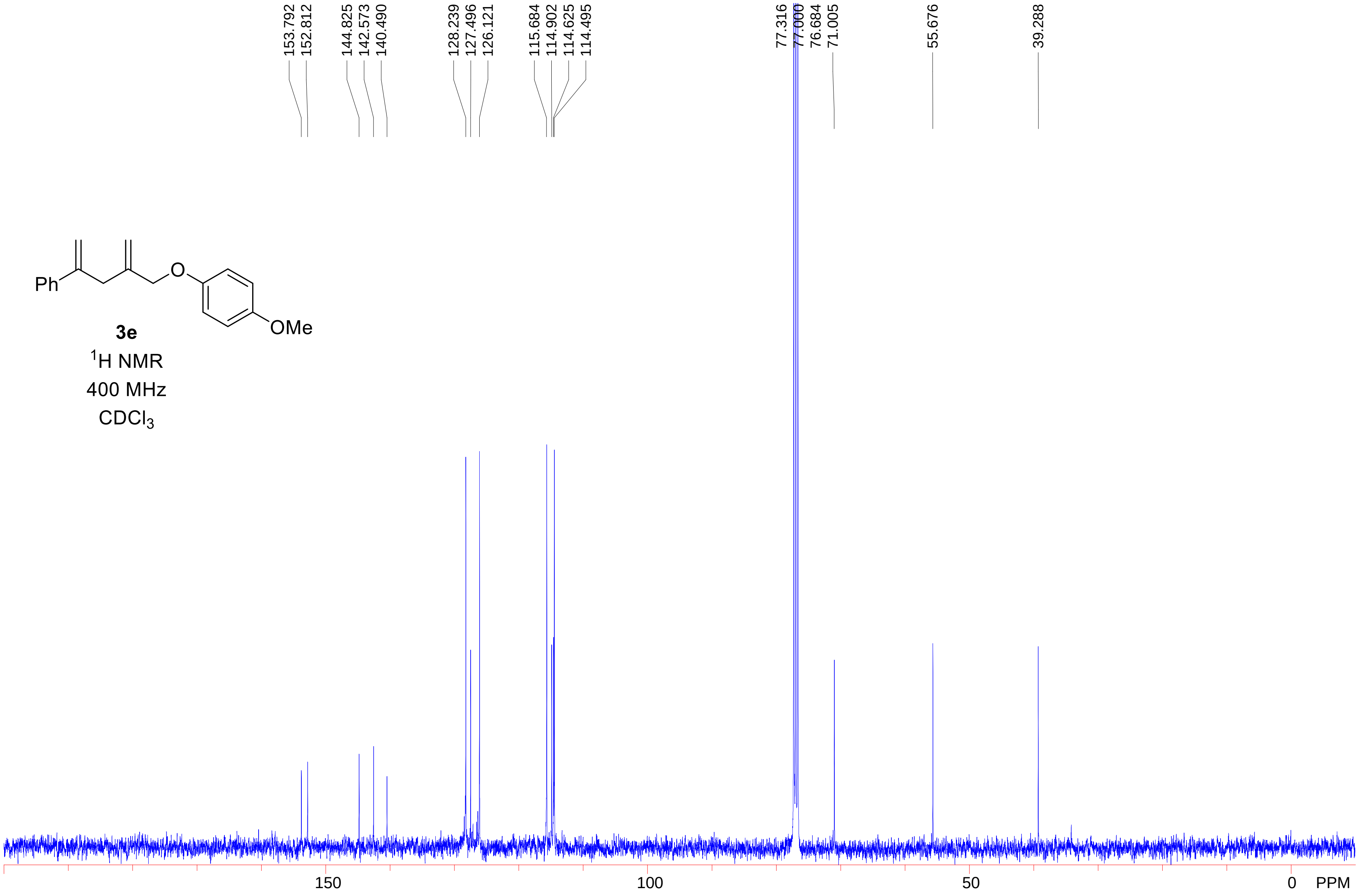

Supplementary Figure 46. ^{13}C NMR spectrum of **3e**

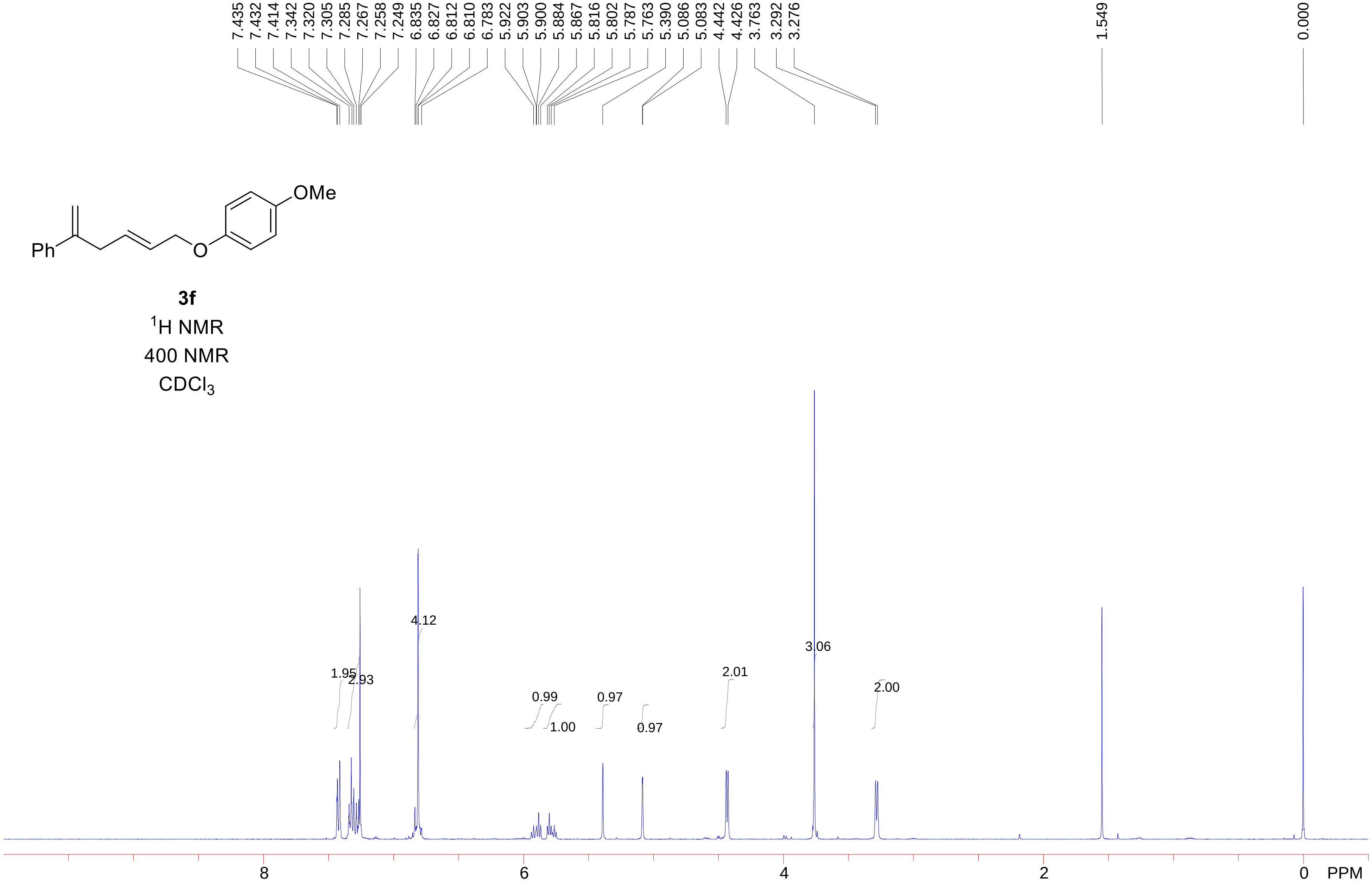

Supplementary Figure 47. ^{1}H NMR spectrum of **3f**

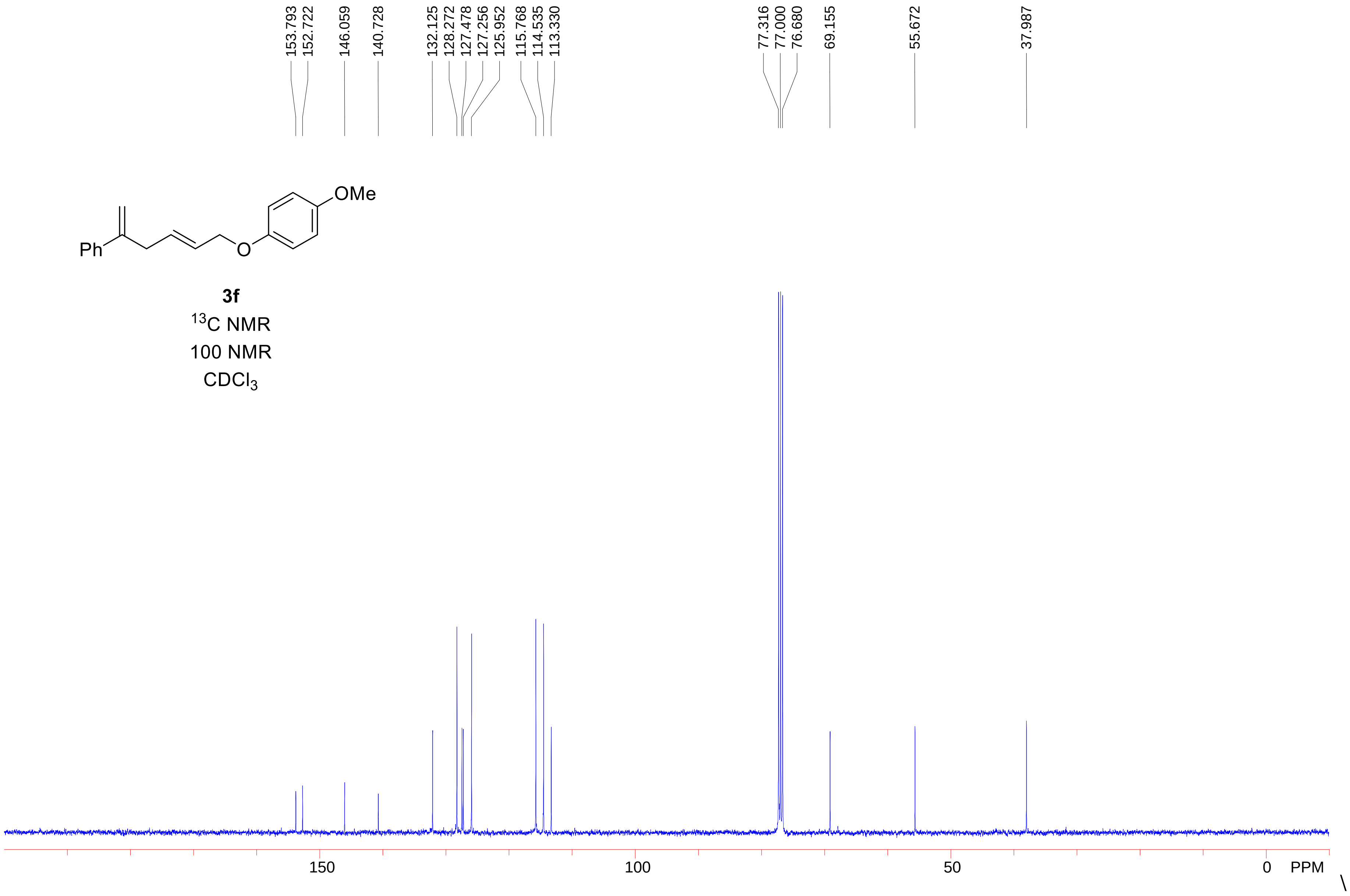

Supplementary Figure 48. ^{13}C NMR spectrum of **3f**

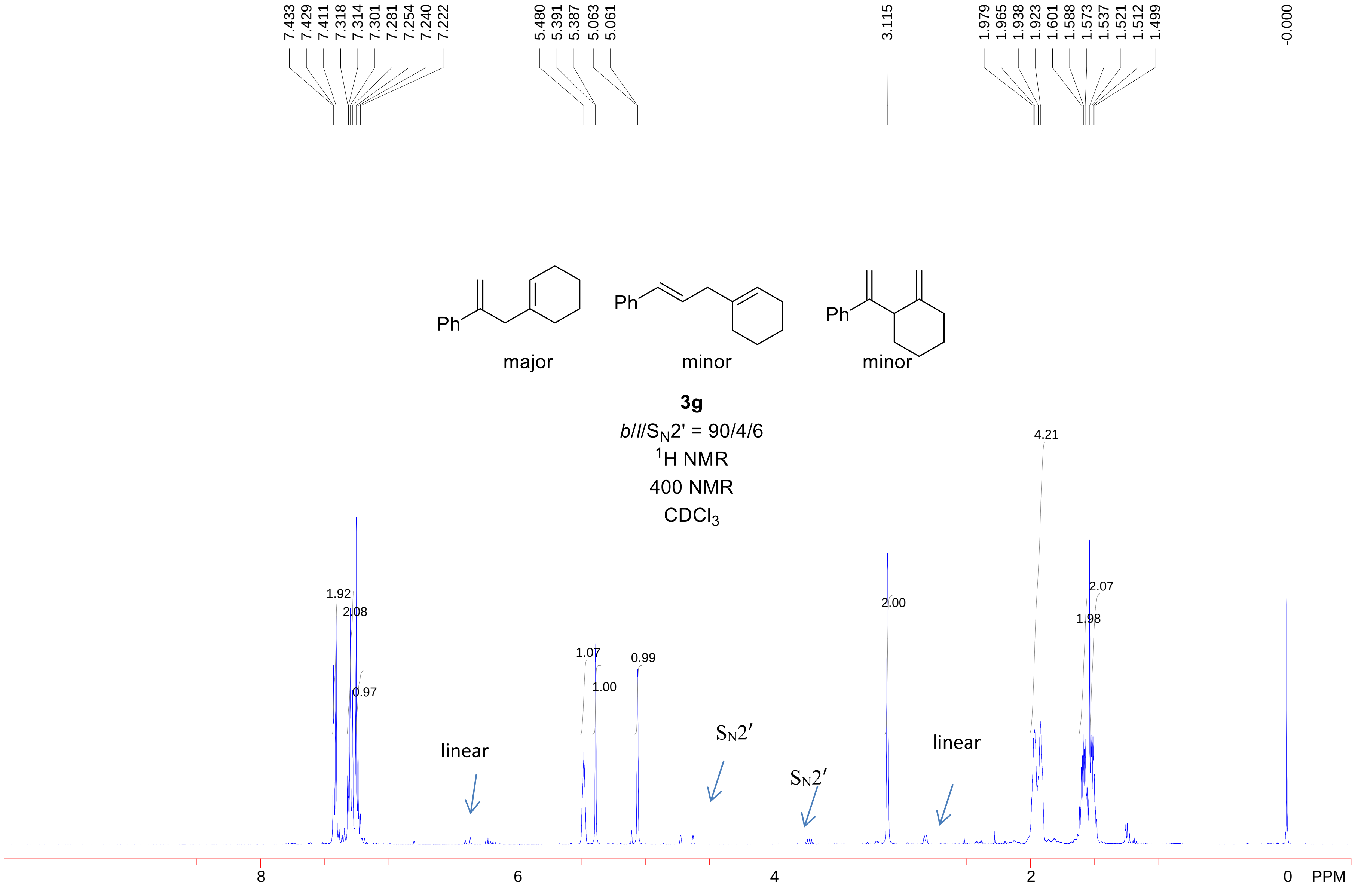

Supplementary Figure 49. ^{1}H NMR spectrum of **3g**

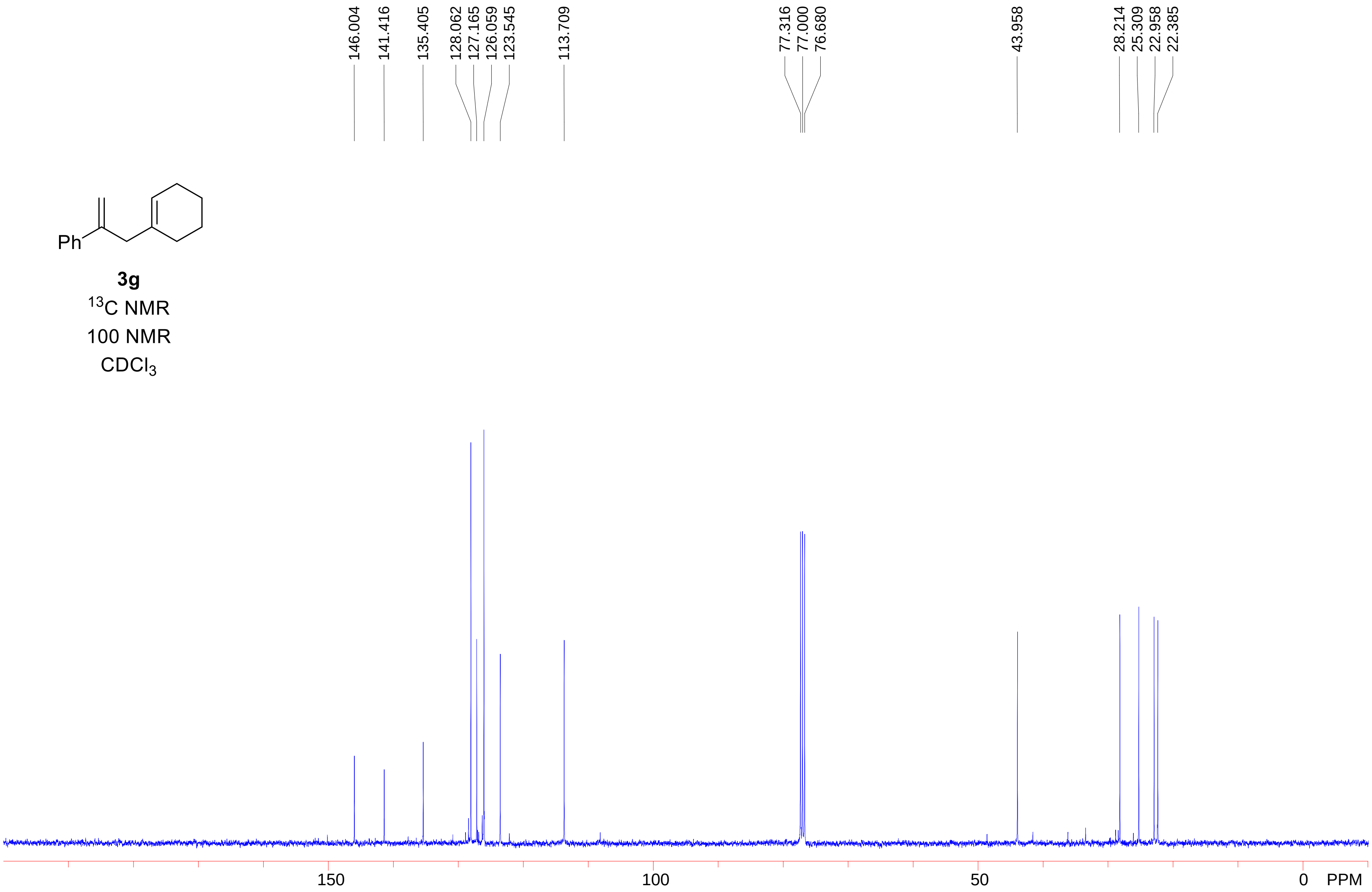

Supplementary Figure 50. ^{13}C NMR spectrum of **3g**

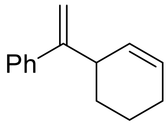

3h
^{1}H NMR
400 NMR
$CDCl_3$

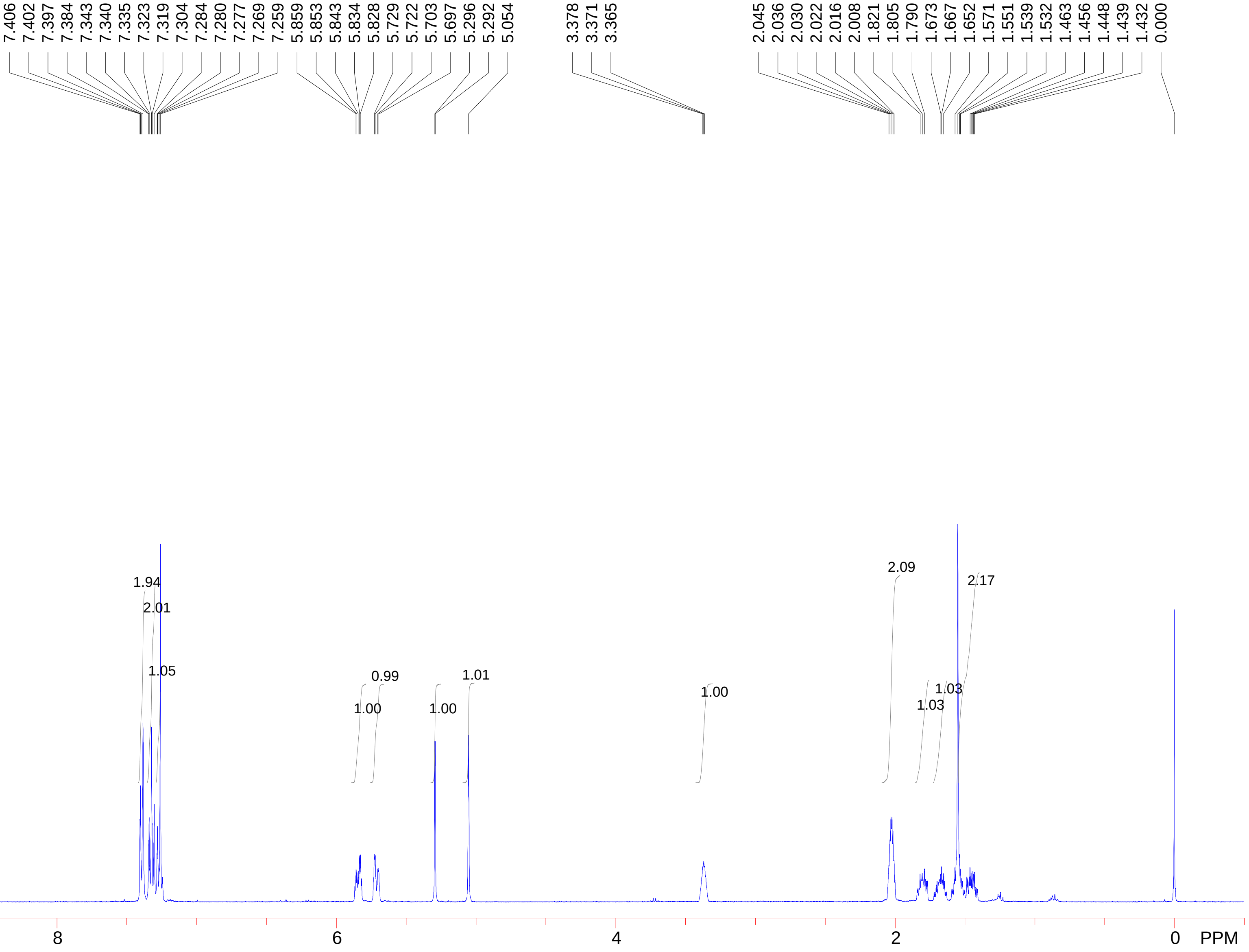

Supplementary Figure 51. ^{1}H NMR spectrum of **3h**

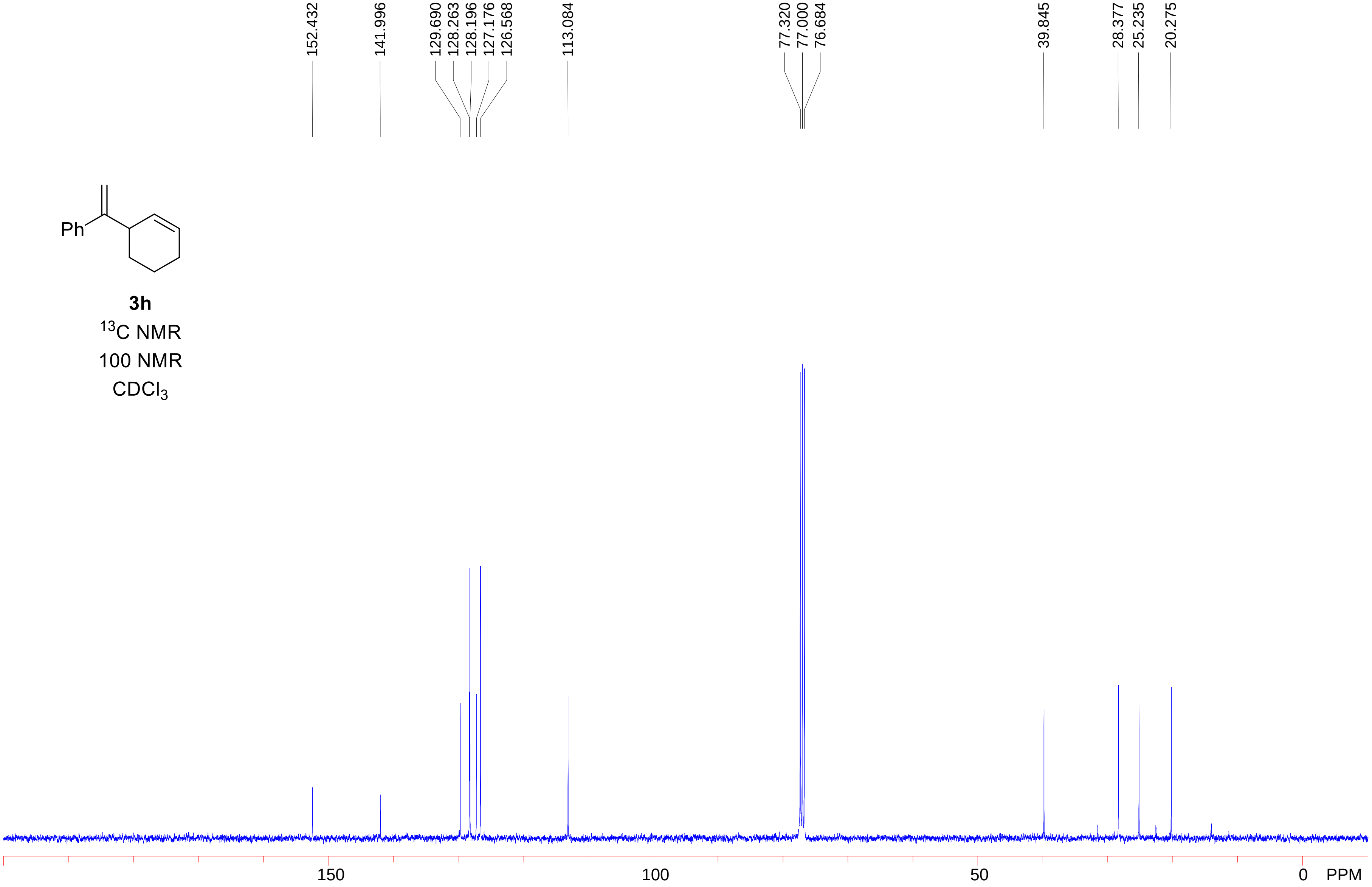

Supplementary Figure 52. ^{13}C NMR spectrum of **3h**

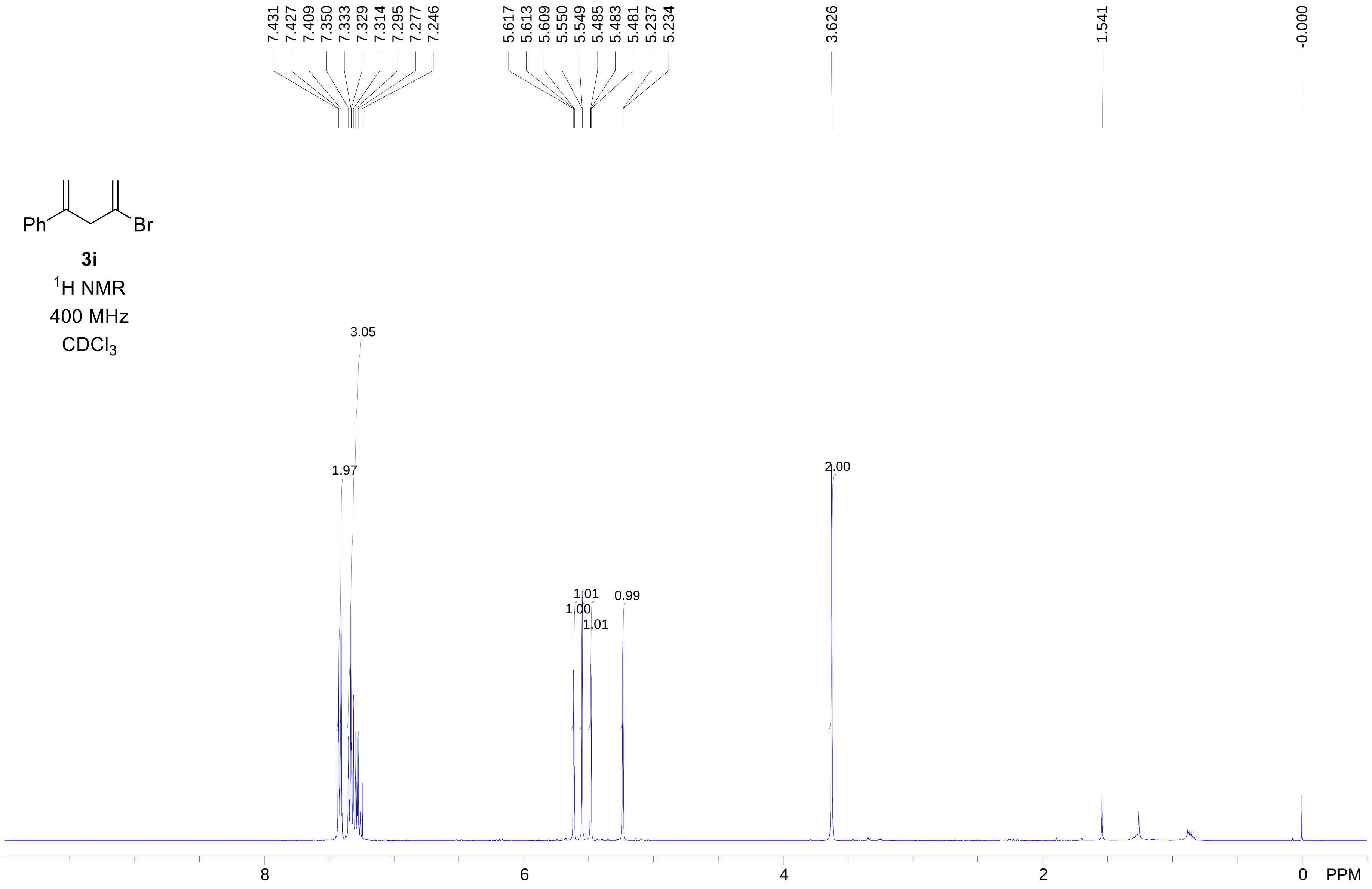

Supplementary Figure 53. ^{1}H NMR spectrum of **3i**

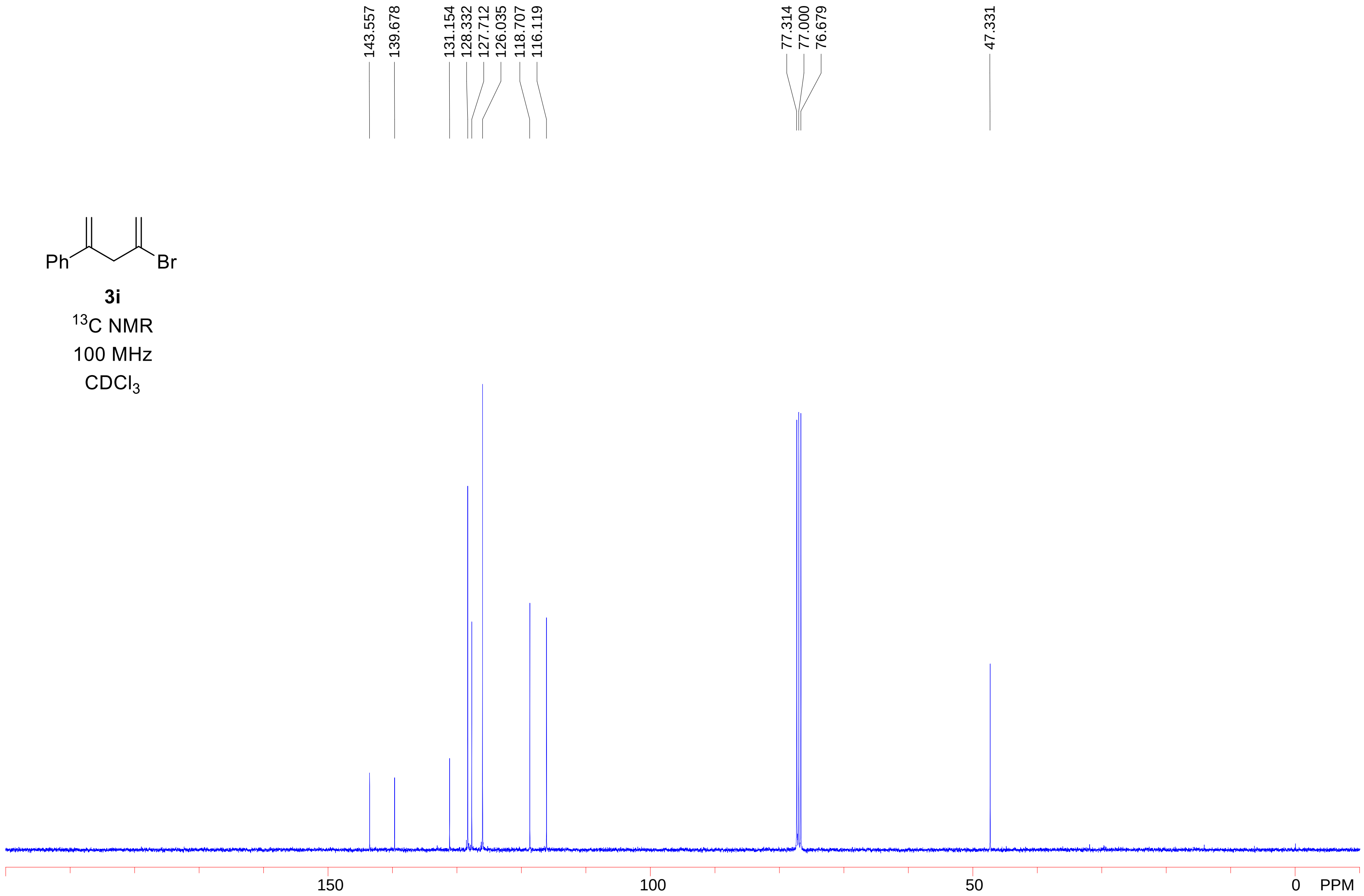

Supplementary Figure 54. ^{13}C NMR spectrum of **3i**

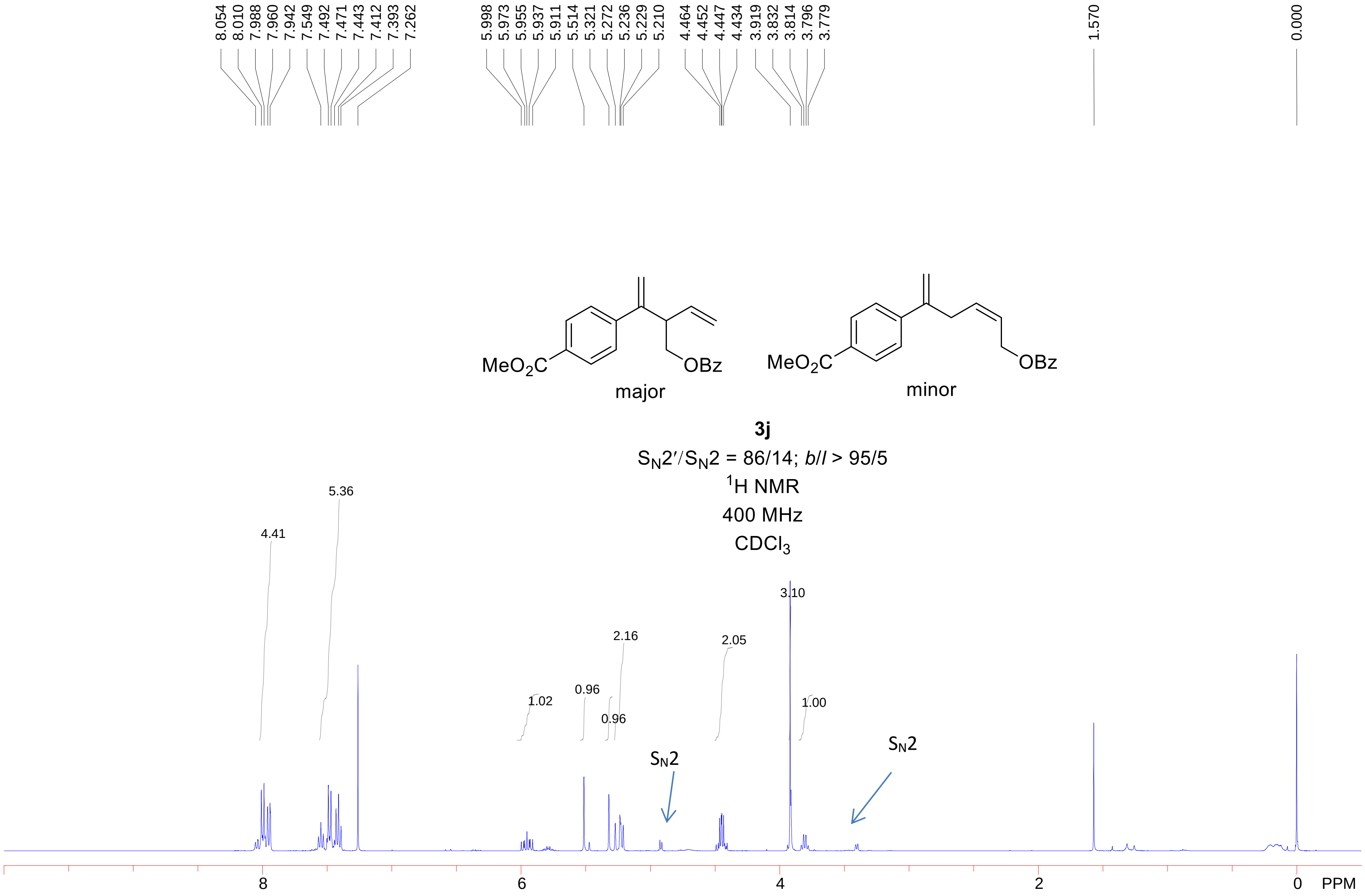

Supplementary Figure 55. ^{1}H NMR spectrum of **3j**

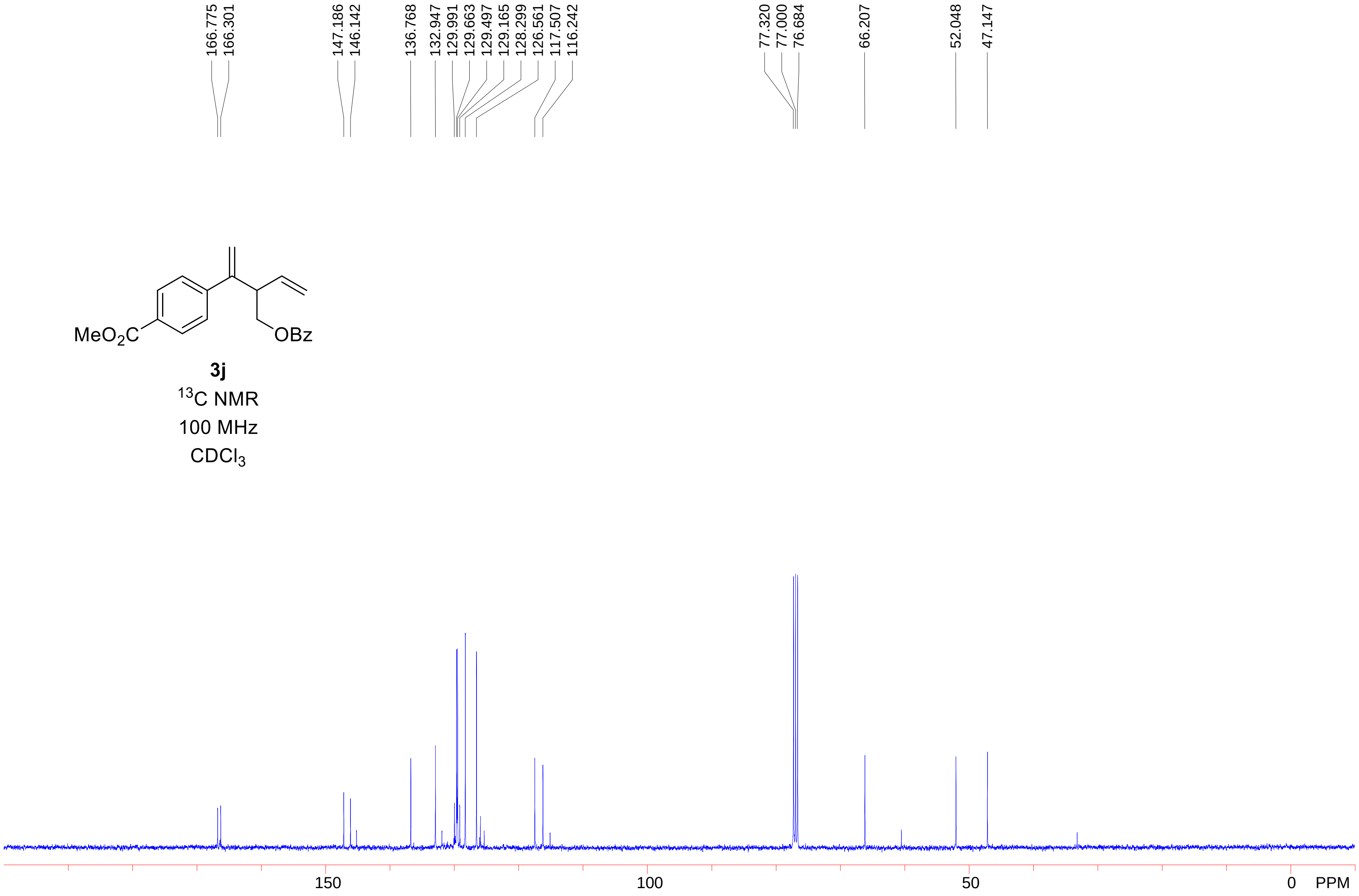

Supplementary Figure 56. ^{13}C NMR spectrum of **3j**

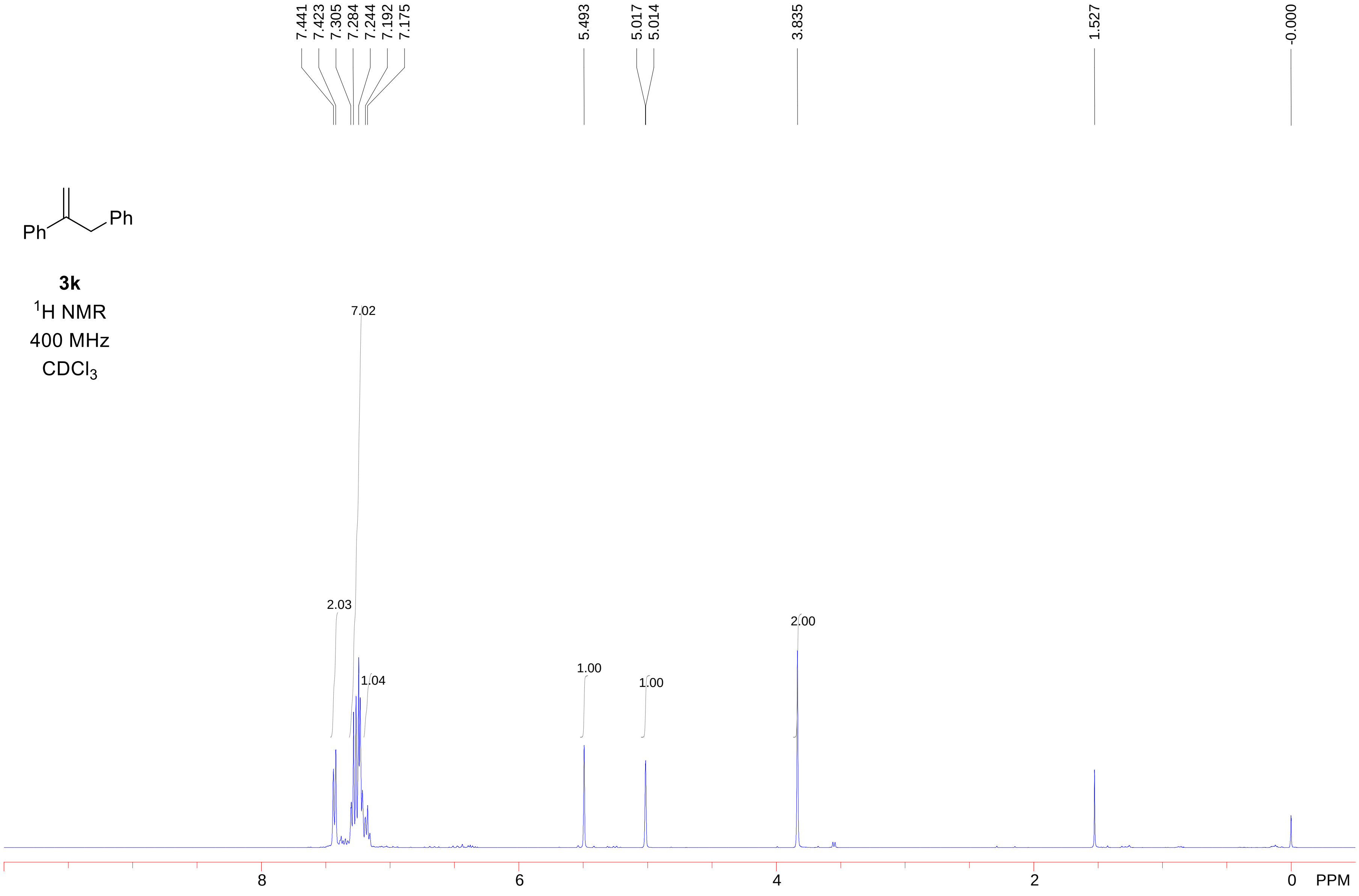

Supplementary Figure 57. ^{1}H NMR spectrum of **3k**

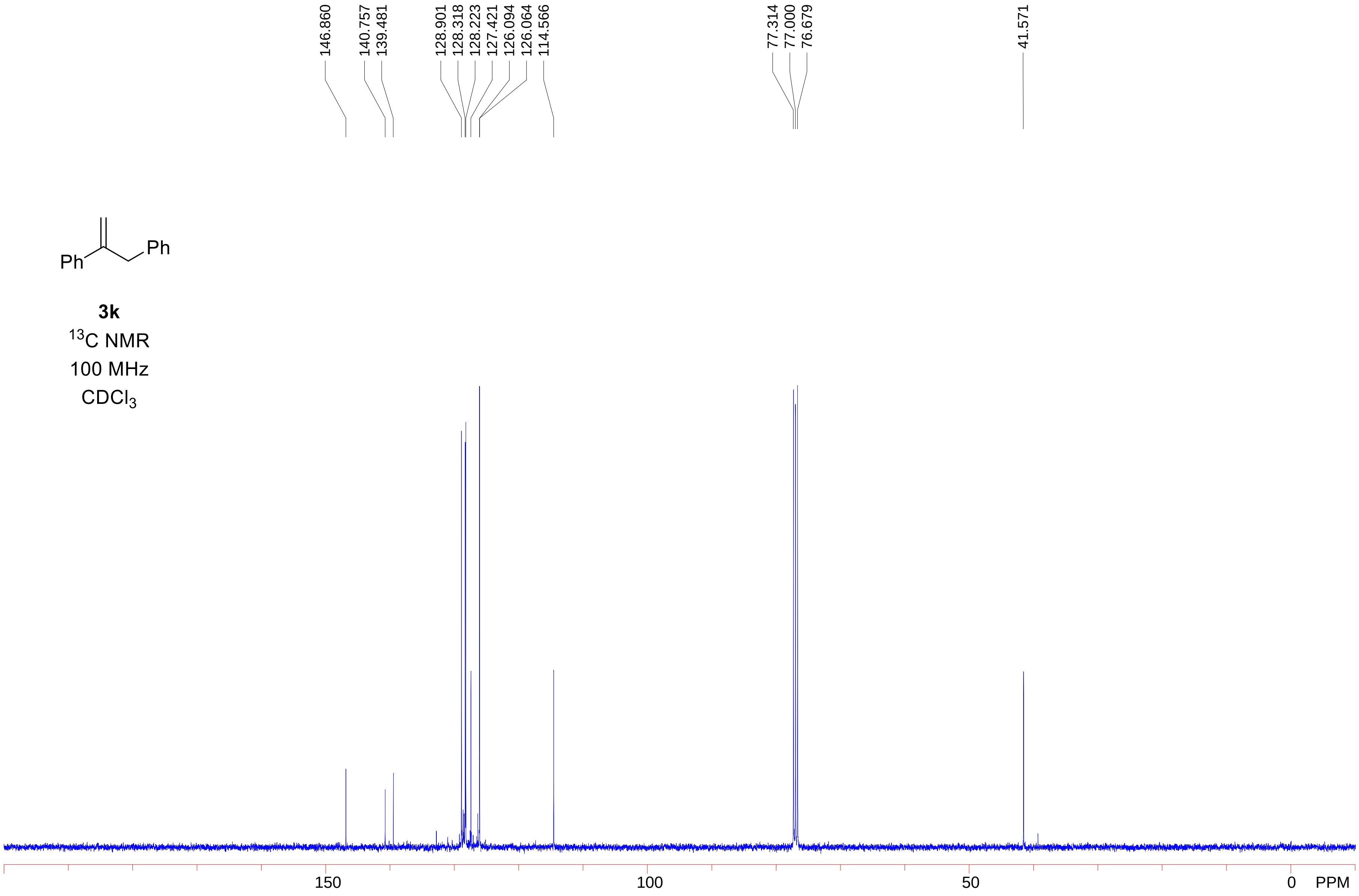

Supplementary Figure 58. ^{13}C NMR spectrum of **3k**

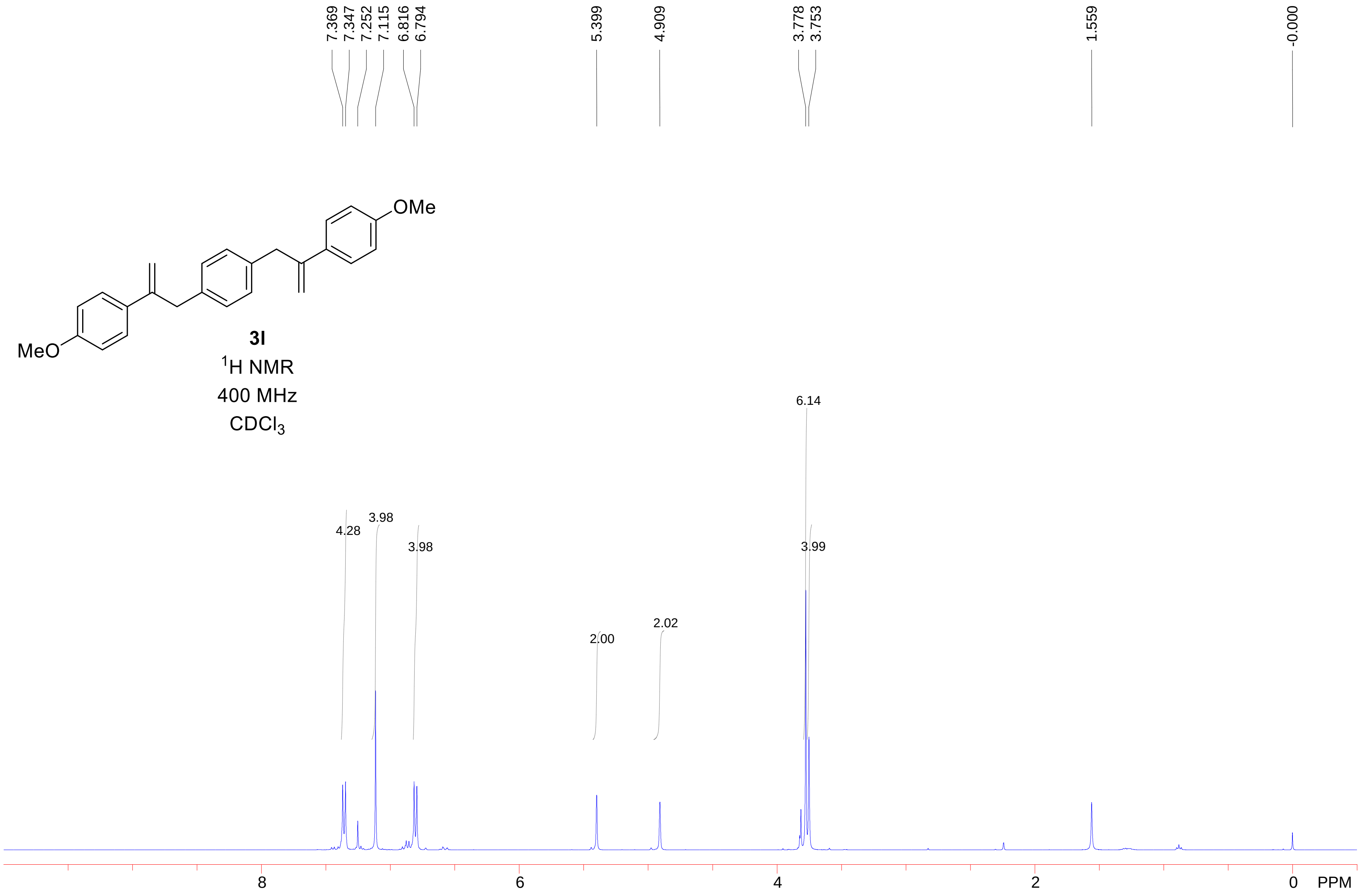

Supplementary Figure 59. ^{1}H NMR spectrum of **3l**

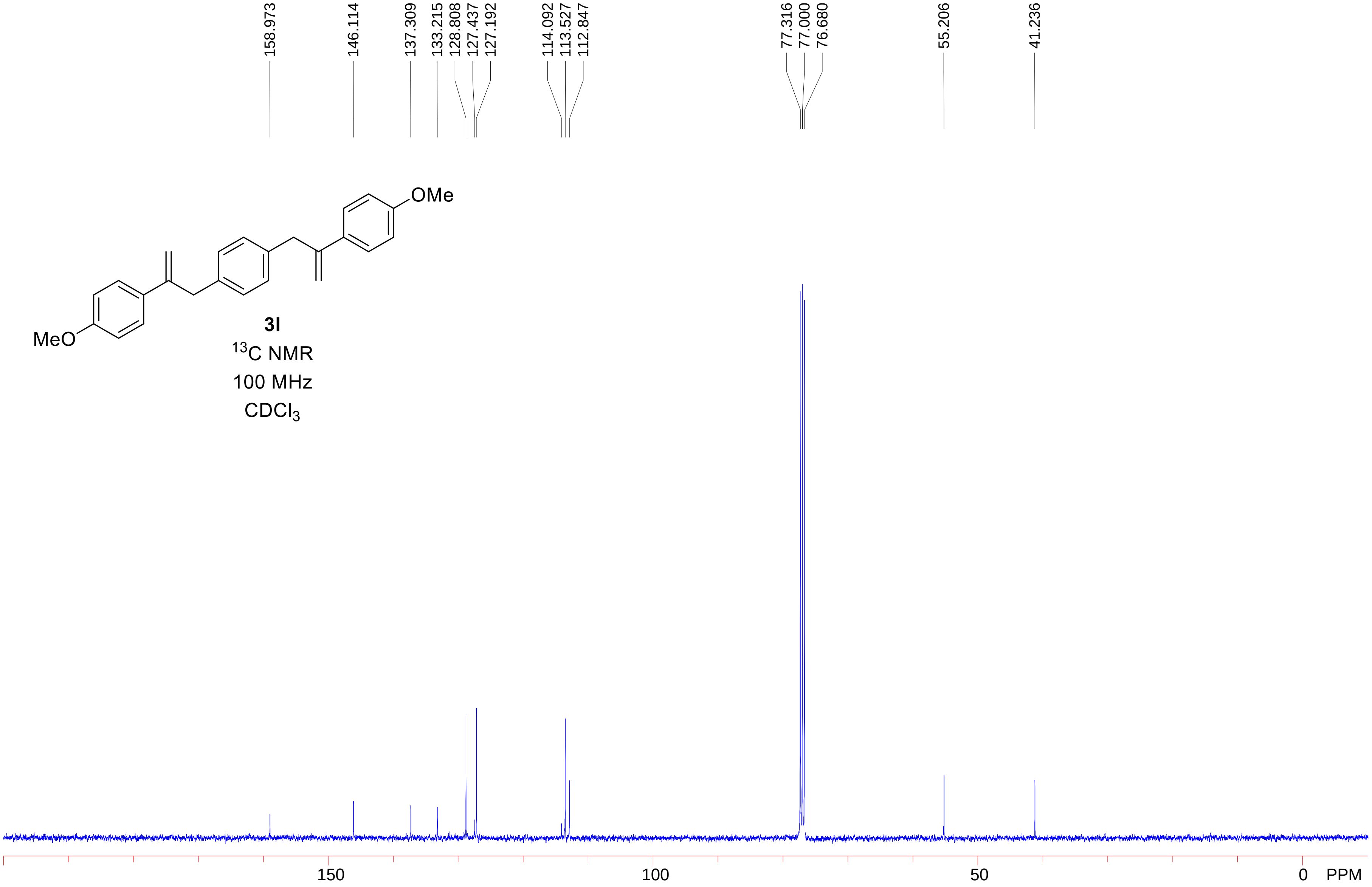

Supplementary Figure 60. ^{13}C NMR spectrum of **3l**

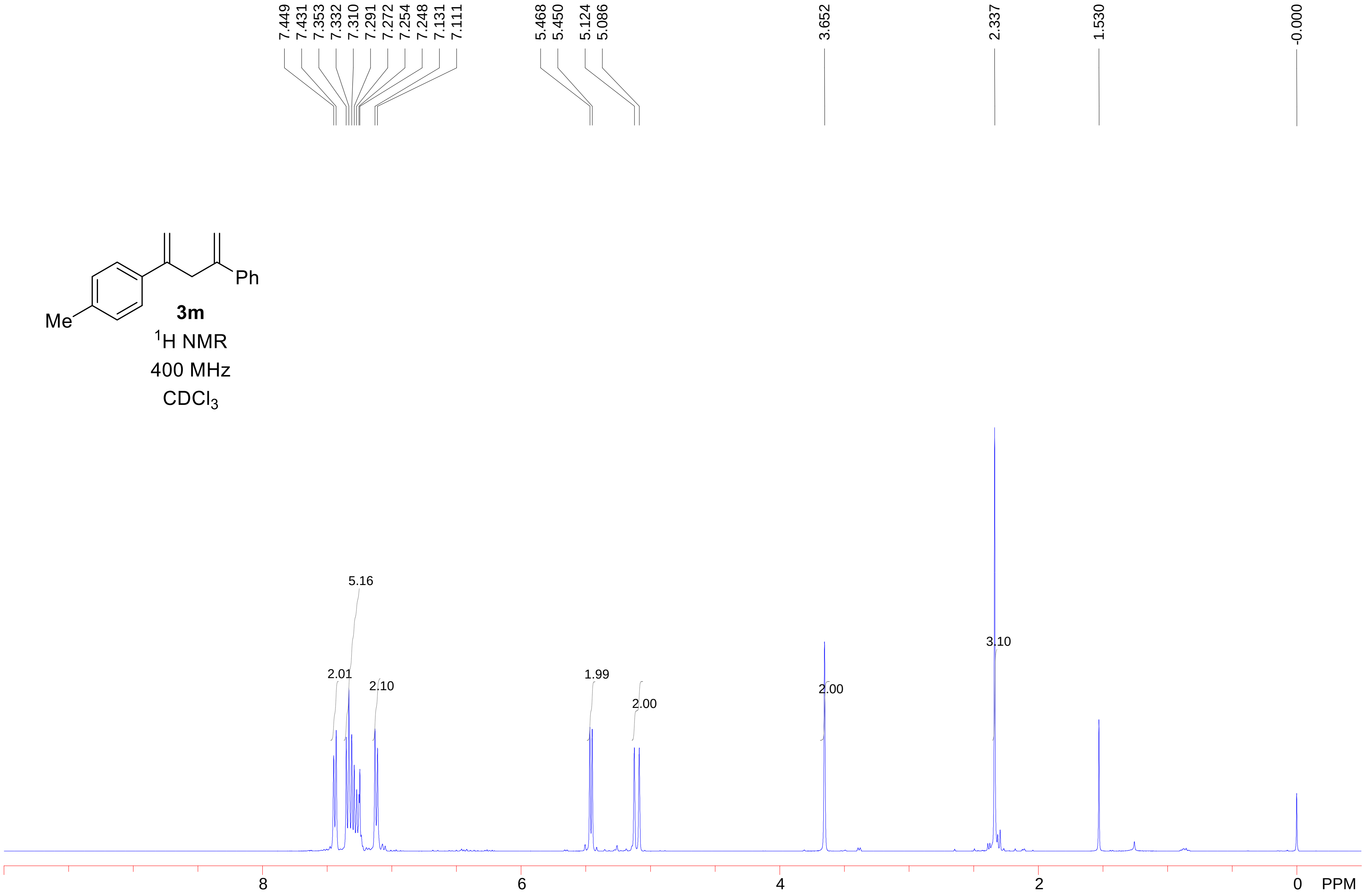

Supplementary Figure 61. ^{1}H NMR spectrum of **3m**

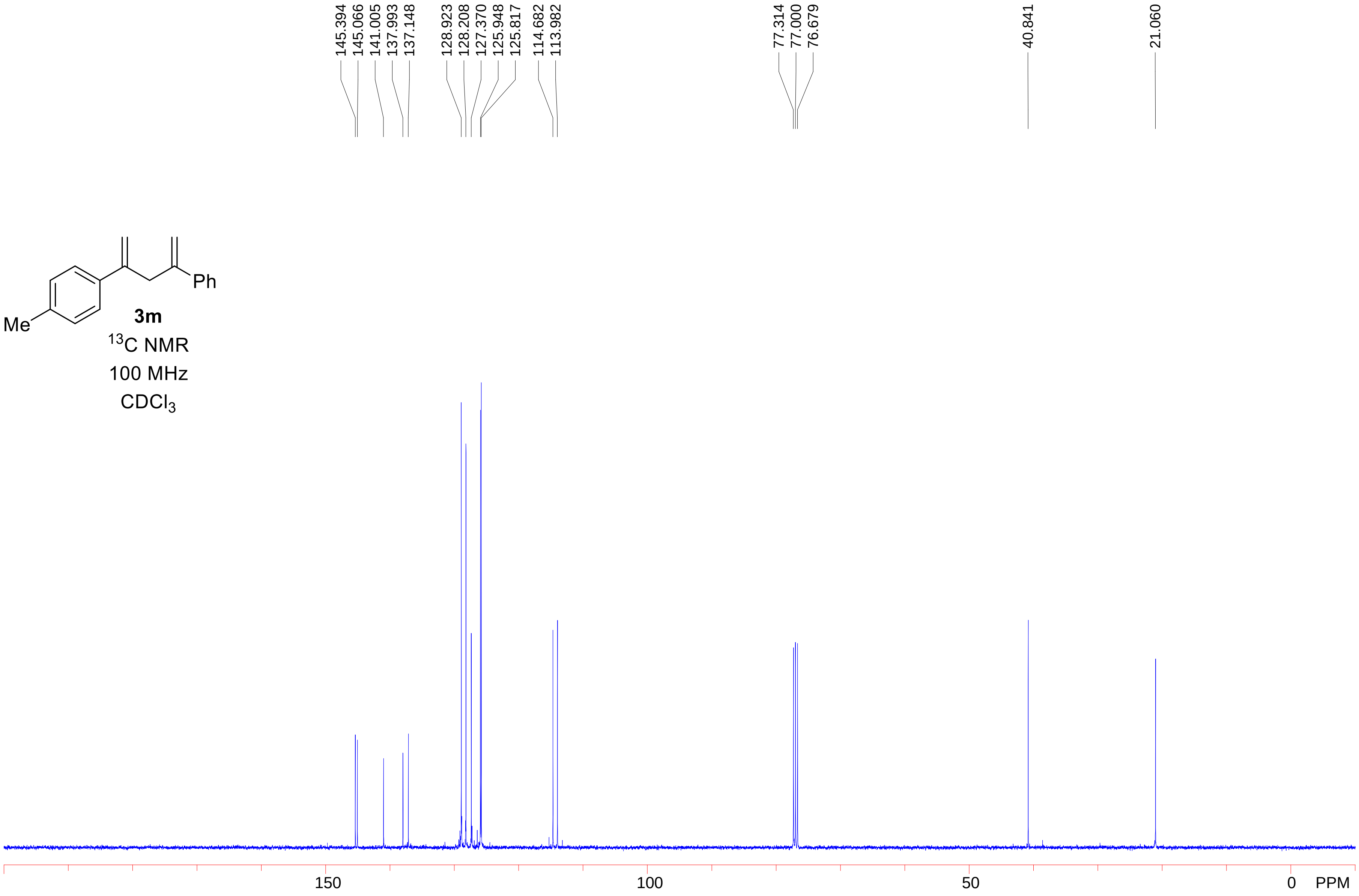

Supplementary Figure 62. ^{13}C NMR spectrum of **3m**

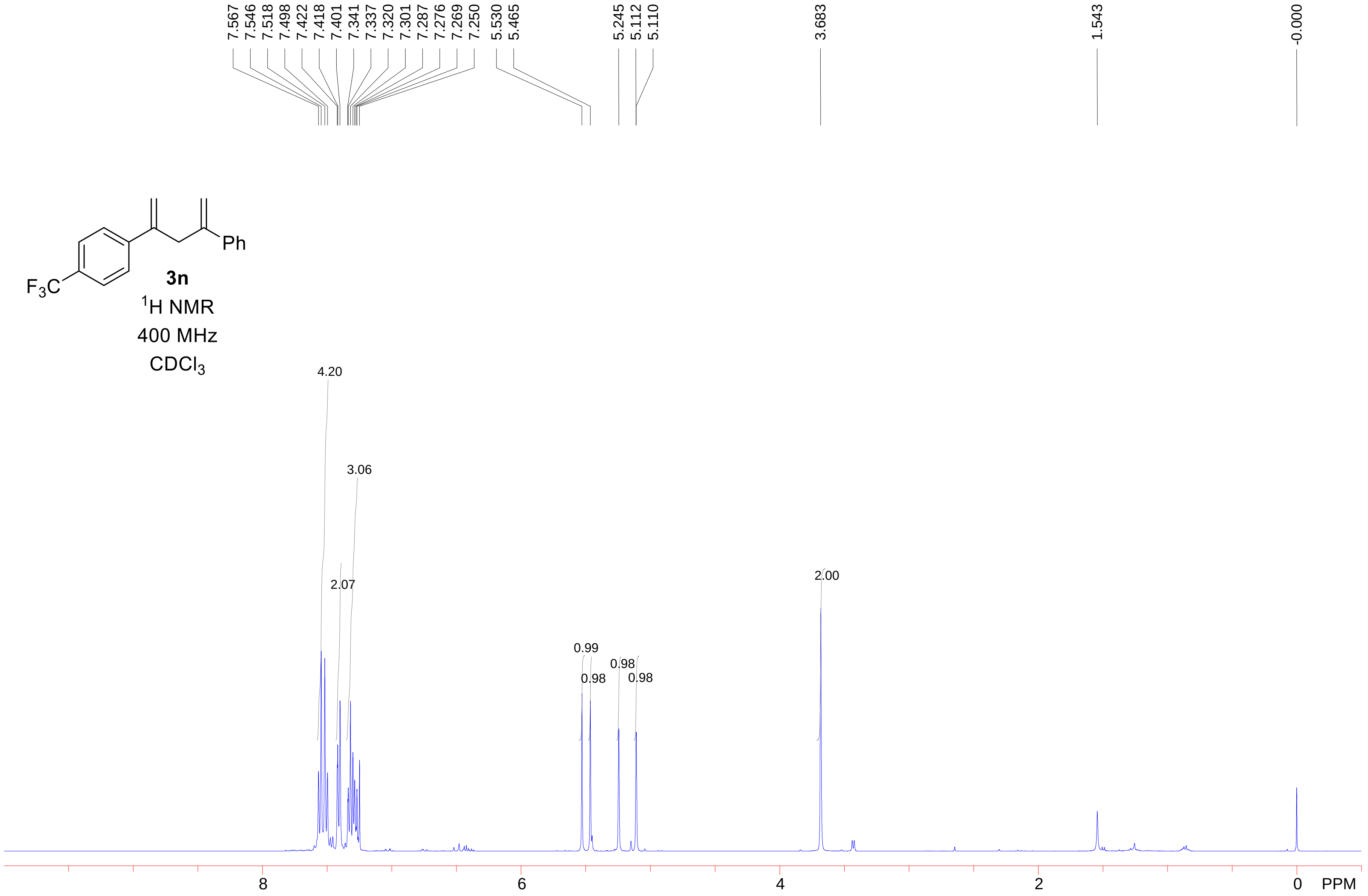

Supplementary Figure 63. ^{1}H NMR spectrum of **3n**

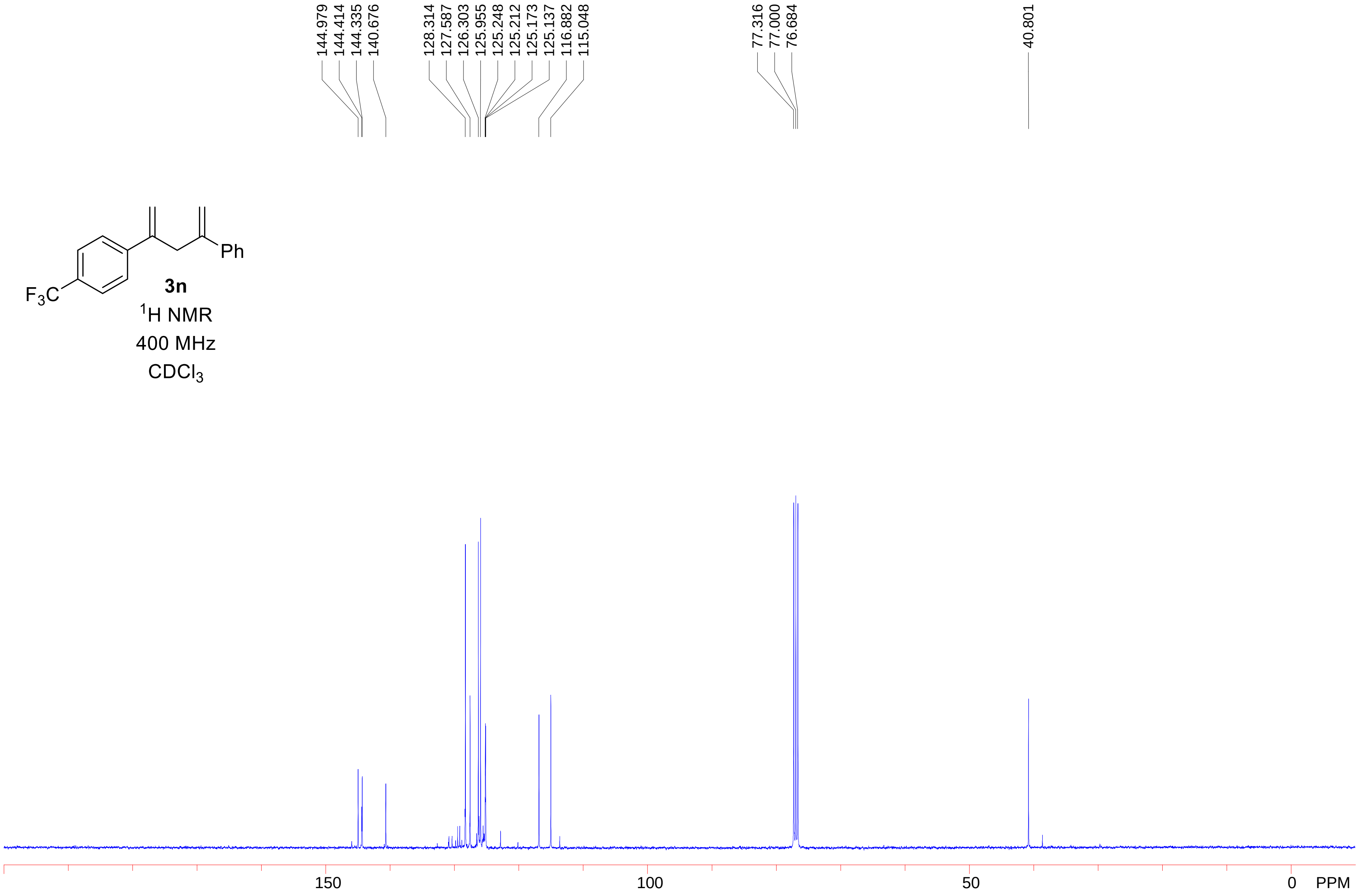

Supplementary Figure 64. ^{13}C NMR spectrum of **3n**

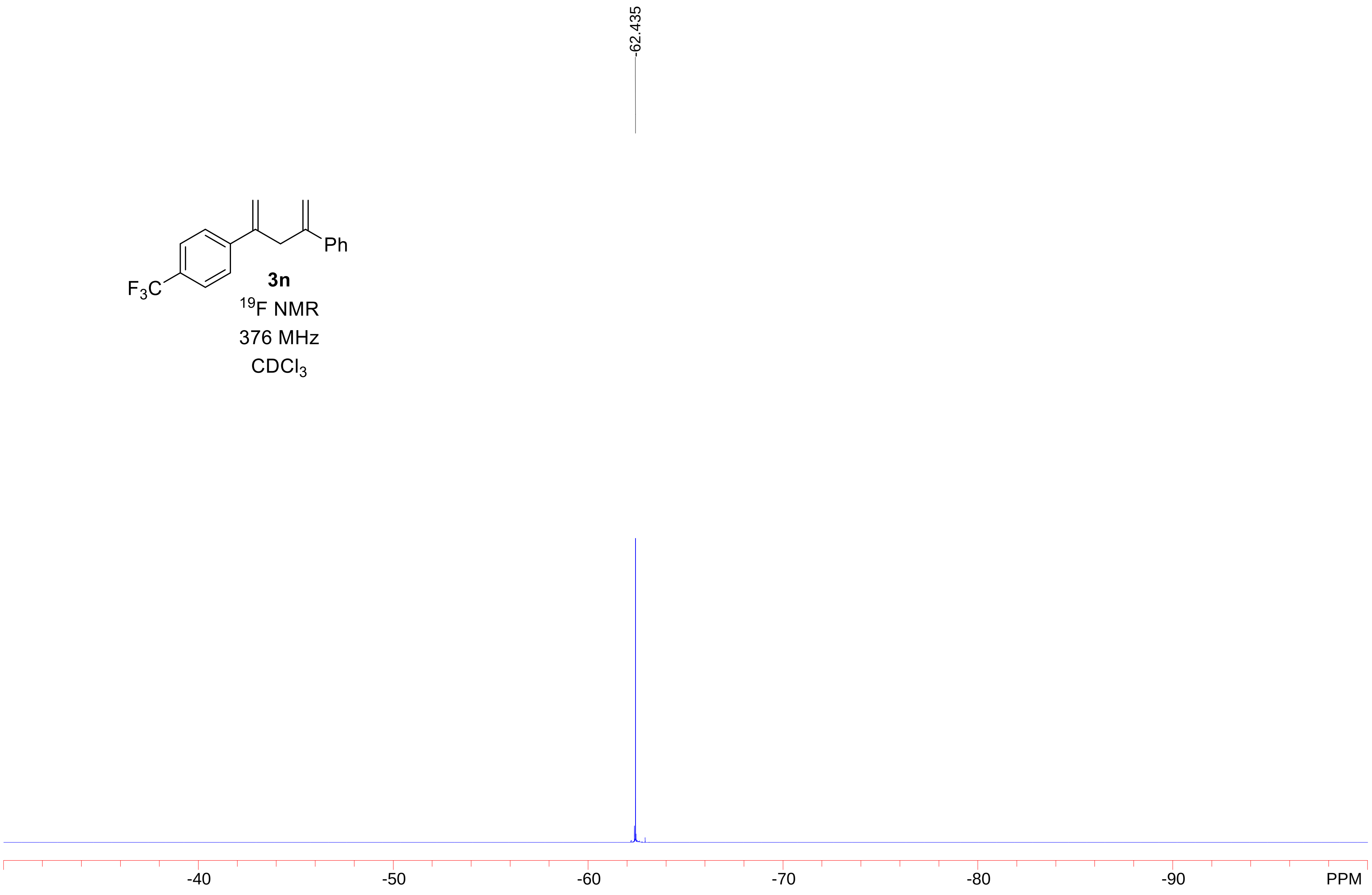

Supplementary Figure 65. ^{19}F NMR spectrum of **3n**

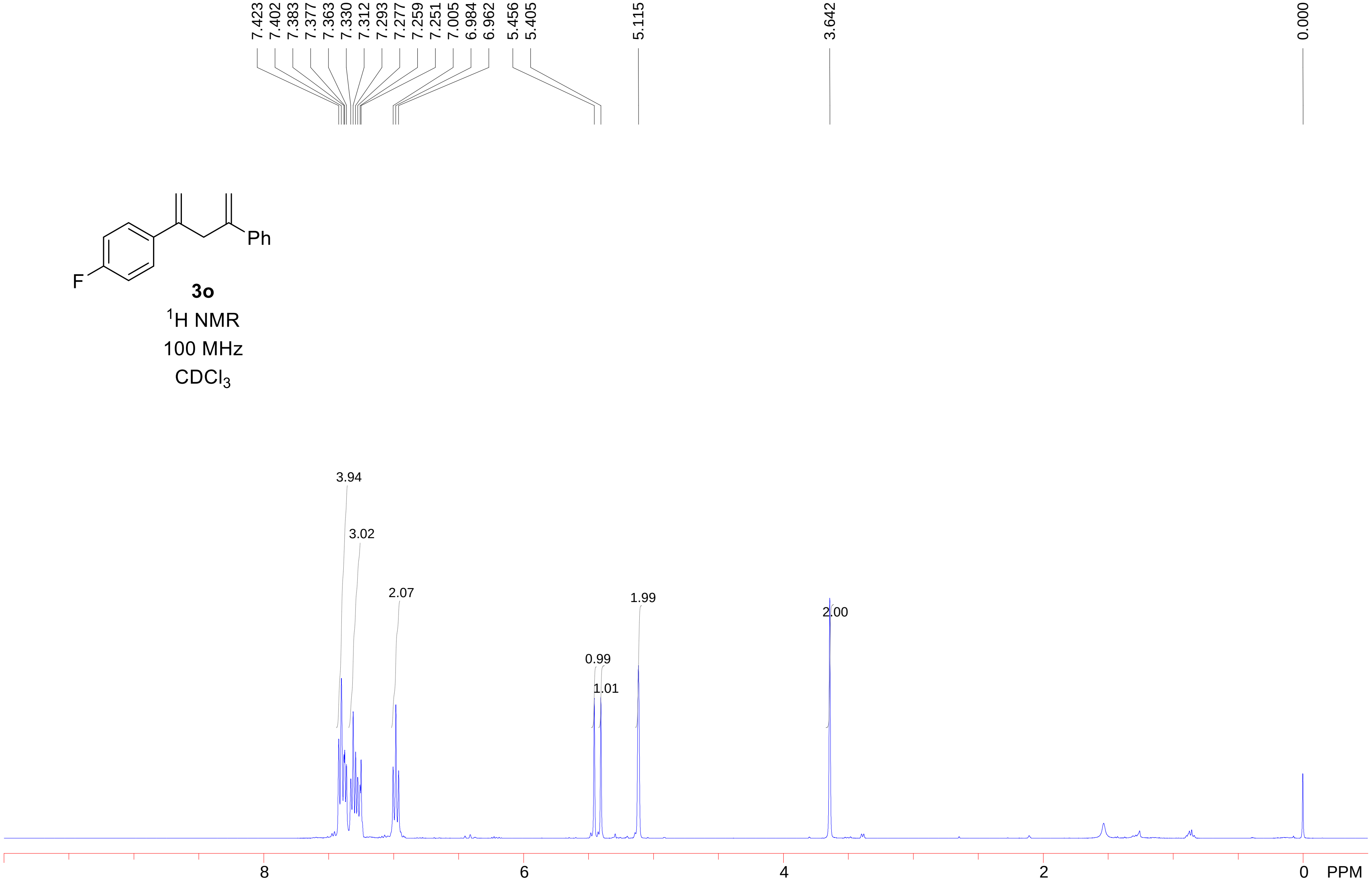

Supplementary Figure 66. ^{1}H NMR spectrum of **3o**

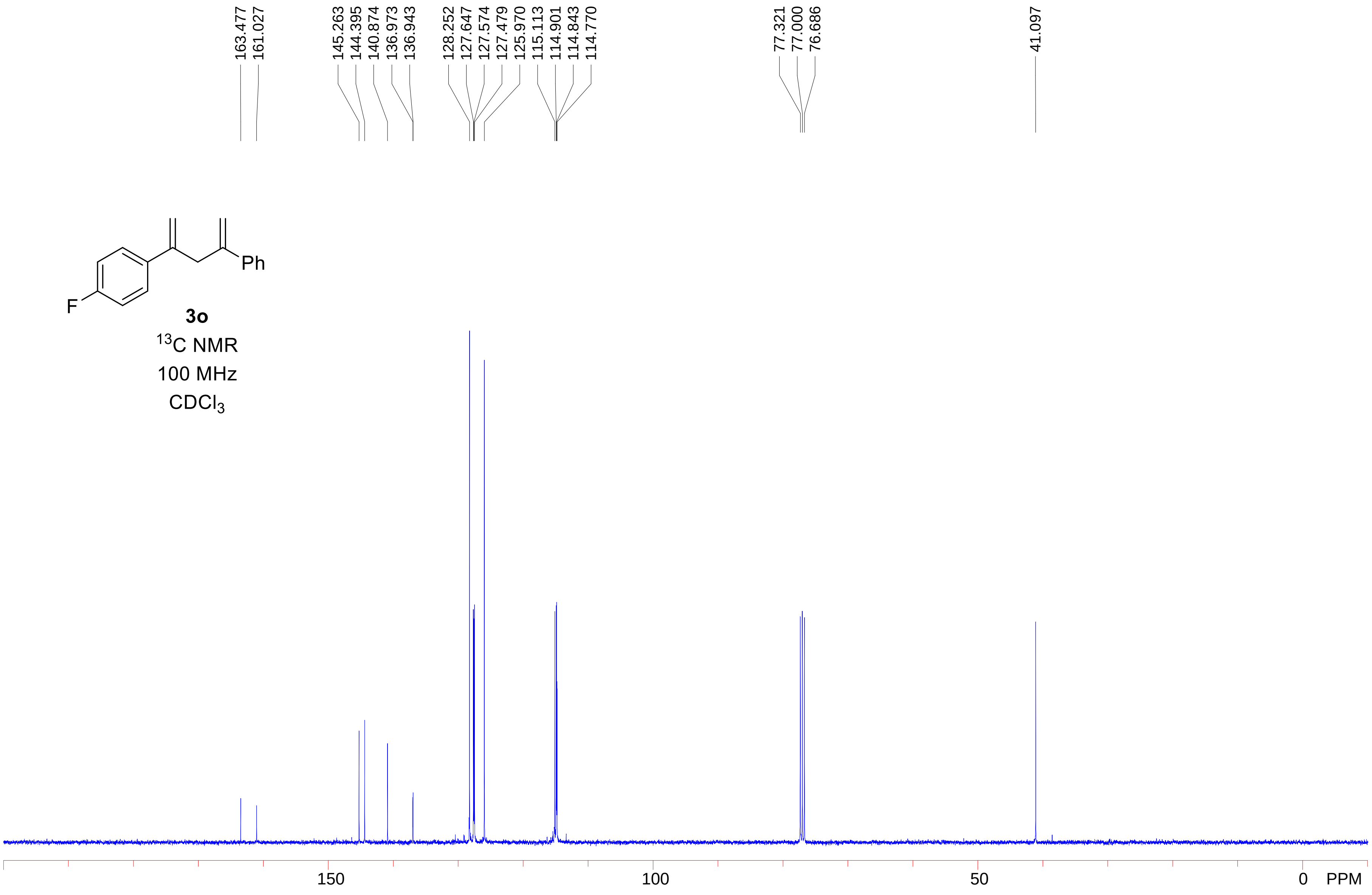

Supplementary Figure 67. ^{13}C NMR spectrum of **3o**

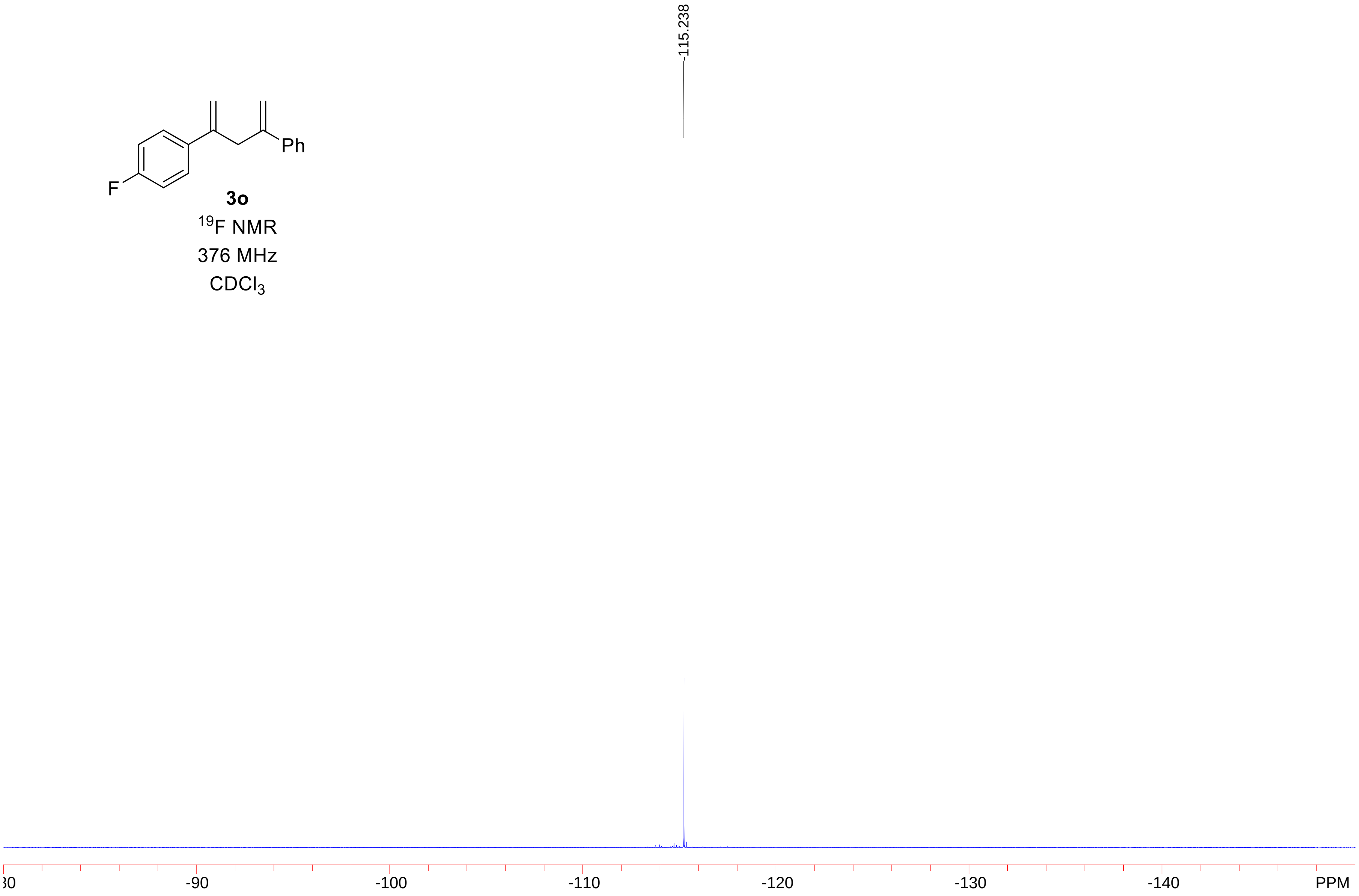

Supplementary Figure 68. ^{1}H NMR spectrum of **3o**

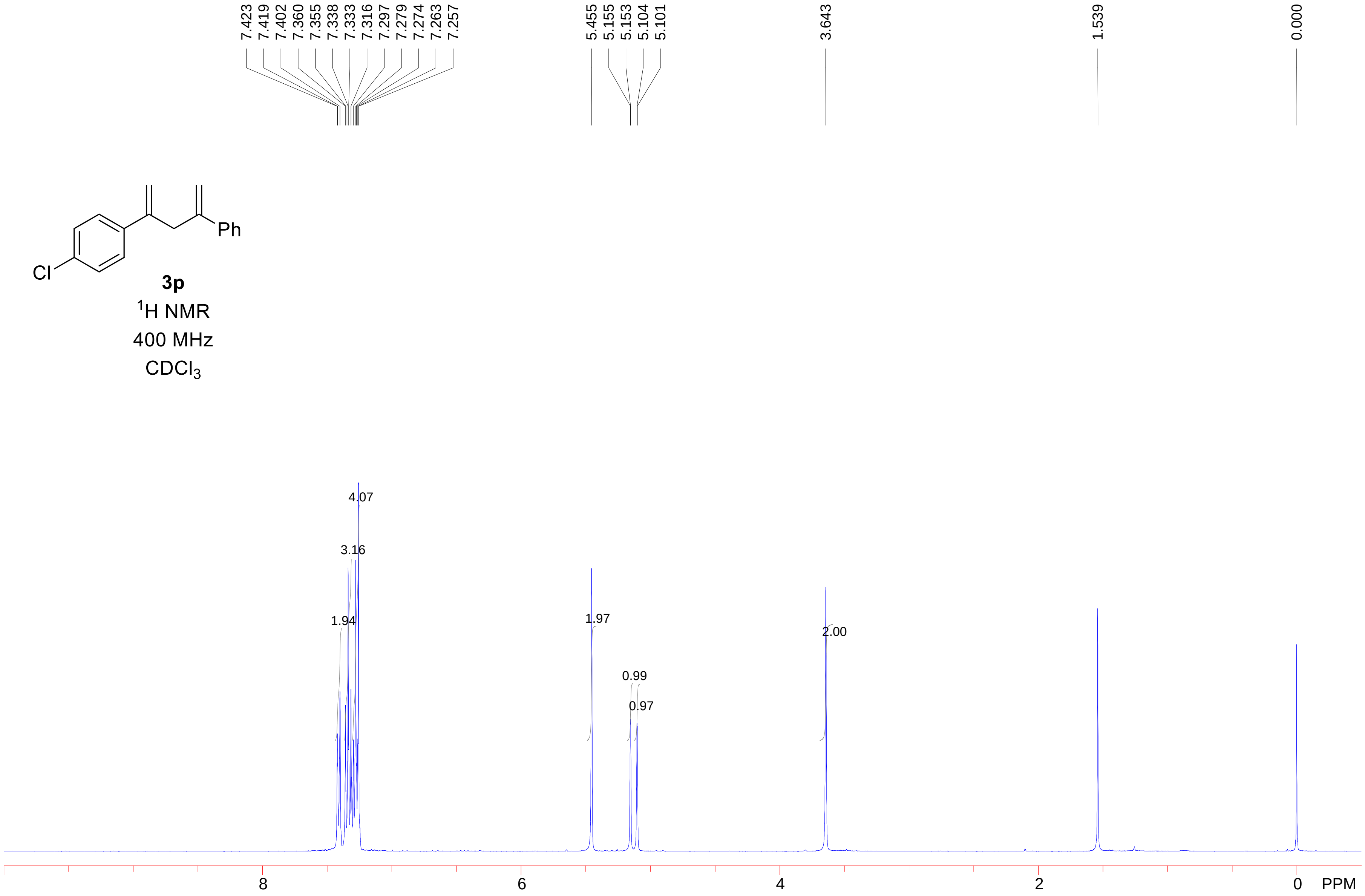

Supplementary Figure 69. ^{1}H NMR spectrum of **3p**

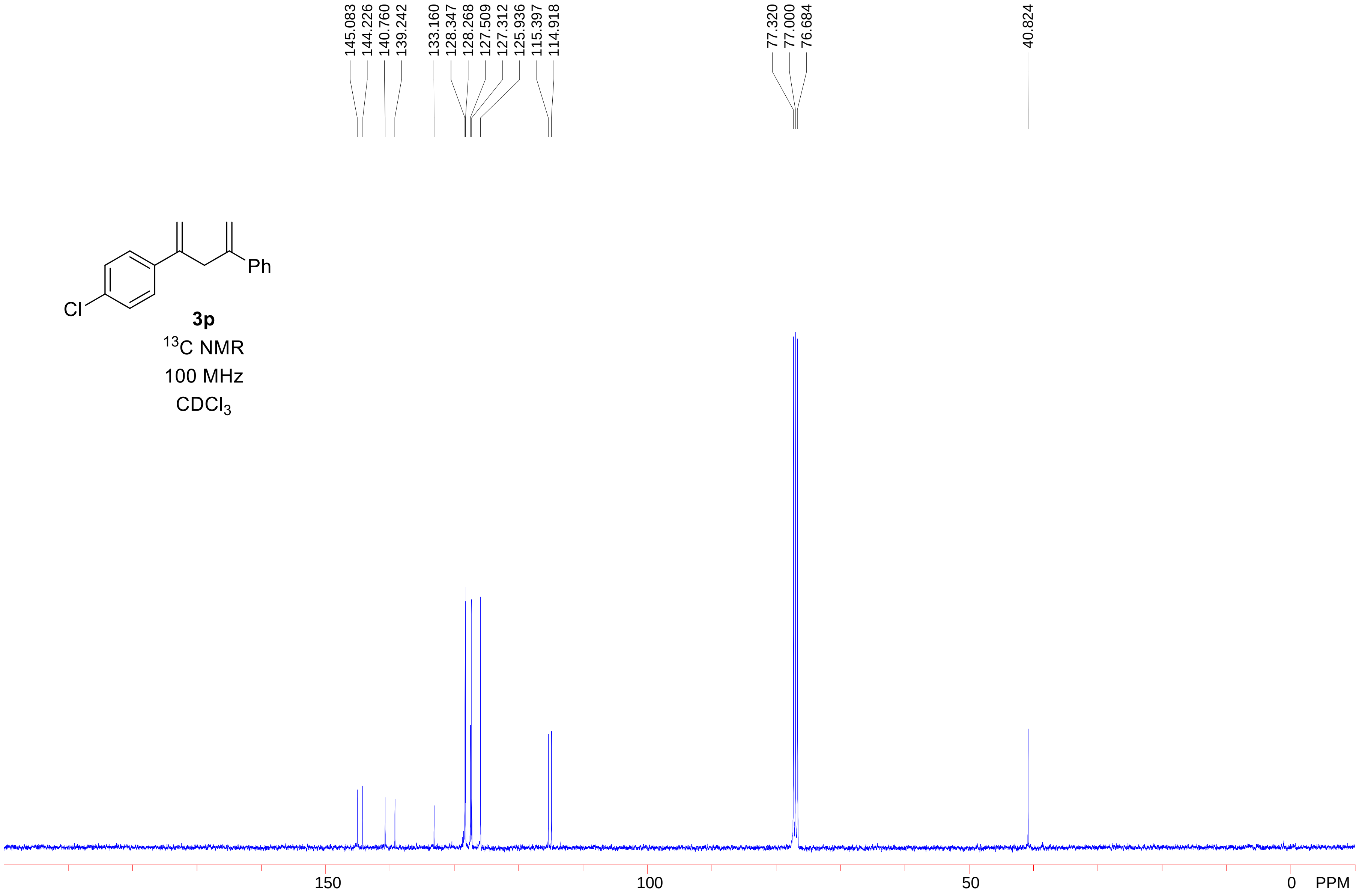

Supplementary Figure 70. ^{13}C NMR spectrum of **3p**

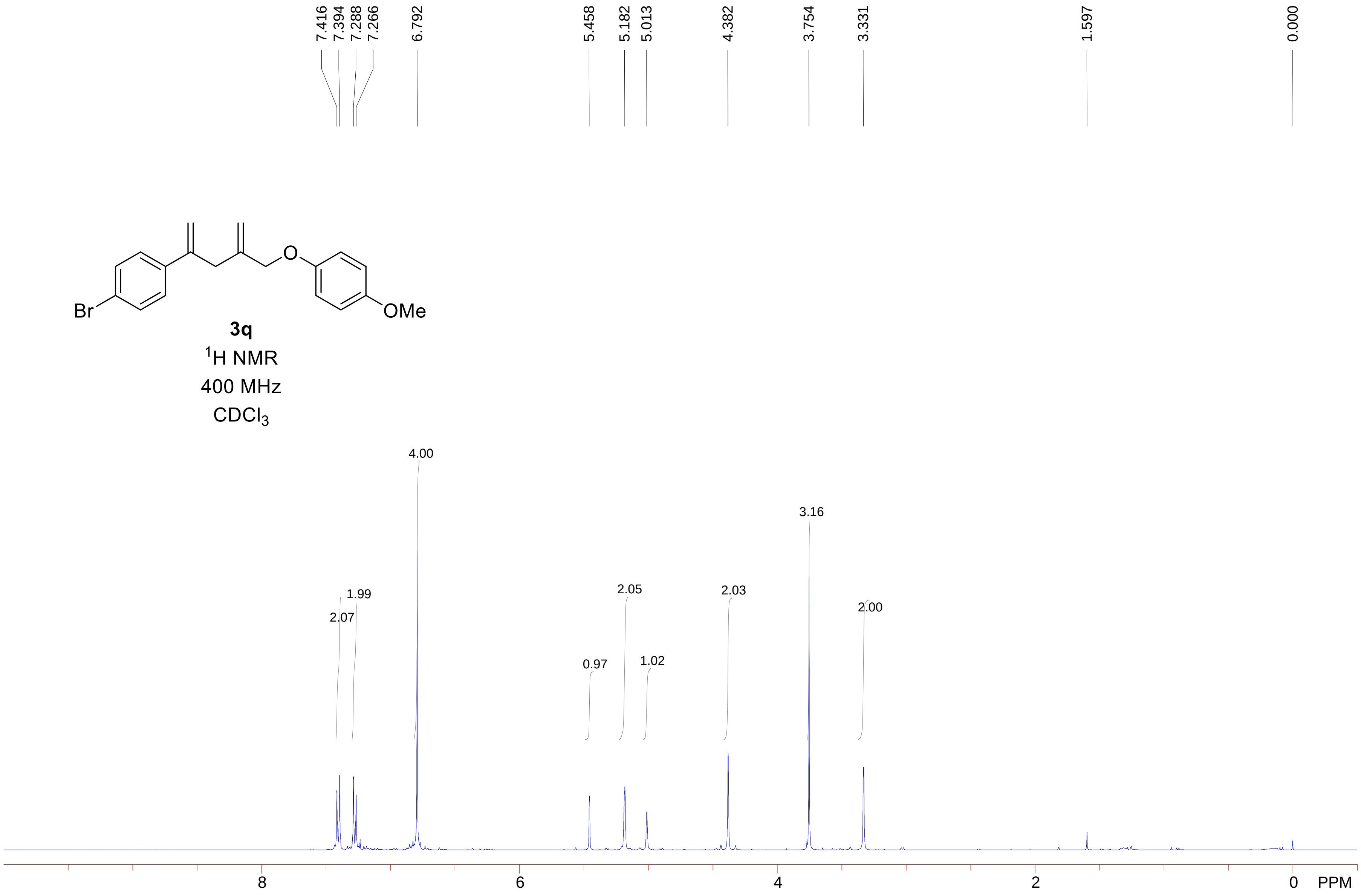

Supplementary Figure 71. ^{1}H NMR spectrum of **3q**

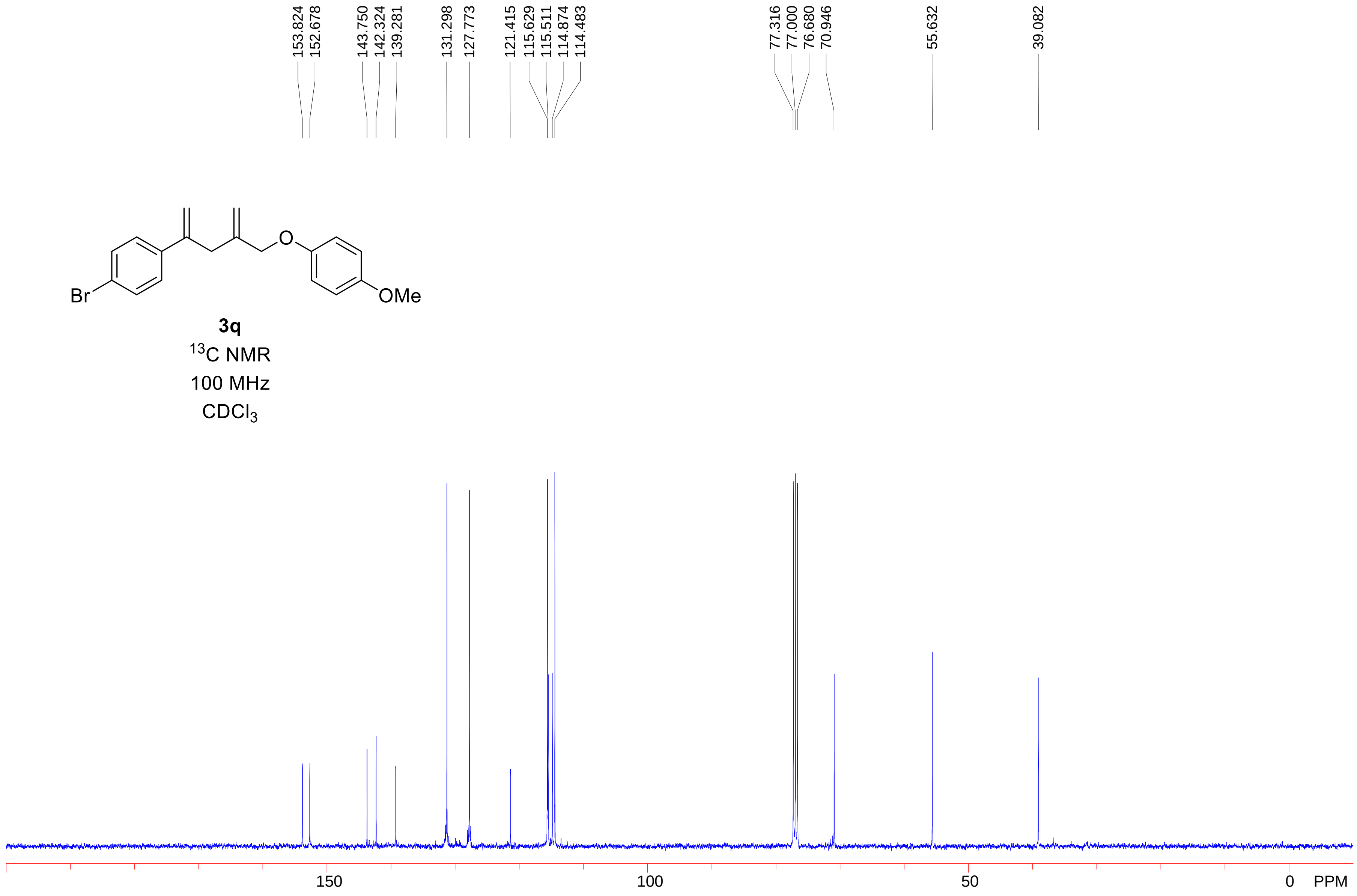

Supplementary Figure 72. ^{13}C NMR spectrum of **3q**

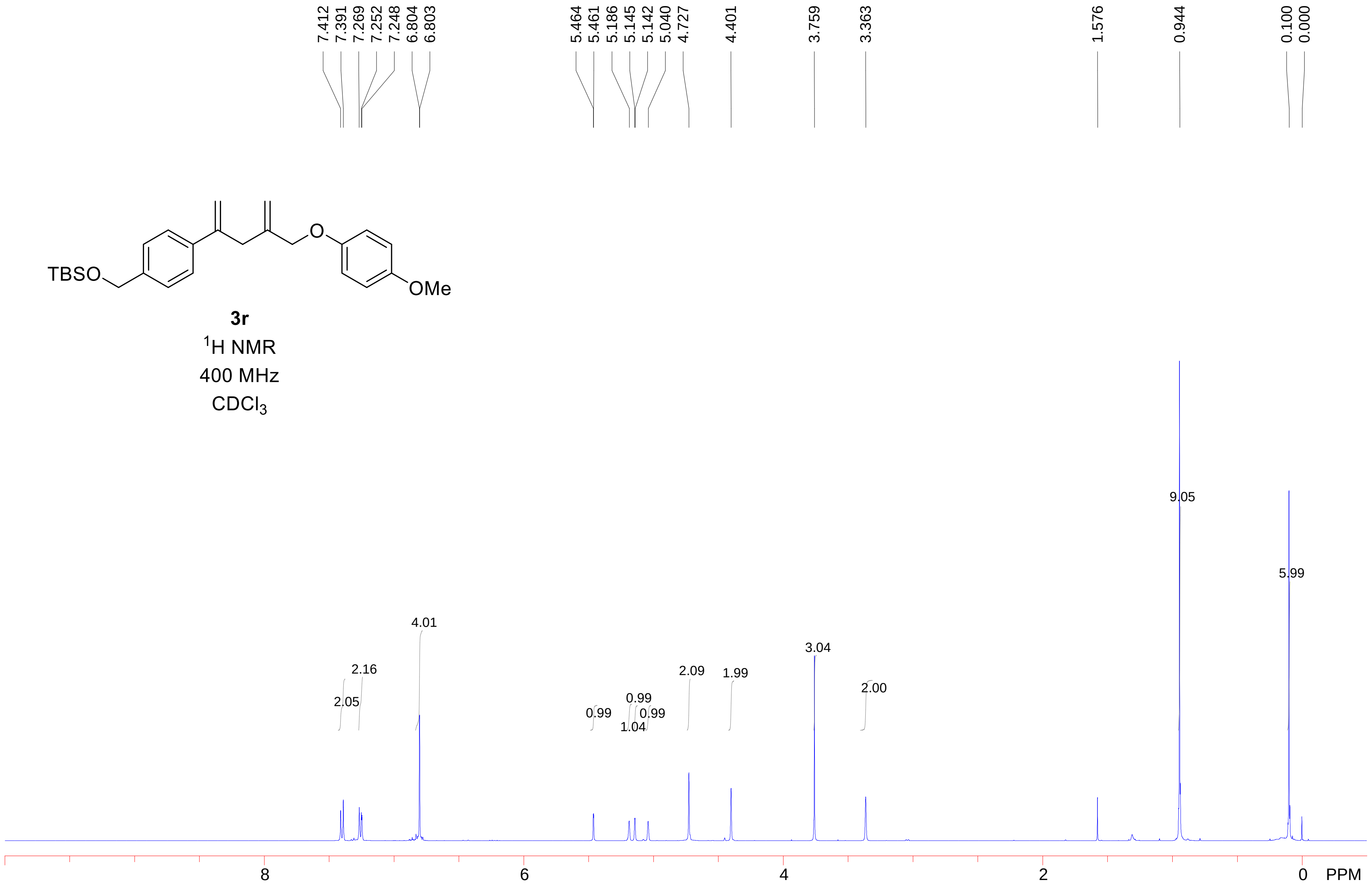

Supplementary Figure 73. ^{1}H NMR spectrum of **3r**

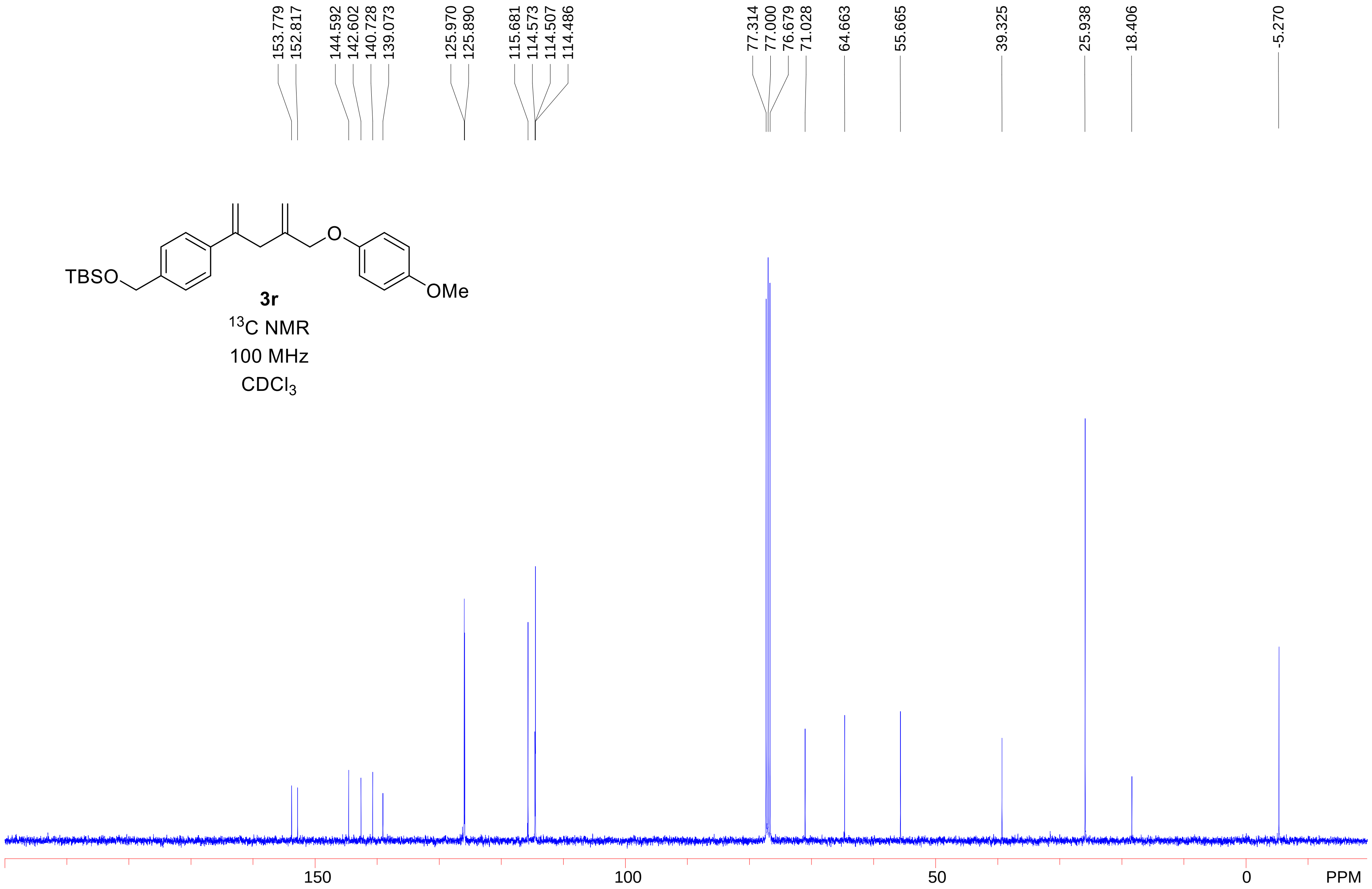

Supplementary Figure 74. ^{13}C NMR spectrum of **3r**

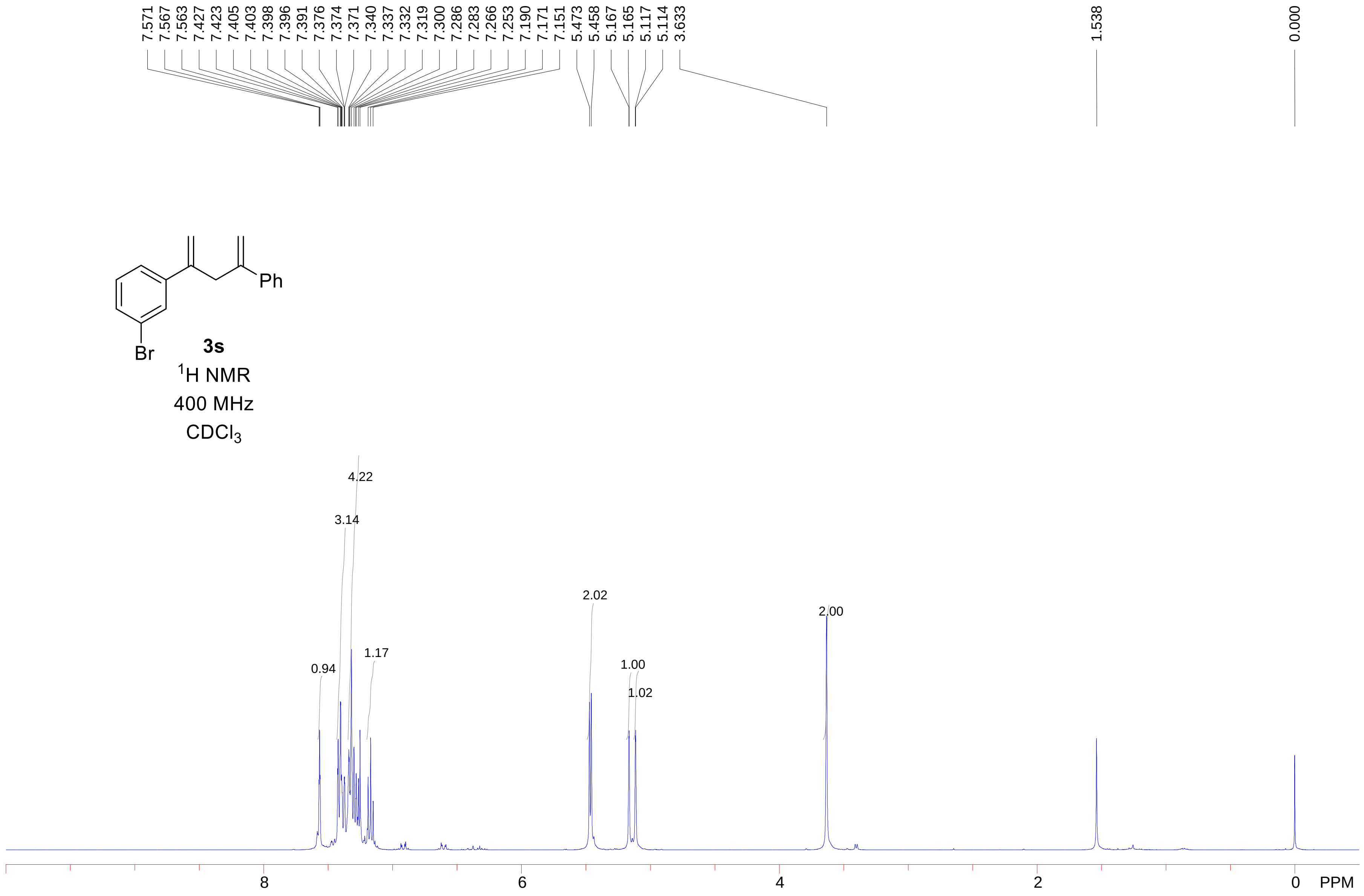

Supplementary Figure 75. ^{1}H NMR spectrum of **3s**

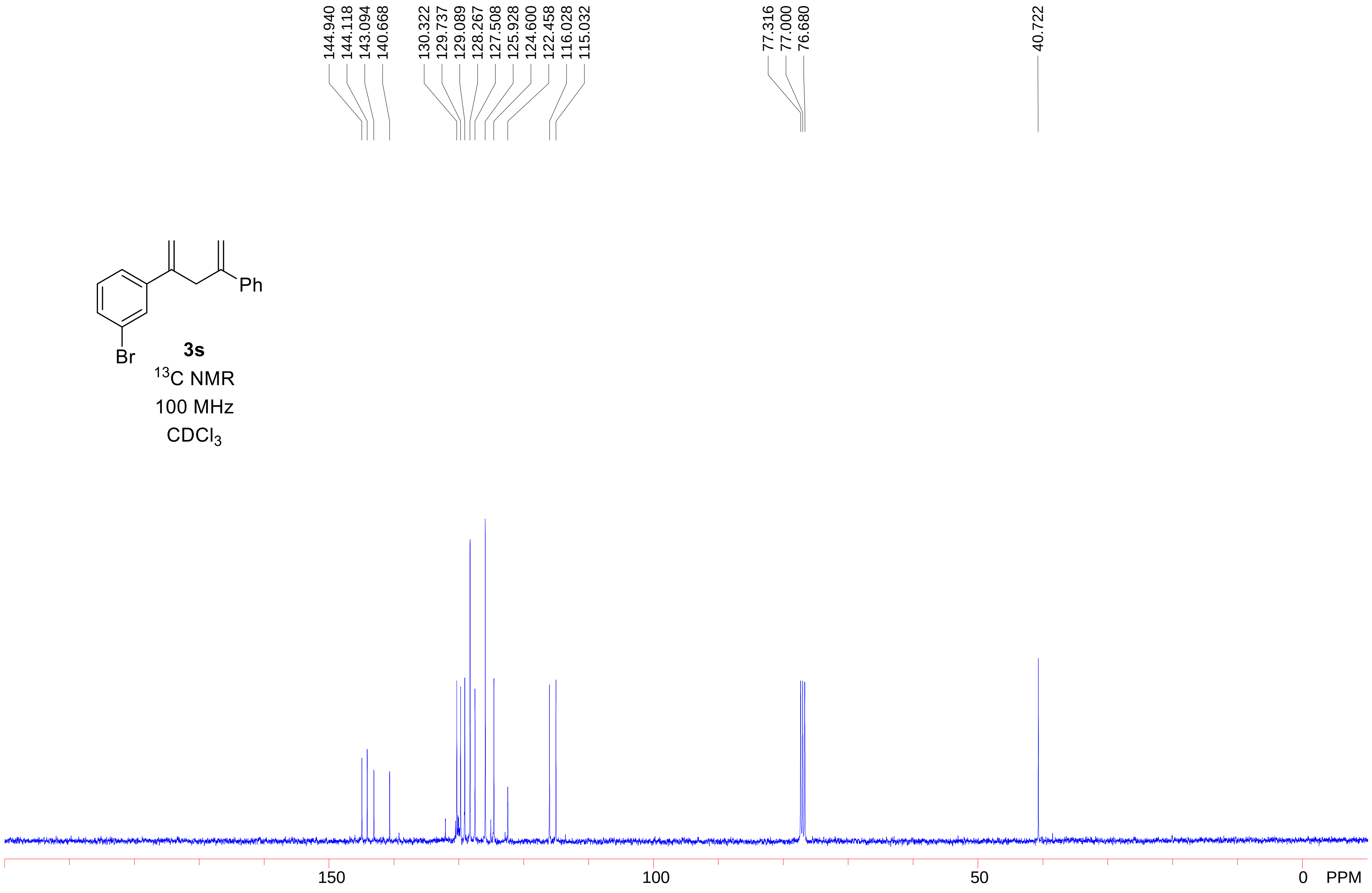

Supplementary Figure 76. ^{13}C NMR spectrum of **3s**

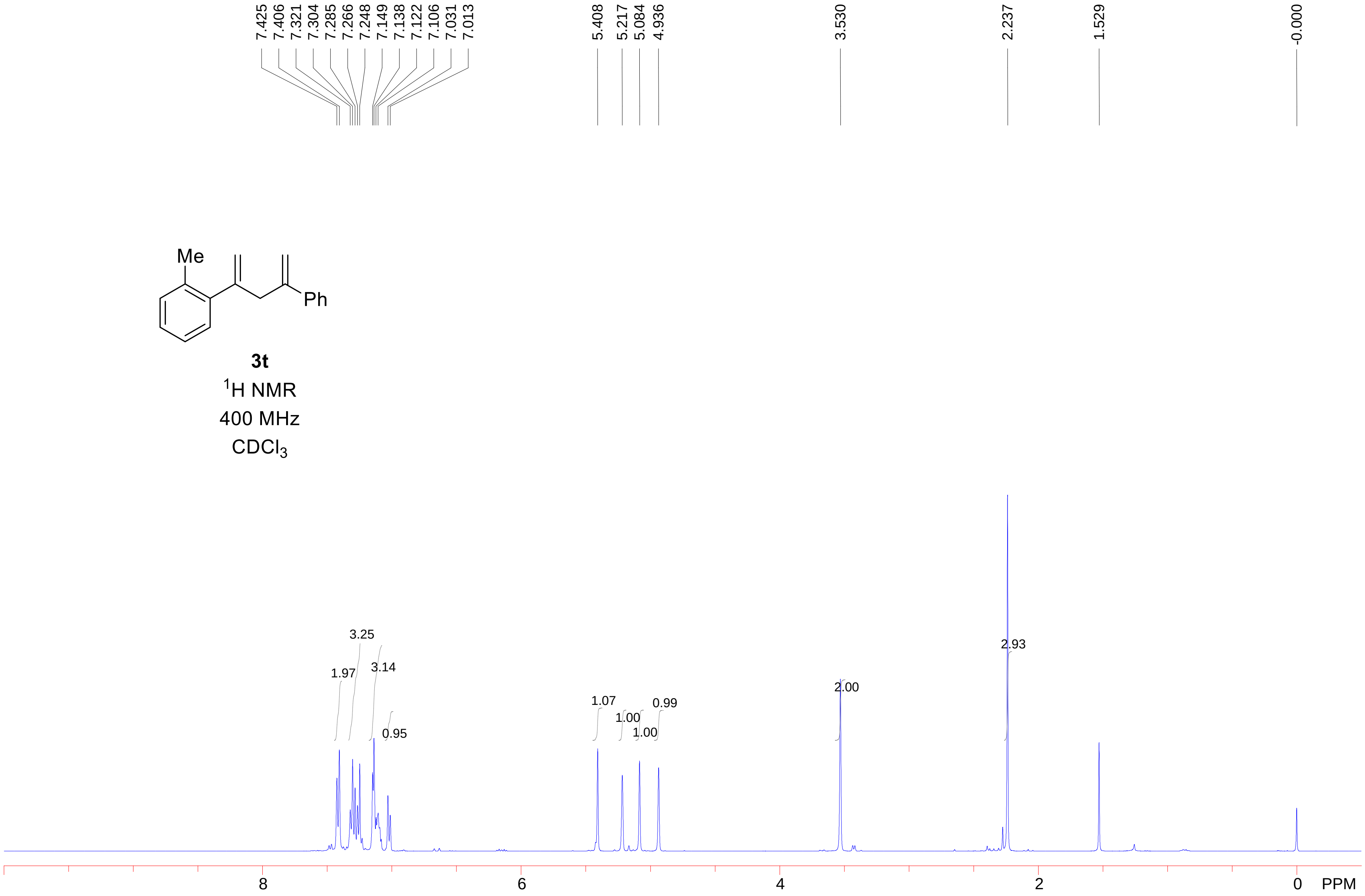

Supplementary Figure 77. ^{1}H NMR spectrum of **3t**

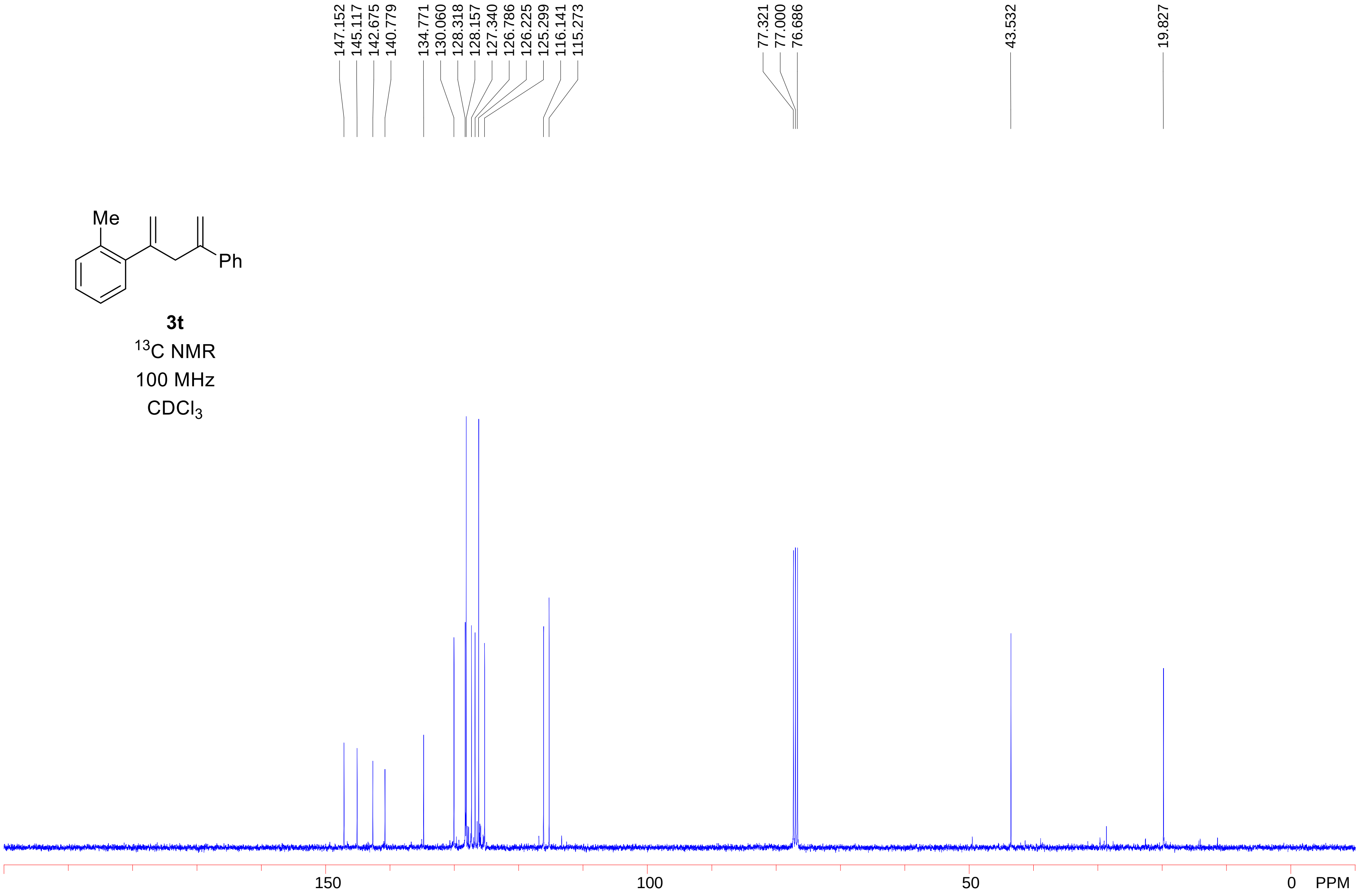

Supplementary Figure 78. ^{13}C NMR spectrum of **3t**

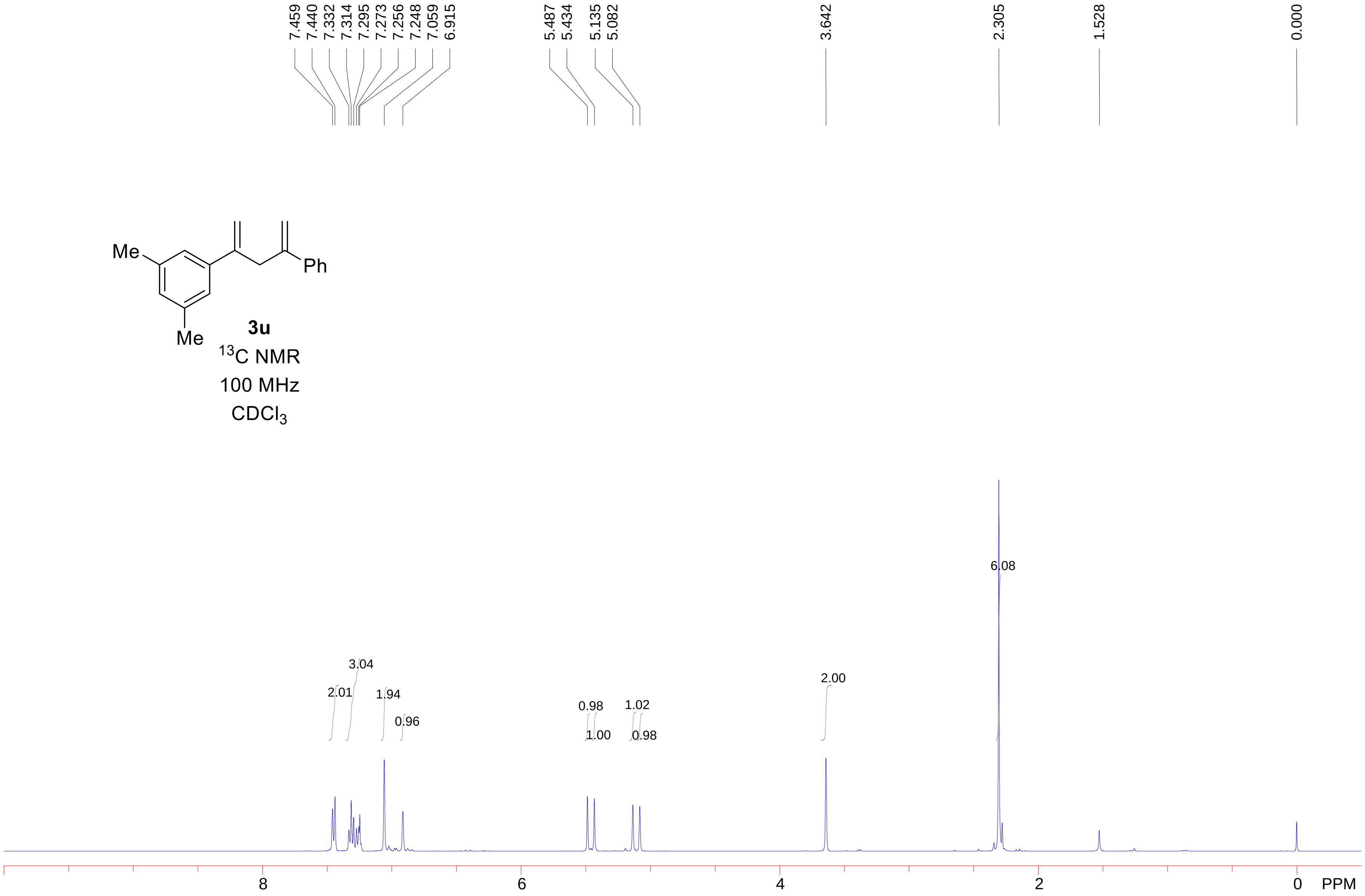

Supplementary Figure 79. ^{1}H NMR spectrum of **3u**

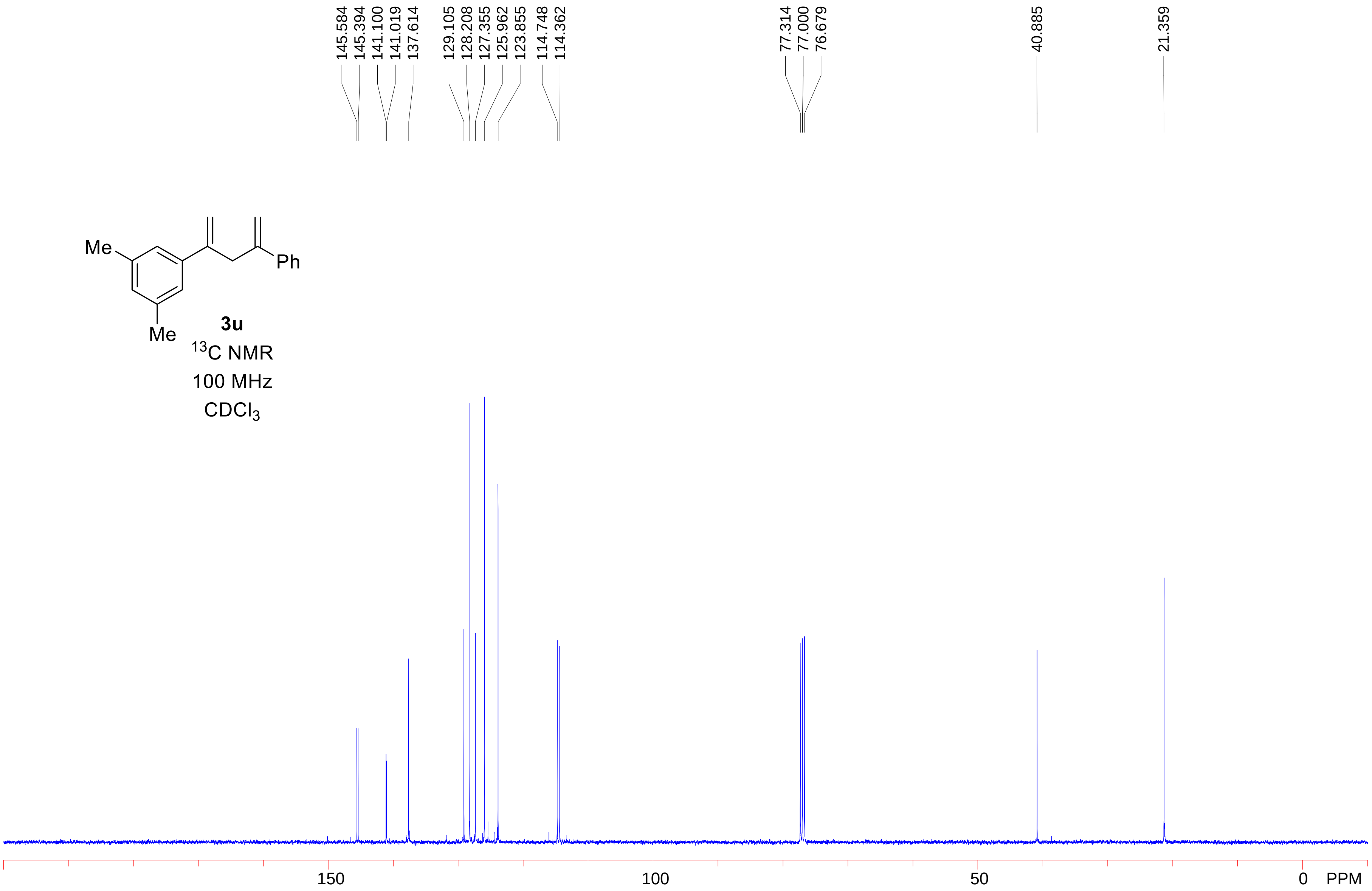

Supplementary Figure 80. ^{13}C NMR spectrum of **3u**

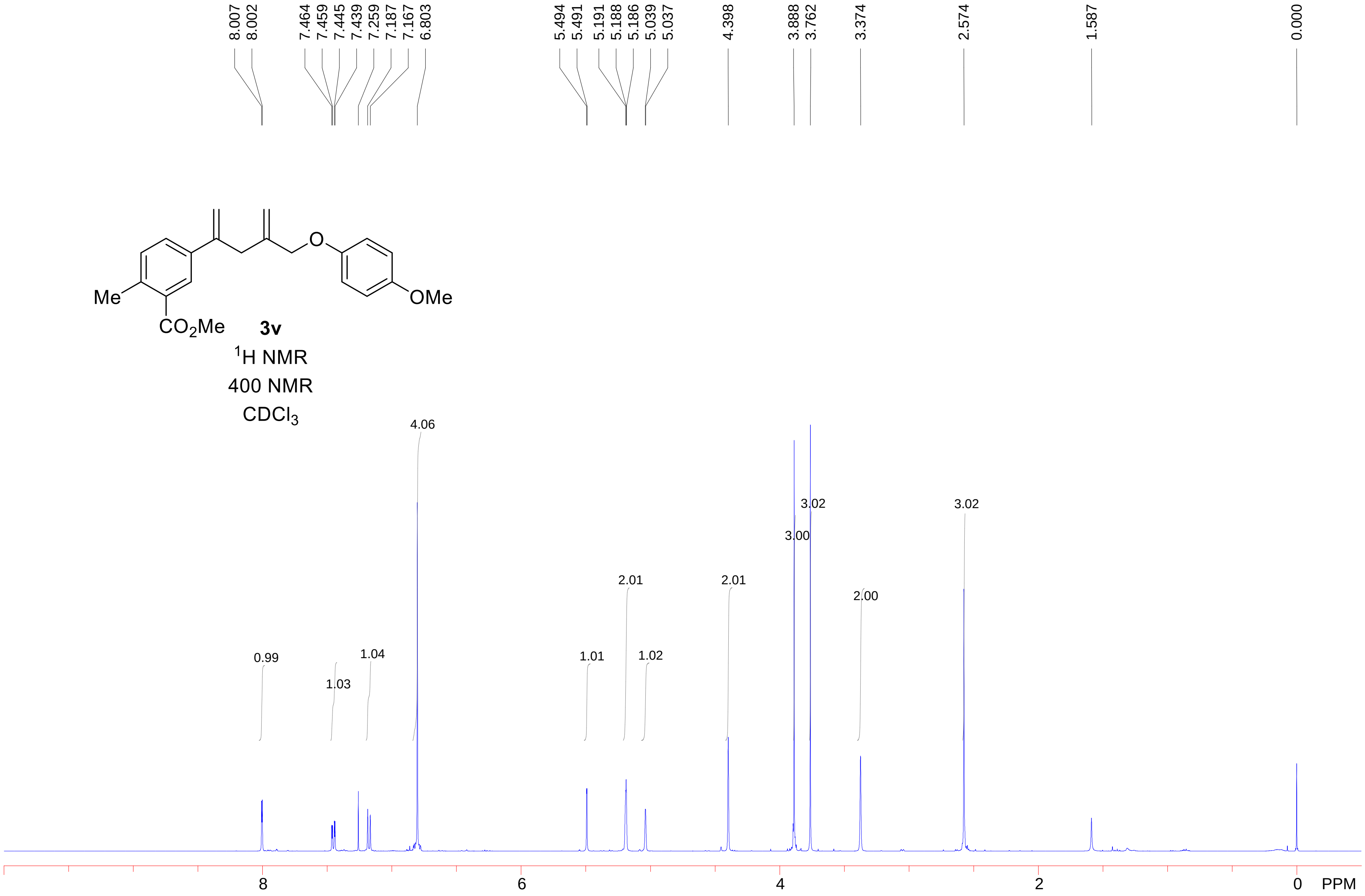

Supplementary Figure 81. ^{1}H NMR spectrum of **3v**

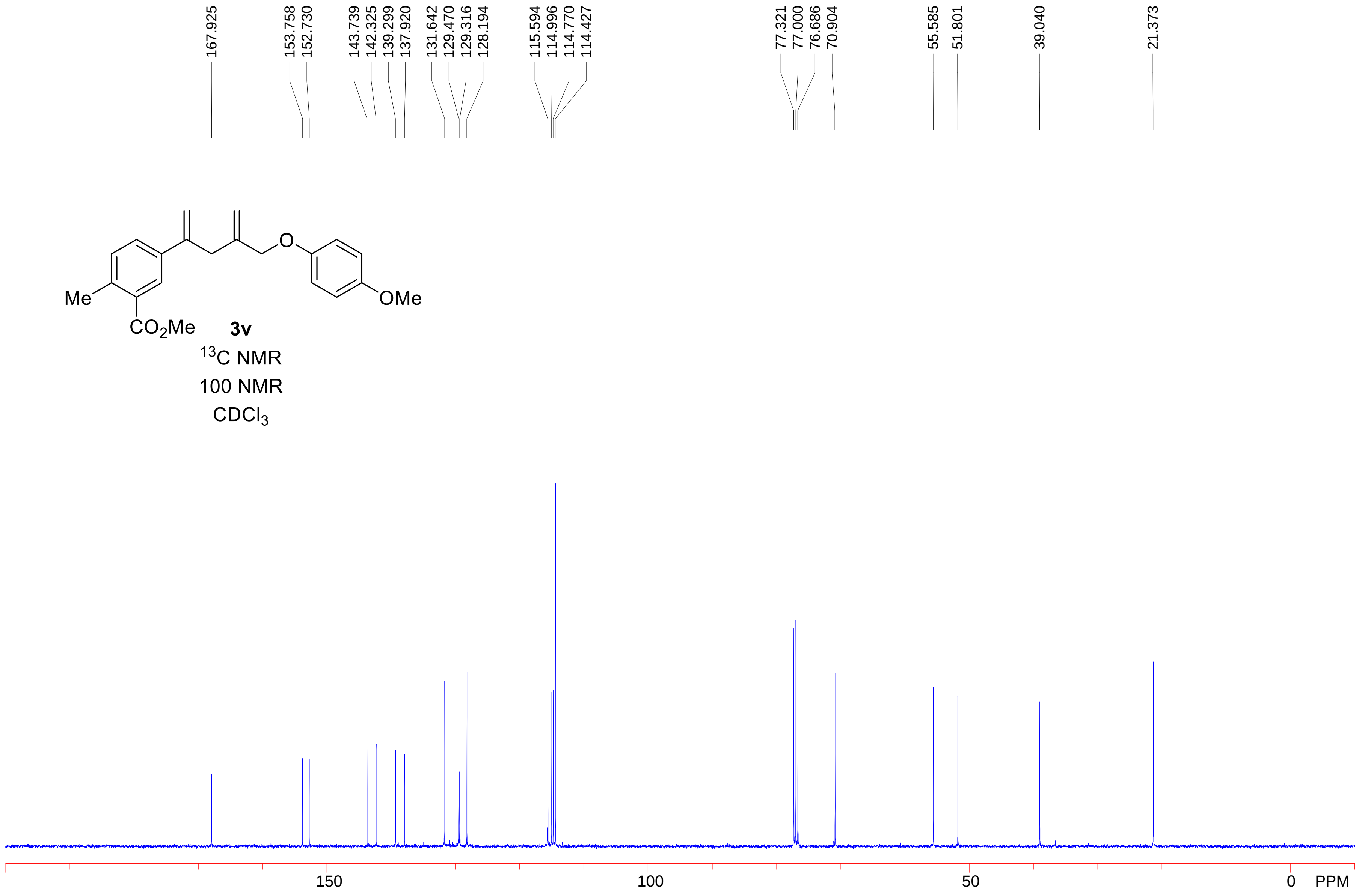

Supplementary Figure 82. ^{13}C NMR spectrum of **3v**

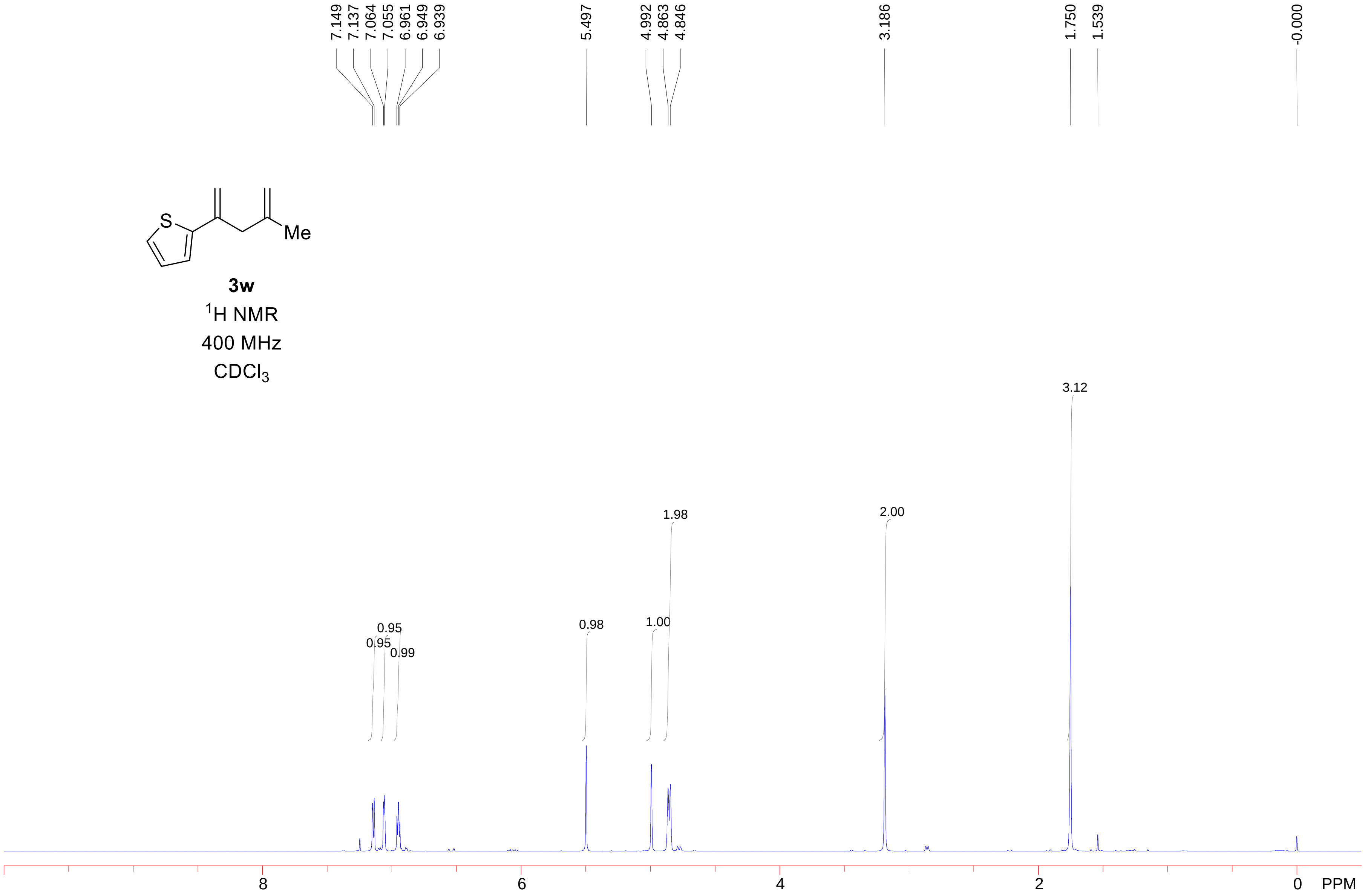

Supplementary Figure 83. ^{1}H NMR spectrum of **3w**

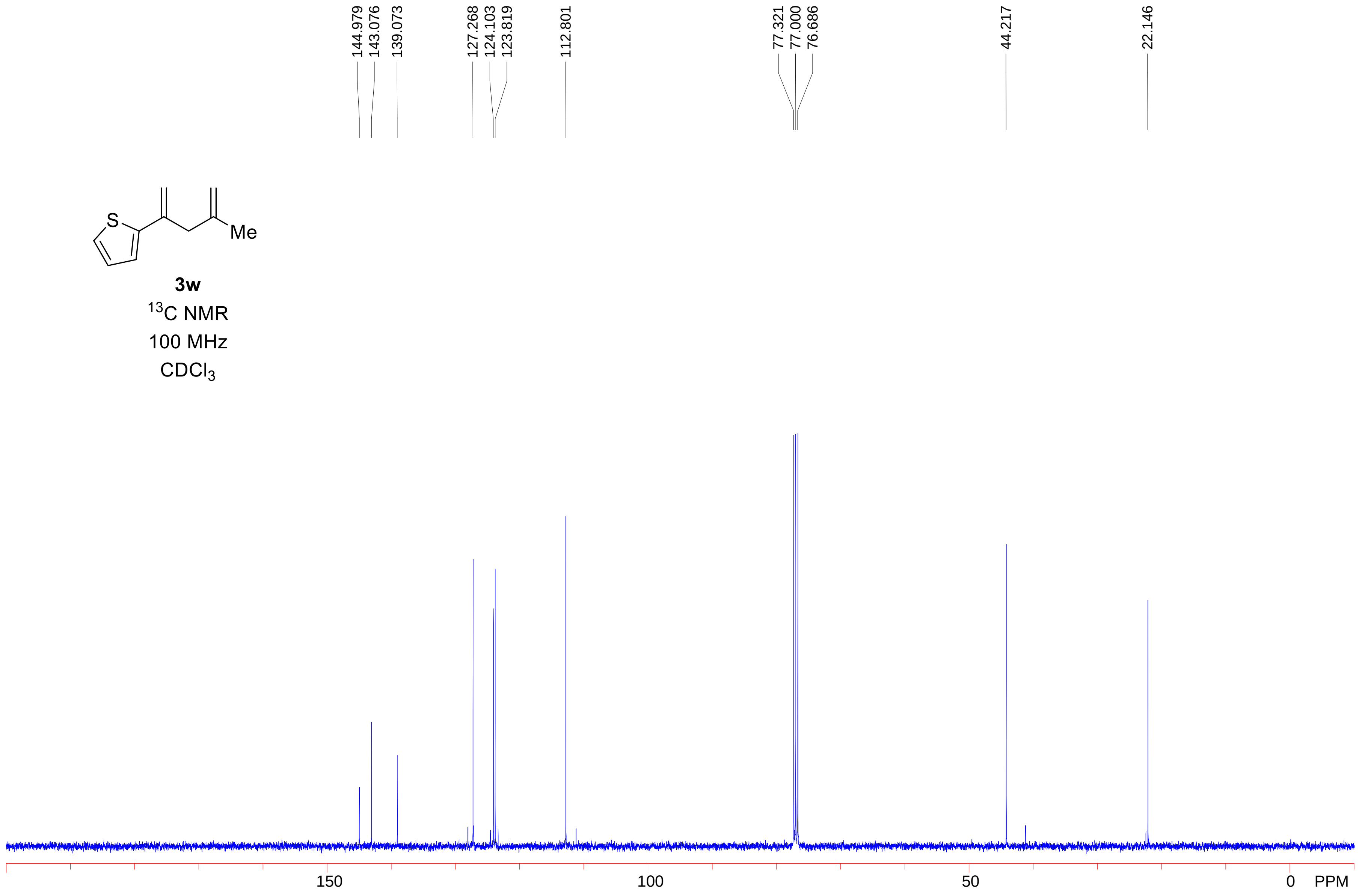

Supplementary Figure 84. ^{13}C NMR spectrum of **3w**

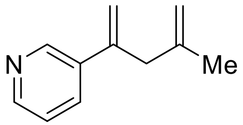

3x
^{1}H NMR
400 MHz
$CDCl_3$

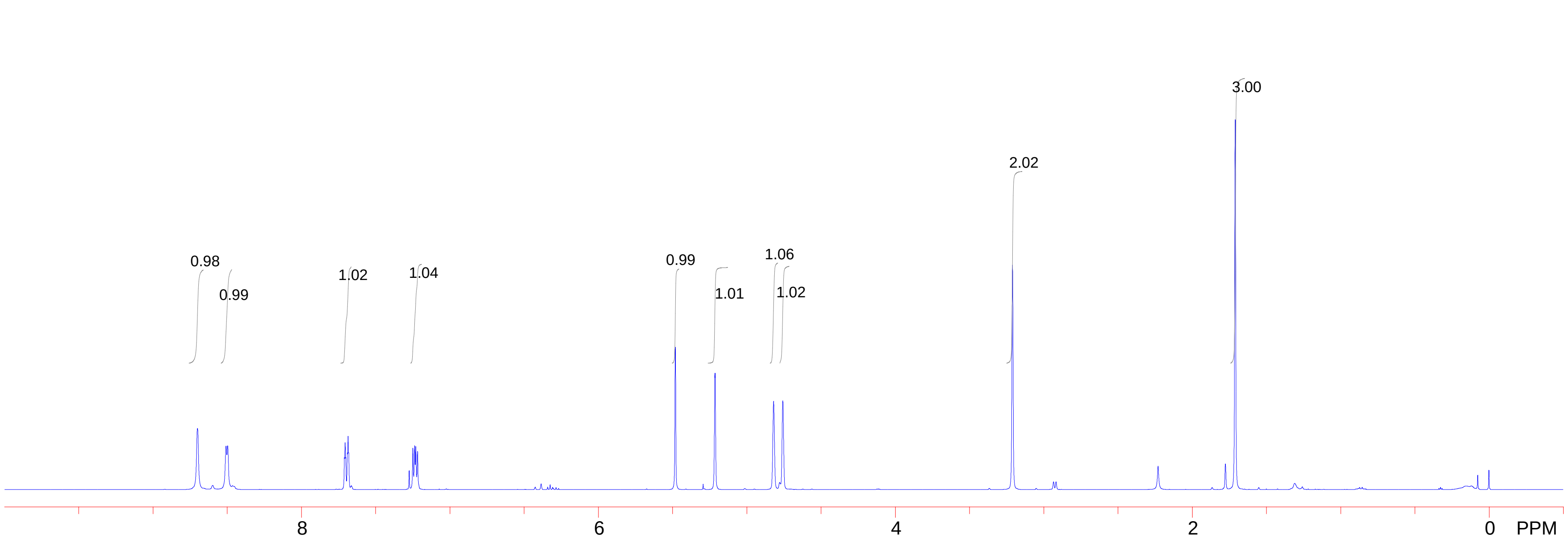

Supplementary Figure 85. ^{1}H NMR spectrum of **3x**

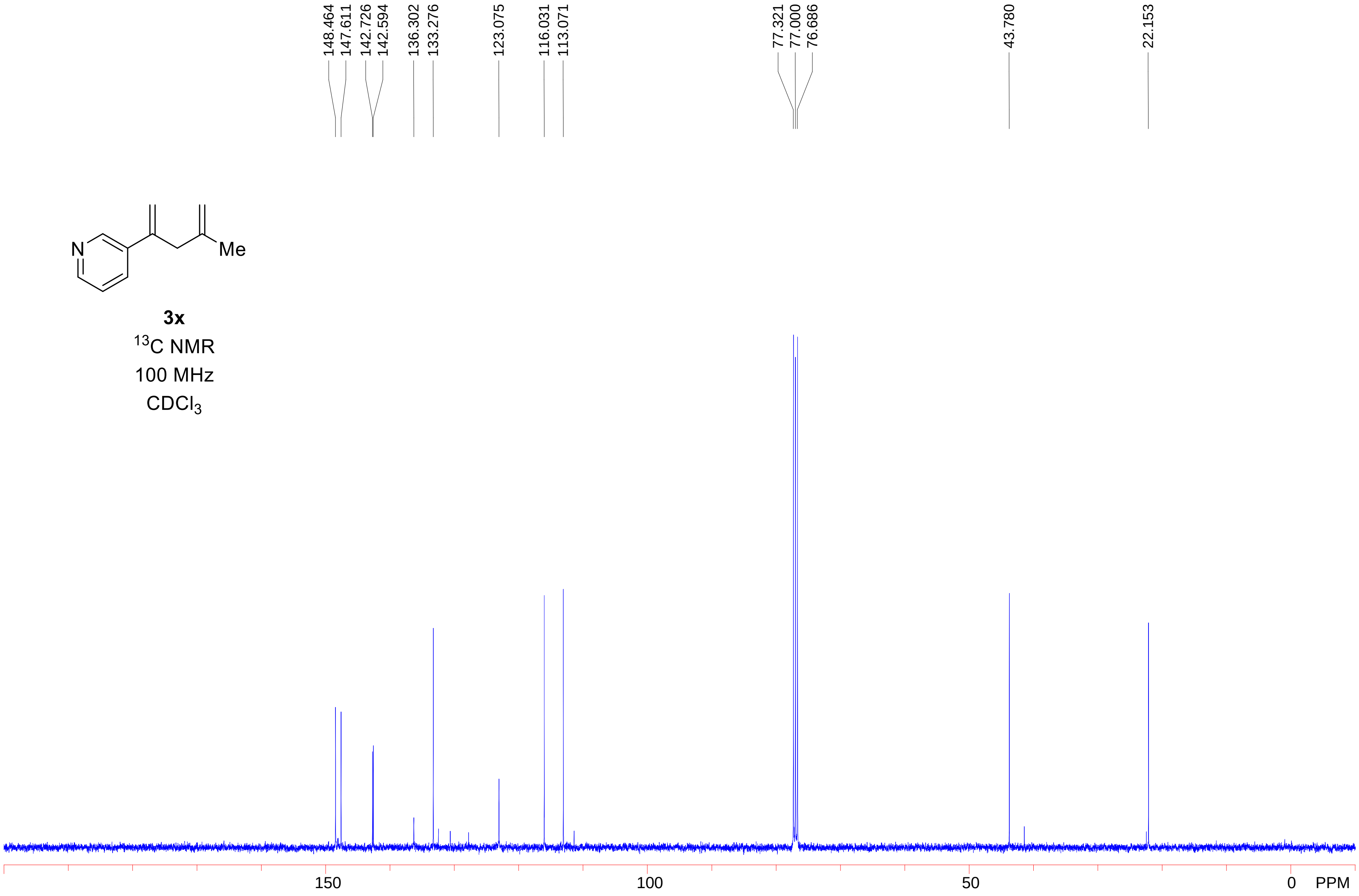

Supplementary Figure 86. ^{13}C NMR spectrum of **3x**

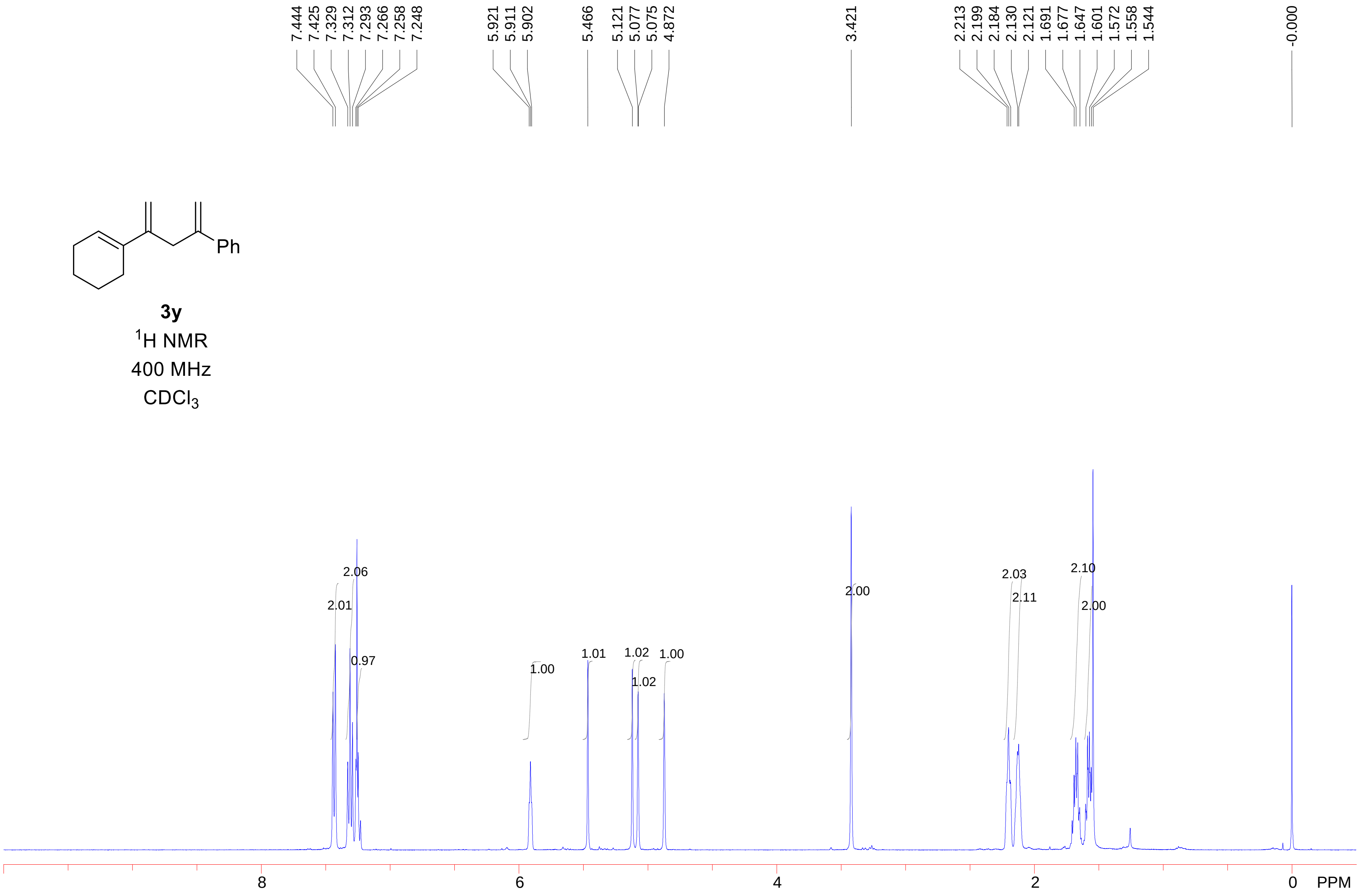

Supplementary Figure 87. ^{1}H NMR spectrum of **3y**

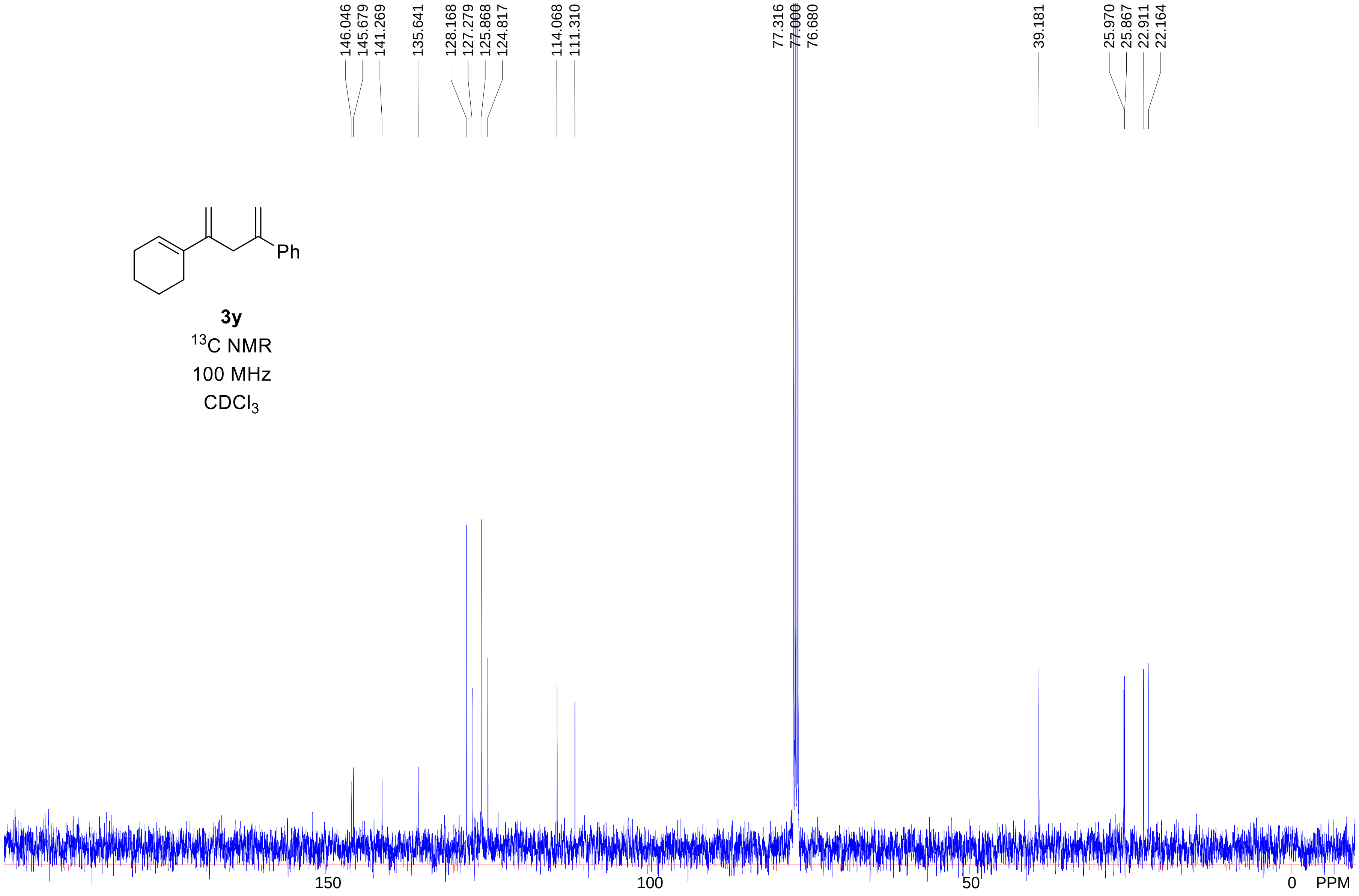

Supplementary Figure 88. ^{13}C NMR spectrum of **3y**

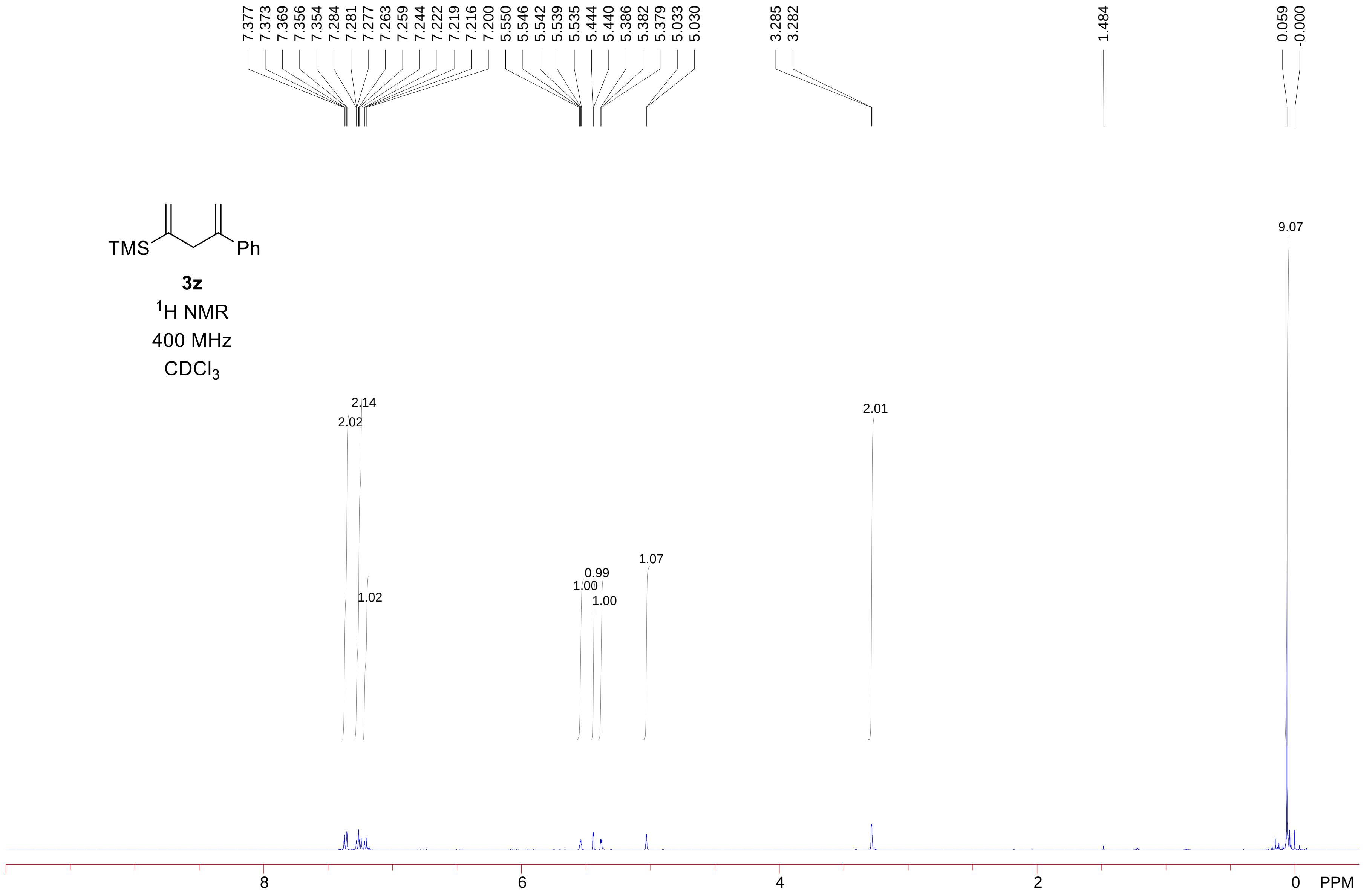

Supplementary Figure 89. ^{1}H NMR spectrum of **3z**

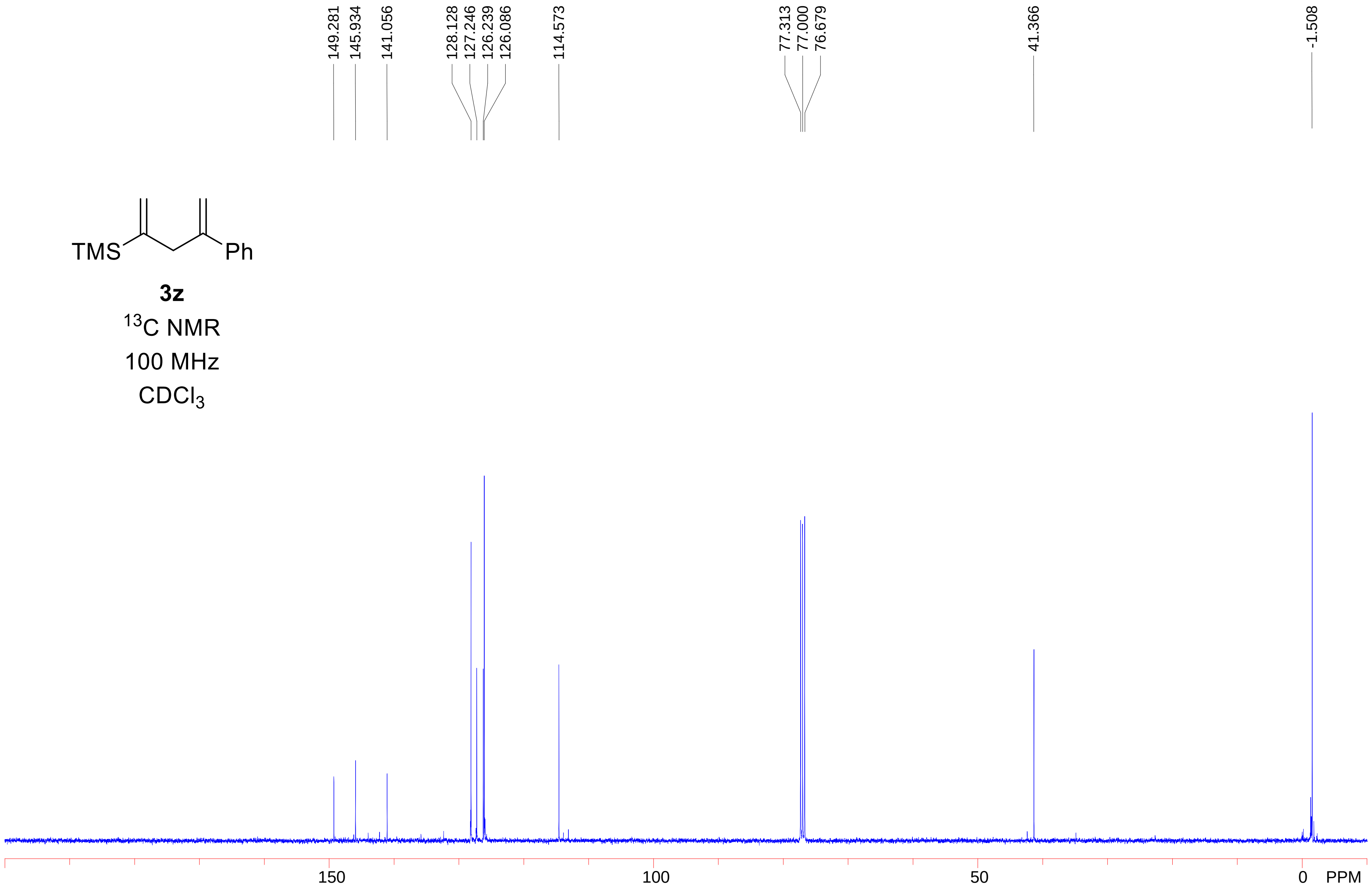

Supplementary Figure 90. ^{13}C NMR spectrum of **3z**

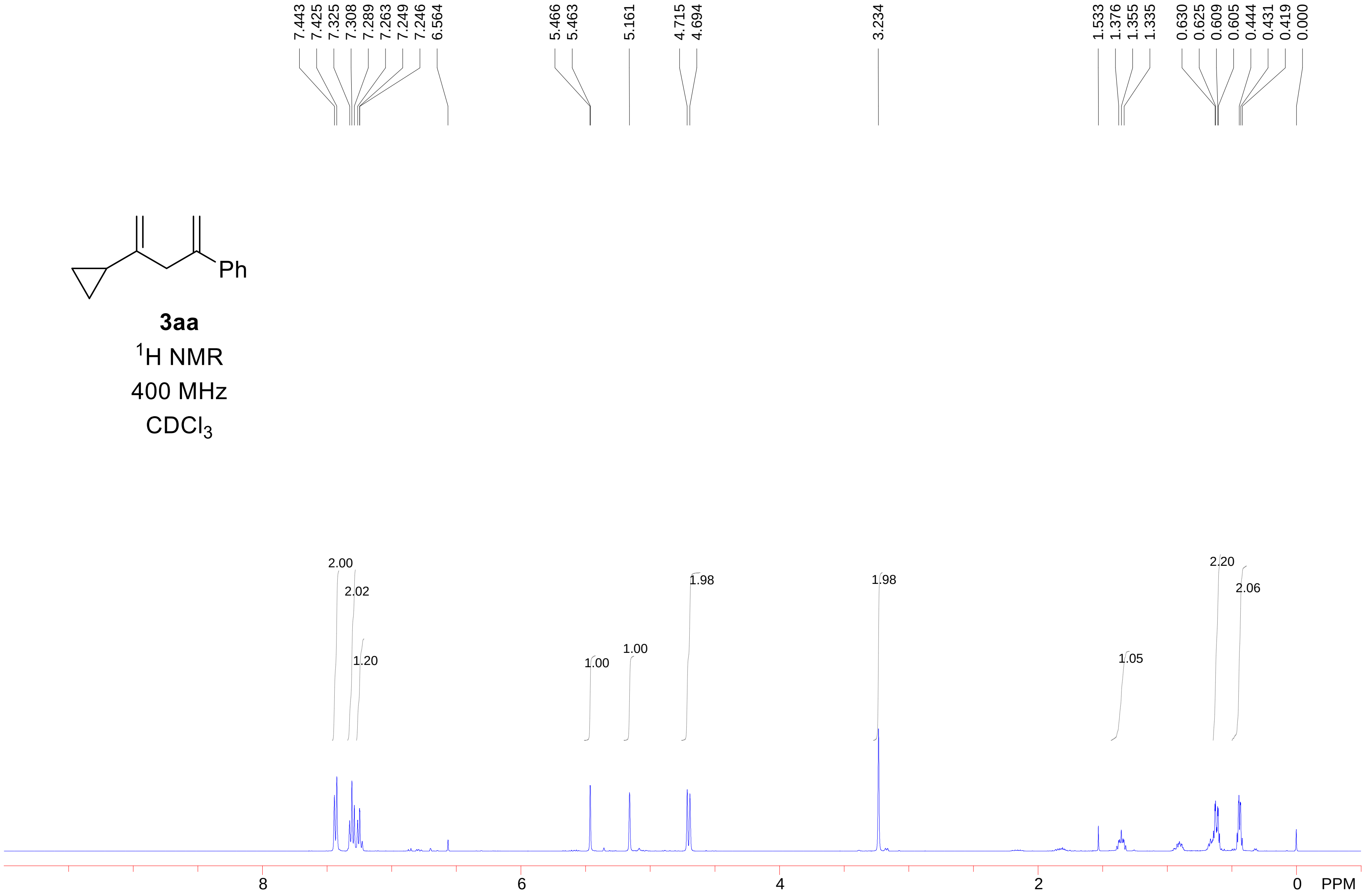

Supplementary Figure 91. ^{1}H NMR spectrum of **3aa**

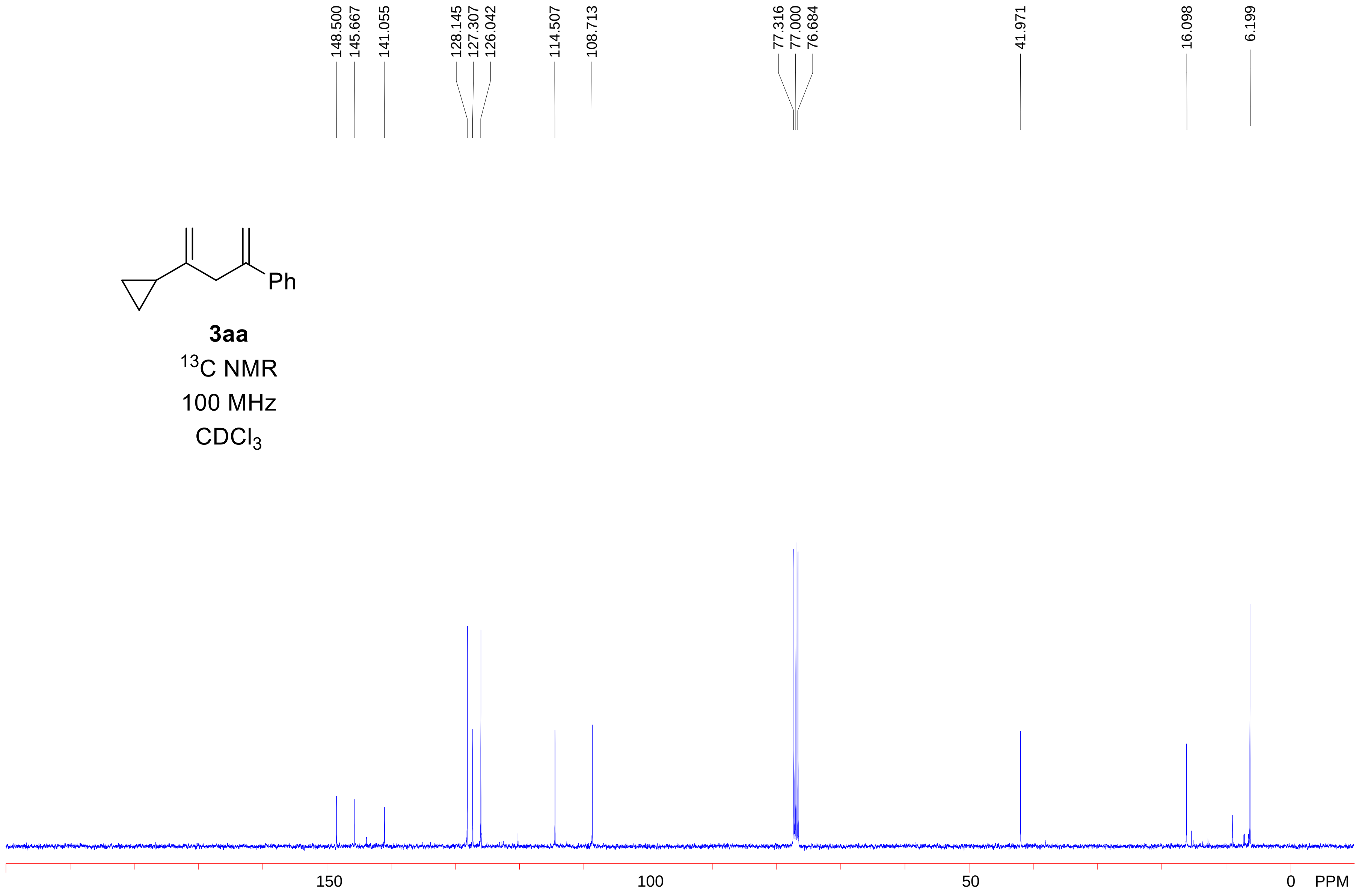

Supplementary Figure 92. ^{13}C NMR spectrum of **3aa**

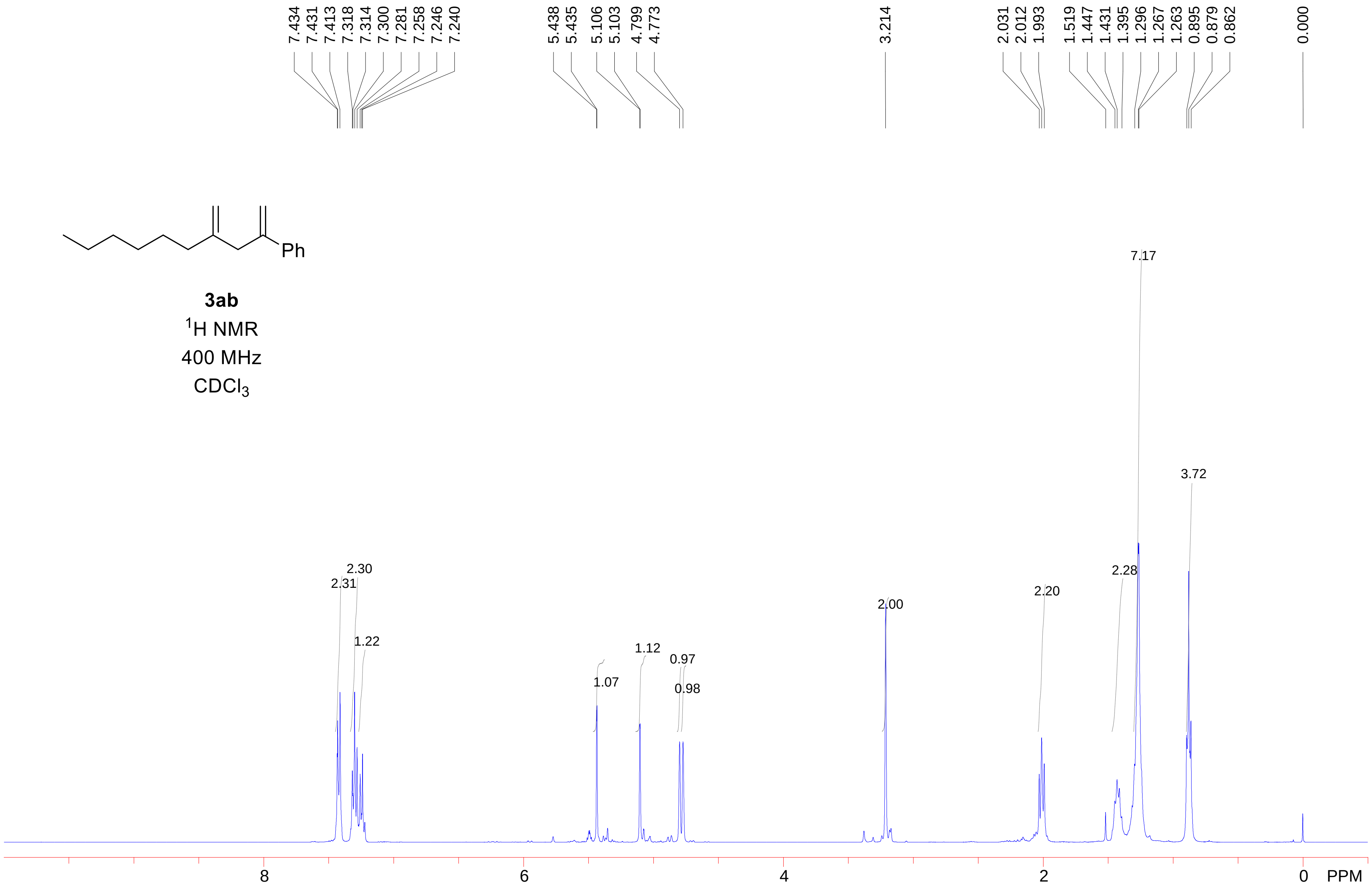

Supplementary Figure 93. ^{1}H NMR spectrum of **3ab**

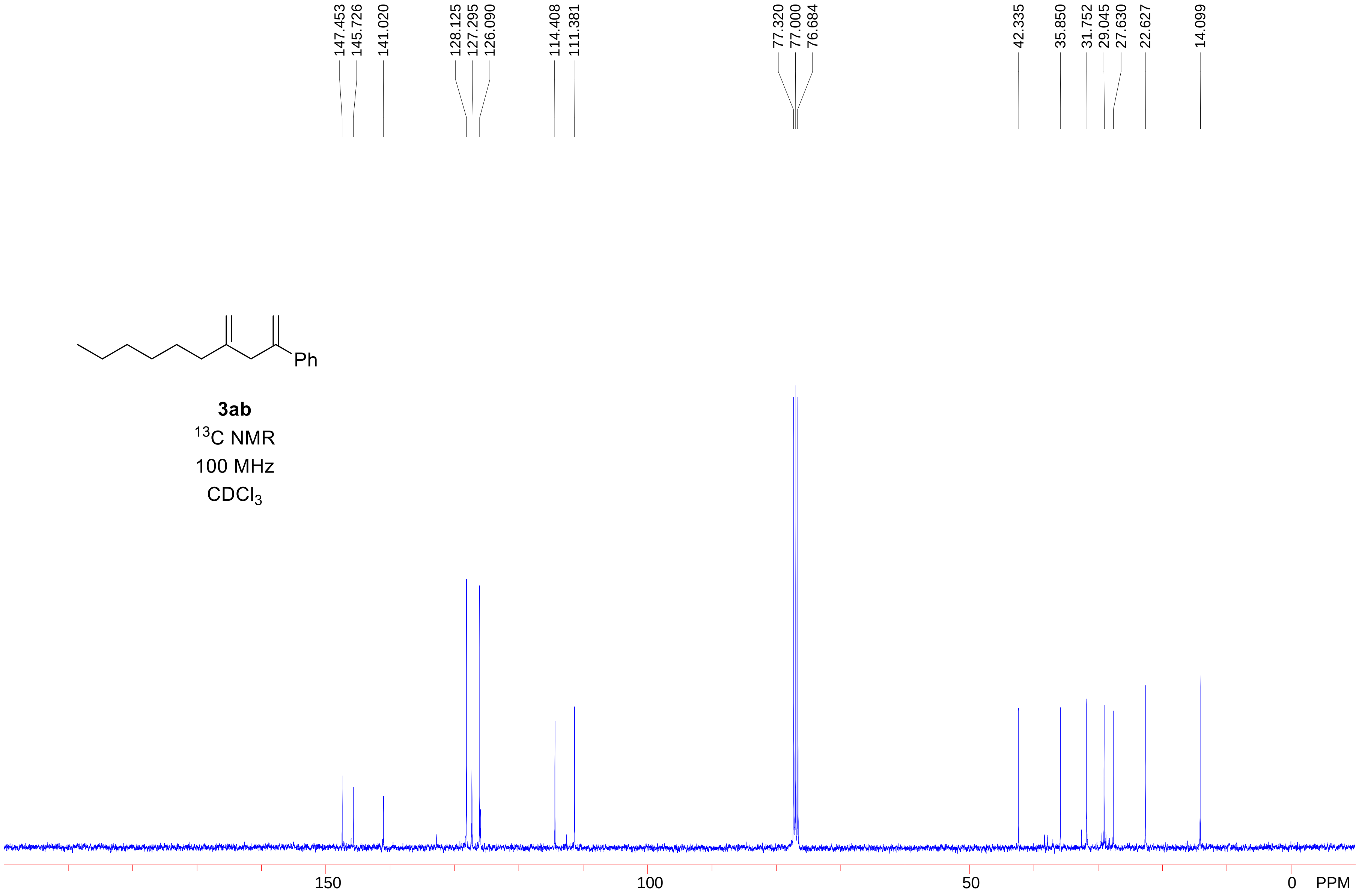

Supplementary Figure 94. ^{13}C NMR spectrum of **3ab**

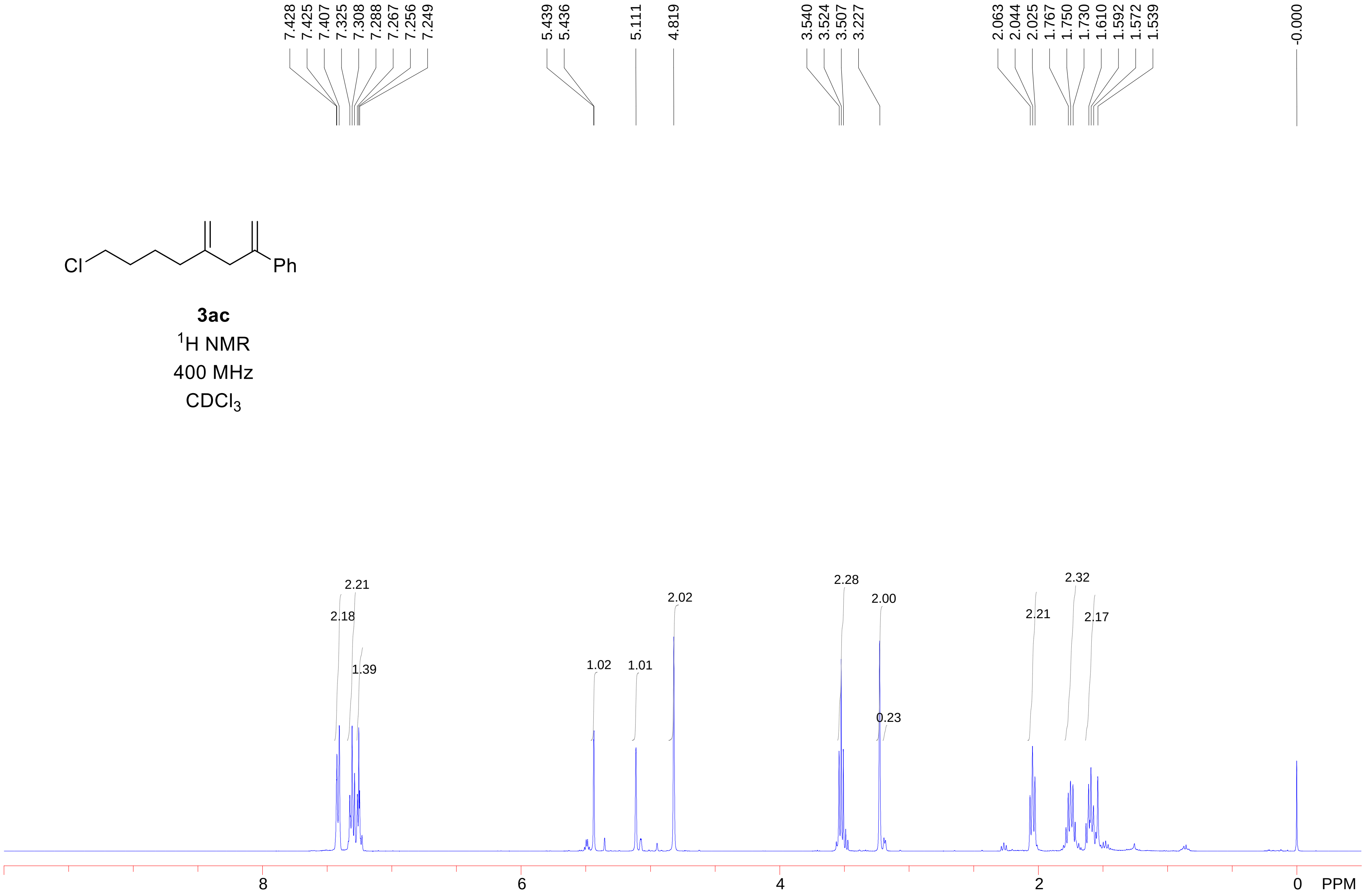

Supplementary Figure 95. ^{1}H NMR spectrum of **3ac**

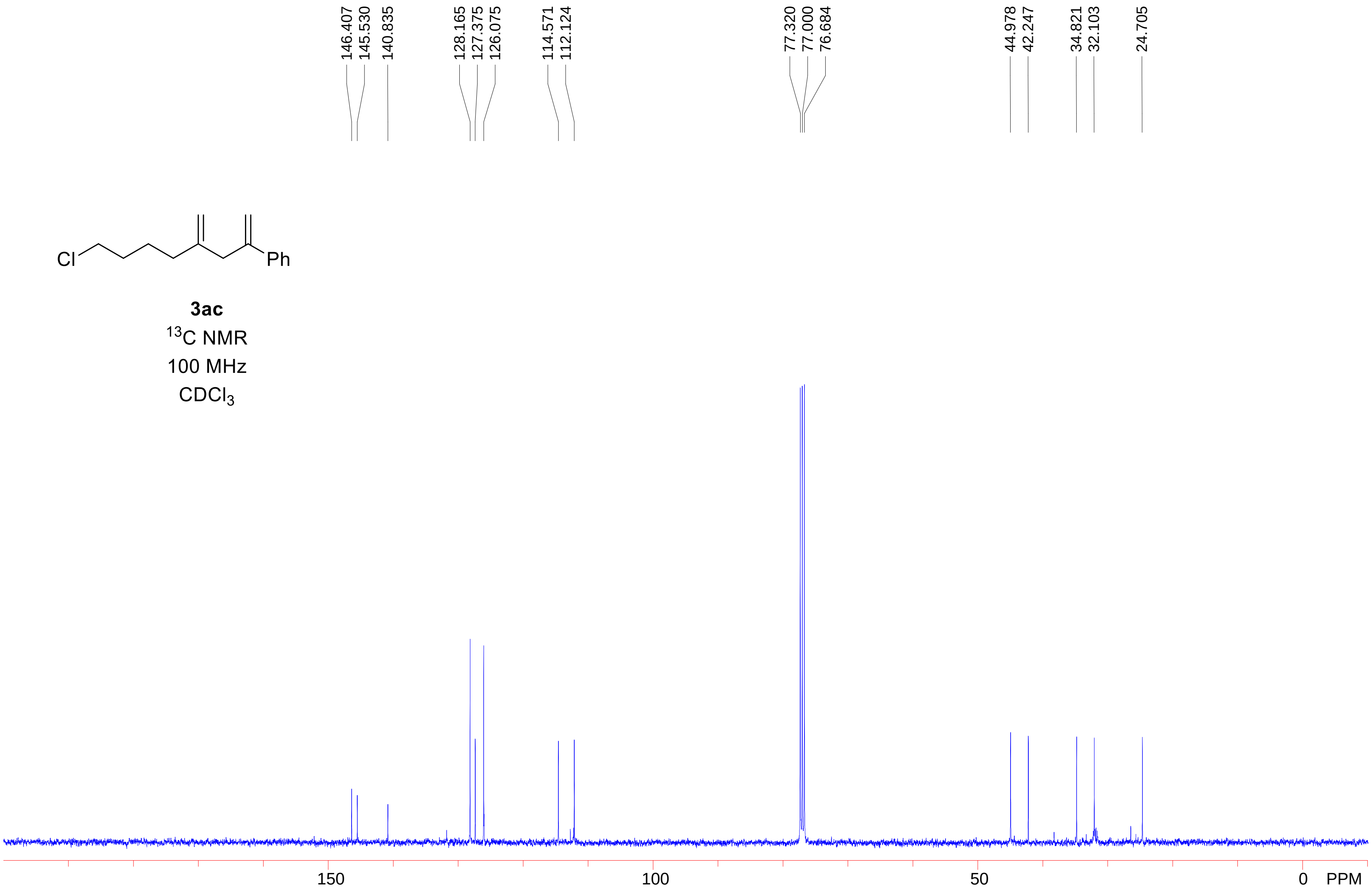

Supplementary Figure 96. ^{13}C NMR spectrum of **3ac**

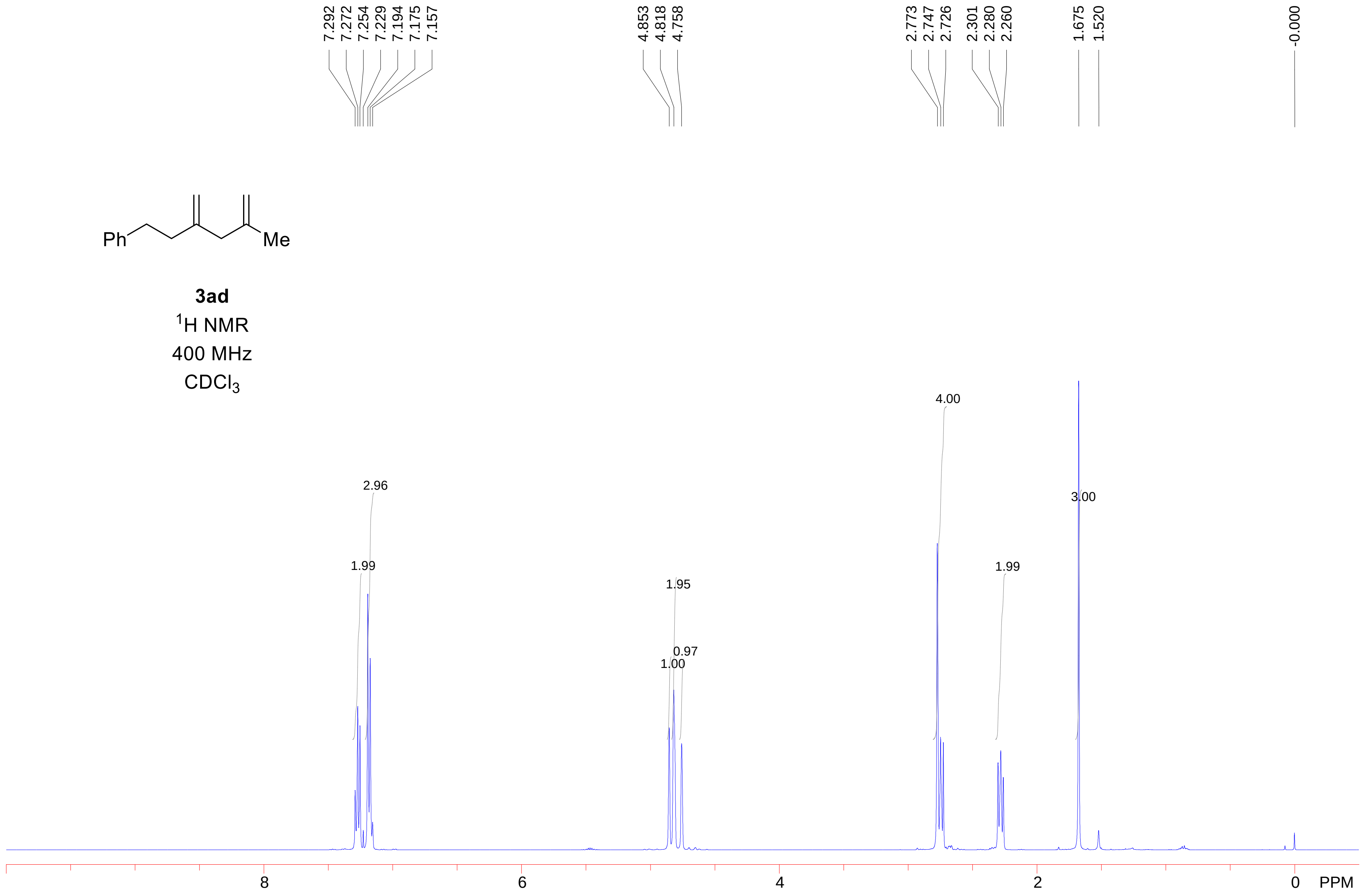

Supplementary Figure 97. ^{1}H NMR spectrum of **3ad**

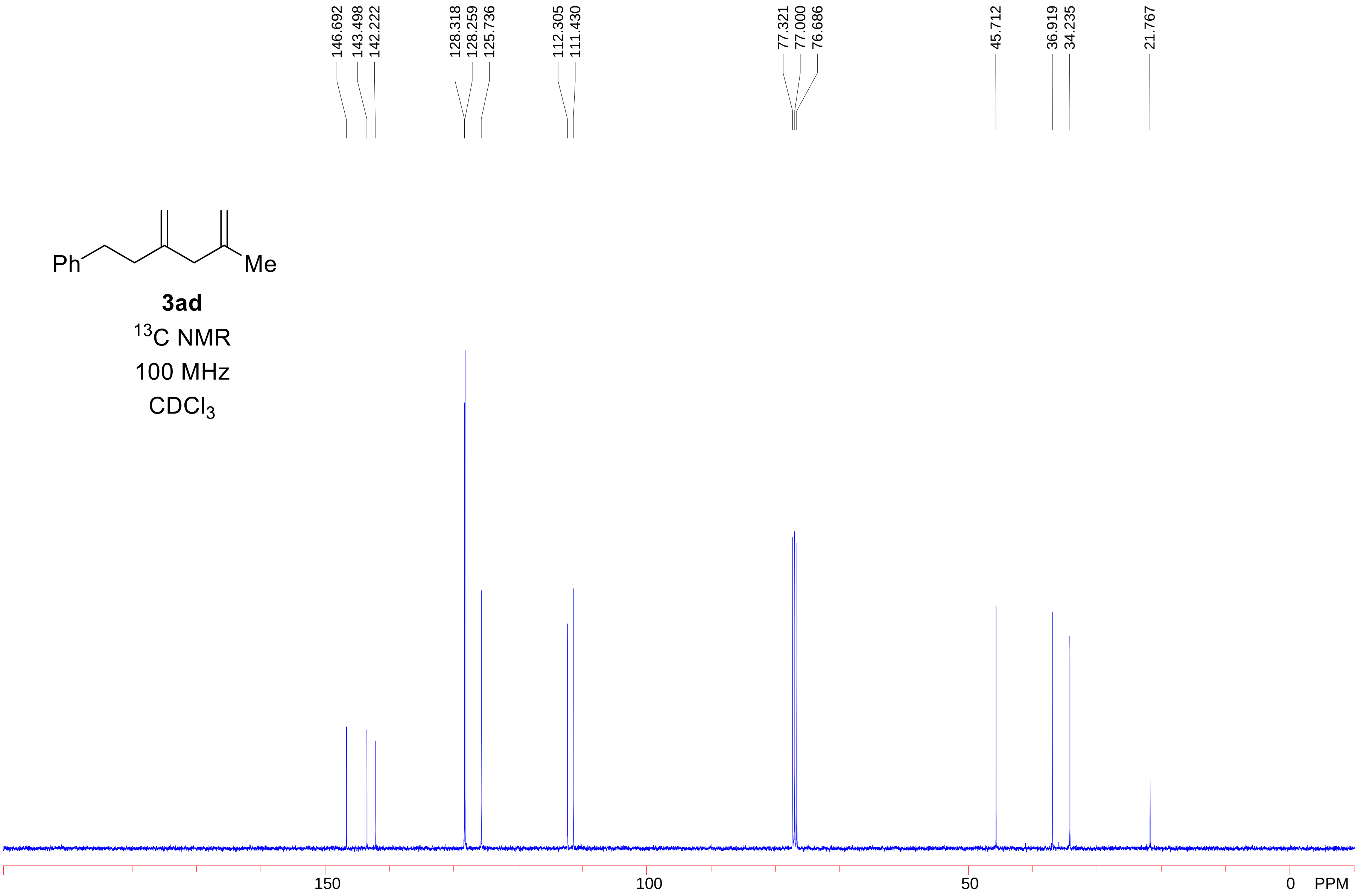

Supplementary Figure 98. ^{13}C NMR spectrum of **3ad**

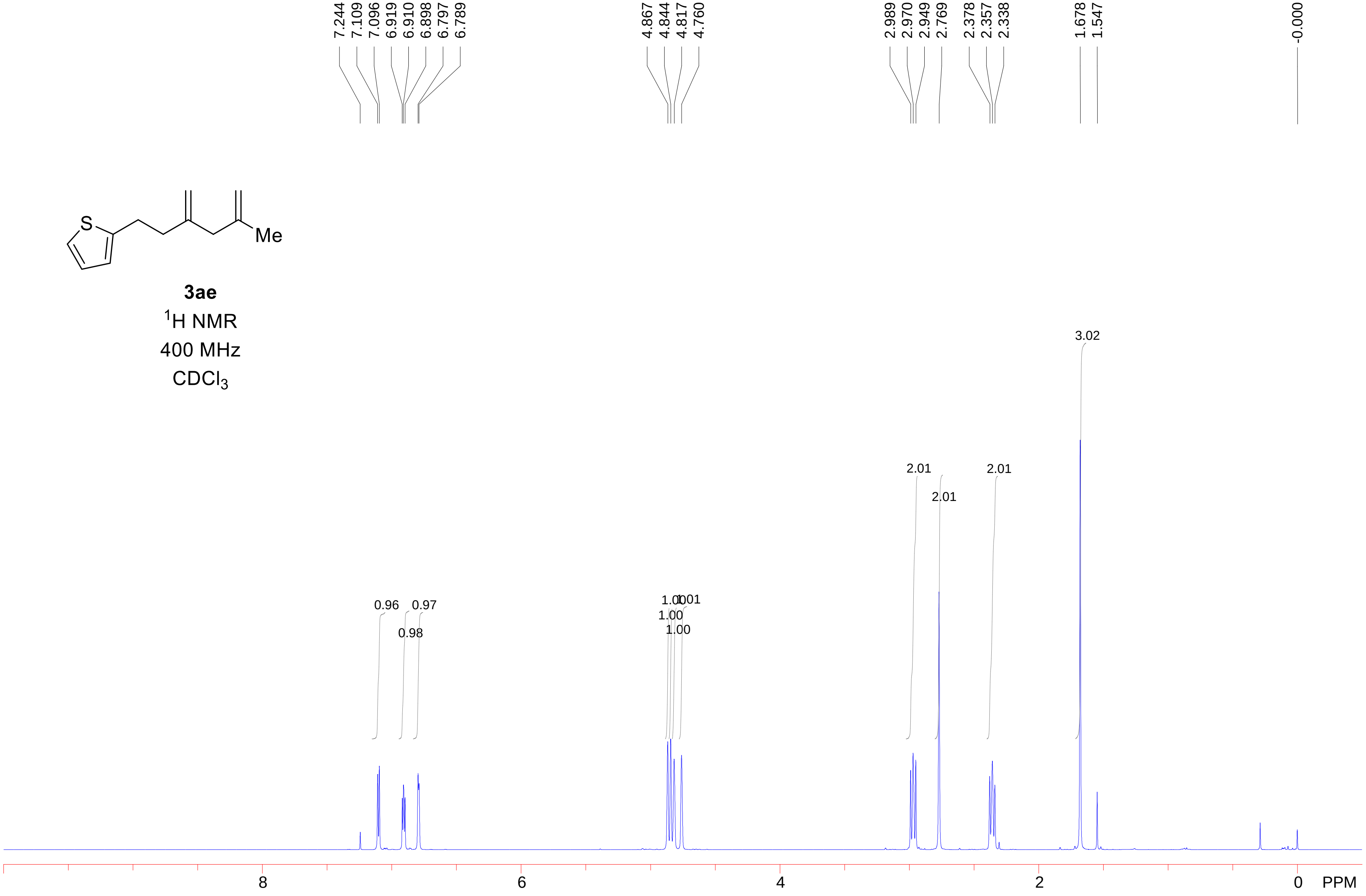

Supplementary Figure 99. ^{1}H NMR spectrum of **3ae**

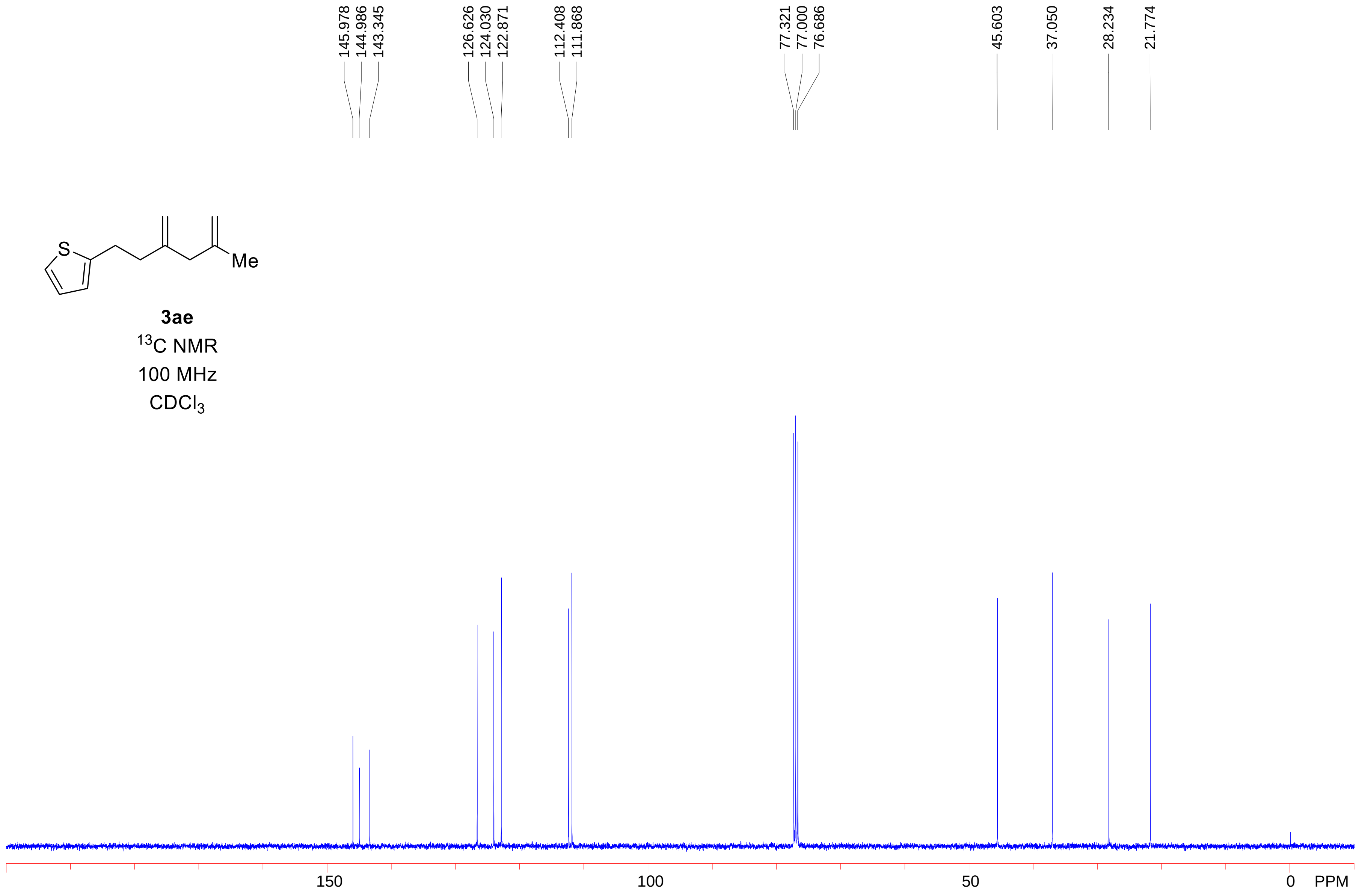

Supplementary Figure 100. ^{13}C NMR spectrum of **3ae**

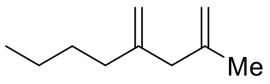

3af
^{1}HNMR
100 MHz
$CDCl_3$

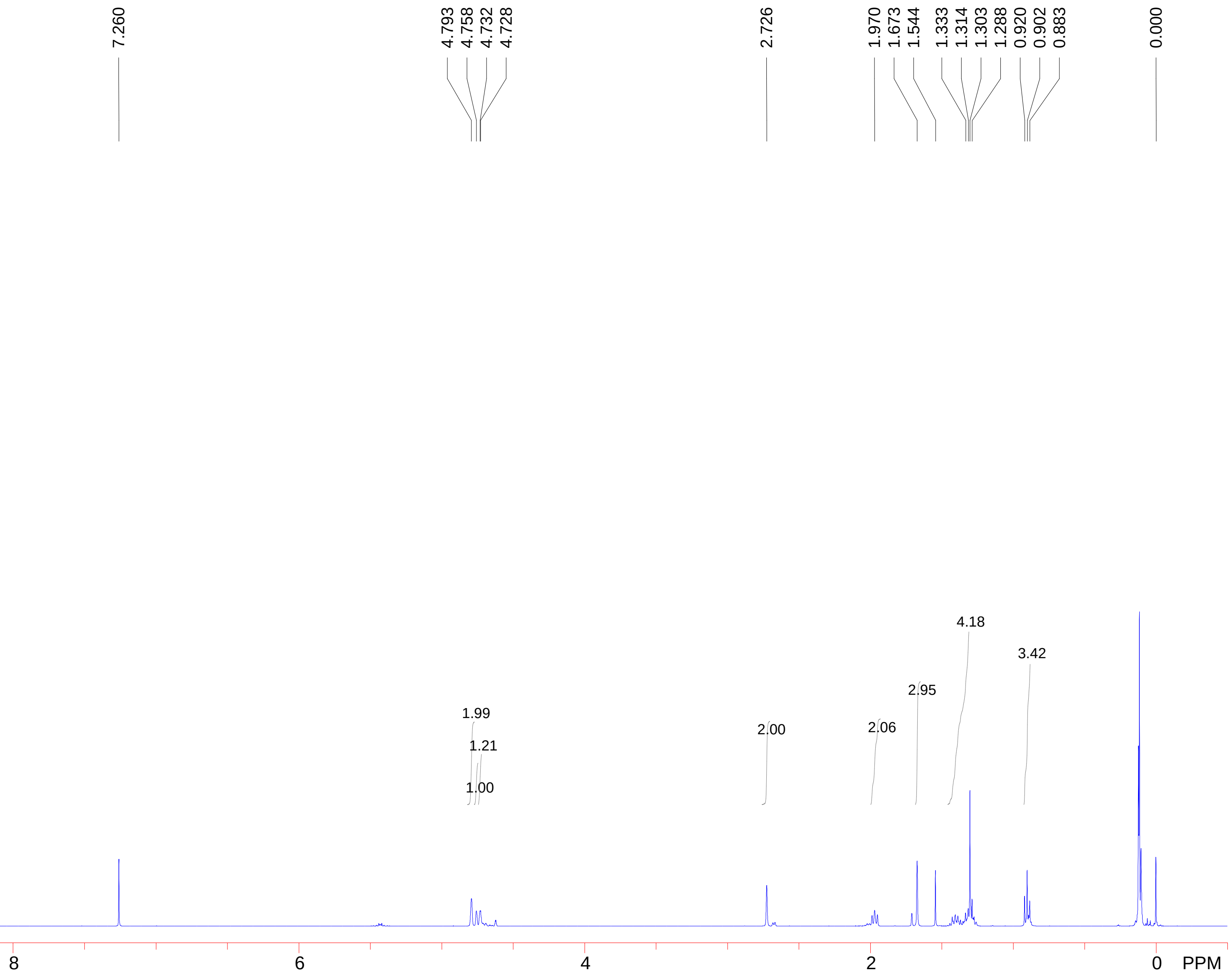

Supplementary Figure 101. ^{1}H NMR spectrum of **3af**

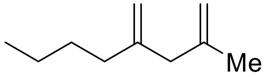

3af
^{13}C NMR
100 MHz
$CDCl_3$

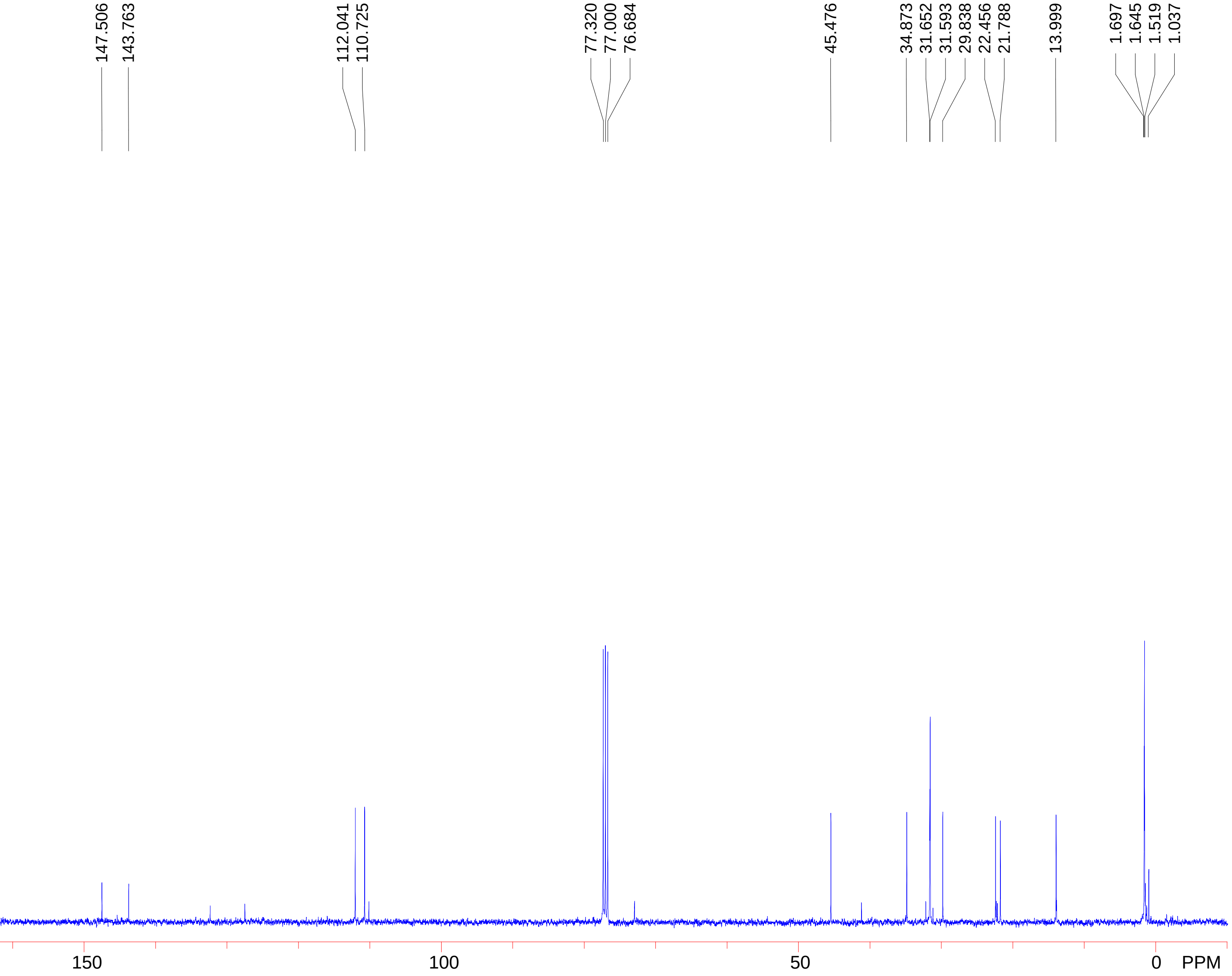

Supplementary Figure 102. ^{13}C NMR spectrum of **3af**

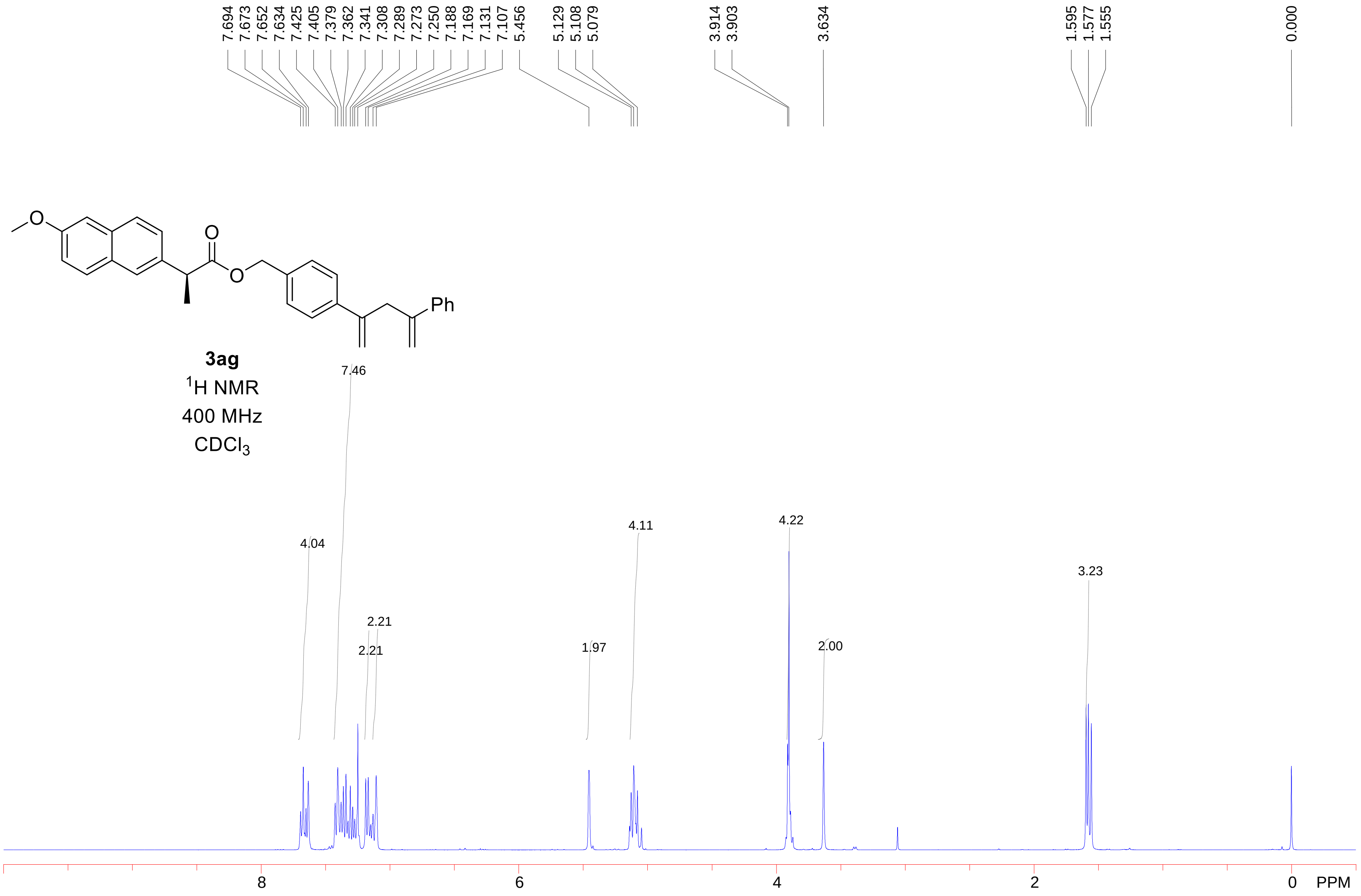

Supplementary Figure 103. ^{1}H NMR spectrum of **3ag**

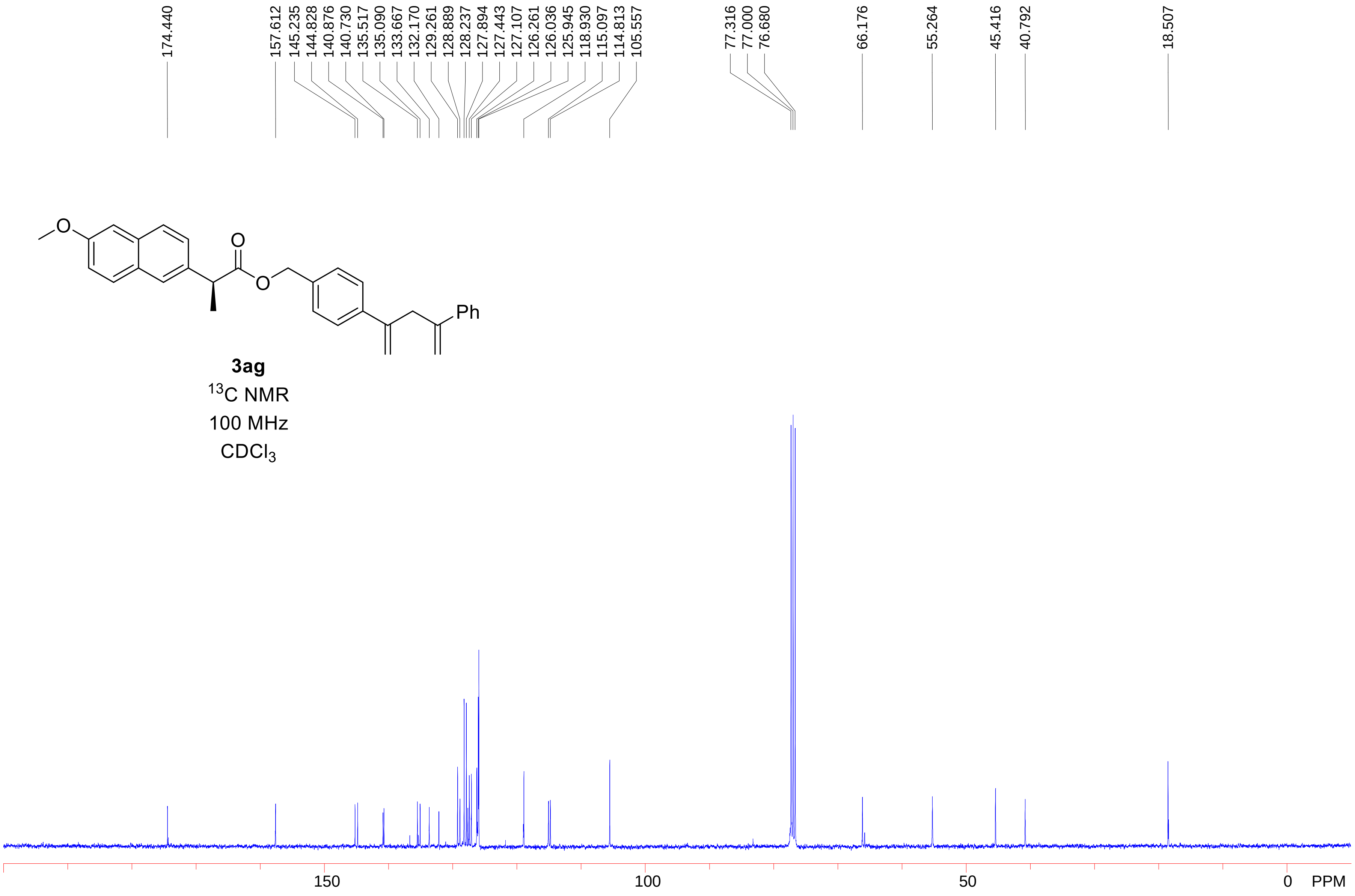

Supplementary Figure 104. ^{13}C NMR spectrum of **3ag**

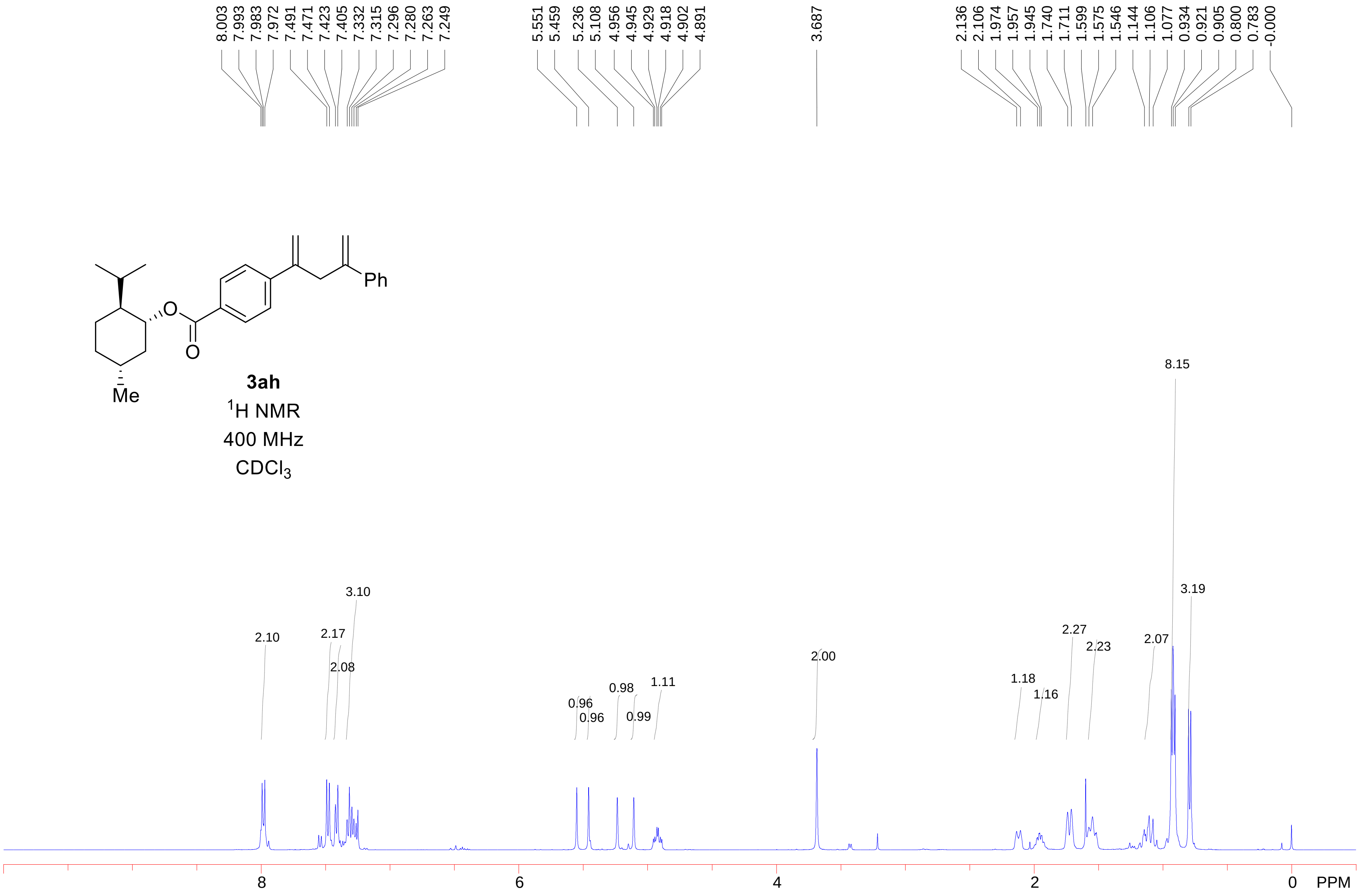

Supplementary Figure 105. ^{1}H NMR spectrum of **3ah**

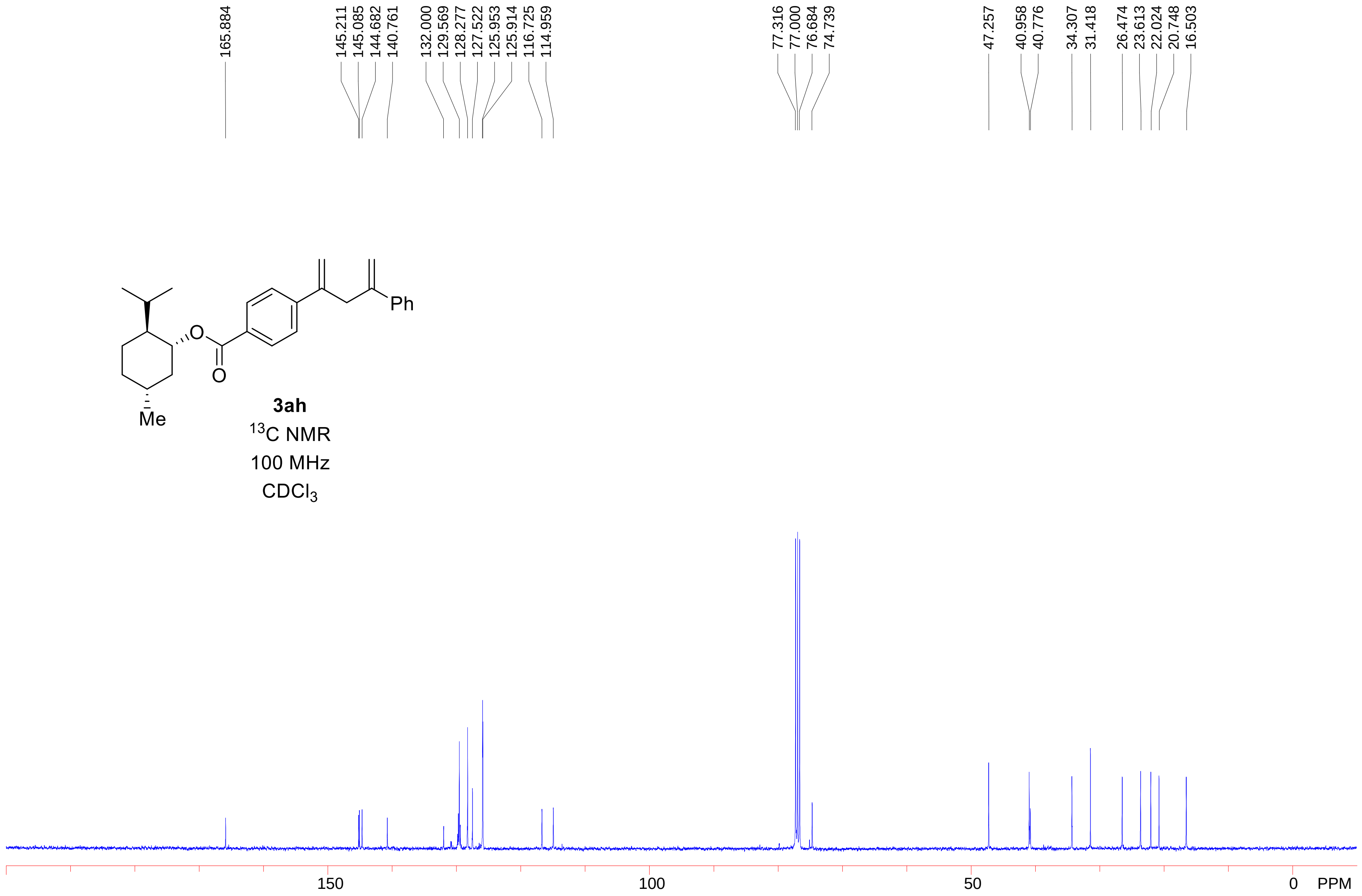

Supplementary Figure 106. ^{13}C NMR spectrum of **3ah**

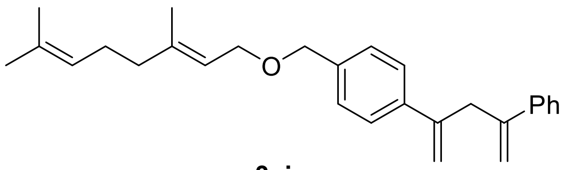

3ai

^{1}H NMR

400 MHz

$CDCl_3$

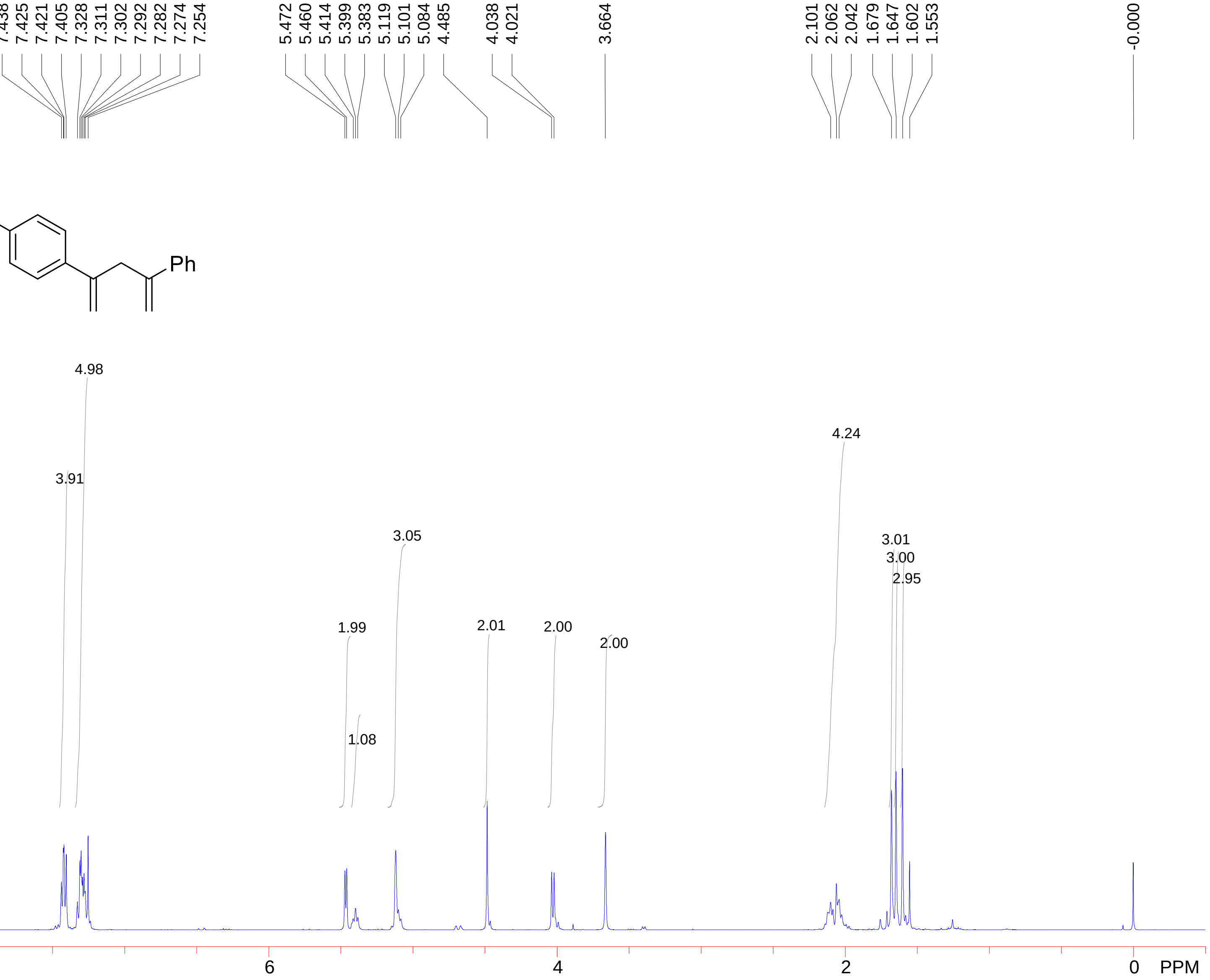

Supplementary Figure 107. ^{1}H NMR spectrum of **3ai**

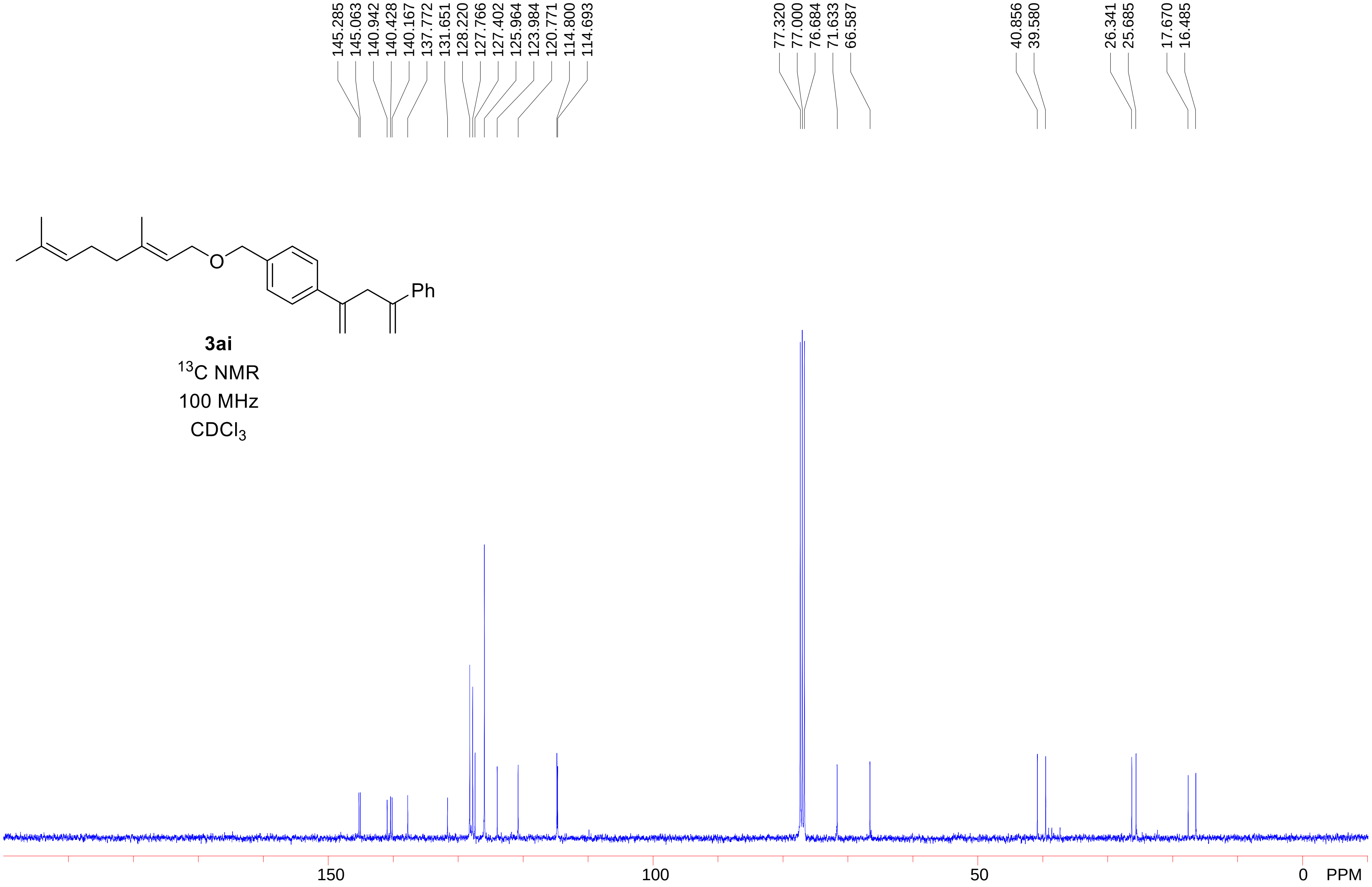

Supplementary Figure 108. ^{13}C NMR spectrum of **3ai**

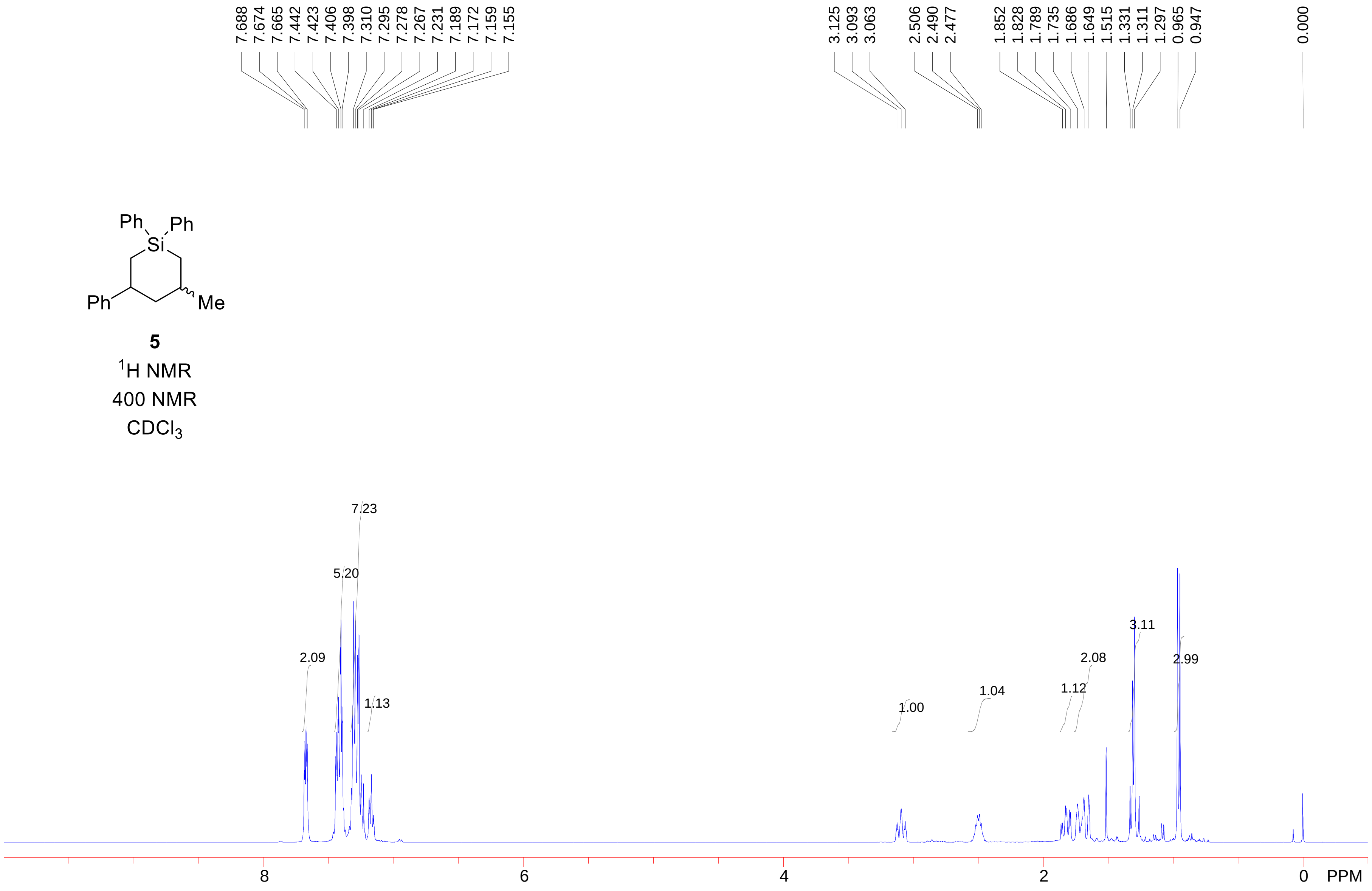

Supplementary Figure 109. ^{1}H NMR spectrum of **5**

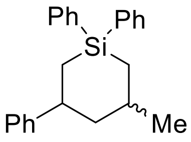

5

^{13}C NMR

100 NMR

$CDCl_3$

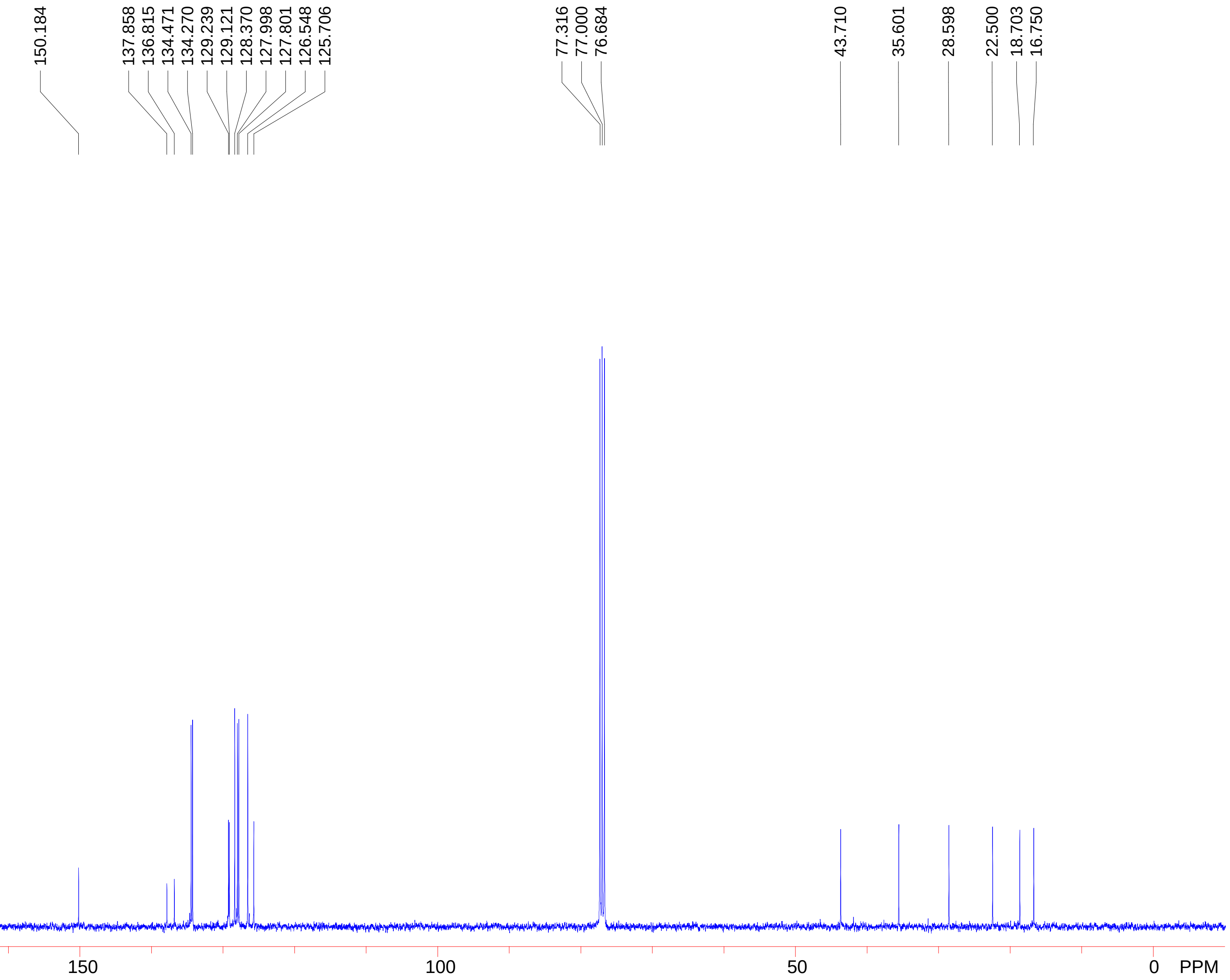

Supplementary Figure 110. ^{13}C NMR spectrum of **L2**

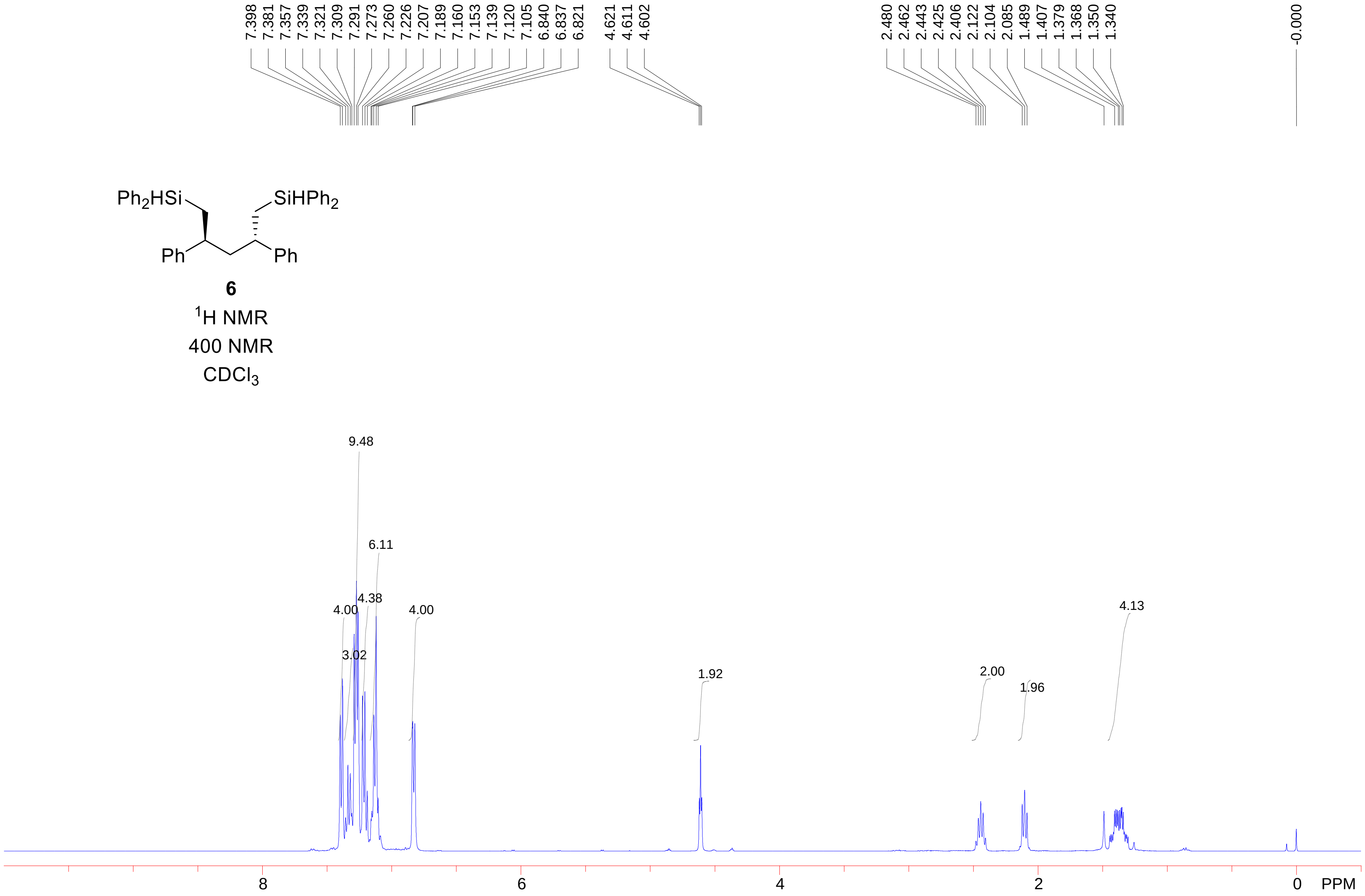

Supplementary Figure 111. ^{1}H NMR spectrum of **6**

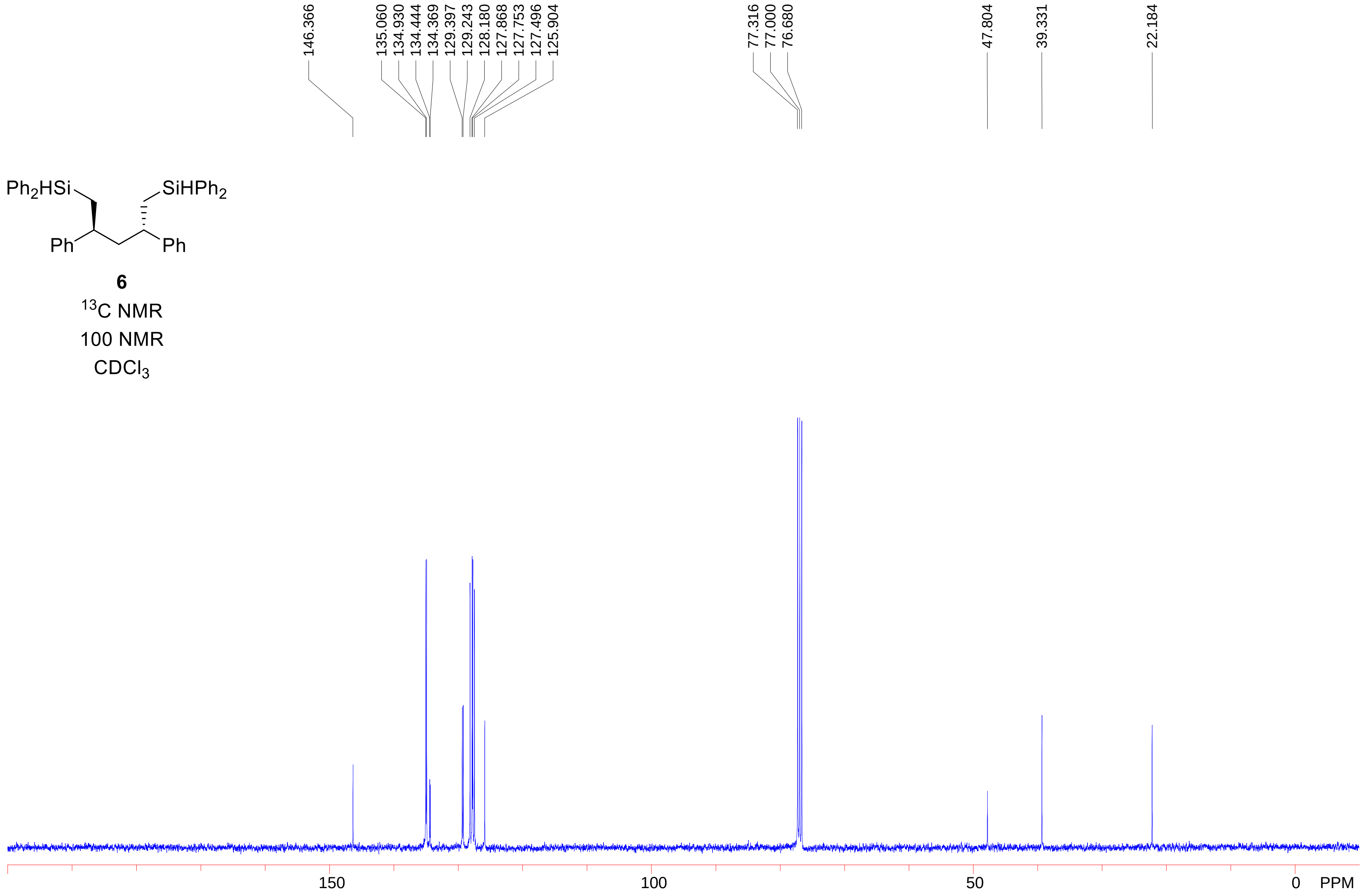

Supplementary Figure 112. ^{13}C NMR spectrum of **6**

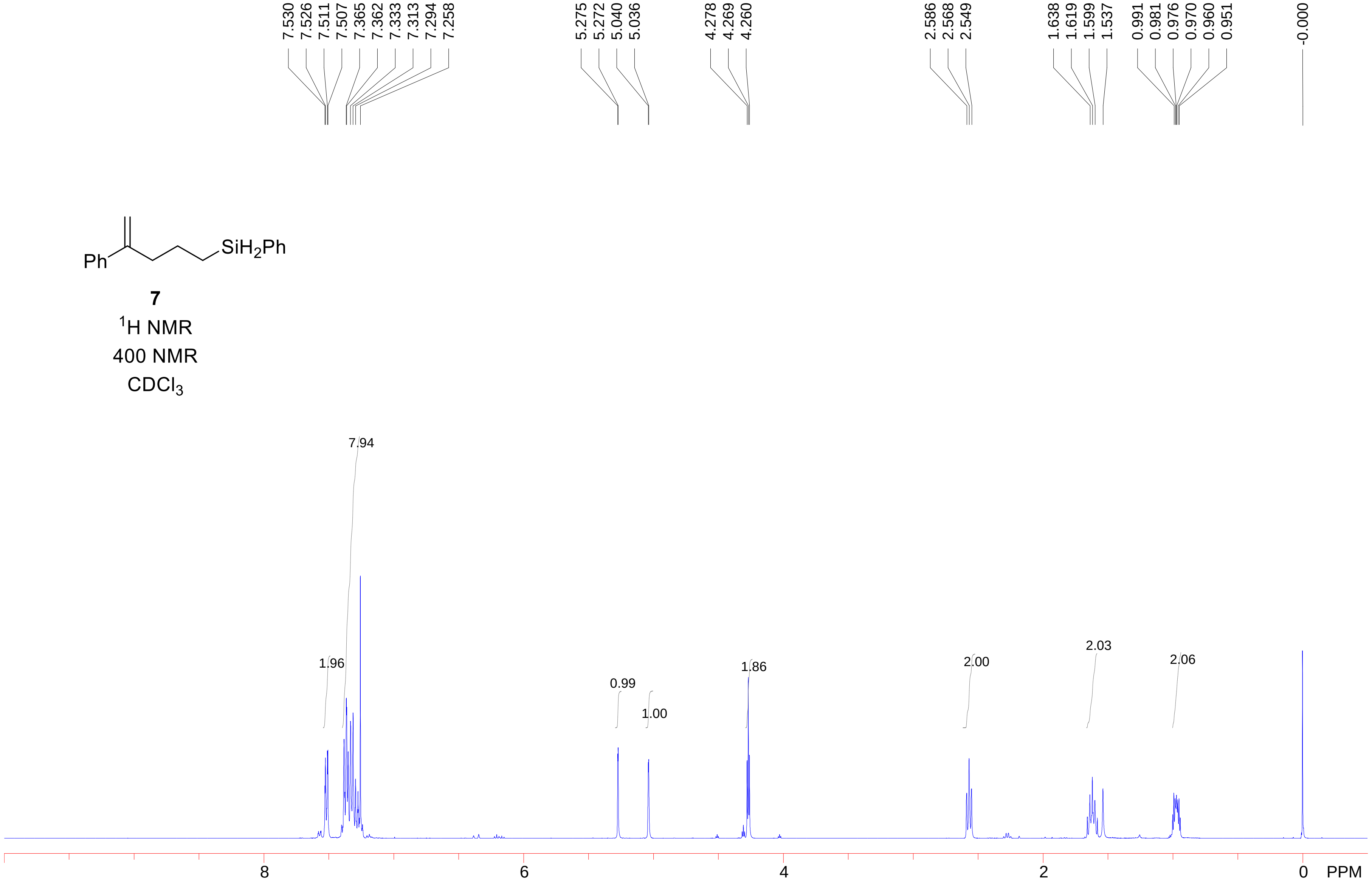

Supplementary Figure 113. ^{1}H NMR spectrum of **7**

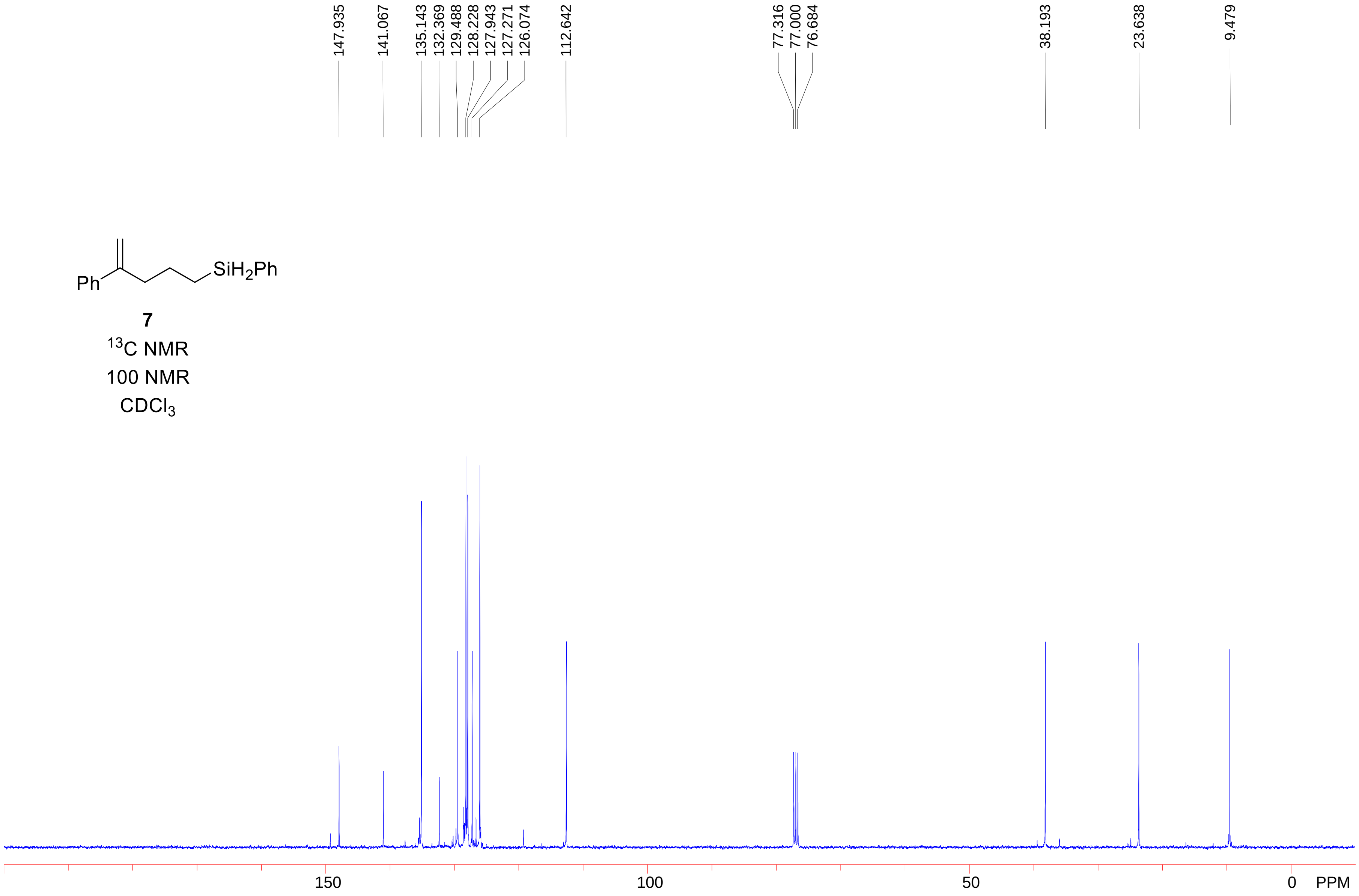

Supplementary Figure 114. ^{13}C NMR spectrum of **7**

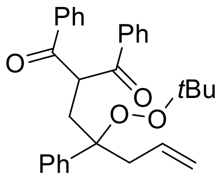

8

^{1}H NMR

400 MHz

$CDCl_3$

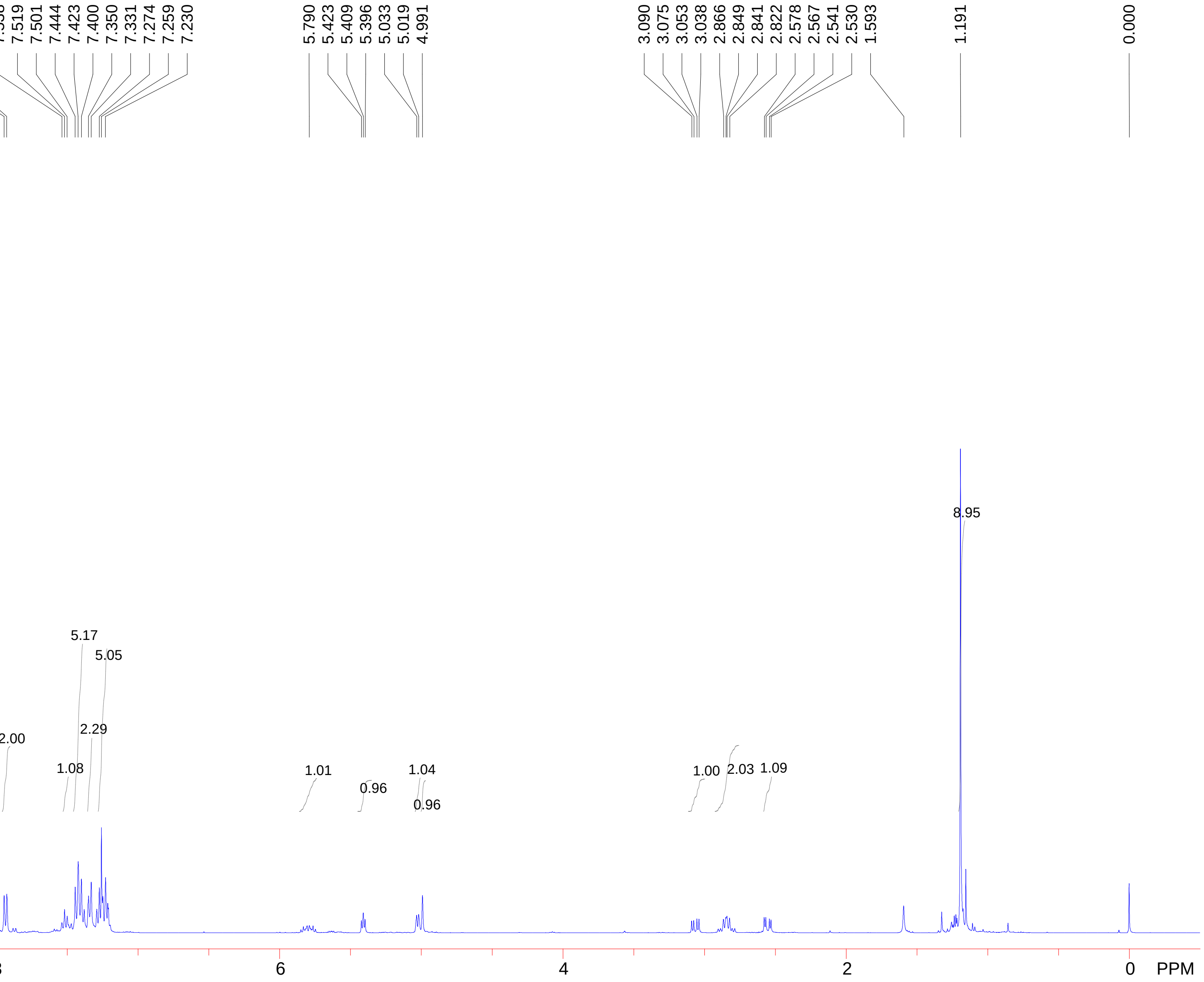

Supplementary Figure 115. ^{1}H NMR spectrum of **8**

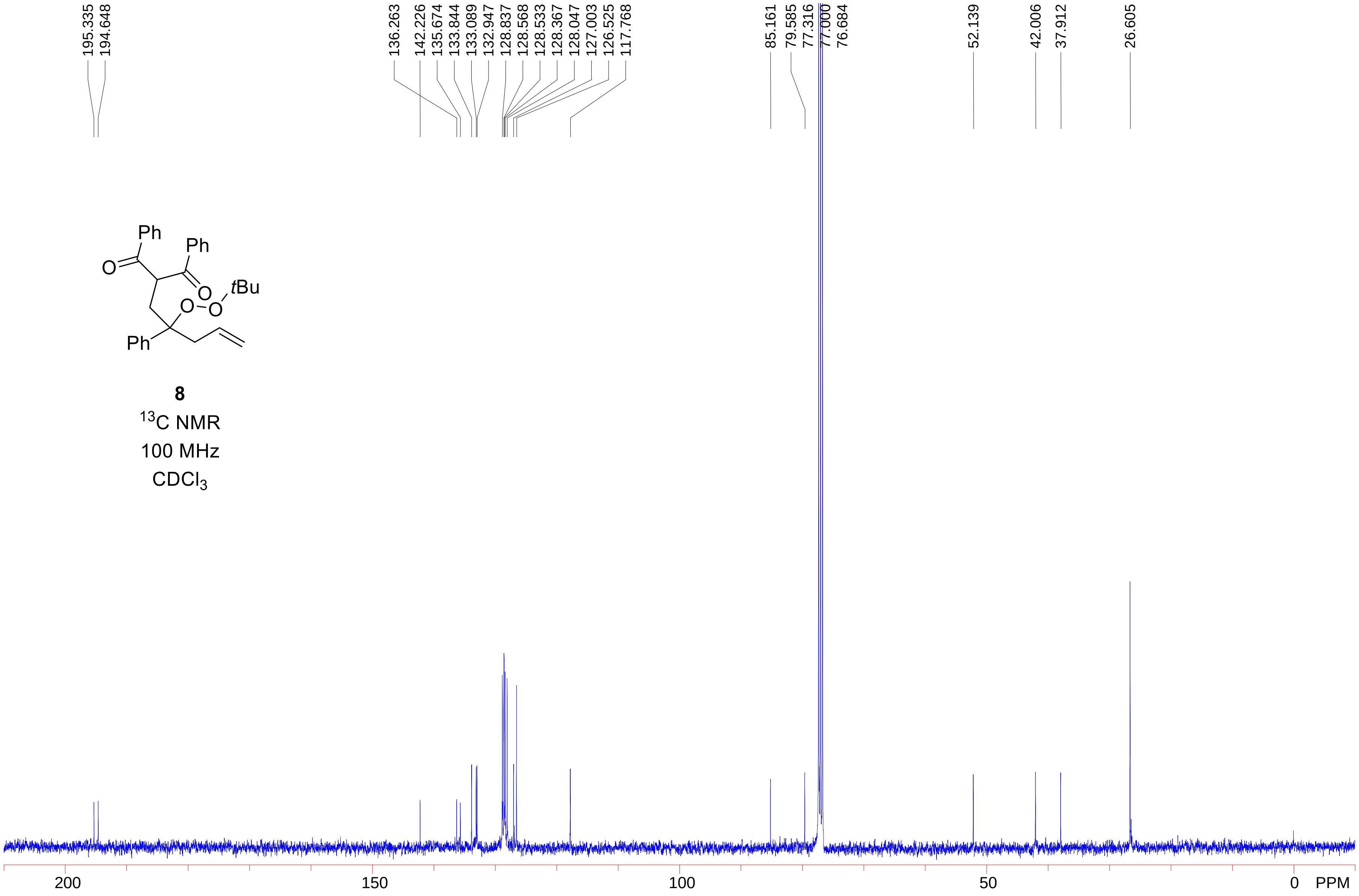

Supplementary Figure 116. ^{13}C NMR spectrum of **8**

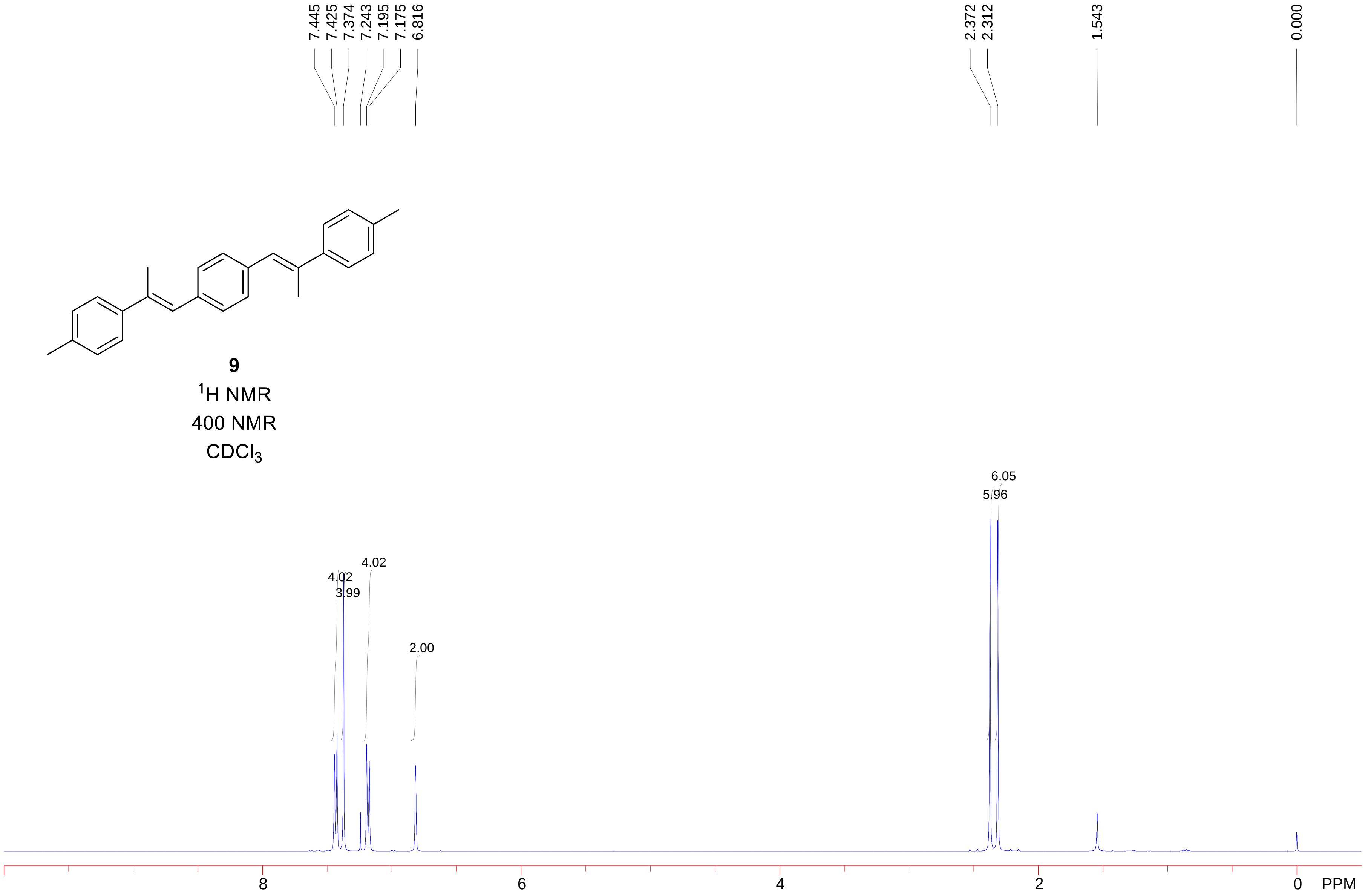

Supplementary Figure 117. ^{1}H NMR spectrum of **9**

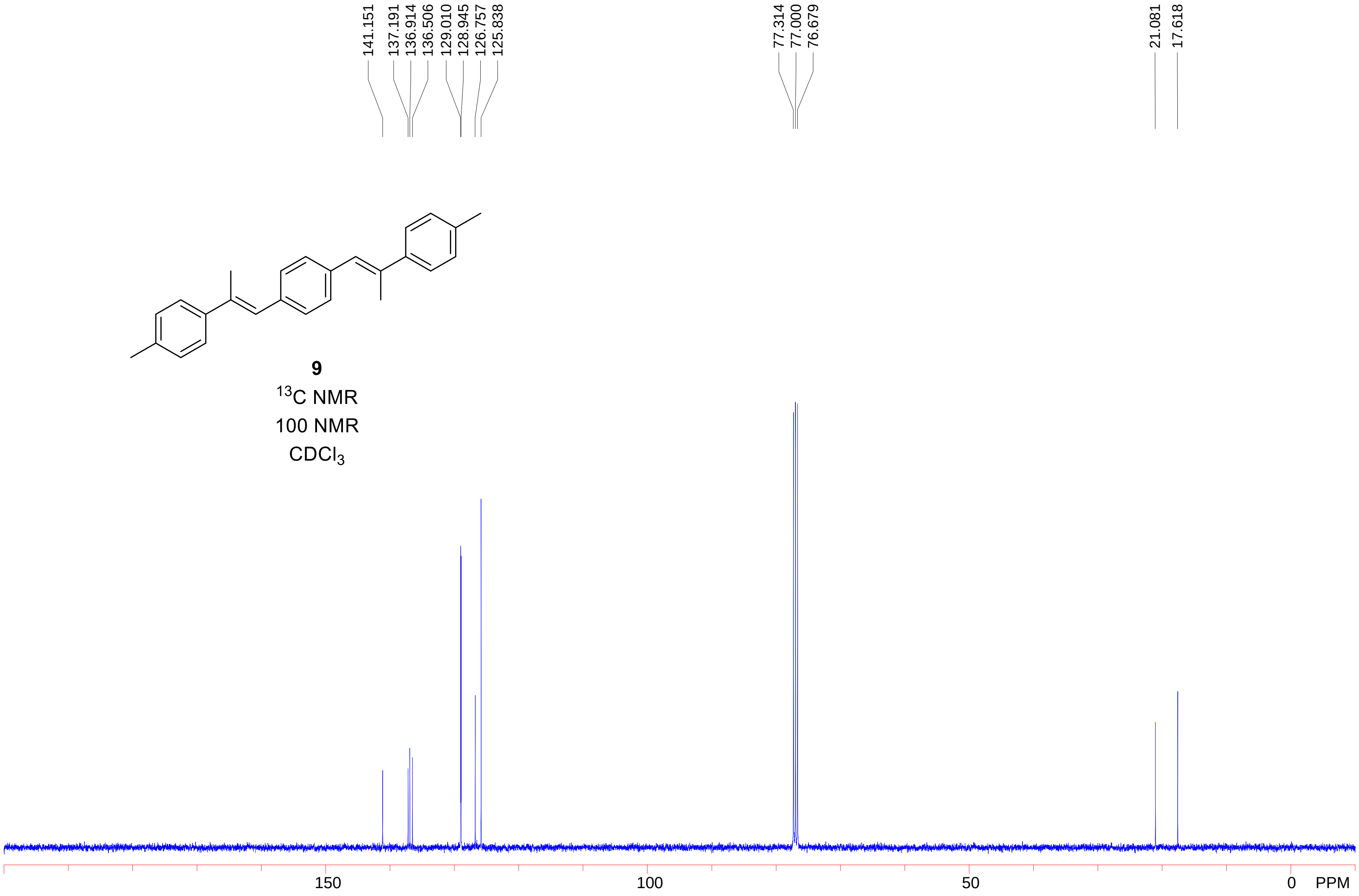

9

^{13}C NMR

100 NMR

$CDCl_3$

Supplementary Figure 118. ^{13}C NMR spectrum of **9**

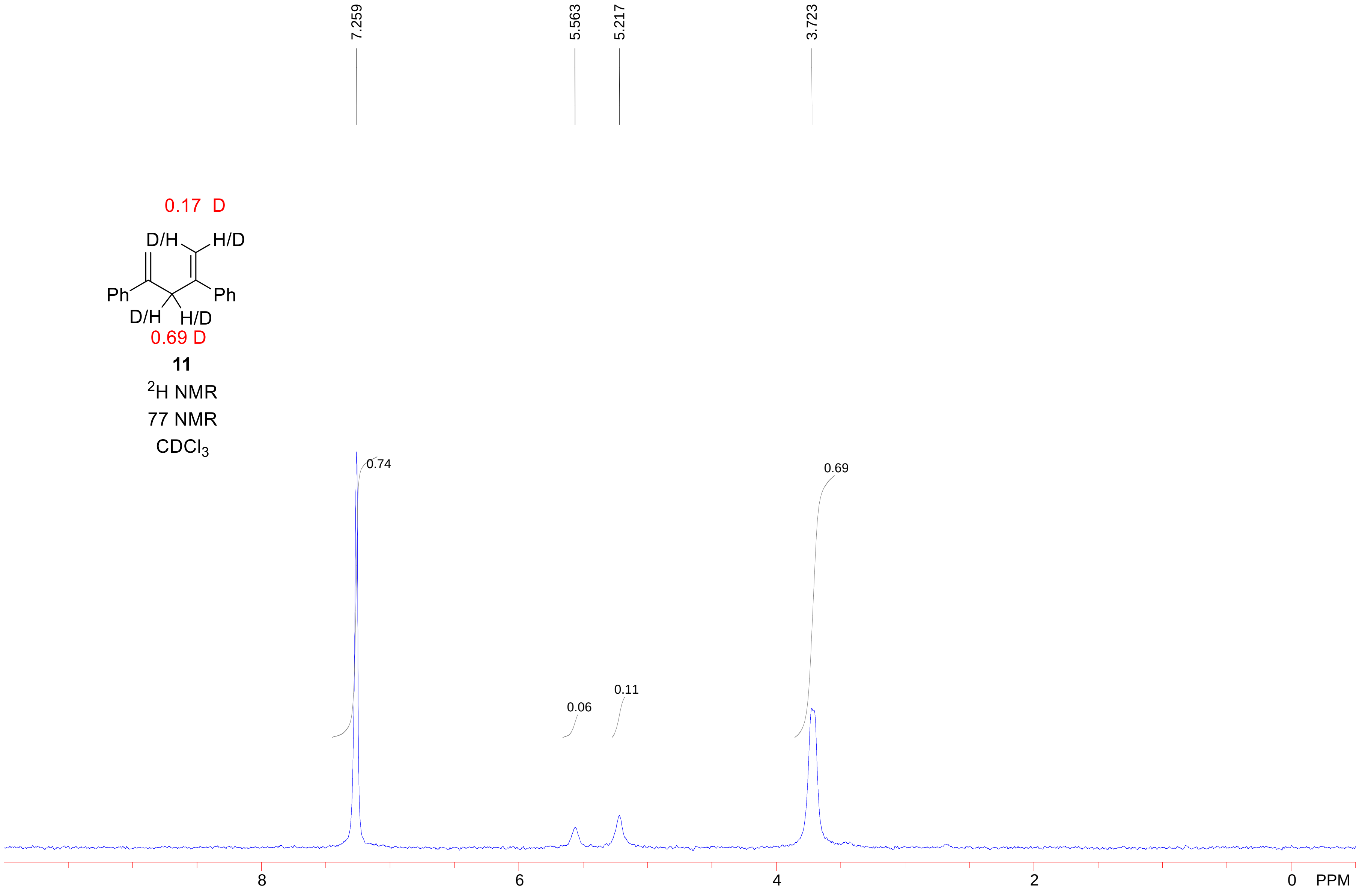

Supplementary Figure 119. ^{2}H NMR spectrum of **11**

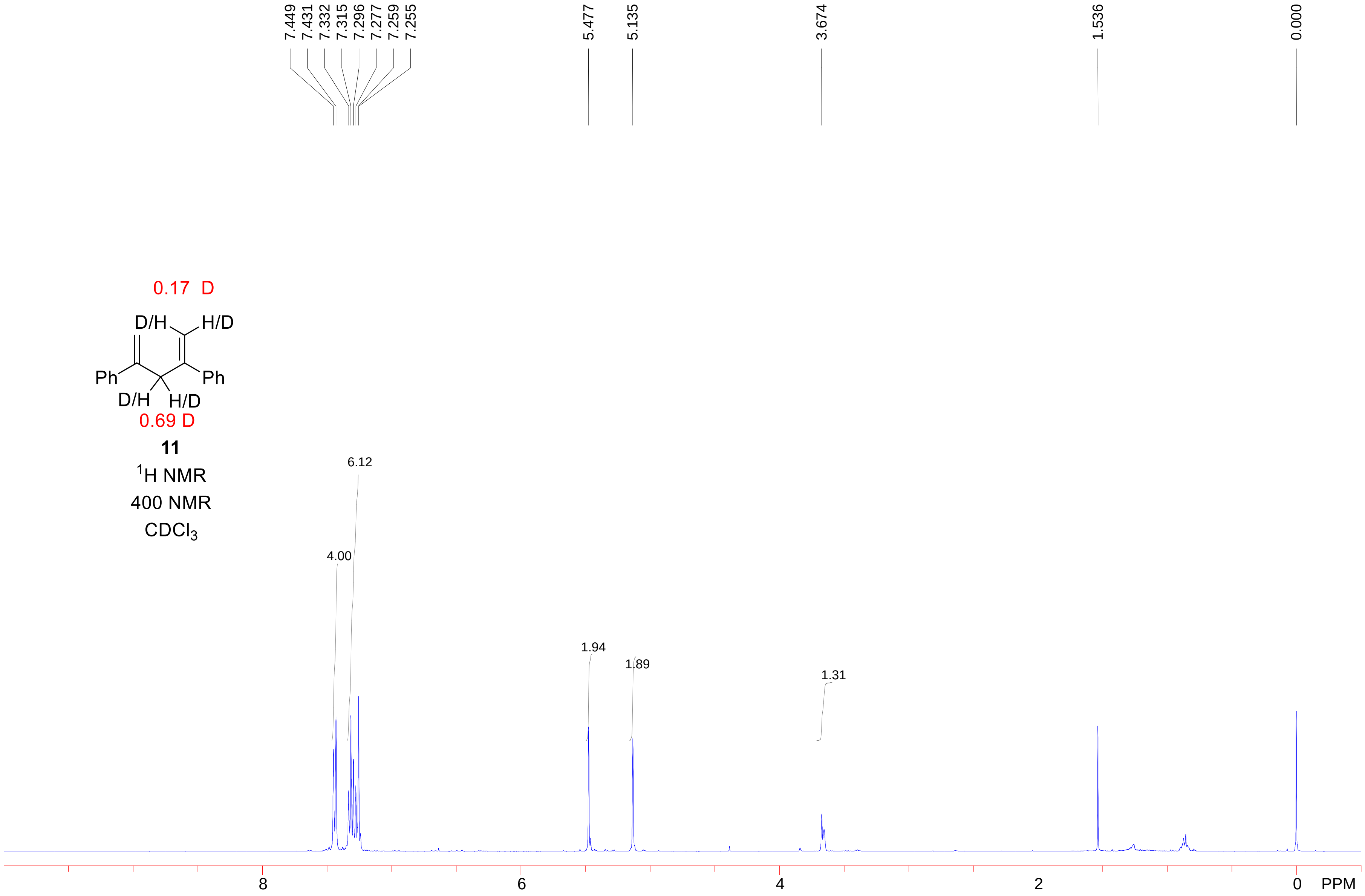

Supplementary Figure 120. ^{1}H NMR spectrum of **11**

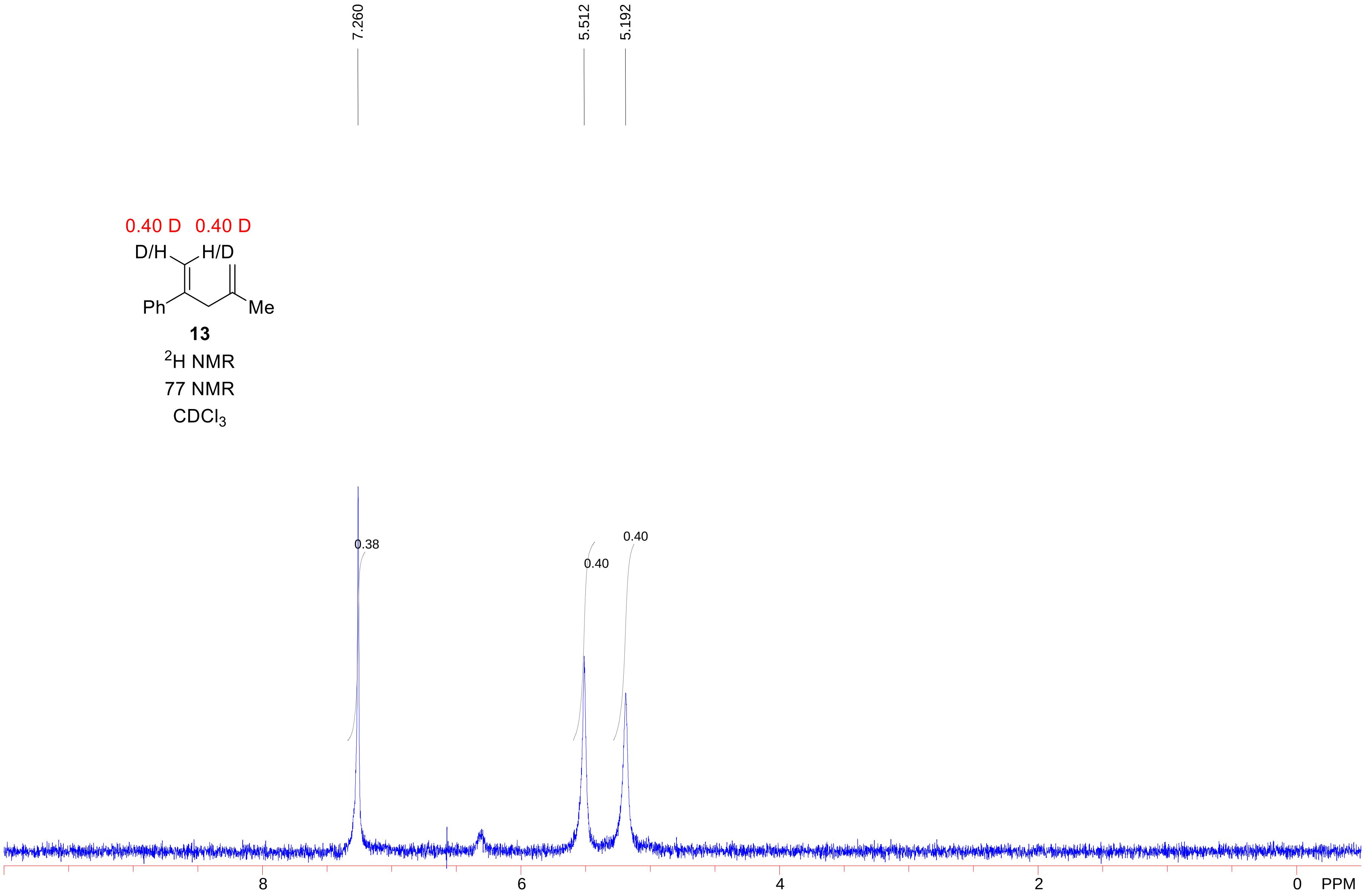

Supplementary Figure 121. ^{1}H NMR spectrum of **13**

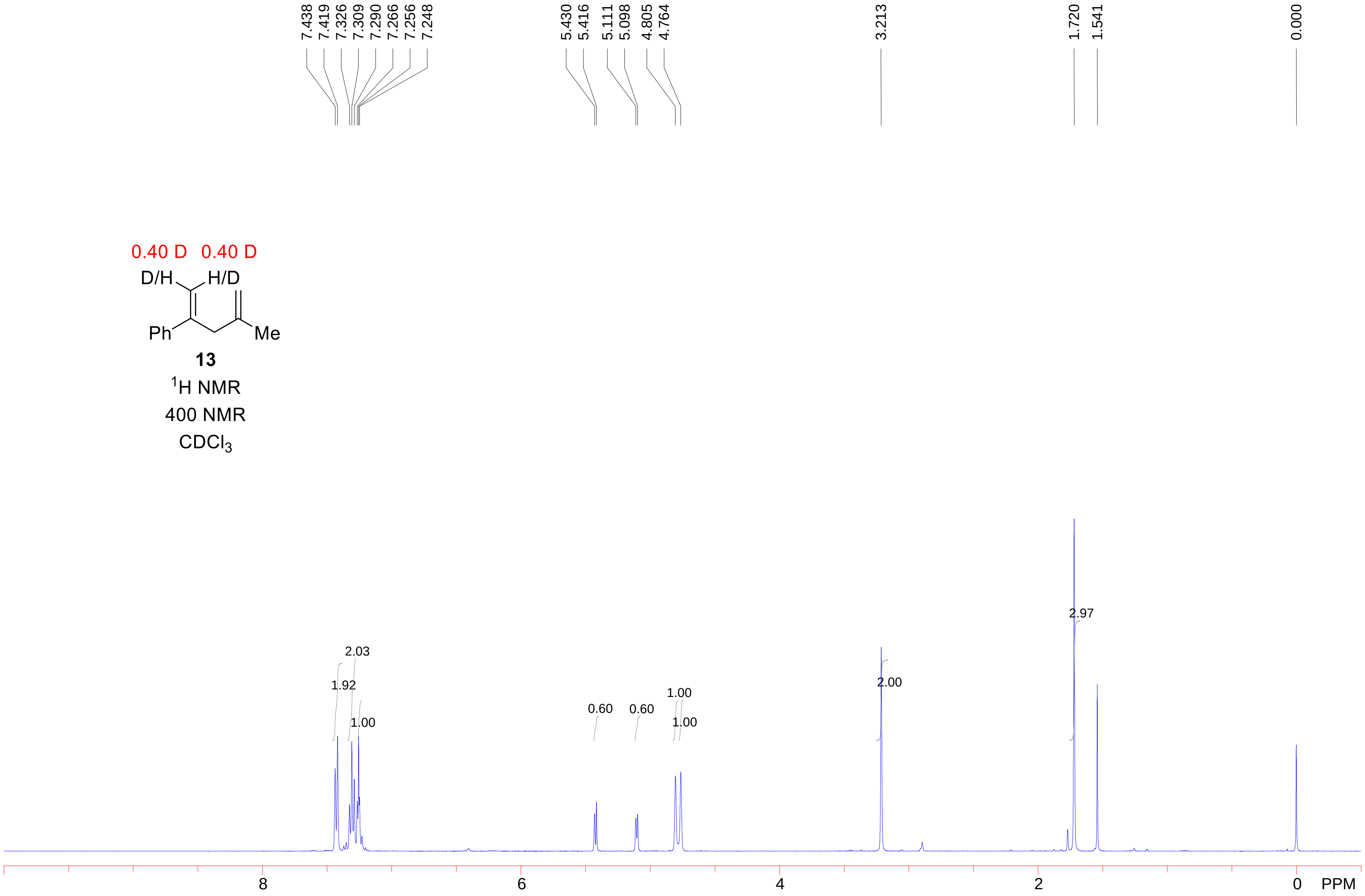

Supplementary Figure 122. ^{1}H NMR spectrum of **13**

Supplementary References

(1) Chen, J.; Shen, X.; Lu, Z. *Angew. Chem. Int. Ed.* **2021**, *60*, 690-694.

(2) Shen, X.; Chen, X.; Chen, J.; Sun, Y.; Cheng, Z.; Lu, Z. *Nat. Commun*. **2020**, *11*, 783.

(3) Kasten, K.; Slawin, A. M. Z.; Smith, A. D.; *Org. Lett*. **2017**, *19*, 5182-5185.

(4) Rami, F.; Bächtle, F.; Plietker, B.; *Catal. Sci. Technol.* **2020**, *10*, 1492-1497.

(5) Allen, C. P.; Benkovics, T.; Turek, A. K.; Yoon, T. P.; *J. Am. Chem. Soc.* **2009**, *131*, 12560-12561.

(6) Taher, D.; Guest, P.; Benton, A.; Ma, X.; Banwell, M. G.; Willis, A. C.; Seiser, T.; Newton, T. W.; Hutzler, J.; *J. Org. Chem.* **2017**, *82*, 211-233.

(7) Guo, J.; Shen, X.; *Angew. Chem. Int. Ed.* **2017**, *56*, 615-618.

(8) Henrion, G.; Chavas, T. E. J.; Goff, X. L.; Gagosz, F.; *Angew. Chem. Int. Ed.* **2013**, *52*, 6277-6282.

(9) Smith, C. A.; Motika, S. E.; Wojtas, L.; Shi, X.; *Chem. Commun*. **2017**, *53*, 2315-2318.

(10) He, J.; Xue, Y.; Han, B.; Zhang, C.; Wang, Y.; Zhu, S. *Angew. Chem. Int. Ed.* **2020**, *59*, 2328-2332.

(11) Sugano, Y.; Kikuchi, F.; Toita, A.; Nakamura, S.; Hashimoto, S. *Chem. Eur. J.* **2012**, *18*, 9682-9690.

(12) He, L.; Byun, H.; Smit, J.; Wilschut, J.; Bittman, R.; *J. Am. Chem. Soc.* **1999**, *121*, 3897-3903.

(13) Hazelden, I. R.; Carmona, R. C.; Langer T.; Pringle, P. G.; J. F. Bower. *Angew. Chem. Int. Ed.* **2018**, 57, 5124-5128.

(14) Shuler, S. A.; Yin, G.; Krause, S. B.; Vesper, C. M.; Watson, D. A. *J. Am. Chem. Soc.* **2016**, *138*, 13830-13833.

(15) Ragoussi, M.; Walker, S. M.; Piccanello, A.; Kariuki, B. M.; Horton, P. N.; Spencer, N. Snaith, J. S. *J. Org. Chem.* **2010**, *75*, 7347-7357.

(16) Sloane, S.; Reyes, A.; Vang, Z.; Li, L.; Behlow, K.; Clark, R. *Org. Lett.* **2020**, *22*, 9139-9144.

(17) Lu, Z.; Ma, S. *J. Org. Chem.* **2006**, *71*, 2655-2660.

(18) Garzan, A.; Jaganathan, A.; Marzijarani, N. S.; Yousefi, R.; Whitehead, D. C.; Jackson, J. E.; Borhan, B. *Chem. Eur. J.* **2013**, *19*, 9015-9021.

(19) Shin, K.; Joung, S.; Kim, Y.; Chang, S. *Adv. Synth. Catal.* **2017**, *359*, 3428-3436.

(20) Lee, K.; Lee, J.; Lee, P. *J. Org. Chem.* **2002**, *67*, 8265-8268.

(21) Zhang, S,; Bedi, D.; Cheng, L.; Unruh, D.; Li, G.; Findlater, M. *J. Am. Chem. Soc.* **2020**, *142*, 8910-8917.

(22) Chen, J.; Cheng, B.; Cao, M.; Lu, Z. *Angew. Chem. Int. Ed.* **2015**, *54*, 4661-4664.

(23) Wu, X.; Ding, G.; Lu, W.; Yang, L.; Wang, J.; Zhang, Y.; Xie, X.; Zhang, Z. *Org. Lett.* **2021**, *23*, 1434-1439.

(24) Lu, S.; Qi, L.; Li, Z. *Asian J. Org. Chem.* **2017**, *6*, 313-321.

(25) Zhao, J.; Cheng, B.; Chen, C.; Lu, Z.; *Org. Lett.* **2020**, *22*, 837-841.
